# Linked electronic health records for research on a nationwide cohort of more than 54 million people in England: data resource

**DOI:** 10.1136/bmj.n826

**Published:** 2021-04-07

**Authors:** Angela Wood, Rachel Denholm, Sam Hollings, Jennifer Cooper, Samantha Ip, Venexia Walker, Spiros Denaxas, Ashley Akbari, Amitava Banerjee, William Whiteley, Alvina Lai, Jonathan Sterne, Cathie Sudlow, CVD-COVID-UK consortium, Abdel Douiri, Abdul Qadr Akinoso-Imran, Adrian Jonas, Ajay Shah, Alex Handy, Alun Davies, Amanj Kurdi, Anna Hansell, Annemarie Docherty, Arun Pherwani, Ashkan Dashtban, Ben Bray, Ben Cairns, Ben Goldacre, Ben Humberstone, Bilal Mateen, Brett Doble, Brian Roberts, Carole Morris, Caroline Dale, Caroline Rogers, Charles Wolfe, Christopher Tomlinson, Claire Lawson, Clea du Toit, Colin Berry, Craig Smith, Dan O’Connell, Daniel Harris, David Brind, David Cromwell, David Hughes, David Moreno Martos, Debbie Ringham, Deborah Lawler, Deborah Lowe, Elena Nikiphorou, Eloise Withnell, Emanuele Di Angelantonio, Eva Morris, Ewan Birney, Fabian Falck, Fatemeh Torabi, Felix Greaves, Florian Falter, Francesco Zaccardi, Frank Kee, Gareth Davies, George Nicholson, Gwenetta Curry, Haoting Zhang, Harry Hemingway, Harry Wilde, Hoda Abbasizanjani, Honghan Wu, Howard Tang, Huan Wang, Ify Mordi, Jackie MacArthur, Jane Lyons, Jennifer Beveridge, Jessica Barrett, Jianhua Wu, Johan Thygesen, John Danesh, John Dennis, Jon Boyle, Julian Halcox, Kamlesh Khunti, Kate Cheema, Katherine Brown, Ken Li, Kim Kavanagh, Laura North, Laura Pasea, Libby Ellins, Livia Pierotti, Lucy Wright, Lydia Martin, Lynn Morrice, Mamas Mamas, Marion Bennie, Mark Barber, Mary Joan Macleod, Massimo Caputo, Maya Buch, Mehrdad Mizani, Michalis Katsoulis, Mike Gravenor, Mike Inouye, Mirek Skrypak, Moritz Gerstung, Munir Pirmohamed, Myer Glickman, Naomi Herz, Neil Davies, Nick Hall, Nilesh Samani, Olena Seminog, Paula Lorgelly, Pedro Machado, Qiuju Li, Rachel Denholm, Raph Goldacre, Raymond Carragher, Reecha Sofat, Rohan Takhar, Ronan Lyons, Rouven Priedon, Rowena Griffiths, Rupert Payne, Ruwanthi Kolamunnage-Dona, Safa Salim, Sandosh Padmanabhan, Sarah Onida, Seamus Kent, Seb Bacon, Sinduja Manohar, Sonya Babu-Narayan, Spencer Keene, Susheel Varma, Thomas Lawrence, Tianxiao Wang, Tim Wilkinson, Tom Norris, Tom Palmer, Vahé Nafilyan

**Affiliations:** 1 British Heart Foundation Cardiovascular Epidemiology Unit, Department of Public Health and Primary Care, University of Cambridge, Cambridge, UK; 2 British Heart Foundation Centre of Research Excellence, University of Cambridge, Cambridge, UK; 3 Health Data Research UK Cambridge, Wellcome Genome Campus and University of Cambridge, Cambridge, UK; 4 National Institute for Health Research Blood and Transplant Research Unit in Donor Health and Genomics, University of Cambridge, Cambridge, UK; 5 The Alan Turing Institute, London, UK; 6 Population Health Sciences, Bristol Medical School, University of Bristol, Bristol, UK; 7 Health Data Research UK, South West Better Care Partnership, Bristol, UK; 8 National Institute for Health Research Bristol Biomedical Research Centre, University of Bristol, Bristol, UK; 9 NHS Digital, Leeds, UK; 10 University of Pennsylvania Perelman School of Medicine, Philadelphia, PA, USA; 11 MRC University of Bristol Integrative Epidemiology Unit, Bristol, UK; 12 British Heart Foundation Research Accelerator, University College London, London, UK; 13 Institute of Health Informatics, University College London, London, UK; 14 National Institute for Health Research University College London Hospitals Biomedical Research Centre, University College London, London, UK; 15 Population Data Science and Health Data Research UK, Swansea University, Swansea, UK; 16 Barts Health NHS Trust, The Royal London Hospital, London, UK; 17 University College London Hospitals NHS Trust, London, UK; 18 Centre for Clinical Brain Sciences, University of Edinburgh, Edinburgh, UK; 19 Nuffield Department of Population Health, University of Oxford, Oxford, UK; 20 BHF Data Science Centre, Health Data Research UK, London, UK; 21 Usher Institute, Edinburgh Medical School, University of Edinburgh, Edinburgh, UK

## Abstract

**Objective:**

To describe a novel England-wide electronic health record (EHR) resource enabling whole population research on covid-19 and cardiovascular disease while ensuring data security and privacy and maintaining public trust.

**Design:**

Data resource comprising linked person level records from national healthcare settings for the English population, accessible within NHS Digital’s new trusted research environment.

**Setting:**

EHRs from primary care, hospital episodes, death registry, covid-19 laboratory test results, and community dispensing data, with further enrichment planned from specialist intensive care, cardiovascular, and covid-19 vaccination data.

**Participants:**

54.4 million people alive on 1 January 2020 and registered with an NHS general practitioner in England.

**Main outcome measures:**

Confirmed and suspected covid-19 diagnoses, exemplar cardiovascular conditions (incident stroke or transient ischaemic attack and incident myocardial infarction) and all cause mortality between 1 January and 31 October 2020.

**Results:**

The linked cohort includes more than 96% of the English population. By combining person level data across national healthcare settings, data on age, sex, and ethnicity are complete for around 95% of the population. Among 53.3 million people with no previous diagnosis of stroke or transient ischaemic attack, 98 721 had a first ever incident stroke or transient ischaemic attack between 1 January and 31 October 2020, of which 30% were recorded only in primary care and 4% only in death registry records. Among 53.2 million people with no previous diagnosis of myocardial infarction, 62 966 had an incident myocardial infarction during follow-up, of which 8% were recorded only in primary care and 12% only in death registry records. A total of 959 470 people had a confirmed or suspected covid-19 diagnosis (714 162 in primary care data, 126 349 in hospital admission records, 776 503 in covid-19 laboratory test data, and 50 504 in death registry records). Although 58% of these were recorded in both primary care and covid-19 laboratory test data, 15% and 18%, respectively, were recorded in only one.

**Conclusions:**

This population-wide resource shows the importance of linking person level data across health settings to maximise completeness of key characteristics and to ascertain cardiovascular events and covid-19 diagnoses. Although this resource was initially established to support research on covid-19 and cardiovascular disease to benefit clinical care and public health and to inform healthcare policy, it can broaden further to enable a wide range of research.

## Introduction

The covid-19 pandemic has increased awareness of the importance of population-wide person level electronic health record (EHR) data from a range of sources for examining, modelling, and reporting disease trends to inform healthcare and public health policy.^1^ Key benefits of research using such data on nationwide cohorts include generalisability of findings across all age groups, ethnicities, geographical locations, and socioeconomic, health, and personal characteristics, and inclusion of large numbers of people and events, enhancing the precision of findings and enabling a wide spectrum of novel research studies (eg, characterising shapes of relations between risk factors and disease, or studying minority groups and rare disease subtypes). While EHRs for whole country cohorts for Wales, Scotland, Denmark, and Sweden (populations of 3 to 10 million) have been used in research for several years,^2^ ^3^ ^4^ ^5^ ^6^ at the start of the covid-19 pandemic, researchers had no access to national linked healthcare data across the population of England to enable critical research to support healthcare decisions and public health policy. There were two main reasons for this: the collection of comprehensive, linkable primary care data did not exist nationally and there was no secure, privacy protecting mechanism for researchers to access and conduct population-wide research using national datasets linked across different parts of the health data system (eg, from primary care, hospitals, death registries, laboratories). EHR research in England to date has therefore not been able to take advantage of the statistical power of studying a population of almost 60 million people, and clinical, public health, and policy insights have directly represented only a subset of the population. Hence there remains a need for accessible, nationwide health data in England for research, while ensuring participant safety and maintaining public trust.

Motivated by the public health importance of fully understanding the relation between covid-19 and cardiovascular disease (CVD), the British Heart Foundation (BHF) Data Science Centre^7^ established the CVD-COVID-UK initiative.^8^ This partnership with national health data custodians in the four nations of the UK aims to provide linked, nationally collated EHRs for the whole population of the UK for approved research within secure, privacy protecting environments. Although established initially to support research into the impact of CVDs, and related treatment and risk factors, on covid-19 and the impact of covid-19 on CVDs, these linked EHR resources will, with appropriate ethical and regulatory approvals, be able to support a wide range of research studies. These could include investigations of the links between the full spectrum of risk factors and health states documented in EHRs and covid-19, the impact of the pandemic on health service activity and provision for non-covid-19 conditions, the nature and determinants of long covid, and the benefits and risks of covid-19 vaccination. Research possibilities also include myriad studies of associations between risk factors and health outcomes beyond the pandemic, health and disease surveillance, quality assurance of health services, and planning recruitment to and follow-up in randomised trials.

In this paper we describe key features of the new English component of this UK-wide effort: a nationwide linked health data resource, provided within a new trusted research environment for England, developed in partnership with NHS Digital.^9^ We use descriptive analyses of the currently available data to illustrate the importance for whole population research studies of linking EHRs from across different health settings.

## Methods

### Data resources

**Fig 1:**
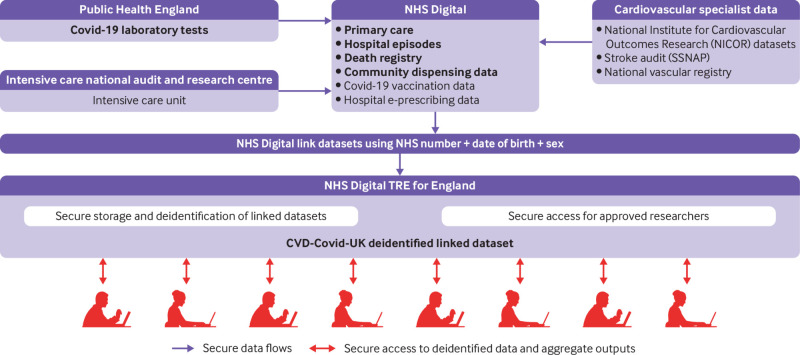
Overview of current (in bold) and planned data flows into NHS Digital Trusted Research Environment (TRE) for England

The newly established NHS Digital Trusted Research Environment (TRE) for England provides researchers with secure, remote access to linked, person level EHR data from national health settings. The data sources currently available include primary care data, hospital episodes (covering inpatient, outpatient, emergency department, and critical care episodes), registered deaths (including causes of death), covid-19 laboratory tests, and community dispensed medicines (table 1, fig 1, CVD-COVID-UK Dataset dashboard,^10^ CVD-COVID-UK Dataset trusted research environment asset in Health Data Research Innovation Gateway).^11^ Further incorporation of specialist intensive care, cardiovascular audit, hospital electronic prescribing, and covid-19 vaccination data are planned soon.

**Table 1:** Overview of available and planned linked resources

Availability of population-wide linked data, and data description	Data resource
**Available January 2021**	
Primary care	GDPPR: General Practice Extraction Service (GPES) data for pandemic planning and research
Hospital episodes	Secondary Uses Service (SUS+) and Hospital Episode Statistics (HES) including: Emergency Care Dataset (ECDS), admitted participant care, adult critical care, and outpatients
Death registry	Office for National Statistics (ONS) death registrations
Laboratory tests for covid-19	Public Health England (PHE) Second Generation Surveillance System (SGSS) test data results for covid-19 (pillars 1 and 2)
Community dispensing data	NHS Business Services Authority (BSA) community dispensing data
**To become available during 2021**	
Intensive care unit	Intensive Care National Audit and Research Centre (ICNARC) data
Cardiovascular specialist audit and registry data	National Institute for Cardiovascular Outcomes Research (NICOR) datasets including: myocardial infarction national audit programme, adult percutaneous coronary interventions, national heart failure audit, cardiac rhythm management audit, congenital heart disease in children and adults, adult cardiac surgery audit, NICOR health technology registries Sentinel Stroke National Audit Programme (SSNAP) data National Vascular Registries (NVR) data
National covid-19 vaccination data	National immunisation management system covid-19 vaccination dataset Adverse reactions dataset for covid-19 vaccination
Hospital electronic prescribing data	

Supplementary figure 1 shows how data generated in hospitals, general practices, community pharmacies, covid-19 NHS and commercial testing services, covid-19 vaccination services, and registry offices flow to NHS Digital. Regular flows to NHS Digital of specialist intensive care and cardiovascular audit data from several different providers were established for the first time during 2020. Furthermore, the new primary care dataset established during spring 2020 by the GP Extraction Service, is the most comprehensive yet to flow to NHS Digital.

### Data processing and linkage

The processing and quality checks applied by data providers and processors before arriving into and within NHS Digital vary by dataset (see notes accompanying supplementary figure 1 for full details). Linkage between datasets is enabled by NHS Digital’s Master Person Service,^12^ which aims to match multiple records with 99% accuracy for each person from different clinical computer systems (eg, hospitals and general practices) to a single unique identifier, the National Health Service number representing a single person. NHS numbers that are included in records are cross checked with associated personal details, including age, sex, and postcode, within the Personal Demographics Service. If the NHS number is verified, no further processing is required. If the NHS number cannot be verified or is not provided, the Master Person Service then attempts to match the records to a single NHS number held in the Personal Demographics Service. The Master Person Service checks the personal details in the submitted data file for closeness to the data held in the Personal Demographics Service and produces an associated match confidence score. Each unique person specific NHS number is replaced with a non-identifying unique master key (or pseudo-identifier), which enables linkage of people’s records between datasets. At present this score is not provided at person level within each dataset, although work is ongoing within NHS Digital to enable this in the future. However, NHS Digital’s monthly reports for data quality maturity index indicate that 97-100% of records submitted to NHS Digital each month include information on NHS number and other key personal variables, providing confidence in the accuracy of the matching process.^13^

### Data resource access: NHS Digital TRE for England

On behalf of the CVD-COVID-UK consortium, the BHF Data Science Centre requested access to the data sources through the NHS Digital online Data Access Request Service^14^ and received approval for the CVD-COVID-UK research programme (reference No DARS-NIC-381078-Y9C5K) following discussion with NHS Digital’s Independent Group Advising on the Release of Data (IGARD).^15^ A data sharing agreement with NHS Digital allows approved researchers based in UK research organisations (universities and NHS bodies) that jointly sign this agreement to access the data held within the NHS Digital TRE service for England.^16^ The BHF Data Science Centre coordinates an approvals and oversight board (including representation from NHS Digital, participating research organisations, and lay members), which ensures that research projects undertaken fall within the scope of the ethical and regulatory approvals for the CVD-COVID-UK consortium programme. The trusted research environment provides secure storage and remote data access, avoiding the need for any person level data to leave NHS Digital (fig 1).

The NHS Digital TRE team provides named, approved researchers with secure log in details for remote access. Users of the trusted research environment can access data within NHS Digital’s data platform, where they can interrogate, manage, analyse, and visualise data using Databricks, a collaborative analytics platform that supports SQL and Python, and R Studio, a data analysis environment for R. Other analytical tools (eg, Stata) are to be made available soon. Flexible cloud compute is available through Amazon Web Services. The CVD-COVID-UK consortium’s data management and methods group has created user guides that explain how to navigate the environment; show where to find the data, data dictionaries, collaborative and personal work spaces and folders, and spaces for imported files (eg, phenotype code lists from external code libraries); and provide rules for database etiquette. NHS Digital hosts a regular user forum to consult with users on further developments in the NHS Digital TRE and runs a service to deal with problems and queries from users.

### CVD-COVID-UK consortium: aims, membership, and principles

The CVD-COVID-UK consortium aims to use analyses of UK population-wide linked EHR data to investigate the effects of CVDs, related risk factors, and treatments on susceptibility to and poor outcomes from covid-19; the direct impact of SARS-CoV-2 infection on acute cardiovascular complications and longer term cardiovascular risk; and the indirect impact of the pandemic on the presentation, diagnosis, management, and outcomes of CVDs.^8^ Lay summaries of approved projects are published on the consortium’s web page.^8^ All consortium members (currently more than 130 people from 40 research or NHS organisations, including NHS data custodians) commit to: conducting research according to the “five safes”^17^; an inclusive approach that enables additional researchers to join the consortium as work evolves; and the open sharing of research protocols, analysis code, and phenotype code lists and algorithms (through the BHF Data Science Centre Github repository and, for phenotypes, the Health Data Research UK Phenotype Library).^18^ ^19^

Researchers based in UK research organisations, including universities, NHS bodies, and charities, can join the consortium provided they are willing and able to agree to its principles, and they can access the data within the NHS Digital TRE provided their organisation is willing and able to sign the joint data sharing agreement with NHS Digital. A streamlined mechanism for requesting information and potential data access is available through the Health Data Research UK Innovation Gateway.^11^ The consortium does not currently have any members based in non-UK organisations and has not yet explored data access for such researchers with NHS Digital, but in the meantime it welcomes contributions from the international research community through collaboration with UK based consortium members (see supplementary annexe 1).

As a result of demand from researchers, in particular those addressing questions of critical policy relevance for the UK government chief scientific adviser’s national core studies programme, established to coordinate the UK’s covid-19 research response,^20^ the BHF Data Science Centre is currently seeking to extend the ethics and regulatory approvals for the consortium’s research programme. This will enable it to cover a comprehensive range of covid-19-relevant research that can be conducted using national linked EHRs.

### Data updates

All the datasets are updated regularly within NHS Digital’s internal systems (between daily and fortnightly depending on the dataset) and have a variable lag behind real time at the point of update (table 2). The datasets are currently refreshed in the NHS Digital TRE on a synchronised monthly schedule, but more frequent updates (eg, weekly or daily) can be requested according to clinical, public health, and health policy research needs.

**Table 2:** Key details of main data resources

	Primary care		Hospital episodes			Death registry	Laboratory tests for covid-19	Community dispensing
Name of resource	GDPPR: General Practice Extraction Service (GPES) data for pandemic planning and research		Secondary Uses Service (SUS+)	Hospital Episode Statistics (HES) including: Emergency Care Dataset (ECDS), admitted participant care, adult critical care, outpatients		Civil Registration-Deaths (Office for National Statistics) asset)	Public Health England (PHE) Second Generation Surveillance System (SGSS) covid-19 test results	NHS Business Services Authority (BSA) community dispensing data
Who is included?	People registered with a general practice in England, without a registered objection to sharing of data with NHS Digital, alive on 1 November 2019		People receiving treatment or care at an NHS hospital in England	People receiving treatment or care at an NHS hospital in England		All people with a registered death in England	People with a laboratory confirmed polymerase chain reaction positive test result under pillar 1 or pillar 2 testing guidelines	People with at least one prescription dispensed in the community
What is recorded?	Personal characteristics, diagnoses, symptoms, signs, prescriptions, referrals, immunisations, behavioural factors, tests		Diagnoses, procedures, personal characteristics (including ethnicity and area level deprivation), admission and discharge dates, hospital and other variables	Diagnoses, procedures, personal characteristics (including ethnicity and area level deprivation), admission and discharge dates, hospital and other variables		Date of death, date death was registered, sex, underlying cause of death, district, subdistrict, place of death (code, establishment, and type)	Personal characteristics (age, sex, ethnicity, lower layer super output areas), date of specimen, laboratory report, reporting laboratory	Information on dispensed drugs (name, strength, substance, quantity)
How are records coded?	SNOMED-CT		ICD-10 (international classification of diseases, 10th revision), OPCS-4 (operating procedure codes, version 4)	ICD-10, OPCS-4, proprietary emergency care codes		ICD-10	Not coded	British National Formulary Dictionary of Medicines and Devices
Period of record dates	From the earliest record for each person to present		November 2019 to present	April 1997 to present		April 1997 to present	March 2020 to present	April 2018 to present
Frequency of provision, and time lag	Extracted fortnightly, up to date at time of each extract		Daily flows into NHS Digital, up to date on submission for completed episodes of care from submitting trusts	Updated monthly (from SUS) within NHS Digital, about 2 months behind real time		Weekly flows into NHS Digital, up to date at time of provision	Provided daily to NHS Digital, up to date at time of provision	Updated monthly, about 7-11 weeks behind real time
No of people with records (before quality assurance exclusions)	57 908 487		7 153 569	61 958 690		14 643 921	884 311	44 546 519
Total No of records	4 937 121 423		2 781 364 103	365 438 996		18 815 693	1 160 138	2 796 440 797
No of people known to be alive on 1 January 2020	54 388 181		6 251 673	42 582 312		417 236	776 503	40 623 625
Total No of records among people alive on 1 January 2020	4 870 642 482		2 491 646 379	228 933 294		457 412	988 174	2 329 914 169

### Data security, privacy, and confidentiality

The data within the NHS Digital TRE are deidentified (ie, directly identifying data items, such as each person’s name, address, NHS number, and exact date of birth, are removed) and pseudonymised (ie, each unique person specific NHS number is replaced with a non-identifying unique master key). Postcodes are replaced with lower layer super output areas that can be converted to indices of multiple deprivation.^21^ NHS Digital also operates a “safe outputs” service: although approved researchers work with record level and person level data within the trusted research environment, only summary, aggregate results can be extracted, subject to approval through disclosure control processes and rules, which follow similar principles to those used by other established trusted research environments, such as the Secure Anonymised Information Linkage (SAIL) Databank for Wales^22^ and the Scottish National Data Safe Haven.^23^ This ensures that no output that might be placed in the public domain contains information that could be used either on its own or in conjunction with other data to identify a person.

### Derivation of participant characteristics and disease diagnoses

For descriptive analyses, we defined a linked cohort as all people in the primary care data known to be alive on 1 January 2020, excluding those who had either died before or were born on or after that date (as recorded in the death registry and in the primary care records, respectively). We censored follow-up on 31 October 2020, the latest common record date across the datasets. We defined eligible records within the hospital episodes, death registry, and covid-19 laboratory test results as those that could be linked by their unique master key to a person included in the primary care data.

We combined primary care and hospital episodes records (covering inpatient, outpatient, emergency department, and critical care episodes) from before the index date of 1 January 2020 to define key characteristics, including sex, age, and ethnicity (categorised into white, mixed, Asian and Asian British, black and black British, and other ethnic groups). For each characteristic, we extracted the most recent record from the primary care data if available, otherwise we used the most recent record from the hospital episodes records. Characteristics were classified as “unknown” for people with no records. Using previously validated phenotypes from the CALIBER resource,^24^ we defined previous diagnoses of myocardial infarction (yes or no), stroke or transient ischaemic attack (defined as ischaemic stroke, haemorrhagic stroke, unspecified stroke, or transient ischaemic attack) (yes or no), diabetes (yes or no), and obesity (yes or no) from Systematized Nomenclature of Medicine Clinical Terms (SNOMED-CT) concept codes in the primary care data and from ICD-10 (international classification of diseases, 10th revision) codes in the hospital episodes (main or secondary diagnostic code position in the admitted patient care component of the Hospital Episode Statistics data) recorded before 1 January 2020. For primary care phenotypes, we translated and expanded the phenotypes defined in Read Terms V2 to SNOMED-CT and cross referenced them with codes in the primary care dataset.^25^ Two clinicians independently reviewed all phenotype code lists and, when applicable, classified ICD-10 terms and SNOMED-CT concepts into prevalent or incident diagnoses (supplementary tables 1-4).^19^

We ascertained people with a first ever incident myocardial infarction or stroke or transient ischaemic attack as those with no diagnosis of myocardial infarction or stroke or transient ischaemic attack before 1 January 2020 and with a diagnosis SNOMED-CT or ICD-10 code appearing in the primary care data, hospital episodes (main or secondary diagnostic code position in the admitted patient care component of the Hospital Episode Statistics data), or death registry (underlying or contributing cause of death) between 1 January and 31 October 2020 (phenotype algorithms provided in supplementary tables 1 and 2).^19^

We ascertained people with a confirmed or suspected covid-19 diagnosis as those with a positive polymerase chain reaction or antigen test result from the covid-19 laboratory test data, with a specimen date on or before 31 October 2020; or with a covid-19 diagnosis SNOMED-CT concept code appearing in the primary care data, with event date on or before 31 October 2020; or with a diagnosis ICD-10 code appearing in the hospital episodes (main or secondary diagnostic code position in the admitted patient care component of the Hospital Episode Statistics), with admission date on or before 31 October 2020; or with a death registration including a diagnosis ICD-10 code (as underlying or contributing cause), with date of death on or before 31 October 2020. Supplementary table 5 provides definitions for all covid-19 phenotypes.^19^

This manuscript was prepared in accordance with the REporting of studies Conducted using Observational Routinely-collected Data (RECORD) guidance (see supplementary annexe 2).^26^

### Patient and public involvement

The lay panel of the UK National Institute for Health Research-BHF Cardiovascular Partnership reviews the CVD-COVID-UK programme every few months and provides feedback that informs ongoing and future research. In addition, lay people directly affected by CVD are members of the consortium and its approvals and oversight board, enabling cogeneration of research ideas and providing valuable perspective and input on research proposals, lay summaries, and research outputs.

## Results

### Overview of data resources

Table 2 provides an overview of the currently available primary care, hospital episodes, death registrations, covid-19 test data, and community dispensing data sources. Supplementary table 6 provides information on the data fields available, and definitions of these within each dataset, and supplementary table 7 summarises the codes included in the primary care dataset.

The primary care dataset includes healthcare information coded with SNOMED-CT concepts for all people registered with an English NHS general practice (excluding around 1.3 million people with a registered objection to their general practice records being provided to NHS Digital).^25^ The dataset includes data from 98% of all English general practices across all relevant general practice computer system suppliers (TPP, EMIS, In Practice Systems, and Microtest) and holds about 4.9 billion records on 54.4 million people alive on 1 January 2020 (>96% of the total population of England based on the UK Office for National Statistics mid-2019 population estimate for England of 56 286 961).^27^ Around 34 000 SNOMED codes are included (>90% of all those currently extracted for a wide range of purposes by NHS Digital’s GP Extraction Service), covering a broad range of diagnoses and procedures (from the start of each person’s records) along with laboratory results, physical measurements, clinical referrals, and prescriptions (supplementary table 7). Although more than 900 000 SNOMED codes are listed in UK and international releases, large numbers of these are either inactive or hardly used.

Administrative and clinical hospital episode data are available from both the Secondary Uses Service (SUS+) and Hospital Episode Statistics resources.^27^ These data include information on length of stay, diagnoses, and procedures during hospital admissions as well as on outpatient, emergency department, and critical care episodes. Diagnoses are coded with ICD-10 codes and procedures with OPCS-4 (operating procedure codes, version 4).^28^ The SUS+ resource contains raw data collected from NHS healthcare providers, representing the most up-to-date hospital episodes within NHS Digital (among hospitals making prompt and complete returns). These data are consolidated, validated, and cleaned monthly to form the Hospital Episode Statistics database.^29^ As a result, each month’s Hospital Episode Statistics data become available about two months behind real time. Thereafter, a fixed update is produced for each full year of Hospital Episode Statistics data. Among the 54.4 million people included in our linked cohort, the SUS+ dataset holds 2.5 billion records for 6.3 million people (from November 2019 onwards) and the Hospital Episode Statistics dataset holds 0.2 billion records for 42.3 million people (from 1997 onwards).

Death registration data^11^ flow daily to NHS Digital from the ONS civil registration dataset, including date, cause (coded with ICD-10), and place of death, and are available from April 1997. Deaths in England should be registered within five days of the date of death, although registration of a death is delayed in some situations.^30^ ^31^ Among the 54.4 million people in our linked cohort, 417 236 died on or before 31 October 2020.

The Second Generation Surveillance System (SGSS)^11^ is the national laboratory reporting system used in England to capture routine laboratory data on mainly infectious diseases, including SARS-CoV-2, and antimicrobial resistance. SGSS provides reports daily to NHS Digital on positive covid-19 test results (including the test date) fed directly from pillar 1 pathology laboratories (ie, established laboratories in hospitals for patients as well as NHS key workers) and indirectly from pillar 2 laboratories (ie, new, centralised, mostly privately run laboratories, created specifically for covid-19 testing for the wider population). By 31 October 2020, a total of 884 341 people had at least one positive covid-19 test result recorded in the SGSS covid-19 laboratory test dataset, with 776 503 (88%) linkable to the 54.4 million person cohort.

The community dispensing dataset, provided monthly to NHS Digital by the NHS Business Services Authority, contains person level information on NHS primary care prescriptions dispensed by community pharmacists, appliance contractors, and dispensing doctors in England, including the name and strength of drug coded from the British National Formulary Dictionary of Medicines and Devices.^32^ Among the 54.4 million person cohort, there were dispensed drug records for more than 40.6 million people and about 2.3 billion records (from April 2018).

### Personal characteristics and CVD incidence

**Fig 2:**
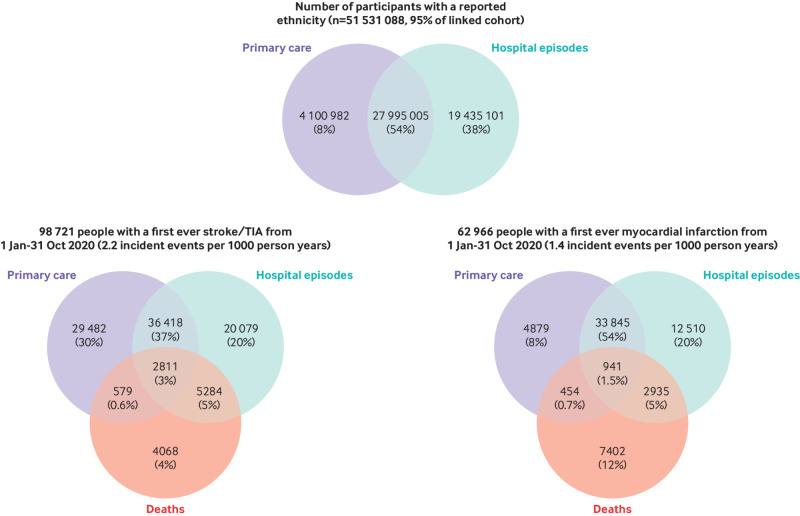
Data sources reporting person level data on ethnicity, incident stroke or transient ischaemic attack (TIA), and incident myocardial infarction

Table 3 shows the characteristics of the linked cohort of 54.4 million people alive on 1 January 2020; 51% were women and 14% were aged 70 years or older, with a mean age of 40.0 years for men and 41.6 years for women. By linking and combining person level records from primary care and hospital episodes, ethnicity information is available for approximately 95% of people, among whom 63% have their ethnic group recorded in primary care and 92% in hospital episodes data (fig 2). A previous diagnosis of stroke or transient ischaemic attack or of myocardial infarction is recorded for 2.2% and 2.1% of people, respectively, whereas 7% and 8% people have a record indicating a previous diagnosis of diabetes and obesity, respectively. Among 53.3 million people with no previous diagnosis of stroke or transient ischaemic attack, 98 721 had a first ever incident stroke or transient ischaemic attack between 1 January and 31 October 2020, 30% of which were recorded only in primary care (ie, not in hospital episodes or death registry data) and 4% only in death registry records (fig 2). Among 53.2 million people with no previous diagnosis of myocardial infarction, 62 966 had an incident myocardial infarction during follow-up, 8% of which were recorded only in primary care and 12% only in death registry records (fig 2).

**Table 3:** Characteristics of linked cohort and of people with a confirmed or suspected covid-19 diagnosis, by data resource. Values are numbers (percentages) unless stated otherwise

Characteristics	Total population (n=54 388 181)	Source of data on confirmed or suspected covid-19 diagnoses (% of subgroup)			
		Primary care records (n=714 162)	Laboratory (n=776 503)	Hospital episodes* (n=126 349)	Death registry (n=50 504)
Sex:					
Women	27 718 313 (51.0)	400 448 (1.4)	424 870 (1.5)	57 789 (0.2)	22 532 (0.1)
Men	26 661 385 (49.0)	313 558 (1.2)	351 383 (1.3)	68 329 (0.3)	27 716 (0.1)
Unknown	8483 (0.0)	156 (1.8)	250 (2.9)	231 (2.6)	256 (3.0)
Age group (years):					
0-17	11 188 814 (20.6)	60 571 (0.5)	68 028 (0.6)	1827 (0.0)	9 (0.0)
18-29	7 925 142 (14.6)	151 304 (1.9)	184 885 (2.3)	4081 (0.1)	70 (0.0)
30-49	14 701 289 (27.0)	207 672 (1.4)	226 179 (1.5)	15 828 (0.1)	891 (0.0)
50-69	13 026 860 (24.0)	179 977 (1.4)	181 399 (1.4)	35 070 (0.3)	6963 (0.1)
≥70	7 543 288 (13.9)	114 505 (1.5)	115 796 (1.5)	69 317 (0.9)	42 316 (0.6)
Unknown	2788 (0.0)	133 (4.8)	216 (7.7)	226 (8.1)	255 (9.1)
Ethnicity:					
White	41 786 891 (76.8)	556 489 (1.3)	588 550 (1.4)	99 629 (0.2)	43 328 (0.1)
Mixed	1 156 060 (2.1)	11 810 (1.0)	14 053 (1.2)	1748 (0.2)	433 (0.0)
Asian and Asian British	4 589 778 (8.4)	95 752 (2.1)	108 455 (2.4)	13 317 (0.3)	3143 (0.1)
Black and black British	1 860 340 (3.4)	20 863 (1.1)	25 051 (1.3)	6540 (0.4)	1684 (0.1)
Other	2 138 019 (3.9)	14 334 (0.7)	18 962 (0.9)	3221 (0.2)	996 (0.0)
Unknown	2 857 093 (5.3)	14 914 (0.5)	21 432 (0.8)	1894 (0.1)	920 (0.0)
Previous diagnoses:					
Stroke or transient ischaemic attack:					
No	53 191 717 (97.8)	685 197 (1.3)	745 978 (1.4)	108 596 (0.2)	39 427 (0.1)
Yes	1 196 464 (2.2)	28 965 (2.4)	30 525 (2.6)	17 753 (1.5)	11 077 (0.9)
Myocardial infarction:					
No	53 250 900 (97.9)	689 909 (1.3)	750 466 (1.4)	110 106 (0.2)	41 415 (0.1)
Yes	1 137 281 (2.1)	24 253 (2.1)	26 037 (2.3)	16 243 (1.4)	9089 (0.8)
Obesity:					
No	49 827 060 (91.6)	625 911 (1.3)	687 445 (1.4)	97 905 (0.2)	40 778 (0.1)
Yes	4 561 121 (8.4)	88 251 (1.9)	89 058 (2.0)	28 444 (0.6)	9726 (0.2)
Diabetes:					
No	50 778 499 (93.4)	642 095 (1.3)	700 133 (1.4)	88 262 (0.2)	33 283 (0.1)
Yes	3 609 682 (6.6)	72 067 (2.0)	76 370 (2.1)	38 087 (1.1)	17 221 (0.5)

* From Hospital Episode Statistics-admitted patient care data.

### Diagnoses of covid-19

**Fig 3:**
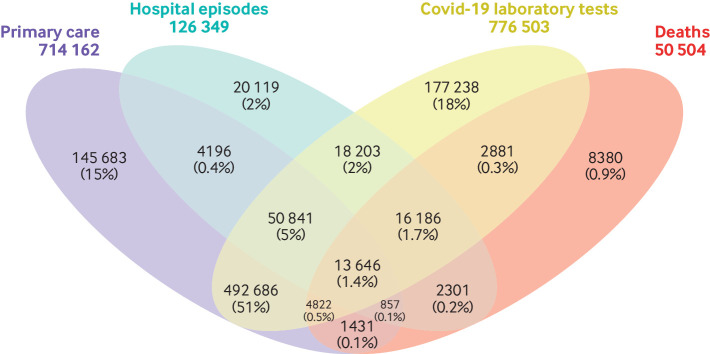
Data sources reporting person level data on confirmed or suspected covid-19 diagnoses between 1 January 2020 and 31 October 2020 (n=959 470). Numbers indicate distinct people with a confirmed or suspected covid-19 diagnosis

Among people in the linked cohort, 959 470 had a confirmed or suspected covid-19 diagnosis between 1 January and 31 October 2020 (714 162 in primary care data, 126 349 in hospital admission records, 776 503 in covid-19 laboratory test data, and 50 504 in death registry records). Although 58% of these were recorded in both primary care and covid-19 laboratory test data, 15% and 18%, respectively, were recorded in only one (fig 3).

Although women were more likely to have a confirmed or suspected covid-19 diagnosis in their primary care records (1.4% women *v* 1.2% men and in covid-19 laboratory test data (1.5% *v* 1.3%), they were less likely to have a covid-19 diagnosis recorded in hospital episodes (0.21% *v* 0.26%) or on death certificates (0.08% *v* 0.10%). Older people were more likely to have a covid-19 diagnosis from hospital episodes and death registrations, although young adults were more likely to have covid-19 diagnoses recorded in covid-19 laboratory test data and primary care. People with unknown age or sex were more than 10 times more likely to have a covid-19 diagnosis recorded in hospital episodes or on death certificates. A higher proportion of Asian and Asian British people had a covid-19 diagnosis in primary care and in the covid-19 laboratory test data compared with other ethnicities. Such differences were not, however, observed in information from hospital episodes or death certificates. People with a previous diagnosis of stroke or transient ischaemic attack, myocardial infarction, obesity, or diabetes were more likely to have a covid-19 diagnosis recorded in all healthcare settings (table 3).

When compared with the latest Public Health England reports of covid-19 laboratory test results,^33^ covid-19 related hospital admissions,^34^ and deaths with covid-19 on the death certificate,^35^ the data from our linked cohort concurs with those from relevant cumulative counts of people with covid-19 (supplementary table 8).

## Discussion

We have described the development and key features of a novel linked EHR resource comprising a range of current and future planned linked datasets covering the entire population of England and forming part of a UK initiative to accelerate UK-wide research on covid-19 and CVD and beyond. We include descriptive analyses of a cohort of 54.4 million people alive at the start of 2020, including more than 96% of the English population. The datasets described are already being accessed through the new NHS Digital TRE service for England to enable an expanding range of research projects through the BHF Data Science Centre’s CVD-COVID-UK consortium. Notably, combining person level information across data sources delivers about 95% complete data on key characteristics, including age, sex, and ethnicity, and is essential for ascertaining CVDs of interest, such as stroke and myocardial infarction. About 90% of people with a positive covid-19 laboratory test result have linkable primary care records, and enriching the covid-19 laboratory test data with primary care, hospital episodes, and death registry data enables ascertainment of about 20% additional people with confirmed or suspected covid-19.

### Comparison with other resources

Previously, research use of linked EHRs in England has been restricted to subsets of the population, according to the coverage of various data providers, including the individual primary care computer system suppliers (eg, Clinical Practice Research Datalink,^36^ Oxford-RCGP Research and Surveillance Centre,^37^ QResearch,^38^ and, more recently, OpenSafely^39^). Population coverage for each of these has increased during the pandemic. Supplementary table 9 provides a summary of the population based linked healthcare resources for England, which cover a population of more than five million people and include primary care data as an anchoring component. As the national provider of information, data, and IT systems for commissioners, analysts, and clinicians in health and social care in England, NHS Digital handles larger volumes of health data than any other organisation in the UK and has extremely well developed and robust processes for maintaining data security and privacy. Alignment of the new NHS Digital TRE for England with NHS Digital’s systems therefore maximises security while minimising the need for transmission of large volumes of linked data to support population scale research.

### Strengths and limitations of this study

The currently available linked data assets comprise, to our knowledge, the world’s largest single population based cohort available for research, which will be further enhanced as further datasets are added. The availability of primary care data linked to such a wide range of other data is unparalleled at this scale, while the resource is also for the first time making linked nationwide covid-19 laboratory testing and community dispensing data available for research. Given that the linked cohort comprises more than 96% coverage of the English population, it represents the English population in terms of age, sex, ethnicity, and diabetes when compared with UK government official statistics for England,^40^ ^41^ ^42^ includes the full distribution of general practices according to geographical location and size,^25^ and includes large enough numbers of people with different characteristics to support a diverse range of statistically well powered research studies. For example, the cohort includes large numbers of: people in subgroups typically underrepresented in research (eg, several tens of thousands in each of the ethnic minority groups); younger people for whom poor outcomes of covid-19 are uncommon but nonetheless devastating (eg, almost 20 million people younger than 30 years, among whom 79 covid-19 related deaths were recorded by 31 October 2020); people experiencing the common exemplar cardiovascular outcomes of stroke or transient ischaemic attack and myocardial infarction (many tens of thousands), suggesting substantial potential to support studies on the impact of covid-19 on subtypes of stroke and myocardial infarction as well as on a wide range of rare conditions.

The NHS Digital TRE for England ensures secure, privacy protecting storage of and access to large volumes of data, while minimising the expense and security risks of data travel. Provision of data in this way is enabling a broad programme of collaborative research, encompassing several projects, which would be challenging to justify under the data dissemination model but which meet the relevant ethics and data access requirements under the trusted research environment model. Researchers from many different organisations have been able to gain rapid access to the linked datasets through the CVD-COVID-UK consortium and its data sharing agreement with NHS Digital, avoiding lengthy and costly processes for multiple separate organisational data access approvals and agreements. The consortium is enabling collaboration among researchers from across the UK, with a wide range of expertise (including clinicians from many different specialist backgrounds, data managers, computer scientists, data wranglers, epidemiologists, and biostatisticians). Furthermore, the consortium has encouraged productive interactions between researchers and NHS Digital staff (including project management, data management, data science, and technical development teams), enabling joint approaches to developing the trusted research environment service and to identifying and solving data provision and linkage challenges. The rich and diverse nature of this interdisciplinary collaboration supports clinically and methodologically informed data curation and analysis pipelines, and it will enhance the interpretation and clinical application of research outputs. Regular dataset updates ensure the contemporary relevance and dynamic nature of the data resource and will enable ongoing long term follow-up of the whole population. The development of publicly shareable, validated phenotyping algorithms^19^ and analytical code^18^ will avoid duplication of effort by additional groups of researchers working with the same or similar datasets. Although developed with the initial intent of supporting the CVD-COVID-UK consortium research programme, the NHS Digital TRE service for England has wider benefits, given its clear potential to expand to support research more broadly beyond covid-19 and beyond the cardiovascular domain. In addition, the work to establish the trusted research environment has generated knowledge about linked EHR data and routes to data access across the UK health data science research community, benefiting other UK-wide initiatives, including the International Severe Acute Respiratory and Emerging Infection Consortium (ISARIC) study on the clinical characteristics of people admitted to hospital with covid-19,^43^ collaborative efforts to address the determinants of covid-19 susceptibility, severity, and outcome through analyses of population based cohorts with biosamples linked to national EHRs^44^ ^45^; the Randomised Evaluation of COVID-19 Therapy (RECOVERY) trial of treatments for covid-19^46^; the COVID-19 Genomics UK (COG-UK) covid-19 viral sequencing study^47^; and the UK government chief scientific adviser’s national core studies programme, established to coordinate the UK’s covid-19 research response (in particular its underpinning data and connectivity theme led by Health Data Research UK).^20^

Nevertheless, limitations do exist: it is not yet possible to bring external cohort studies or trials into the environment for linkage (although data can be linked to these through NHS Digital’s standard data dissemination route); the primary care data are currently restricted to a large subset of SNOMED codes and limited to people known to be alive from November 2019 onwards, although NHS Digital is currently enacting its plans to obtain a fully comprehensive primary care dataset, to be updated daily, which will become available during 2021 and will eventually replace the current primary care dataset; the trusted research environment currently has a relatively limited range of services and analytical tools, although NHS Digital is committed to expanding these; the descriptive results presented here provide an overview of the available resources with illustrative examples but are not designed to inform reliable conclusions about the associations between patient characteristics and covid-19 outcomes, as the analyses are unadjusted and so prone to confounding; information on the accuracy of the Master Patient Service matching at the level of each record within each dataset is needed to provide assurance of high linkage quality and to allow assessment of whether this varies by important patient characteristics, such as age, ethnicity, and deprivation; several previous studies have shown that nationally collated, coded health data from primary and secondary healthcare settings in the UK are sufficiently accurate for many research purposes, using a range of different validation approaches,^48^ ^49^ but detailed, methodologically robust validation studies comparing contemporary linked UK routine data sources with expert adjudication based on the complete medical records for particular conditions are relatively rare, because they are time consuming, resource intensive, and challenging to perform^50^ ^51^; while data quality checks have been performed on each dataset before creating the linked data resource, these vary by dataset (see supplementary figure 1, notes on data processing and quality checks) and future analyses might require additional checks to detect duplicates and minimise influential errors, outliers, and inconsistent records.

### Sustainability for the future

NHS Digital has already invested in the infrastructure of its Data Access Environment that supports the new trusted research environment service. Because this service is new, ongoing costs remain uncertain but will operate on the basis of cost recovery and will vary depending on numbers of datasets being accessed, number of researchers, software licensing costs, and compute usage. The costs of establishing and maintaining the trusted research environment and associated services for the CVD-COVID-UK consortium and research aligned to the UK government’s chief scientific officer’s national core studies programme are being met by the BHF Data Science Centre and funding from the national core studies programme. We anticipate that data access costs for future research will be met through a range of research funding routes (potentially including the National Institutes of Health Research, UK research councils, charities, industry, and others) given the considerable value and research potential of these data.

### Extending coverage across the four nations of the UK

The BHF Data Science Centre has also enabled access for members of the CVD-COVID-UK consortium to similar, albeit not identical, linked health data in separate, national trusted research environments in Scotland (Scottish National Data Safe Haven) and Wales (SAIL Databank), with plans to extend further to Northern Ireland.^10^ ^22^ ^23^ Thus we have already extended coverage to enable studies across a population of more than 65 million people, including the majority of people in the UK and increasing possibilities for geographical comparisons across the UK. Owing to differences in data structure and coding procedures between nations, we advocate the development of analysis plans that aim for maximum consistency but allow for these nation specific differences. When appropriate, results of nation specific analyses can be combined to produce UK-wide results. Such combined analyses will, increasingly, be able to take advantage of Health Data Research UK’s plans to provide the infrastructure, methods, and tools to enable federation of analyses across trusted research environments.^52^

### Conclusion

We describe provision for research of linked nationwide EHR data for England and show the importance of linking person level data from different health settings for defining exemplar CVD outcomes, covid-19 diagnoses, and key characteristics. By covering almost the entire population of England, the resource includes all age groups, ethnicgroups, geographical locations, and socioeconomic, health, and personal characteristics, and it can enable statistically powerful population scale research with large numbers of outcomes. The resource is accessible to approved researchers through a secure trusted research environment hosted by NHS Digital to support research on covid-19 and CVD, with plans to expand to benefit a broad range of research.

**What is already known on this topic**

At the start of the covid-19 pandemic, approved researchers were unable to access national, linked health data across the whole UK population to conduct analyses that would support healthcare and public health policy

**What this study adds**

In partnership with NHS Digital, the British Heart Foundation Data Science Centre has developed a new trusted research environment for England, providing researchers with secure access to linked health data from primary and secondary care, registered deaths, covid-19 laboratory and vaccination data, and cardiovascular specialist audits
These datasets cover almost the entire population of England (>54 million people); similar linked data have been made available in trusted research environments for Scotland and Wales (>8 million people)
Large numbers of approved researchers are now accessing health data on almost all people in the UK to address important covid-19 related research questions

## Data Availability

The authors and colleagues across the CVD-COVID-UK consortium have invested considerable time and energy in developing the data resource described here and are keen to ensure that it is used widely to maximise its value. For inquiries about data access, please see www.healthdatagateway.org/dataset/7e5f0247-f033-4f98-aed3-3d7422b9dc6d or email bhfdsc@hdruk.ac.uk.

## Web extra

Extra material supplied by authors

Supplementary material: Additional figures, tables, and information

Supplementary information: Annexes 1 and 2

## Notes

In addition, the following people are acknowledged:

Consortium coordination (BHF Data Science Centre core team): Rouven Priedon, Cathie Sudlow, Lynn Morrice, and Debbie Ringham.

Consortium membership: www.hdruk.ac.uk/wp-content/uploads/2021/01/210128-CVD-COVID-UK-Consortium-Members.pdf (also see supplementary annexe 1)—consortium members who contributed to discussions leading up to this manuscript and provided helpful insights and comments.

Public and patient advisory panel: Suzannah Power, Lynn Laidlaw, Michael Molete, and John Walsh.

NHS Digital (coordination, data management or provision, data access request, and information governance support, trusted research environment support: Garry Coleman, Cath Day, Elizabeth Gaffney, Tim Gentry, Lisa Gray, Sam Hollings, Richard Irvine, Brian Roberts, Estelle Spence, and Janet Waterhouse.

The results described are based on data from patients, collected by the NHS as part of its care and support. We also thank the data providers who make anonymised data available for research.

The three lead authors, AW, JS, and CS (the manuscript’s guarantors), affirm that the manuscript is an honest, accurate, and transparent account of the resource and analyses being described and that no important aspects have been omitted.

Dissemination to participants and related patient and public communities: Results will be disseminated through the British Heart Foundation (BHF) Data Science Centre and CVD-COVID-UK webpages on the Health Data Research UK website, BHF communication channels, the BHF Data Science Centre’s lay members panel, and NHS Digital communications channels.

Provenance and peer review: Not commissioned; externally peer reviewed.
