# Supplementary material for: Linked electronic health records for research on a nationwide cohort of more than 54 million people in England: data resource
Source: BMJ. 2021 Apr 7;373:n826. doi: 10.1136/bmj.n826 (PMC8413899; doi:10.1136/bmj.n826)
Supplement: Supplementary file 1 — Supplementary material: Additional figures, tables, and information [file wooa065067.ww1.pdf]

Linked electronic health records for research on a nationwide cohort including over 54 million people in England

Supplementary Material

Supplementary Figure 1. Data sources and flows into NHS Digital

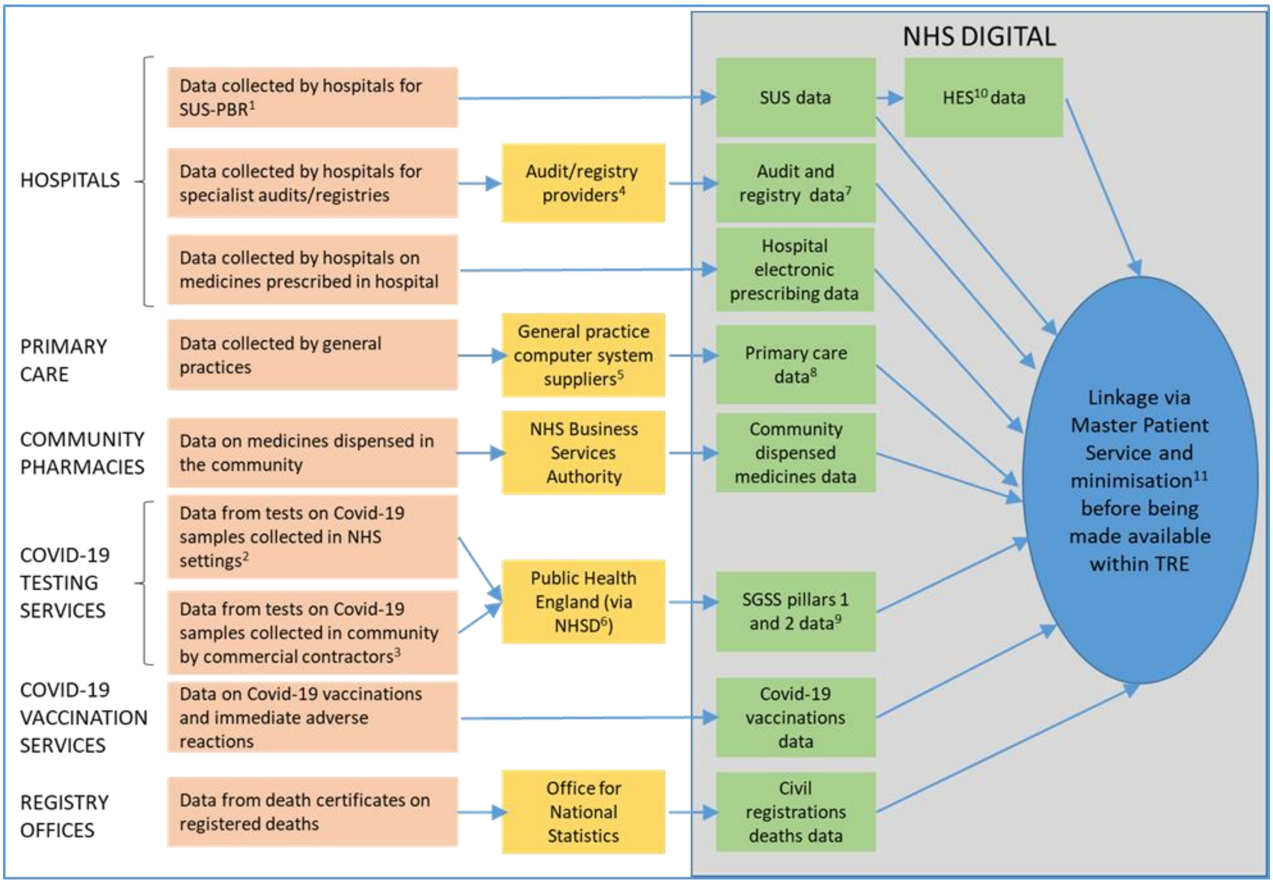

1. Secondary Use Services Payment by Results dataset covering hospital inpatient, critical care, outpatient and emergency department activity (https://digital.nhs.uk/services/secondary-uses-service-sus).
2. These samples are processed and analysed in NHS laboratories
3. These samples are processed and analysed in Lighthouse and partner laboratories (https://www.gov.uk/government/publications/nhs-test-and-trace-how-we-test-your-samples/nhs-test-and-trace-how-we-test-your-samples)
4. Data are collected and processed by the National Institute for Cardiac Outcomes Research https://www.nicor.org.uk/ (Myocardial Infarction National Audit Programme, Adult Percutaneous Coronary Interventions, National Heart Failure Audit, Cardiac Rhythm Management Audit, Congenital Heart Disease in Children and Adults, Adult Cardiac Surgery Audit, NICOR Health Technology Registries), Sentinel Stroke National Audit Programme https://www.strokeaudit.org/ (stroke audit data), Vascular Services Quality Improvement Programme https://www.vsqip.org.uk/ (national vascular registries) and Intensive Care National Audit and Research Programme https://www.icnarc.org/ (ICNARC critical care data). The majority of the audit data collections within NICOR, and those within SSNAP and VSQIP are commissioned by the Health Quality Improvement Programme https://www.hqip.org.uk/ on behalf of NHS England.
5. There are currently four general practice computer system suppliers operating in England. The two largest, covering the vast majority of practices, are TPP and EMIS, and the others are InPractice Systems and Microtest.
6. NHSD: NHS Digital
7. The audit and registry datasets are as listed under footnote 4.
8. The primary care dataset currently flowing from each general practice computer system supplier to NHS Digital is the GP Data for Pandemic Planning and Research (GDPPR). NHS Digital is currently establishing a new, comprehensive data extract, the GP Data for Planning and Research (GPDPR), which will be updated daily and should be fully operational during 2021.
9. SGSS: Public Health England's Second Generation Surveillance System, the national laboratory reporting system used in England to capture routine laboratory data on mainly infectious diseases and antimicrobial resistance. Pillar 1 data comprises test results from NHS settings and pillar 2 data comprises test results from community settings.
10. HES: hospital episode statistics, comprising inpatient, critical care, outpatient and emergency care data. HES data are derived from SUS data returns, following processing within NHS Digital.
11. Minimisation involves the removal of data items not included in the BHF Data Science Centre's data access request, removal of all direct identifiers and replacement of NHS numbers with person-specific pseudo-identifiers.

Information on data processing and quality checks to accompany Supplementary Figure 1

SUS and HES hospital data. Various initial checks are applied to data submitted to SUS, including checks for data items not exceeding the prescribed number of characters, date fields containing only date values, and mandatory items being populated. If a submission fails one or more of these checks then the whole set of data is rejected. After the submitted data has passed the initial validation checks, additional checks are performed to ensure that, for example, dates fall within valid ranges. Providers receive feedback about data items which fail these checks so that they can be rectified to allow activity to be tariffed. Further checks of SUS PbR data completeness, coverage and validity are performed by NHS Digital, both overall and by provider. Key performance indicator reports and data quality dashboards are published at provider level, allowing organisations to assess their own data in SUS to ensure that it is comprehensive and compliant with data standards.

Each month, at pre-arranged dates during the year, a SUS data extract is sent to HES. NHS Digital then validates and cleans the extract, before deriving new items and making the information available in the data warehouse. The data cleaning processes include mapping of providers (e.g. to account for provider mergers), removal of duplicate records, application of automatic data cleaning rules (e.g., when sex is submitted as M or F change to 1 or 2, or when sex is invalid change to 0, set dates of birth before 1/1/1885 as invalid), and a range of other checks of overall and provider-specific data volumes, and population and coverage of submitted data fields. (https://digital.nhs.uk/data-and-information/data-tools-and-services/data-services/hospital-episode-statistics/the-processing-cycle-and-hes-data-quality).

National audit datasets undergo various quality checks by the audit/registry provider organisations prior to their analysis for audit or onward provision for other purposes, including to NHS Digital. The provision of these audit data to NHS Digital is new in 2020/2021. To enable rapid use by researchers, only very limited quality checks within NHS Digital are expected prior to linkage, minimisation and provision into the TRE.

- For the NICOR audit datasets, NICOR has provided (as of mid-2020) each hospital with a data completeness tool to help them improve data completeness and accuracy (https://www.nicor.org.uk/for-hospital-clinical-and-audit-teams/online-reporting-tools/). The tool gives an overview of the hospital's data completeness for each audit and allows them to examine data for individual patients to determine where data are missing. In addition a new national quality improvement metric tool allows each hospital to look at how it currently compares with others, and to check their data and make appropriate valid corrections to improve data accuracy.

- For the SSNAP national stroke audit, data quality is enhanced through the provision of detailed help notes to ensure standard interpretation of the dataset questions, as well as FAQ's, user guides and videos (https://www.strokeaudit.org/Resources/New-SSNAP-Users.aspx). Completeness and timeliness of recording of the various data items required is assessed and fed back to each hospital quarterly (https://www.strokeaudit.org/results/Clinical-audit/National-Results.aspx).

- For the VSQIP national vascular registries, validation rules are built into the IT system, which reduce the chances of errors being made at the time of data entry. Analysts within the NVR team also carry out various data cleaning and duplicate checks as part of the analysis process (https://www.vsqip.org.uk/).

- For ICNARC critical care data, each individual event is checked for missing, unusual and invalid data, data for each site are checked for duplication and unusual patterns, each individual patient

is checked for readmission to critical care, ensuring data across these events are logical and consistent, and reliability testing is carried out to ensure that sites are collecting data consistently (https://www.icnarc.org/Our-Audit/About/Audit-Data-Management).

Hospital electronic prescribing and administration data. Since December 2020, NHS Digital has established a daily collection of data from hospital electronic prescription and administration systems supplied by WellSky, covering around 9% of secondary care prescribing activity, with an additional 6% to be available later in 2021 from Cerner EPMA systems. Trusts do not currently use their EPMA systems to record prescribing for all specialities, with coverage varying between trusts. NHS Digital has provided guidance for users, including information and advice on handling duplicate records (https://digital.nhs.uk/data-and-information/data-collections-and-data-sets/data-collections/electronic-prescribing-and-administration-epma-data-in-secondary-care).

Primary care data. To help users and potential users understand the coverage and quality of the GDPPR dataset, the GPES team has produced aggregate counts, proportions and distributions of items within the dataset along with data quality and interpretation notes. As the GDPPR asset was developed rapidly in response to the coronavirus outbreak, limited quality assurance checks are applied during data processing. The GPES team has therefore produced information to highlight data quality issues (e.g., implausible dates) identified by current users of the dataset to inform people of the limitations of the dataset, prevent duplication of initial quality checks by users and aid users in their understanding of the data (https://digital.nhs.uk/coronavirus/gpes-data-for-pandemic-planning-and-research/guide-for-analysts-and-users-of-the-data).

Community dispensed medicines data. Since July 2020, NHS Digital has established a collection of data from prescriptions submitted to the NHS Business Services Authority for reimbursement each month. The data comprise prescriptions for medicines dispensed or supplied by community pharmacies, appliance contractors and dispensing doctors in England. Prescriptions issued but not dispensed or not claimed are not included. Quality assurance checks on these data are carried out both by NHS Prescriptions Services and by the NHS BSA Data Warehouse team. Around 1.1 billion prescription items are processed annually. A statistically random sample of these is reprocessed each month to estimate accuracy. Results for January to December 2019 had an average value of 99.75% (https://www.nhsbsa.nhs.uk/sites/default/files/202007/QAAD_Assessment_Prescription_Data_v001_0.pdf).

SGSS Covid-19 testing data. These data are processed and de-duplicated within Public Health England before being provided to NHS Digital. They include the first positive results for people testing positive from pillar 1 and pillar 2 tests. (https://digital.nhs.uk/services/data-access-request-service-dars/dars-products-and-services).

Covid-19 vaccinations data. These include data from the National Immunisation Management Service (NIMS) on details of all vaccinations administered in England in hospital hubs, local vaccination service sites (e.g. GP practices) and vaccination centres. They include records relating to patients with adverse reactions within the first 15 minutes of vaccine administration. There is no information currently on data quality checks within NHS Digital for this new dataset (https://digital.nhs.uk/services/data-access-request-service-dars/dars-products-and-services).

Civil registrations deaths data. Data on registered deaths are received by NHS Digital daily from the Office for national Statistics (ONS). Details of deaths are received by ONS from register offices electronically. Routine and automated checks are carried out on each file (e.g., identification of missing entries, checks for duplicate records) and the combined data are then loaded onto the deaths database. Weekly contacts are made with registrars to resolve any problems. Once on the

database, the data are passed through a series of automated validation processes (e.g., checks for consistency between dates of birth, death and registration) with any inconsistencies highlighted. Coding, including of cause of death, is followed by further validity and consistency checks. Nonetheless, ONS data provided to NHS Digital may not contain all deaths which have occurred or been registered (e.g. due to registration delays for a small proportion of deaths) and so the data are subject to later small changes (https://www.ons.gov.uk/peoplepopulationandcommunity/birthsdeathsandmarriages/deaths/methodologies/userguidetomortalitystatisticsjuly2017#introduction).

Supplementary Table 1: Codelists for Myocardial Infarction (see https://github.com/BHFDSC/Linked-EHR-England-2021/tree/main/Phenotypes)

Code type	Code	Descriptions
ICD10 codes	I252	Old myocardial infarction (History of MI)
	I21	Acute myocardial infarction (Acute MI not further specified)
	I22	Subsequent myocardial infarction
	I23	Certain current complications following acute myocardial infarction (Complications of MI)
	I241	Dressler's syndrome (complications of MI)
SNOMED CONCEPT IDS	1755008	Old myocardial infarction
	10273003	Acute infarction of papillary muscle
	15990001	Acute myocardial infarction of posterolateral wall
	22298006	Myocardial infarction
	42531007	Microinfarct of heart
	52035003	Acute anteroapical myocardial infarction
	54329005	Acute myocardial infarction of anterior wall
	57054005	Acute myocardial infarction
	58612006	Acute myocardial infarction of lateral wall
	62695002	Acute anteroseptal myocardial infarction
	65547006	Acute myocardial infarction of inferolateral wall
	66189004	Postmyocardial infarction syndrome
	70211005	Acute myocardial infarction of anterolateral wall
	70422006	Acute subendocardial infarction
	71023004	Pericarditis secondary to acute myocardial infarction
	73795002	Acute myocardial infarction of inferior wall
	76593002	Acute myocardial infarction of inferoposterior wall
	79009004	Acute myocardial infarction of septum
	91335003	Mural thrombus of heart
	113155009	Myocardial infarction education
	129574000	Postoperative myocardial infarction
	161502000	H/O: myocardial infarct at less than 60
	161503005	H/O: myocardial infarct at greater than 60
	164865005	ECG: myocardial infarction
	194798004	Acute anteroapical infarction
	194802003	True posterior myocardial infarction
	194809007	Acute atrial infarction
	194856005	Subsequent myocardial infarction
	194857001	Subsequent myocardial infarction of anterior wall
	194858006	Subsequent myocardial infarction of inferior wall
	194861007	Certain current complications following acute myocardial infarction
	194862000	Haemopericardium as current complication following acute myocardial infarction
	194863005	Atrial septal defect as current complication following acute myocardial infarction
	194865003	Rupture of cardiac wall without hemopericardium as current complication following acute myocardial infarction
	194866002	Rupture of chordae tendinae as current complication following acute myocardial infarction
	194867006	Rupture of papillary muscle as current complication following acute myocardial infarction
	194868001	Thrombosis of atrium, auricular appendage, and ventricle as current complications following acute myocardial infarction
	233838001	Acute posterior myocardial infarction
	233843008	Silent myocardial infarction
	233846000	Ventricular septal defect as current complication following acute myocardial infarction
	233847009	Cardiac rupture after acute myocardial infarction
	233860003	Post-infarction mitral papillary muscle rupture
	233885007	Post-infarction pericarditis
	233889001	Post-infarction hemopericardium
	233929004	Post-infarction mural thrombus
	266897007	Family history of myocardial infarction
	267390005	Post-infarction hypopituitarism
	304914007	Acute Q wave myocardial infarction
	307140009	Acute non-Q wave infarction
	308065005	H/O: Myocardial infarction in last year
	311792005	Postoperative transmural myocardial infarction of anterior wall
	311793000	Postoperative transmural myocardial infarction of inferior wall
	311796008	Postoperative subendocardial myocardial infarction
	314116003	Post infarct angina
	315287002	Diabetes mellitus insulin-glucose infusion in acute myocardial infarction

	371068009	Myocardial infarction with complication (disorder)
	398274000	Coronary artery thrombosis (disorder)
	399211009	History of - myocardial infarction (context-dependent category)
	401303003	Acute ST segment elevation myocardial infarction (disorder)
	401314000	Acute non-ST segment elevation myocardial infarction (disorder)
	428752002	Recent myocardial infarction (situation)
	461000119108	History of MI less than 8 weeks
	698593009	History of non-ST segment elevation myocardial infarction (situation)
	703330009	Mitral valve regurgitation due to acute myocardial infarction with papillary muscle and chordal rupture
	703328007	Mitral valve regurgitation due to acute myocardial infarction without papillary muscle and chordal rupture
	703326006	Mitral regurgitation due to acute myocardial infarction
	103011000119106	Coronary arteriosclerosis in patient with history of previous myocardial infarction (situation)
	285721000119104	History of acute ST segment elevation myocardial infarction (situation)
	15960981000119105	Mural thrombus of left ventricle following acute myocardial infarction (disorder)
	723858002	Ventricular aneurysm as current complication following acute myocardial infarction (disorder)
	723859005	Pulmonary embolism as current complication following acute myocardial infarction (disorder)
	723860000	Arrhythmia as current complication following acute myocardial infarction (disorder)
	723861001	Cardiogenic shock unrelated to mechanical complications as current complication following acute myocardial infarction (disorder)
	736978009	Mural thrombus of right ventricle following acute myocardial infarction (disorder)
	1077002	Septal infarction by electrocardiogram (finding)
	1089431000000107	Postoperative nontransmural myocardial infarction (disorder)
	1089451000000100	Acute nontransmural myocardial infarction (disorder)
	15712881000119105	Acute ST segment elevation myocardial infarction of anterolateral wall (disorder)
	15712921000119103	Acute ST segment elevation myocardial infarction of lateral wall (disorder)
	15712961000119108	Acute ST segment elevation myocardial infarction of anteroseptal wall (disorder)
	15713121000119105	Acute ST segment elevation myocardial infarction due to right coronary artery occlusion (disorder)
	15713161000119100	Acute ST segment elevation myocardial infarction of septum (disorder)
	17531000119105	Acute myocardial infarction due to left coronary artery occlusion (disorder)
	233825009	Acute Q wave infarction - anteroseptal (disorder)
	233826005	Acute non-Q wave infarction - anteroseptal (disorder)
	233832000	Acute non-Q wave infarction - inferolateral (disorder)
	285981000119103	Acute ST segment elevation myocardial infarction involving left anterior descending coronary artery (disorder)
	285991000119100	Acute ST segment elevation myocardial infarction involving left main coronary artery (disorder)
	394659003	Acute coronary syndrome (disorder)
	394710008	First myocardial infarction (disorder)
	413444003	Acute myocardial ischemia (disorder)
	428196007	Mixed myocardial ischemia and infarction (disorder)
	429391004	New myocardial infarction compared to prior study (finding)
	59063002	Acute myocardial infarction of apical-lateral wall (disorder)
	63670007	Myocardial imaging for infarct, planar technique (procedure)
	703165004	Acute ST segment elevation myocardial infarction of anterior wall involving right ventricle (disorder)
	703210007	Subsequent ST segment elevation myocardial infarction of anterior wall (disorder)
	703213009	Acute ST segment elevation myocardial infarction of inferior wall (disorder)
	703251009	Acute myocardial infarction of inferior wall involving right ventricle (disorder)
	703252002	Acute myocardial infarction of anterior wall involving right ventricle (disorder)
	1089441000000103	Postoperative transmural myocardial infarction (disorder)
	1089471000000109	Acute transmural myocardial infarction (disorder)
	12238111000119106	Acute ST segment elevation myocardial infarction of inferolateral wall (disorder)
	12238151000119107	Acute ST segment elevation myocardial infarction of inferoposterior wall (disorder)
	15712841000119100	Acute ST segment elevation myocardial infarction of posterolateral wall (disorder)
	15713041000119103	Acute ST segment elevation myocardial infarction of posterior wall (disorder)
	15713081000119108	Acute ST segment elevation myocardial infarction due to left coronary artery occlusion (disorder)
	15713201000119105	Acute ST segment elevation myocardial infarction of posterobasal wall (disorder)
	23311000119105	Acute myocardial infarction due to right coronary artery occlusion (disorder)
	233827001	Acute Q wave infarction - anterolateral (disorder)
	233828006	Acute non-Q wave infarction - anterolateral (disorder)
	233829003	Acute Q wave infarction - inferior (disorder)

	233830008	Acute non-Q wave infarction - inferior (disorder)
	233831007	Acute Q wave infarction - inferolateral (disorder)
	233833005	Acute Q wave infarction - lateral (disorder)
	233834004	Acute non-Q wave infarction - lateral (disorder)
	233835003	Acute widespread myocardial infarction (disorder)
	233836002	Acute Q wave infarction - widespread (disorder)
	233837006	Acute non-Q wave infarction - widespread (disorder)
	282006	Acute myocardial infarction of basal-lateral wall (disorder)
	30277009	Acute myocardial infarction with rupture of ventricle (disorder)
	314207007	Non-Q wave myocardial infarction (disorder)
	41466009	Myocardial imaging for infarct with ejection fraction, first pass technique (procedure)
	418044006	Myocardial infarction in recovery phase (disorder)
	64627002	Acute myocardial infarction of high lateral wall (disorder)
	703164000	Acute ST segment elevation myocardial infarction of anterior wall (disorder)
	703209002	Subsequent ST segment elevation myocardial infarction of inferior wall (disorder)
	703211006	Subsequent ST segment elevation myocardial infarction (disorder)
	703212004	Acute myocardial infarction during procedure (disorder)
	703253007	Acute ST segment elevation myocardial infarction of inferior wall involving right ventricle (disorder)
	703360004	Subsequent non-ST segment elevation myocardial infarction (disorder)
	70998009	Acute myocardial infarction of posterobasal wall (disorder)

Supplementary Table 2a: Codelists for Ischaemic Stroke (see https://github.com/BHFDSC/Linked-EHR-England-2021/tree/main/Phenotypes)

Code type	Code	Descriptions
**ICD10 codes**	I63.0	Cerebral infarction due to thrombosis of precerebral arteries
	I63.1	Cerebral infarction due to embolism of precerebral arteries
	I63.2	Cerebral infarction due to unspecified occlusion or stenosis of precerebral arteries
	I63.3	Cerebral infarction due to thrombosis of cerebral arteries
	I63.4	Cerebral infarction due to embolism of cerebral arteries
	I63.5	Cerebral infarction due to unspecified occlusion or stenosis of cerebral arteries
	I63.8	Other cerebral infarction
	I63.9	Cerebral infarction, unspecified
	I69.3	Sequelae of cerebral infarction
SNOMED CONCEPT IDS	71444005	Cerebral thrombosis
	75543006	Cerebral embolism
	78569004	Posterior inferior cerebellar artery syndrome
	95457000	Brain stem infarction
	95460007	Cerebellar infarction
	195185009	Cerebral infarct due to thrombosis of precerebral arteries
	195186005	Cerebral infarction due to embolism of precerebral arteries
	195189003	Cerebral infarction due to thrombosis of cerebral arteries
	195190007	Cerebral infarction due to embolism of cerebral arteries
	195200006	Carotid artery syndrome hemispheric
	195201005	Multiple and bilateral precerebral artery syndromes
	195209007	Middle cerebral artery syndrome
	195210002	Anterior cerebral artery syndrome
	195211003	Posterior cerebral artery syndrome
	195243003	Sequelae of cerebral infarction
	230691006	CVA - cerebrovascular accident due to cerebral artery occlusion
	230692004	Infarction - precerebral
	230698000	Lacunar infarction
	230699008	Pure motor lacunar syndrome
	230700009	Pure sensory lacunar infarction
	266253001	Precerebral arterial occlusion
	307766002	Left sided cerebral infarction
	307767006	Right sided cerebral infarction
	373606000	Occlusive stroke (disorder)
	413102000	Infarction of basal ganglia (disorder)
	422504002	Ischemic stroke (disorder)
	432504007	Cerebral infarction (disorder)
	441526008	Infarct of cerebrum due to iatrogenic cerebrovascular accident
	723083001	Late effects of cerebral ischemic stroke (disorder)
	724424009	Cerebral ischemic stroke due to small artery occlusion (disorder)
	724425005	Cerebral ischemic stroke due to intracranial large artery atherosclerosis (disorder)
	724426006	Cerebral ischemic stroke due to extracranial large artery atherosclerosis (disorder)
	724787004	Epilepsy due to cerebrovascular accident (disorder)
	724993002	Cerebral ischemic stroke due to occlusion of extracranial large artery (disorder)
	724994008	Cerebral ischemic stroke due to stenosis of extracranial large artery (disorder)
	725132001	Ischemic stroke without residual deficits (disorder)
	23671000119107	Sequela of ischemic cerebral infarction (disorder)
	33301000119105	Sequela of cardioembolic stroke
	33331000119103	Sequela of lacunar stroke
	33331000119103	Sequela of lacunar stroke
	46421000119102	Behaviour disorder as sequela of cerebral infarction
	91601000119109	Sequela of thrombotic stroke (disorder)
	118951000119103	History of thrombotic stroke without residual deficits
	118961000119101	History of hemorrhagic cerebrovascular accident without residual deficits (situation)
	125081000119106	Cerebral infarction due to occlusion of precerebral artery
	140701000119108	History of hemorrhagic stroke with hemiparesis (situation)
	140711000119106	History of hemorrhagic stroke with hemiplegia
	140911000119109	Ischaemic stroke with coma
	140921000119102	Ischaemic stroke without coma
	141281000119101	History of ischemic stroke without residual deficits (situation)
	141811000119106	History of hemorrhagic cerebrovascular accident with residual deficit (situation)
	141821000119104	History of ischemic cerebrovascular accident with residual deficit (situation)
	141831000119101	History of embolic stroke with deficits
	145741000119101	Apraxia as late effect of cerebrovascular disease

	293831000119105	Cerebral infarction due to stenosis of precerebral artery
	672441000119103	Hemiplegia of nondominant side due to and following ischemic cerebrovascular accident (disorder)
	672461000119104	Hemiplegia of dominant side due to and following ischemic cerebrovascular accident (disorder)
	672501000119104	Dysarthria due to and following ischemic cerebrovascular accident (disorder)
	672521000119108	Dysphasia due to and following ischemic cerebrovascular accident (disorder)
	672541000119102	Aphasia due to and following ischaemic cerebrovascular accident
	672561000119103	Cognitive deficit due to and following ischemic cerebrovascular accident (disorder)
	674091000119108	Vertigo due to and following ischemic cerebrovascular accident (disorder)
	674111000119100	Ataxia due to and following ischemic cerebrovascular accident
	674121000119107	Ataxia due to and following hemorrhagic cerebrovascular accident (disorder)
	674161000119102	Monoplegia of upper limb due to and following ischemic cerebrovascular accident (disorder)
	674361000119104	Apraxia due to and following ischemic cerebrovascular accident (disorder)
	674381000119108	Weakness of facial muscle due to and following ischemic cerebrovascular accident (disorder)
	674391000119106	Speech and language deficit due to and following hemorrhagic cerebrovascular accident (disorder)
	674401000119108	Speech and language deficit due to and following ischemic cerebrovascular accident (disorder)
	16896891000119100	History of cerebrovascular accident due to ischemia (situation)
	1089421000000100	Cerebral infarction due to stenosis of cerebral artery (disorder)
	16000351000119100	Cerebrovascular accident due to occlusion of left posterior cerebral artery (disorder)
	16000431000119100	Cerebrovascular accident due to occlusion of right middle cerebral artery (disorder)
	16000511000119100	Cerebrovascular accident due to occlusion of left middle cerebral artery (disorder)
	16002031000119100	Cerebrovascular accident due to thrombus of right middle cerebral artery (disorder)
	16002111000119100	Cerebrovascular accident due to thrombus of left middle cerebral artery (disorder)
	16218291000119100	Acute cerebral ischemia (disorder)
	195230003	Cerebral infarction due to cerebral venous thrombosis, non-pyogenic (disorder)
	20059004	Occlusion of cerebral artery (disorder)
	230693009	Anterior cerebral circulation infarction (disorder)
	230696001	Posterior cerebral circulation infarction (disorder)
	230701008	Pure sensorimotor lacunar infarction (disorder)
	292851000119109	Lacunar ataxic hemiparesis of right dominant side (disorder)
	307363008	Multiple lacunar infarcts (disorder)
	329361000119107	Cerebrovascular accident due to occlusion of right middle cerebral artery by embolus (disorder)
	329421000119107	Cerebrovascular accident due to occlusion of right posterior cerebral artery by embolus (disorder)
	329431000119105	Cerebrovascular accident due to occlusion of left posterior cerebral artery by embolus (disorder)
	329461000119102	Cerebrovascular accident due to occlusion of left cerebellar artery by embolus (disorder)
	329561000119101	Occlusion of right posterior cerebral artery (disorder)
	329641000119104	Cerebrovascular accident due to thrombus of basilar artery (disorder)
	329651000119102	Cerebrovascular accident due to thrombus of right carotid artery (disorder)
	330411000119109	Lacunar ataxic hemiparesis of left nondominant side (disorder)
	330421000119102	Lacunar ataxic hemiparesis of right nondominant side (disorder)
	371040005	Thrombotic stroke (disorder)
	371041009	Embolic stroke (disorder)
	444657001	Superior cerebellar artery syndrome (disorder)
	724429004	Stroke co-occurrent with migraine (disorder)
	734383005	Thrombosis of left middle cerebral artery (disorder)
	734961002	Embolus of left posterior cerebral artery (disorder)
	734963004	Embolus of right posterior cerebral artery (disorder)
	734964005	Embolus of left middle cerebral artery (disorder)
	762629007	Occlusion of right middle cerebral artery by embolus (disorder)
	762630002	Occlusion of left middle cerebral artery by embolus (disorder)
	762651004	Occlusion of right posterior cerebral artery by embolus (disorder)
	87555007	Claude's syndrome (disorder)
	1089411000000100	Cerebral infarction due to occlusion of cerebral artery (disorder)
	14309005	Anterior choroidal artery syndrome (disorder)
	15978431000119100	Thrombosis of right vertebral artery (disorder)
	16000391000119100	Cerebrovascular accident due to occlusion of right posterior cerebral artery (disorder)
	195213000	Cerebellar stroke syndrome (disorder)
	230694003	Total anterior cerebral circulation infarction (disorder)
	230695002	Partial anterior cerebral circulation infarction (disorder)
	230702001	Lacunar ataxic hemiparesis (disorder)

	230703006	Dysarthria-clumsy hand syndrome (disorder)
	230704000	Multi-infarct state (disorder)
	24654003	Weber-Gubler syndrome (disorder)
	25133001	Completed stroke (disorder)
	276219001	Occipital cerebral infarction (disorder)
	276220007	Foville syndrome (disorder)
	276221006	Millard-Gubler syndrome (disorder)
	276222004	Top of basilar syndrome (disorder)
	292861000119106	Lacunar ataxic hemiparesis of left dominant side (disorder)
	329371000119101	Cerebrovascular accident due to occlusion of left middle cerebral artery by embolus (disorder)
	329481000119106	Occlusion of right middle cerebral artery (disorder)
	329491000119109	Occlusion of left middle cerebral artery (disorder)
	329571000119107	Occlusion of left posterior cerebral artery (disorder)
	330791000119108	Cerebrovascular accident due to thrombus of left carotid artery (disorder)
	413758000	Cardioembolic stroke (disorder)
	426107000	Acute lacunar infarction (disorder)
	426983002	Infarction of medulla oblongata (disorder)
	734384004	Thrombosis of right middle cerebral artery (disorder)
	734965006	Embolus of right middle cerebral artery (disorder)
	762652006	Occlusion of left posterior cerebral artery by embolus (disorder)
	90099008	Subcortical leukoencephalopathy (disorder)

Supplementary Table 2b: Codelists for Haemorrhagic Stroke (see https://github.com/BHFDSC/Linked-EHR-England-2021/tree/main/Phenotypes)

Code type	Code	Descriptions
**ICD10 codes**	I61	Intracerebral haemorrhage
	I69.1	Sequelae of intracerebral haemorrhage
SNOMED CONCEPT IDS	1508000	Intracerebral hemorrhage
	7713009	Intrapontine hemorrhage
	10458001	Evacuation of intracerebral hematoma
	28837001	Bulbar hemorrhage
	49422009	Cortical hemorrhage
	52201006	Internal capsule hemorrhage
	75038005	Cerebellar hemorrhage
	195165005	Basal ganglia hemorrhage
	195167002	External capsule hemorrhage
	195168007	Intracerebral haemorrhage, intraventricular
	195169004	Intracerebral hemorrhage, multiple localized
	195241001	Sequelae of intracerebral haemorrhage
	195242008	Sequelae of other non-traumatic intracranial hemorrhage
	230710000	Lobar cerebral hemorrhage
	274100004	Cerebral hemorrhage
	308128006	Right sided intracerebral hemorrhage, unspecified
	417506008	Hemorrhagic stroke monitoring (regime/therapy)
	428267002	History of cerebral hemorrhage (situation)
	732923001	Hemorrhage of medulla oblongata (disorder)
	118961000119101	History of hemorrhagic cerebrovascular accident without residual deficits (situation)
	140701000119108	History of hemorrhagic stroke with hemiparesis (situation)
	140711000119106	History of hemorrhagic stroke with hemiplegia
	141811000119106	History of hemorrhagic cerebrovascular accident with residual deficit (situation)
	145741000119101	Apraxia as late effect of cerebrovascular disease
	674121000119107	Ataxia due to and following hemorrhagic cerebrovascular accident (disorder)
	674391000119106	Speech and language deficit due to and following hemorrhagic cerebrovascular accident (disorder)
	230712008	Lacunar hemorrhage (disorder)
	276722003	Intracerebellar and posterior fossa hemorrhage (disorder)
	291531000119108	Spontaneous hemorrhage of cerebral hemisphere (disorder)
	291541000119104	Spontaneous hemorrhage of brain stem (disorder)
	95454007	Brain stem hemorrhage (disorder)
	1078001000000100	Haemorrhagic stroke (disorder)
	20908003	Subcortical cerebral hemorrhage (disorder)
	230709005	Massive supratentorial cerebral hemorrhage (disorder)
	230711001	Thalamic hemorrhage (disorder)
	291511000119103	Spontaneous hemorrhage of deep cerebral hemisphere (disorder)
	291521000119105	Spontaneous hemorrhage of cortical intracerebral hemisphere (disorder)

Supplementary Table 2c: Codelists for Unspecified Stroke (see https://github.com/BHFDSC/Linked-EHR-England-2021/tree/main/Phenotypes)

Code type	Code	Descriptions
**ICD10 codes**	G46.3	Brain stem stroke syndrome
	G46.4	Cerebellar stroke syndrome
	G46.5	Pure motor lacunar syndrome
	G46.6	Pure sensory lacunar syndrome
	G46.7	Other lacunar syndromes
	G46.8	Other vascular syndromes of brain in cerebrovascular diseases
	I64	Stroke, not specified as haemorrhage or infarction
	I69.4	Sequelae of stroke, not specified as haemorrhage or infarction
SNOMED CONCEPT IDS	6594005	Cerebrovascular disorder in the puerperium
	170600009	Stroke monitoring
	195212005	Brainstem stroke syndrome
	195213000	Cerebellar stroke syndrome
	195216008	Left sided cerebral hemisphere cerebrovascular accident
	195217004	Right sided CVA
	195239002	Late effects of cerebrovascular disease
	195608005	[X]Sequelae of stroke, not specified as haemorrhage or infarction
	230690007	CVA - Cerebrovascular accident
	275434003	Stroke in the puerperium
	275526006	H/O: CVA
	275527002	H/O: stroke
	277286006	CPSP - Central post-stroke pain
	308067002	H/O: Stroke in last year
	425642008	Monoplegia of dominant lower limb as a late effect of cerebrovascular accident (disorder)
	425882004	Paralytic syndrome as late effect of stroke (disorder)
	426033005	Dysphagia as a late effect of cerebrovascular accident (disorder)
	426788002	Vertigo as late effect of stroke (disorder)
	427065003	Monoplegia of dominant upper limb as a late effect of cerebrovascular accident (disorder)
	427432001	Paralytic syndrome as late effect of thalamic stroke (disorder)
	428668000	Apraxia due to cerebrovascular accident (disorder)
	429993008	History of cerebrovascular accident without residual deficits (situation)
	430947007	Paralytic syndrome of nondominant side as late effect of stroke (disorder)
	430959006	Paralytic syndrome of dominant side as late effect of stroke (disorder)
	431310008	History of occlusion of cerebral artery (situation)
	433183000	Neurogenic bladder as late effect of cerebrovascular accident (disorder)
	440140008	History of cerebrovascular accident with residual deficit (situation)
	441529001	Dysphasia as late effect of cerebrovascular disease
	441630004	Aphasia as late effect of cerebrovascular disease
	441735003	Sensory disorder as a late effect of cerebrovascular disease
	441759008	Abnormal vision as a late effect of cerebrovascular disease
	441887006	Monoplegia of lower limb as late effect of cerebrovascular disease
	441894009	Monoplegia of nondominant lower limb as a late effect of cerebrovascular accident
	441960006	Speech and language deficit as late effect of cerebrovascular accident
	441991000	Hemiparesis as late effect of cerebrovascular accident
	442024001	Hemiplegia as late effect of cerebrovascular disease
	442097001	Monoplegia of upper limb as late effect of cerebrovascular disease
	441894009	Monoplegia of nondominant lower limb as a late effect of cerebrovascular accident
	441960006	Speech and language deficit as late effect of cerebrovascular accident
	441991000	Hemiparesis as late effect of cerebrovascular accident
	442024001	Hemiplegia as late effect of cerebrovascular disease
	442097001	Monoplegia of upper limb as late effect of cerebrovascular disease
	442181008	Monoplegia of nondominant upper limb as a late effect of cerebrovascular accident
	442212003	Residual cognitive deficit as late effect of cerebrovascular accident
	442617003	Aphasia as late effect of cerebrovascular accident
	442668000	Hemiplegia of nondominant side as late effect of cerebrovascular disease
	442676003	Hemiplegia of dominant side as late effect of cerebrovascular disease
	442733008	Hemiplegia as late effect of cerebrovascular accident
	698767004	Post-cerebrovascular accident epilepsy
	699270006	Stroke annual review

	699429007	History of cerebrovascular accident in last eight weeks (situation)
	713410003	Pain following cerebrovascular accident
	720849008	Education about stroke
	722929005	Perinatal arterial ischemic stroke (disorder)
	40161000119102	Weakness of face muscles as sequela of stroke
	48601000119107	Hemiplegia and/or hemiparesis following stroke
	87551000119101	Visual disturbance as sequela of cerebrovascular disease (disorder)
	92341000119107	Weakness of extremities as sequela of stroke
	97531000119106	History of parietal cerebrovascular accident
	99051000119101	History of lacunar cerebrovascular accident
	102831000119104	Paraplegia or paraparesis as sequela of stroke
	103761000119107	Paralytic syndrome of all four limbs as sequela of stroke (disorder)
	133981000119106	Dysarthria as late effects of cerebrovascular disease
	133991000119109	Fluency disorder as sequela of cerebrovascular disease
	134771000119108	Alteration of sensation as late effect of stroke (disorder)
	137991000119103	Seizure disorder as sequela of stroke
	140281000119108	Hemiparesis as late effect of cerebrovascular disease
	148871000119109	Weakness as a late effect of stroke
	186831000119104	Apraxia due to and following cerebrovascular accident (disorder)
	290581000119101	Ataxia due to and following cerebrovascular accident (disorder)
	290621000119101	Cognitive deficit due to and following cerebrovascular disease (disorder)
	290631000119103	Dysarthria due to and following cerebrovascular accident (disorder)
	290791000119105	Fluency disorder due to and following cerebrovascular accident (disorder)
	290931000119108	Monoplegia of lower limb due to and following cerebrovascular accident (disorder)
	291091000119102	Monoplegia of left nondominant upper limb due to and following cerebrovascular accident (disorder)
	291111000119105	Monoplegia of right nondominant upper limb due to and following cerebrovascular accident (disorder)
	291121000119103	Monoplegia of upper limb due to and following cerebrovascular accident (disorder)
	292851000119109	Lacunar ataxic hemiparesis of right dominant side (disorder)
	292861000119106	Lacunar ataxic hemiparesis of left dominant side (disorder)
	330411000119109	Lacunar ataxic hemiparesis of left nondominant side (disorder)
	330421000119102	Lacunar ataxic hemiparesis of right nondominant side (disorder)
	690051000119100	History of stroke of cerebellum
	12242711000119100	Weakness of left facial muscle due to and following cerebrovascular accident (disorder)
	12242751000119100	Weakness of right facial muscle due to and following cerebrovascular accident (disorder)
	12367511000119100	Paraplegia due to and following cerebrovascular accident (disorder)
	15982271000119100	Weakness of right facial muscle due to and following cerebrovascular disease (disorder)
	15982311000119100	Weakness of left facial muscle due to and following cerebrovascular disease (disorder)
	16260551000119100	Dysphasia due to and following cerebrovascular accident (disorder)
	111297002	Nonparalytic stroke (disorder)
	116288000	Paralytic stroke (disorder)
	16371781000119100	Cerebellar stroke (disorder)
	195209007	Middle cerebral artery syndrome (disorder)
	195210002	Anterior cerebral artery syndrome (disorder)
	195211003	Posterior cerebral artery syndrome (disorder)
	195212005	Brainstem stroke syndrome (disorder)
	195216008	Left sided cerebral hemisphere cerebrovascular accident (disorder)
	195217004	Right sided cerebral hemisphere cerebrovascular accident (disorder)
	230706003	Hemorrhagic cerebral infarction (disorder)
	230708002	Posterior cerebral circulation hemorrhagic infarction (disorder)
	281240008	Extension of cerebrovascular accident (disorder)
	297138001	Embolus of circle of Willis (disorder)
	444172003	Recurrent transient cerebral ischemic attack (disorder)
	230690007	Cerebrovascular accident (disorder)
	230707007	Anterior cerebral circulation hemorrhagic infarction (disorder)
	230713003	Stroke of uncertain pathology (disorder)
	230714009	Anterior circulation stroke of uncertain pathology (disorder)
	230715005	Posterior circulation stroke of uncertain pathology (disorder)
	275434003	Stroke in the puerperium (disorder)
	426814001	Transient cerebral ischemia due to atrial fibrillation (disorder)
	57981008	Progressing stroke (disorder)

Supplementary Table 2d: Codelists for Transient Ischaemic Attack (see https://github.com/BHFDSC/Linked-EHR-England-2021/tree/main/Phenotypes)

Code type	Code	Descriptions
**ICD10 codes**	G45.0	Vertebro-basilar artery syndrome
	G45.1	Carotid artery syndrome (hemispheric)
	G45.2	Multiple and bilateral precerebral artery syndromes
	G45.3	Amaurosis fugax
	G45.4	Transient global amnesia
	G45.8	Other transient cerebral ischaemic attacks and related syndromes
	G45.9	Transient cerebral ischaemic attack, unspecified
	G46.0	Middle cerebral artery syndrome
	G46.1	Anterior cerebral artery syndrome
	G46.2	Posterior cerebral artery syndrome
	I65	Occlusion and stenosis of precerebral arteries, not resulting in cerebral infarction
	I66	Occlusion and stenosis of cerebral arteries, not resulting in cerebral infarction
SNOMED CONCEPT IDS	88032003	Amaurosis fugax
	195206000	Intermittent cerebral ischaemia
	230716006	Anterior circulation transient ischaemic attack
	266257000	TIA
	710575003	Transient ischemic attack due to embolism
	140221000119109	History of transient ischemic attack due to embolism (situation)
	13016361000119100	History of amaurosis fugax (situation)
	15258001	Subclavian steal syndrome (disorder)
	195199008	Vertebrobasilar artery syndrome (disorder)
	195205001	Impending cerebral ischemia (disorder)
	34781003	Vertebral artery syndrome (disorder)
	64009001	Basilar artery syndrome (disorder)
	751371000000107	Personal history of transient ischaemic attack (situation)
	230717002	Vertebrobasilar territory transient ischemic attack (disorder)

Supplementary Table 3: Codelists for Obesity (see https://github.com/BHFDSC/Linked-EHR-England-2021/tree/main/Phenotypes)

Code type	Code	Descriptions
**ICD10 codes**	E66	Obesity
SNOMED CONCEPT IDS	162864005	Body mass index 30+ - obesity (finding)
	408512008	Body mass index 40+ - severely obese (finding)
	914721000000105	Obese class I (body mass index 30.0 - 34.9) (finding)
	914731000000107	Obese class II (body mass index 35.0 - 39.9) (finding)
	914741000000103	Obese class III (body mass index equal to or greater than 40.0) (finding)
	921031000000102	Child body mass index 98.1st-99.6th centile (finding)
	443371000124107	Obese class I (finding)
	443381000124105	Obese class II (finding)
	921051000000109	Child body mass index greater than 99.6th centile (finding)

Supplementary Table 4: Codelists for Diabetes (see https://github.com/BHFDSC/Linked-EHR-England-2021/tree/main/Phenotypes)

**ICD10 codes**	E10	Insulin-dependent diabetes mellitus
	E11	Non-insulin-dependent diabetes mellitus
	E12	Malnutrition-related diabetes mellitus
	O242	Diabetes mellitus in pregnancy: Pre-existing malnutrition-related diabetes mellitus
	E13	Other specified diabetes mellitus
	E14	Unspecified diabetes mellitus
	G590	Diabetic mononeuropathy
	G632	Diabetic polyneuropathy
	H280	Diabetic cataract
	H360	Diabetic retinopathy
	M142	Diabetic arthropathy
	N083	Glomerular disorders in diabetes mellitus
	O240	Diabetes mellitus in pregnancy: Pre-existing diabetes mellitus, insulin-dependent
	O241	Diabetes mellitus in pregnancy: Pre-existing diabetes mellitus, non-insulin-dependent
	O243	Diabetes mellitus in pregnancy: Pre-existing diabetes mellitus, unspecified
SNOMED CONCEPT IDS	4855003	Diabetic retinopathy
	6143009	Diabetic patient education
	8801005	Secondary diabetes mellitus
	9859006	Insulin-resistant diabetes mellitus AND acanthosis nigricans
	11530004	Brittle diabetes
	19378003	Diabetic pseudotabes
	19429009	Chronic ulcer of skin
	21858001	Diabetes with renal manifestations
	24927004	Diabetes with ketoacidosis
	25093002	Diabetic oculopathy
	25412000	Diabetic retinal microaneurysm
	25907005	Diabetic gangrene
	26298008	Diabetic coma with ketoacidosis
	28453007	Maturity onset diabetes mellitus in young
	31411005	Background diabetic retinopathy
	33248009	Diabetes with non-ketotic non-hyperosmolar coma
	34140002	Diabetic gastroparesis
	35777006	Diabetic mononeuropathy multiplex
	38046004	Diffuse type diabetic glomerulosclerosis
	38205001	Diarrhea in diabetes
	39058009	Diabetic amyotrophy
	39127005	Symmetric diabetic proximal motor neuropathy
	39181008	Diabetic radiculopathy
	43959009	Diabetic cataract
	44054006	Diabetes mellitus type II
	46635009	Insulin dependent diabetes mellitus
	48951005	Bullosis diabeticorum
	49455004	Diabetic polyneuropathy
	50620007	Diabetic autonomic neuropathy
	51002006	Diabetes mellitus associated with pancreatic disease
	54181000	Diabetes-nephrosis syndrome
	55692006	Diabetes with hyperosmolar coma
	59276001	Proliferative diabetic retinopathy
	62260007	Pretibial pigmental patches in diabetes
	63510008	Nodular type diabetic glomerulosclerosis
	73211009	Diabetes mellitus
	74627003	Diabetic complication
	75524006	Malnutrition related diabetes mellitus
	79554005	Asymmetric diabetic proximal motor neuropathy
	81830002	Diabetic mononeuropathy simplex
	82980005	Anemia of diabetes
	110996009	Armanni-Ebstein kidney
	111552007	Diabetes mellitus without complication
	111556005	Diabetic ketoacidosis without coma
	111557001	Diabetes with coma
	111558006	Insulin coma
	126534007	Diabetic mixed sensory-motor polyneuropathy
	126535008	Diabetic motor polyneuropathy
	127011001	Diabetic sensory polyneuropathy
	127012008	Lipoatrophic diabetes
	127013003	Diabetic renal disease
	127014009	Diabetic peripheral angiopathy
	134395001	Diabetic retinopathy screening
	161649006	H/O: insulin therapy
	170745003	Diabetic on diet only
	170746002	Diabetic on oral treatment

	170747006	Diabetic on insulin
	170763003	Diabetic - good control
	170766006	Loss of hypoglycemic warning
	170769004	Diabetic - cooperative patient
	170770003	Diabetic-uncooperative patient
	171183004	Diabetes mellitus screening
	183056000	Patient advised about diabetic diet
	190325001	Diabetes mellitus, juvenile type, with ketoacidosis
	190326000	Diabetes mellitus, adult onset, with ketoacidosis
	190327009	Other specified diabetes mellitus with ketoacidosis
	190328004	Diabetes mellitus NOS with ketoacidosis
	190329007	Diabetes mellitus with hyperosmolar coma
	190330002	Diabetes mellitus, juvenile type, with hyperosmolar coma
	190331003	Diabetes mellitus, adult onset, with hyperosmolar coma
	190332005	Diabetes mellitus NOS with hyperosmolar coma
	190333000	Diabetes mellitus with ketoacidotic coma
	190334006	Diabetes mellitus, juvenile type, with ketoacidotic coma
	190335007	Diabetes mellitus, adult onset, with ketoacidotic coma
	190337004	Diabetes mellitus NOS with ketoacidotic coma
	190339001	Diabetes mellitus, juvenile type, with renal manifestation
	190340004	Diabetes mellitus, adult onset, with renal manifestation
	190341000	Other specified diabetes mellitus with renal complications
	190342007	Diabetes mellitis with nephropathy NOS
	190343002	Diabetes mellitus with ophthalmic manifestation
	190345009	Diabetes mellitus, juvenile type, with ophthalmic manifestation
	190346005	Diabetes mellitus, adult onset, with ophthalmic manifestation
	190347001	Other specified diabetes mellitus with ophthalmic complications
	190348006	Diabetes mellitus NOS with ophthalmic manifestation
	190350003	Diabetes mellitus, juvenile type, with neurological manifestation
	190351004	Diabetes mellitus, adult onset, with neurological manifestation
	190352006	Other specified diabetes mellitus with neurological complications
	190353001	Diabetes mellitus NOS with neurological manifestation
	190354007	Diabetes mellitus with: [gangrene] or [peripheral circulatory disorder]
	190355008	Diabetes mellitus, juvenile type, with peripheral circulatory disorder
	190356009	Diabetes mellitus, adult onset, with peripheral circulatory disorder
	190357000	Diabetes mellitus, adult with gangrene
	190358005	IDDM with peripheral circulatory disorder
	190359002	NIDDM with peripheral circulatory disorder
	190360007	Other specified diabetes mellitus with peripheral circulatory complications
	190361006	Diabetes mellitus NOS with peripheral circulatory disorder
	190363009	Type I diabetes mellitus with renal complications
	190364003	Type 1 diabetes mellitus with ophthalmic complications
	190365002	Type 1 diabetes mellitus with neurological complications
	190366001	Type I diabetes mellitus with multiple complications
	190368000	Type I diabetes mellitus with ulcer
	190369008	Type 1 diabetes mellitus with gangrene
	190370009	Type I diabetes mellitus with retinopathy
	190372001	Type I diabetes mellitus maturity onset
	190385003	Type 2 diabetes mellitus with renal complications
	190386002	Type II diabetes mellitus with ophthalmic complications
	190387006	Type 2 diabetes mellitus with neurological complications
	190388001	Type II diabetes mellitus with multiple complications
	190389009	Type 2 diabetes mellitus with ulcer
	190390000	Type II diabetes mellitus with gangrene
	190391001	Type 2 diabetes mellitus with retinopathy
	190405001	Malnutrition-related diabetes mellitus with coma
	190406000	Malnutrition-related diabetes mellitus with ketoacidosis
	190408004	Malnutrition-related diabetes mellitus with ophthalmic complications
	190409007	Malnutrition-related diabetes mellitus with neurological complications
	190410002	Malnutrition-related diabetes mellitus with peripheral circulatory complications
	190411003	Malnutrition-related diabetes mellitus with multiple complications
	190422004	Diabetes mellitus with unspecified complication
	190423009	Diabetes mellitus, juvenile type, with unspecified complication
	190424003	Diabetes mellitus, adult onset, with unspecified complication
	190425002	Other specified diabetes mellitus with unspecified complications
	190426001	Diabetes mellitus NOS with unspecified complication
	190430003	Hypoglycemic coma NOS
	193141005	Diabetic mononeuritis multiplex
	193156001	Diabetic mononeuritis NOS
	193183000	Acute painful diabetic neuropathy
	193184006	Chronic painful diabetic neuropathy
	193185007	Asymptomatic diabetic neuropathy
	193349004	Preproliferative diabetic retinopathy
	193350004	Advanced diabetic maculopathy
	193353002	Diabetic retinopathy NOS
	193489006	Diabetic iritis
	197605007	Nephrotic syndrome in diabetes mellitus

	199229001	Pre-existing diabetes mellitus, insulin-dependent
	199230006	Pre-existing diabetes mellitus, non-insulin-dependent
	199231005	Pre-existing malnutrition-related diabetes mellitus
	200687002	Cellulitis in diabetic foot
	201250006	Ischaemic ulcer diabetic foot
	201251005	Neuropathic diabetic ulcer - foot
	201252003	Mixed diabetic ulcer - foot
	201723002	Diabetic hand syndrome
	201724008	Diabetic Charcot's arthropathy
	230572002	Diabetic neuropathy
	230573007	Diabetic distal sensorimotor polyneuropathy
	230574001	Diabetic acute painful polyneuropathy
	230575000	Diabetic chronic painful polyneuropathy
	230576004	Diabetic asymmetric polyneuropathy
	230577008	Diabetic mononeuropathy
	230578003	Diabetic truncal radiculopathy
	230579006	Diabetic thoracic radiculopathy
	232019003	Visually threatening diabetic retinopathy
	232020009	Diabetic maculopathy
	232021008	Proliferative diabetic retinopathy new vessels on disc
	232022001	Proliferative diabetic retinopathy with new vessels elsewhere than on disc
	232023006	Diabetic traction retinal detachment
	236499007	Microalbuminuric diabetic nephropathy
	236500003	Clinical diabetic nephropathy
	237599002	Insulin-treated non-insulin-dependent diabetes mellitus
	237604008	Diabetes mellitus autosomal dominant type II
	237620003	Abnormal metabolic state in diabetes mellitus
	237621004	Diabetic severe hyperglycemia
	237633009	Hypoglycemic state in diabetes
	237635002	Nocturnal hypoglycemia
	238981002	Soft tissue complication of diabetes mellitus
	238982009	Diabetic dermopathy
	238983004	Diabetic thick skin syndrome
	238984005	Diabetic rubeosis
	248542005	Diabetic relative
	267381003	Diabetes mellitus with renal manifestation
	267382005	Diabetes mellitus with neurological manifestation
	267383000	Diabetes mellitus with peripheral circulatory disorder
	267384006	Hypoglycaemic coma
	267474009	Diabetes with other complications
	267604001	Myasthenic syndrome due to diabetic amyotrophy
	268519009	Diabetic - poor control
	274589008	[EDTA] Diabetes Type I (insulin dependent) associated with renal failure
	274590004	[EDTA] Diabetes Type II (non-insulin-dependent) associated with renal failure
	275522008	Diabetes mellitus with gangrene
	280137006	Diabetic foot
	281023007	Diabetic child
	284350006	Dietary advice for diabetes mellitus
	286912007	Diabetes with ketoacidosis - no coma
	290002008	Unstable type I diabetes mellitus
	308105005	O/E - Right diabetic foot at risk
	308106006	O/E - Left diabetic foot at risk
	309426007	Diabetic glomerulopathy
	309595004	Retinal abnormality - diabetes-related
	309635005	H/O: Admission in last year for diabetes foot problem
	310387003	Diabetic intracapillary glomerulosclerosis
	310505005	Diabetic hyperosmolar non-ketotic state
	311366001	Kimmelstiel-Wilson syndrome
	311782002	Advanced diabetic retinal disease
	312903003	Mild non proliferative diabetic retinopathy
	312904009	Moderate non proliferative diabetic retinopathy
	312905005	Severe non proliferative diabetic retinopathy
	312906006	Proliferative diabetic retinopathy - non high risk
	312907002	Proliferative diabetic retinopathy - high risk
	312908007	Proliferative diabetic retinopathy - quiescent
	312909004	Proliferative diabetic retinopathy - iris neovascularisation
	312910009	Diabetic vitreous hemorrhage
	312912001	Diabetic macular edema
	313435000	Type I diabetes mellitus without complication
	313436004	Non-insulin-dependent diabetes mellitus without complication
	314010006	Diffuse diabetic maculopathy
	314011005	Focal diabetic maculopathy
	314014002	Ischaemic diabetic maculopathy
	314015001	Mixed diabetic maculopathy
	314194001	Diabetic on insulin and oral treatment
	314368001	Type I diabetes mellitus with mononeuropathy
	314369009	Type 1 diabetes mellitus with polyneuropathy

	314370005	Type II diabetes mellitus with mononeuropathy
	314371009	Type II diabetes mellitus with polyneuropathy
	314377008	Type I diabetes mellitus with nephropathy
	314378003	Type 2 diabetes mellitus with nephropathy
	314537004	Diabetic optic papillopathy
	314771006	Type 1 diabetes mellitus with hypoglycaemic coma
	314772004	Type 2 diabetes mellitus with hypoglycaemic coma
	314887002	Insulin dependent diabetes mellitus with diabetic cataract
	314888007	Type II diabetes mellitus with diabetic cataract
	314892000	Type I diabetes mellitus with peripheral angiopathy
	314893005	Type 1 diabetes mellitus with arthropathy
	314894004	Type I diabetes mellitus with neuropathic arthropathy
	314902007	Type II diabetes mellitus with peripheral angiopathy
	314903002	Non-insulin dependent diabetes mellitus with arthropathy
	314904008	Type II diabetes mellitus with neuropathic arthropathy
	359611005	Diabetic neuropathy with neurologic complication
	361216007	Diabetic femoral mononeuropathy
	371054002	Type I diabetes mellitus with complication (disorder)
	371055001	Type I diabetes mellitus with ketoacidosis (disorder)
	371056000	Type II diabetes mellitus with complication (disorder)
	371086007	Diabetes mellitus with skin ulcer (disorder)
	371087003	Diabetic foot ulcer (disorder)
	372069003	Diabetes mellitus with complication (disorder)
	390834004	Non proliferative diabetic retinopathy (disorder)
	390850007	O/E - no right diabetic retinopathy (context-dependent category)
	390853009	O/E - no left diabetic retinopathy (context-dependent category)
	390854003	O/E - diabetic maculopathy present both eyes (context-dependent category)
	390855002	O/E - diabetic maculopathy absent both eyes (context-dependent category)
	391178000	High risk non proliferative diabetic retinopathy (disorder)
	395204000	Hyperosmolar non-ketotic state in type 2 diabetes mellitus (disorder)
	398140007	Somogyi phenomenon (disorder)
	398819009	Diabetic foot at risk (context-dependent category)
	399862001	Proliferative diabetic retinopathy - high risk with no macular edema (disorder)
	399863006	Very severe nonproliferative diabetic retinopathy with no macular edema (disorder)
	399864000	Diabetic macular edema not clinically significant (disorder)
	399865004	Very severe proliferative diabetic retinopathy (disorder)
	399866003	Diabetic retinal venous beading (disorder)
	399868002	Diabetic intraretinal microvascular anomalies (disorder)
	399869005	High risk proliferative diabetic retinopathy not amenable to photocoagulation (disorder)
	399870006	Non-high-risk proliferative diabetic retinopathy with no macular edema (disorder)
	399871005	Visually threatening diabetic retinopathy (disorder)
	399872003	Severe nonproliferative diabetic retinopathy with clinically significant macular edema (disorder)
	399873008	Severe nonproliferative diabetic retinopathy with no macular edema (disorder)
	399874002	Proliferative diabetic retinopathy - high risk with clinically significant macular edema (disorder)
	399875001	Non-high-risk proliferative diabetic retinopathy with clinically significant macular edema (disorder)
	399876000	Very severe nonproliferative diabetic retinopathy (disorder)
	399877009	Very severe nonproliferative diabetic retinopathy with clinically significant macular edema (disorder)
	401087005	Diabetes mellitus with persistent microalbuminuria (disorder)
	401088000	Diabetes mellitus with persistent proteinuria (disorder)
	401109007	Type 1 diabetes mellitus with persistent proteinuria (disorder)
	401110002	Type 1 diabetes mellitus with persistent microalbuminuria (disorder)
	401111003	Type 2 diabetes mellitus with persistent proteinuria (disorder)
	401112005	Type 2 diabetes mellitus with persistent microalbuminuria (disorder)
	401191002	Diabetic foot examination (regime/therapy)
	403423006	Congenital total lipoatrophy (disorder)
	408287009	Type 1 diabetes mellitus with exudative maculopathy (disorder)
	408396006	Diabetic retinopathy screening not indicated (context-dependent category)
	408397002	Diabetic foot examination not indicated (context-dependent category)
	408409007	O/E - right eye background diabetic retinopathy (context-dependent category)
	408410002	O/E - left eye background diabetic retinopathy (context-dependent category)
	408411003	O/E - right eye preproliferative diabetic retinopathy (context-dependent category)
	408412005	O/E - left eye preproliferative diabetic retinopathy (context-dependent category)
	408413000	O/E - right eye proliferative diabetic retinopathy (context-dependent category)
	408414006	O/E - left eye proliferative diabetic retinopathy (context-dependent category)
	408415007	O/E - right eye diabetic maculopathy (context-dependent category)
	408416008	O/E - left eye diabetic maculopathy (context-dependent category)
	408417004	Type 2 diabetes mellitus with exudative maculopathy (disorder)
	408660003	Type II diabetes mellitus with ketoacidosis (disorder)
	412752009	Diabetic foot examination declined (context-dependent category)
	413122001	Diabetic retinopathy screening refused (context-dependent category)
	413180006	Pan retinal photocoagulation for diabetes (procedure)

	414894003	O/E - left eye stable treated proliferative diabetic retinopathy (context-dependent category)
	414910007	O/E - right eye stable treated proliferative diabetic retinopathy (context-dependent category)
	417677008	O/E - sight threatening diabetic retinopathy (context-dependent category)
	419100001	Infection of foot associated with diabetes (disorder)
	420270002	Ketoacidosis in type I diabetes mellitus (disorder)
	420279001	Renal disorder associated with type II diabetes mellitus (disorder)
	420414003	Multiple complications of type II diabetes mellitus (disorder)
	420422005	Ketoacidosis in diabetes mellitus (disorder)
	420436000	Mononeuropathy associated with type II diabetes mellitus (disorder)
	420486006	Exudative maculopathy associated with type I diabetes mellitus (disorder)
	420514000	Persistent proteinuria associated with type I diabetes mellitus (disorder)
	420662003	Coma associated with diabetes mellitus (disorder)
	420683009	Neurological disorder associated with malnutrition-related diabetes mellitus (disorder)
	420715001	Persistent microalbuminuria associated with type II diabetes mellitus (disorder)
	420756003	Diabetic cataract associated with type II diabetes mellitus (disorder)
	420789003	Diabetic retinopathy associated with type I diabetes mellitus (disorder)
	420825003	Gangrene associated with type I diabetes mellitus (disorder)
	420868002	Disorder associated with type I diabetes mellitus (disorder)
	420918009	Mononeuropathy associated with type I diabetes mellitus (disorder)
	420996007	Coma associated with malnutrition-related diabetes mellitus (disorder)
	421075007	Ketoacidotic coma in type I diabetes mellitus (disorder)
	421164006	Hypoglycemic coma in type II diabetes mellitus (disorder)
	421165007	Diabetic oculopathy associated with type I diabetes mellitus (disorder)
	421256007	Ophthalmic complication of malnutrition-related diabetes mellitus (disorder)
	421305000	Persistent microalbuminuria associated with type I diabetes mellitus (disorder)
	421326000	Neurologic disorder associated with type II diabetes mellitus (disorder)
	421365002	Peripheral circulatory disorder associated with type I diabetes mellitus (disorder)
	421437000	Hypoglycemic coma in type I diabetes mellitus (disorder)
	421468001	Neurological disorder associated with type I diabetes mellitus (disorder)
	421631007	Gangrene associated with type II diabetes mellitus (disorder)
	421707005	Polyneuropathy associated with type II diabetes mellitus (disorder)
	421725003	Hypoglycemic coma in diabetes mellitus (disorder)
	421750000	Ketoacidosis in type II diabetes mellitus (disorder)
	421779007	Exudative maculopathy associated with type II diabetes mellitus (disorder)
	421847006	Ketoacidotic coma in type II diabetes mellitus (disorder)
	421893009	Renal disorder associated with type I diabetes mellitus (disorder)
	421895002	Peripheral circulatory disorder associated with diabetes mellitus (disorder)
	421920002	Diabetic cataract associated with type I diabetes mellitus (disorder)
	421966007	Non-ketotic non-hyperosmolar coma associated with diabetes mellitus (disorder)
	421986006	Persistent proteinuria associated with type II diabetes mellitus (disorder)
	422014003	Disorder associated with type II diabetes melliltus (disorder)
	422034002	Diabetic retinopathy associated with type II diabetes mellitus (disorder)
	422088007	Neurologic disorder associated with diabetes mellitus (disorder)
	422099009	Diabetic oculopathy associated with type II diabetes mellitus (disorder)
	422126006	Hyperosmolar coma associated with diabetes mellitus (disorder)
	422166005	Peripheral circulatory disorder associated with type II diabetes mellitus (disorder)
	422183001	Skin ulcer associated with diabetes mellitus (disorder)
	422228004	Multiple complications of type I diabetes mellitus (disorder)
	422275004	Gangrene associated with diabetes mellitus (disorder)
	422297002	Polyneuropathy associated with type I diabetes mellitus (disorder)
	423263001	Diabetic autonomic neuropathy associated with type 2 diabetes mellitus (disorder)
	424736006	Diabetic peripheral neuropathy (disorder)
	424989000	Diabetic gastroparesis associated with type 2 diabetes mellitus (disorder)
	425159004	Diabetic gastroparesis associated with type 1 diabetes mellitus (disorder)
	425442003	Diabetic autonomic neuropathy associated with type 1 diabetes mellitus (disorder)
	425455002	Diabetic glomerulonephritis (disorder)
	426705001	Diabetes mellitus associated with cystic fibrosis (disorder)
	426875007	Latent autoimmune diabetes mellitus in adult (disorder)
	426907004	Small vessel disease due to type 1 diabetes mellitus (disorder)
	427027005	Amyotrophy due to type 2 diabetes mellitus (disorder)
	427134009	Small vessel disease due to type 2 diabetes mellitus (disorder)
	427571000	Amyotrophy due to type 1 diabetes mellitus (disorder)
	427943001	Diabetic ophthalmoplegia (disorder)
	428007007	Erectile dysfunction associated with type 2 diabetes mellitus (disorder)
	428274007	Dietary education for type II diabetes mellitus (procedure)
	428896009	Hyperosmolality due to uncontrolled type 1 diabetes mellitus (disorder)
	429094000	Dietary education for type I diabetes mellitus (procedure)
	429729007	Diabetic education completed (situation)
	433874008	History of diabetic peripheral angiopathy (situation)
	441628001	Multiple complications due to diabetes mellitus
	441656006	Hyperglycaemic crisis in diabetes mellitus
	443694000	Type II diabetes mellitus uncontrolled (finding)
	444073006	Type I diabetes mellitus uncontrolled (finding)
	445170001	Macroalbuminuric diabetic nephropathy (disorder)
	472969004	History of diabetes mellitus type 2 (situation)

	472970003	History of diabetes mellitus type 1 (situation)
	472972006	History of autosomal dominant diabetes mellitus
	473134007	Symptomatic diabetic peripheral neuropathy absent
	473180009	Discussion about diabetic ketoacidosis in pregnancy (procedure)
	609561005	Maturity-onset diabetes of the young (disorder)
	609562003	Maturity onset diabetes of the young, type 1 (disorder)
	700414001	Education about diabetes and driving
	706891008	Neuropathy due to brittle type I diabetes mellitus
	706894000	Retinopathy due to unstable diabetes mellitus type 1 (disorder)
	707221002	Diabetic glomerulosclerosis
	712882000	Diabetic autonomic neuropathy due to type 1 diabetes mellitus
	712883005	Diabetic autonomic neuropathy due to type 2 diabetes mellitus
	713130008	Assessment of diabetic foot ulcer
	713150007	Diabetic foot ulcer prevention (regime/therapy)
	713457002	Neovascular glaucoma due to diabetes mellitus (disorder)
	713702000	Gastroparesis due to type 1 diabetes mellitus
	713703005	Gastroparesis due to type 2 diabetes mellitus (disorder)
	713704004	Gastroparesis due to diabetes mellitus (disorder)
	713705003	Polyneuropathy due to type 1 diabetes mellitus
	713706002	Polyneuropathy due to type 2 diabetes mellitus
	715759002	Provision of written information about diabetes and hypertension (procedure)
	715879000	Provision of written information about diabetes and high cholesterol (procedure)
	715894001	Provision of written information about diabetes mellitus (procedure)
	719216001	Hypoglycemic coma co-occurrent and due to diabetes mellitus type II (disorder)
	719943000	Provision of written information about diabetes and high hemoglobin A1c level (procedure)
	721283000	Acidosis due to type 1 diabetes mellitus (disorder)
	721284006	Acidosis due to type 2 diabetes mellitus (disorder)
	722161008	Diabetic retinal eye exam (procedure)
	723074006	Renal papillary necrosis due to diabetes mellitus (disorder)
	724136006	Diabetic mastopathy
	724810001	Radiculoplexoneuropathy due to diabetes mellitus (disorder)
	724876003	Lesion of skin co-occurrent and due to diabetes mellitus (disorder)
	724997001	Lumbosacral plexopathy co-occurrent and due to diabetes mellitus (disorder)
	735200002	Absence of lower limb due to diabetes mellitus (disorder)
	735537007	Hyperosmolar hyperglycemic coma due to diabetes mellitus without ketoacidosis (disorder)
	735538002	Lactic acidosis co-occurrent and due to diabetes mellitus (disorder)
	735539005	Metabolic acidosis co-occurrent and due to diabetes mellitus (disorder)
	739681000	Diabetic oculopathy due to type I diabetes mellitus
	762489000	Acute complication with diabetes mellitus
	768792007	Cataract of right eye co-occurrent and due to diabetes mellitus
	768793002	Diabetic cataract of left eye
	768794008	Bilateral diabetic cataracts
	768797001	Iritis of right eye co-occurrent and due to diabetes mellitus
	768798006	Iritis of left eye co-occurrent and due to diabetes mellitus
	768799003	Iritis of bilateral eyes co-occurrent and due to diabetes mellitus (disorder)
	769181007	Preproliferative retinopathy of right eye co-occurrent and due to diabetes mellitus (disorder)
	769182000	Preproliferative diabetic retinopathy of left eye
	769183005	Mild nonproliferative diabetic retinopathy of right eye
	769184004	Mild nonproliferative retinopathy of left eye
	769185003	Moderate non-proliferative diabetic retinopathy of right eye
	769186002	Moderate nonproliferative diabetic retinopathy of left eye
	769187006	Severe nonproliferative diabetic retinopathy of right eye
	769188001	Severe nonproliferative retinopathy of left eye co-occurrent and due to diabetes mellitus (disorder)
	769190000	Very severe nonproliferative diabetic retinopathy of right eye
	769191001	Very severe nonproliferative diabetic retinopathy of left eye
	769217008	Diabetic macular edema of right eye
	769218003	Macular oedema of left eye co-occurrent and due to diabetes mellitus
	769219006	Macular edema due to type 1 diabetes mellitus
	769220000	Macular edema co-occurrent and due to type 2 diabetes mellitus
	769221001	Clinically significant macular edema of right eye co-occurrent and due to diabetes mellitus (disorder)
	769222008	Clinically significant macular oedema of left eye co-occurrent and due to diabetes mellitus
	769244003	Diabetic maculopathy of right eye
	769245002	Disorder of left macula co-occurrent and due to diabetes mellitus (disorder)
	770094004	Cervical radiculoplexus neuropathy co-occurrent and due to diabetes mellitus (disorder)
	770095003	Cranial nerve palsy due to diabetes mellitus
	770096002	Erectile dysfunction co-occurrent and due to diabetes mellitus (disorder)
	770097006	Clinically significant macular edema co-occurrent and due to diabetes mellitus (disorder)
	770098001	Cranial nerve palsy with type 1 diabetes mellitus
	770323005	Retinal edema due to diabetes mellitus

	770324004	Retinal ischemia due to diabetes mellitus
	770361008	Vitreous hemorrhage of right eye co-occurrent and due to diabetes mellitus
	770362001	Vitreous haemorrhage of left eye with diabetes mellitus
	770581008	Diabetic retinal microaneurysm of right eye
	770582001	Retinal microaneurysm of left eye co-occurrent and due to diabetes mellitus
	770599000	Retinal venous beading of right eye co-occurrent and due to diabetes mellitus
	770600002	Retinal venous beading of left eye with diabetes mellitus
	770765001	Proliferative diabetic retinopathy of right eye
	770766000	Proliferative diabetic retinopathy of left eye
	201000119106	Disorder associated with well controlled type 2 diabetes mellitus
	691000119103	Erectile dysfunction associated with type I diabetes mellitus
	701000119103	Mixed hyperlipidaemia associated with type II diabetes mellitus
	711000119100	Diabetic stage 5 chronic renal impairment associated with type 2 diabetes mellitus
	721000119107	Diabetic stage 4 chronic renal impairment associated with type 2 diabetes mellitus
	731000119105	Chronic kidney disease stage 3 associated with type 2 diabetes mellitus
	741000119101	Chronic kidney disease stage 2 associated with type 2 diabetes mellitus
	751000119104	Chronic kidney disease stage 1 associated with type 2 diabetes mellitus
	761000119102	Diabetic dyslipidemia associated with type 2 diabetes mellitus
	771000119108	Chronic renal impairment associated with type II diabetes mellitus
	781000119106	Diabetic neuropathic arthropathy associated with type 2 diabetes mellitus
	791000119109	Angina associated with type II diabetes mellitus
	1491000119102	Diabetic vitreous haemorrhage associated with type II diabetes mellitus
	1501000119109	Proliferative diabetic retinopathy associated with type II diabetes mellitus
	1511000119107	Diabetic peripheral neuropathy associated with type II diabetes mellitus
	1521000119100	Diabetic foot ulcer associated with type 2 diabetes mellitus
	1531000119102	Diabetic dermopathy associated with diabetes mellitus type 2
	1541000119106	Diabetic skin ulcer associated with type 2 diabetes mellitus
	1551000119108	Nonproliferative diabetic retinopathy associated with type II diabetes mellitus
	1561000119105	Diabetic peripheral neuropathy associated with type I diabetes mellitus
	1571000119104	Mixed hyperlipidemia associated with type 1 diabetes mellitus (disorder)
	4551000175107	Diabetic foot exam not done
	4581000175103	Diabetic retinal eye exam not done (situation)
	12811000119100	Complication due to diabetes mellitus type 2
	18521000119106	Microalbuminuria due to type 1 diabetes mellitus
	28331000119107	Retinal oedema due to type 2 diabetes mellitus
	31211000119101	Peripheral vascular disease due to type I diabetes
	41911000119107	Glaucoma due to type 2 diabetes mellitus (disorder)
	60951000119105	Blindness due to type 2 diabetes mellitus
	60961000119107	Nonproliferative diabetic retinopathy due to type 1 diabetes mellitus
	60971000119101	Proliferative retinopathy due to type 1 diabetes mellitus
	60991000119100	Blindness due to type 1 diabetes mellitus (disorder)
	71421000119105	Hypertension in chronic kidney disease due to type 2 diabetes mellitus (disorder)
	71441000119104	Nephrotic syndrome due to type 2 diabetes mellitus
	71701000119105	Hypertension in chronic kidney disease due to type 1 diabetes mellitus
	71721000119101	Nephrotic syndrome due to type 1 diabetes mellitus
	71771000119100	Type 1 diabetes mellitus with neuropathic arthropathy
	71791000119104	Peripheral neuropathy due to type 1 diabetes mellitus
	72021000119109	Diabetic dermopathy due to type 1 diabetes mellitus
	72031000119107	Severe malnutrition due to type 1 diabetes mellitus (disorder)
	72041000119103	Osteomyelitis due to type 1 diabetes mellitus (disorder)
	72051000119101	Severe malnutrition due to type 2 diabetes mellitus
	72061000119104	Osteomyelitis due to type 2 diabetes mellitus (disorder)
	72141000119104	Chronic ulcer of skin due to type 1 diabetes mellitus
	82541000119100	Traction retinal detachment due to type 2 diabetes mellitus
	82551000119103	Rubeosis iridis due to type 2 diabetes mellitus
	82571000119107	Traction retinal detachment due to type 1 diabetes mellitus
	82581000119105	Rubeosis iridis due to type 1 diabetes mellitus
	84361000119102	Insulin reactive hypoglycemia in type 2 diabetes mellitus
	84371000119108	Hypoglycemia due to type 1 diabetes mellitus
	87441000119104	Ankle ulcer due to type 2 diabetes mellitus (disorder)
	87451000119102	Heel AND/OR midfoot ulcer due to type 2 diabetes mellitus
	87461000119100	Forefoot ulcer due to type 2 diabetes mellitus
	87471000119106	Ankle ulcer due to type 1 diabetes mellitus (disorder)
	87481000119109	Heel AND/OR midfoot ulcer due to type 1 diabetes mellitus
	87491000119107	Forefoot ulcer due to type 1 diabetes mellitus
	87921000119104	Cranial nerve palsy due to type 2 diabetes mellitus
	90721000119101	Chronic kidney disease stage 1 due to type 1 diabetes mellitus (disorder)
	90731000119103	Chronic kidney disease stage 2 due to type 1 diabetes mellitus
	90741000119107	Chronic kidney disease stage 3 due to type 1 diabetes mellitus
	90751000119109	Chronic kidney disease stage 4 due to type 1 diabetes mellitus
	90761000119106	Chronic kidney disease stage 5 due to type 1 diabetes mellitus (disorder)
	90771000119100	End stage renal disease on dialysis due to type 1 diabetes mellitus (disorder)
	90781000119102	Microalbuminuria due to type 2 diabetes mellitus (disorder)
	90791000119104	End stage renal disease on dialysis due to type 2 diabetes mellitus (disorder)
	96441000119101	Chronic kidney disease due to type 1 diabetes mellitus
	97331000119101	Macular edema and retinopathy due to type 2 diabetes mellitus (disorder)
	97341000119105	Proliferative retinopathy with retinal oedema due to type 2 diabetes mellitus

	97621000119107	Stasis ulcer due to type 2 diabetes mellitus (disorder)
	102621000119101	Skin ulcer due to type 2 diabetes mellitus
	102781000119107	Sensory neuropathy due to type 1 diabetes mellitus (disorder)
	103981000119101	Proliferative diabetic retinopathy following surgery
	104941000119109	Retinal ischemia due to type 1 diabetes mellitus
	104951000119106	Vitreous haemorrhage due to type 1 diabetes mellitus
	104961000119108	Retinal ischemia due to type 2 diabetes mellitus
	108781000119105	Neuropathic ulcer of midfoot AND/OR heel due to type 2 diabetes mellitus (disorder)
	108791000119108	Suspected glaucoma due to type 2 diabetes mellitus (situation)
	109171000119104	Retinal oedema due to type 1 diabetes mellitus
	110141000119100	Ulcer of lower limb due to type 1 diabetes mellitus (disorder)
	110171000119107	Leg ulcer due to type 2 diabetes mellitus
	110181000119105	Peripheral sensory neuropathy due to type 2 diabetes mellitus
	111231000119109	Dyslipidemia with high density lipoprotein below reference range and triglyceride above reference range due to type 2 diabetes mellitus
	119831000119106	Hypoglycemia unawareness in type 2 diabetes mellitus (disorder)
	120711000119108	Hypoglycaemic unawareness in type 1 diabetes mellitus
	120731000119103	Hypoglycemia due to type 2 diabetes mellitus (disorder)
	127991000119101	Hypertension concurrent and due to end stage renal disease on dialysis due to type 2 diabetes mellitus
	128001000119105	Hypertension concurrent and due to end stage renal disease on dialysis due to type 1 diabetes mellitus (disorder)
	137931000119102	Hyperlipidemia due to type 2 diabetes mellitus
	137941000119106	Hyperlipidemia due to type 1 diabetes mellitus
	138881000119106	Mild nonproliferative retinopathy due to type 1 diabetes mellitus
	138891000119109	Moderate nonproliferative retinopathy due to type 1 diabetes mellitus (disorder)
	138911000119106	Mild nonproliferative retinopathy due to type 2 diabetes mellitus
	138921000119104	Moderate nonproliferative retinopathy due to type 2 diabetes mellitus (disorder)
	140101000119109	Hypertension in chronic kidney disease stage 5 due to type 2 diabetes mellitus
	140111000119107	Hypertension in chronic kidney disease stage 4 due to type 2 diabetes mellitus (disorder)
	140121000119100	Hypertension in chronic kidney disease stage 3 due to type 2 diabetes mellitus
	140131000119102	Hypertension in chronic kidney disease stage 2 due to type 2 diabetes mellitus (disorder)
	140381000119104	Neuropathic toe ulcer due to type 2 diabetes mellitus (disorder)
	140391000119101	Ulcer of toe due to type 2 diabetes mellitus (disorder)
	140521000119107	Ischemic foot ulcer due to type 2 diabetes mellitus (disorder)
	140531000119105	Neuropathic foot ulcer due to type 2 diabetes mellitus (disorder)
	142881000119105	History of nocturnal hypoglycaemia
	157141000119108	Proteinuria due to type 2 diabetes mellitus
	164881000119109	Foot ulcer due to type 1 diabetes mellitus (disorder)
	243421000119104	Proteinuria due to type 1 diabetes mellitus (disorder)
	324301000119107	Arthropathy due to metabolic disorder (disorder)
	367991000119101	Hyperglycemia due to type 1 diabetes mellitus
	368051000119109	Hyperglycemia due to type 2 diabetes mellitus (disorder)
	368521000119107	Disorder of nerve co-occurrent and due to type 1 diabetes mellitus (disorder)
	368551000119104	Dyslipidemia due to type 1 diabetes mellitus (disorder)
	368561000119102	Hyperosmolarity due to type 1 diabetes mellitus (disorder)
	368581000119106	Neuropathy due to type 2 diabetes mellitus (disorder)
	368591000119109	Cheiropathy due to type 2 diabetes mellitus (disorder)
	368601000119102	Hyperosmolar coma due to secondary diabetes mellitus
	368711000119106	Mild nonproliferative retinopathy due to secondary diabetes mellitus
	368721000119104	Non-proliferative retinopathy due to secondary diabetes mellitus (disorder)
	368741000119105	Moderate non-proliferative retinopathy due to secondary diabetes mellitus (disorder)
	430801000124103	Proliferative retinopathy (disorder)
	10656231000119100	Skin ulcer of toe due to diabetes mellitis type 1 (disorder)
	10656271000119100	Skin ulcer of toe due to diabetes mellitis type 2
	10660471000119100	Ulcer of left foot co-occurrent and due to diabetes mellitus type 2 (disorder)
	10661671000119100	Ulcer of right foot co-occurrent and due to diabetes mellitus type 2 (disorder)
	10995761000119100	History of diabetic foot ulcer (situation)
	1481000119100	Diabetes mellitus type 2 without retinopathy (disorder)
	164971000119101	Type 2 diabetes mellitus controlled by diet (finding)
	24481000000101	Type 2 diabetic on diet only (finding)
	335621000000101	Maternally inherited diabetes mellitus (disorder)
	359642000	Diabetes mellitus type 2 in nonobese (disorder)
	445353002	Brittle type II diabetes mellitus (finding)
	703138006	Type II diabetes mellitus in remission (disorder)
	81531005	Diabetes mellitus type 2 in obese (disorder)
	237627000	Pregnancy and type 2 diabetes mellitus (disorder)
	24471000000103	Type 2 diabetic on insulin (finding)
	279321000000104	Diabetes type 2 review (regime/therapy)
	444110003	Type II diabetes mellitus well controlled (finding)
	609567009	Pre-existing type 2 diabetes mellitus in pregnancy (disorder)
	754121000000107	Type II diabetic dietary review (regime/therapy)
	724205009	Laminopathy type Decaudain Vigouroux (disorder)
	1067201000000100	Eating disorder co-occurrent with diabetes mellitus type 1 (disorder)
	111307005	Leprechaunism syndrome (disorder)

	123763000	Houssay's syndrome (disorder)
	190416008	Steroid-induced diabetes mellitus without complication (disorder)
	198131000000101	Hypoglycaemic warning good (disorder)
	20678000	Extreme insulin resistance with acanthosis nigricans, hirsutism AND autoantibodies to the insulin receptors (disorder)
	237600004	Malnutrition-related diabetes mellitus - fibrocalculous (disorder)
	237601000	Secondary endocrine diabetes mellitus (disorder)
	237610008	Acrorenal field defect, ectodermal dysplasia, and lipoatrophic diabetes (disorder)
	237612000	Photomyoclonus, diabetes mellitus, deafness, nephropathy and cerebral dysfunction (disorder)
	237618001	Insulin-dependent diabetes mellitus secretory diarrhea syndrome (disorder)
	237632004	Hypoglycemic event in diabetes (disorder)
	267467004	Diabetes mellitus (& [ketoacidosis]) (disorder)
	2751001	Fibrocalculous pancreatic diabetes (disorder)
	279291000000109	Diabetes type 1 review (regime/therapy)
	33559001	Pineal hyperplasia AND diabetes mellitus syndrome (disorder)
	368171000119104	Dermatitis due to drug induced diabetes mellitus (disorder)
	385041000000108	Diabetes mellitus with multiple complications (disorder)
	385051000000106	Pre-existing diabetes mellitus (disorder)
	408539000	Insulin autoimmune syndrome (disorder)
	413183008	Diabetes mellitus caused by non-steroid drugs without complication (disorder)
	413184002	Fibrocalculous pancreatopathy without complication (disorder)
	609564002	Pre-existing type 1 diabetes mellitus in pregnancy (disorder)
	609565001	Permanent neonatal diabetes mellitus (disorder)
	609568004	Diabetes mellitus due to genetic defect in beta cell function (disorder)
	609571007	Maturity-onset diabetes of the young, type 4 (disorder)
	609572000	Maturity-onset diabetes of the young, type 5 (disorder)
	609575003	Maturity-onset diabetes of the young, type 8 (disorder)
	703136005	Diabetes mellitus in remission (disorder)
	721973006	Lipodystrophy, intellectual disability, deafness syndrome (disorder)
	722206009	Pancreatic hypoplasia, diabetes mellitus, congenital heart disease syndrome (disorder)
	722454003	Intellectual disability, craniofacial dysmorphism, hypogonadism, diabetes mellitus syndrome (disorder)
	734022008	Wolfram-like syndrome (disorder)
	737212004	Diabetes mellitus caused by chemical (disorder)
	763325000	Insulin resistance (disorder)
	771571000000102	History of secondary diabetes mellitus (situation)
	894741000000107	Hypoglycaemic warning absent (disorder)
	105401000119101	Diabetes mellitus due to pancreatic injury (disorder)
	106281000119103	Pre-existing diabetes mellitus in mother complicating childbirth (disorder)
	10754881000119100	Diabetes mellitus in mother complicating childbirth (disorder)
	1102351000000100	Ketosis-prone diabetes mellitus (disorder)
	112991000000101	Lipoatrophic diabetes mellitus without complication (disorder)
	190407009	Malnutrition-related diabetes mellitus with renal complications (disorder)
	190412005	Malnutrition-related diabetes mellitus without complications (disorder)
	190447002	Steroid-induced diabetes (disorder)
	198121000000103	Hypoglycaemic warning impaired (disorder)
	23045005	Insulin dependent diabetes mellitus type IA (disorder)
	237608006	Lipodystrophy, partial, with Rieger anomaly, short stature, and insulinopenic diabetes mellitus (disorder)
	237613005	Hyperproinsulinemia (disorder)
	237616002	Hypogonadism, diabetes mellitus, alopecia, mental retardation and electrocardiographic abnormalities (disorder)
	237617006	Megaloblastic anemia, thiamine-responsive, with diabetes mellitus and sensorineural deafness (disorder)
	237619009	Diabetes-deafness syndrome maternally transmitted (disorder)
	237622006	Poor glycemic control (disorder)
	237650006	Insulin resistance in diabetes (disorder)
	237651005	Insulin resistance - type A (disorder)
	237652003	Insulin resistance - type B (disorder)
	24203005	Extreme insulin resistance with acanthosis nigricans, hirsutism AND abnormal insulin receptors (disorder)
	267471001	Diabetes + eye manifestation (& [cataract] or [retinopathy]) (disorder)
	28032008	Insulin dependent diabetes mellitus type IB (disorder)
	284449005	Congenital total lipodystrophy (disorder)
	303059007	Postpancreatectomy hypoinsulinemia (disorder)
	31321000119102	Diabetes mellitus type 1 without retinopathy (disorder)
	367261000119100	Hyperosmolarity co-occurrent and due to drug induced diabetes mellitus (disorder)
	408540003	Diabetes mellitus caused by non-steroid drugs (disorder)
	427089005	Diabetes mellitus due to cystic fibrosis (disorder)
	42954008	Diabetes mellitus associated with receptor abnormality (disorder)
	444074000	Type I diabetes mellitus well controlled (finding)
	445260006	Posttransplant diabetes mellitus (disorder)
	530558861000132000	Atypical diabetes mellitus (disorder)
	532411000000102	Diabetes mellitus, adult onset, with no mention of complication (disorder)

5368009	Drug-induced diabetes mellitus (disorder)
57886004	Protein-deficient diabetes mellitus (disorder)
59079001	Diabetes mellitus associated with hormonal etiology (disorder)
5969009	Diabetes mellitus associated with genetic syndrome (disorder)
609563008	Pre-existing diabetes mellitus in pregnancy (disorder)
609566000	Pregnancy and type 1 diabetes mellitus (disorder)
609569007	Diabetes mellitus due to genetic defect in insulin action (disorder)
609570008	Maturity-onset diabetes of the young, type 3 (disorder)
609573005	Maturity-onset diabetes of the young, type 6 (disorder)
609574004	Maturity-onset diabetes of the young, type 7 (disorder)
609576002	Maturity-onset diabetes of the young, type 9 (disorder)
609577006	Maturity-onset diabetes of the young, type 10 (disorder)
609578001	Maturity-onset diabetes of the young, type 11 (disorder)
658011000000104	Diabetes mellitus with other specified manifestation (disorder)
703137001	Type I diabetes mellitus in remission (disorder)
70694009	Diabetes mellitus AND insipidus with optic atrophy AND deafness (disorder)
709147009	Gingivitis co-occurrent with diabetes mellitus (disorder)
716362006	Gingival disease co-occurrent with diabetes mellitus (disorder)
720519003	Atherosclerosis, deafness, diabetes, epilepsy, nephropathy syndrome (disorder)
724067006	Permanent neonatal diabetes mellitus with cerebellar agenesis syndrome (disorder)
733072002	Alaninuria, microcephaly, dwarfism, enamel hypoplasia, diabetes mellitus syndrome (disorder)
735199000	History of diabetes related lower limb amputation (situation)
754101000000103	Type I diabetic dietary review (regime/therapy)
754461000000105	Referral to type I diabetes structured education programme (procedure)
75682002	Diabetes mellitus caused by insulin receptor antibodies (disorder)
773001000000103	Symptomatic diabetic peripheral neuropathy (disorder)
775841000000109	Diabetic retinopathy detected by national screening programme (disorder)
80660001	Mauriac's syndrome (disorder)
83728000	Polyglandular autoimmune syndrome, type 2 (disorder)
91352004	Diabetes mellitus due to structurally abnormal insulin (disorder)
10154911000001100	Glimepiride 1mg tablets (Alpharma Ltd) (product)
10155111000001100	Glimepiride 2mg tablets (Alpharma Ltd) (product)
10155311000001100	Glimepiride 3mg tablets (Alpharma Ltd) (product)
10155511000001100	Glimepiride 4mg tablets (Alpharma Ltd) (product)
10272611000001100	Glimepiride 1mg tablets (Unichem Plc) (product)
10273811000001100	Glimepiride 1mg tablets (A A H Pharmaceuticals Ltd) (product)
10274311000001100	Glimepiride 3mg tablets (A A H Pharmaceuticals Ltd) (product)
10274511000001100	Glimepiride 4mg tablets (A A H Pharmaceuticals Ltd) (product)
10293511000001100	Metformin 850mg tablets (Relonchem Ltd) (product)
10377311000001100	Glibenclamide 2.5mg tablets (Arrow Generics Ltd) (product)
10377711000001100	Glibenclamide 5mg tablets (Arrow Generics Ltd) (product)
10378111000001100	Gliclazide 80mg tablets (Arrow Generics Ltd) (product)
10390211000001100	Metformin 500mg tablets (Arrow Generics Ltd) (product)
10390811000001100	Metformin 850mg tablets (Arrow Generics Ltd) (product)
10437311000001100	Glimepiride 4mg tablets (Niche Generics Ltd) (product)
10458811000001100	Actos 15mg tablets (PI) (Dowelhurst Ltd) (product)
10465111000001100	Avandia 8mg tablets (PI) (Dowelhurst Ltd) (product)
10486511000001100	NovoNorm 500microgram tablets (PI) (Dowelhurst Ltd) (product)
10487311000001100	NovoNorm 2mg tablets (PI) (Dowelhurst Ltd) (product)
10488111000001100	Avandamet 4mg/1000mg tablets (PI) (Waymade Ltd) (product)
10517711000001100	Glimepiride 1mg tablets (Winthrop Pharmaceuticals UK Ltd) (product)
10518111000001100	Glimepiride 3mg tablets (Winthrop Pharmaceuticals UK Ltd) (product)
10518311000001100	Glimepiride 4mg tablets (Winthrop Pharmaceuticals UK Ltd) (product)
10539211000001100	Glimepiride 1mg tablets (PI) (Waymade Ltd) (product)
10539511000001100	Glimepiride 2mg tablets (PI) (Waymade Ltd) (product)
10539711000001100	Glimepiride 3mg tablets (PI) (Waymade Ltd) (product)
10540011000001100	Glimepiride 4mg tablets (PI) (Waymade Ltd) (product)
10542811000001100	Humalog 100units/ml solution for injection 10ml vials (PI) (Waymade Ltd) (product)
10543211000001100	NovoRapid FlexPen 100units/ml solution for injection 3ml pre-filled pens (Waymade Healthcare Plc) (product)
10548211000001100	Lantus 100units/ml solution for injection 10ml vials (PI) (Waymade Ltd) (product)
10548611000001100	Lantus 100units/ml solution for injection 3ml pre-filled OptiSet pens (Waymade Healthcare Plc) (product)
10549011000001100	Lantus 100units/ml solution for injection 3ml cartridges (PI) (Waymade Ltd) (product)
10617511000001100	Glimepiride 2mg tablets (Somex Pharma) (product)
10689011000001100	Glimepiride 2mg tablets (Pliva Pharma Ltd) (product)
10689211000001100	Glimepiride 3mg tablets (Pliva Pharma Ltd) (product)
10741611000001100	Metsol 500mg/5ml oral solution (Orbis Consumer Products Ltd) (product)

10750111000001100	Metformin 500mg/5ml oral solution sugar free (product)
10832711000001100	Metformin 500mg/5ml oral solution sugar free (Unichem Plc) (product)
10898411000001100	Apidra 100units/ml solution for injection 3ml OptiClik cartridges (sanofi-aventis) (product)
10922311000001100	Competact 15mg/850mg tablets (Takeda UK Ltd) (product)
10956911000001100	Prandin 1mg tablets (Novo Nordisk Ltd) (product)
10963611000001100	Metformin 500mg/5ml oral solution sugar free (A A H Pharmaceuticals Ltd) (product)
11018411000001100	Glimepiride 4mg tablets (Dr Reddy's Labs) (product)
11018911000001100	Glimepiride 2mg tablets (Dr Reddy's Labs) (product)
11019211000001100	Glimepiride 1mg tablets (Dr Reddy's Labs) (product)
11114611000001100	Levemir Penfill 100units/ml solution for injection 3ml cartridges (Waymade Ltd) (product)
11116211000001100	Pork Mixtard 30 100units/ml suspension for injection 10ml vials (Waymade Ltd) (product)
11118911000001100	Hypurin Bovine Neutral 100units/ml solution for injection 10ml vials (Waymade Ltd) (product)
11121911000001100	Velosulin 100units/ml solution for injection 10ml vials (Waymade Ltd) (product)
11124011000001100	Mixtard 30 100units/ml suspension for injection 10ml vials (Waymade Ltd) (product)
11124411000001100	Mixtard 30 InnoLet 100units/ml suspension for injection 3ml pre-filled pens (Waymade Healthcare Plc) (product)
11134711000001100	Insulatard 100units/ml suspension for injection 10ml vials (Waymade Ltd) (product)
11135611000001100	Mixtard 10 Penfill 100units/ml suspension for injection 3ml cartridges (Waymade Ltd) (product)
11148111000001100	Levemir InnoLet 100units/ml solution for injection 3ml pre-filled pens (Novo Nordisk Ltd) (product)
11149211000001100	Glimepiride 1mg tablets (Teva UK Ltd) (product)
11149411000001100	Glimepiride 2mg tablets (Teva UK Ltd) (product)
11149611000001100	Glimepiride 3mg tablets (Teva UK Ltd) (product)
11342811000001100	Glimepiride 1mg tablets (Sandoz Ltd) (product)
11343511000001100	Glimepiride 3mg tablets (Sandoz Ltd) (product)
11406411000001100	Gliclazide 80mg tablets (Pliva Pharma Ltd) (product)
11473711000001100	Januvia 100mg tablets (Merck Sharp & Dohme Ltd) (product)
11492211000001100	Gliclazide 80mg tablets (Winthrop Pharmaceuticals UK Ltd) (product)
11494611000001100	Byetta 10micrograms/0.04ml solution for injection 2.4ml pre-filled pens (AstraZeneca UK Ltd) (product)
11494811000001100	Byetta 5micrograms/0.02ml solution for injection 1.2ml pre-filled pens (AstraZeneca UK Ltd) (product)
11766011000001100	Prandin 1mg tablets (Waymade Healthcare Plc) (product)
11766211000001100	Prandin 2mg tablets (Waymade Healthcare Plc) (product)
11933011000001100	Lantus 100units/ml solution for injection 3ml pre-filled SoloStar pens (Sanofi) (product)
12107511000001100	Glibenclamide 625micrograms/5ml oral solution (Special Order) (product)
12108411000001100	Glibenclamide 2.5mg/5ml oral suspension (Special Order) (product)
12108711000001100	Glibenclamide 2.5mg/5ml oral solution (Special Order) (product)
12109911000001100	Gliclazide 50mg/5ml oral suspension (Special Order) (product)
12110211000001100	Gliclazide 60mg/5ml oral suspension (Special Order) (product)
12135411000001100	Glibenclamide 15mg/5ml oral suspension (product)
12135511000001100	Glibenclamide 2.5mg/5ml oral solution (product)
12135611000001100	Glibenclamide 2.5mg/5ml oral suspension (product)
12135811000001100	Glibenclamide 625micrograms/5ml oral suspension (product)
12135911000001100	Gliclazide 100mg/5ml oral suspension (product)
12136111000001100	Gliclazide 20mg/5ml oral suspension (product)
12136211000001100	Gliclazide 50mg/5ml oral suspension (product)
12144611000001100	Apidra 100units/ml solution for injection 3ml pre-filled SoloStar pens (Sanofi) (product)
12147311000001100	Glimepiride 2mg tablets (Kent Pharmaceuticals Ltd) (product)
12200111000001100	Repaglinide 500microgram tablets (PI) (Waymade Healthcare Plc) (product)
12200311000001100	Repaglinide 1mg tablets (PI) (Waymade Healthcare Plc) (product)
12200511000001100	Repaglinide 2mg tablets (PI) (Waymade Healthcare Plc) (product)
12798011000001100	Metformin 1g/5ml oral solution (product)
13100611000001100	Avandamet 4mg/1000mg tablets (Dowelhurst Ltd) (product)
13180611000001100	NovoMix 30 Penfill 100units/ml suspension for injection 3ml cartridges (Dowelhurst Ltd) (product)
13186611000001100	Prandin 0.5mg tablets (Dowelhurst Ltd) (product)
13318011000001100	Tolbutamide 250mg/5ml oral suspension (Special Order) (product)
13411611000001100	Galvus 50mg tablets (Novartis Pharmaceuticals UK Ltd) (product)
13413111000001100	Metformin 850mg / Vildagliptin 50mg tablets (product)
13456711000001100	Nazdol MR 30mg tablets (Consilient Health Ltd) (product)
134587001	Gliclazide 30mg m/r tablet (product)
134608004	Product containing precisely nateglinide 180 milligram/1 each conventional release oral tablet (clinical drug)
13462811000001100	Gliclazide 30mg/5ml oral suspension (Special Order) (product)

13466811000001100	Gliclazide 30mg/5ml oral suspension (product)
13511000001103	Diabetamide 2.5mg tablets (Ashbourne Pharmaceuticals Ltd) (product)
13549911000001100	Gliclazide 30mg modified-release tablets (Kent Pharmaceuticals Ltd) (product)
13613911000001100	Gliclazide 30mg modified-release tablets (A A H Pharmaceuticals Ltd) (product)
13626011000001100	Niddaryl 1mg tablets (Dee Pharmaceuticals Ltd) (product)
13627211000001100	Niddaryl 4mg tablets (Dee Pharmaceuticals Ltd) (product)
13720911000001100	Glimepiride 3mg tablets (Ranbaxy (UK) Ltd) (product)
13748611000001100	Glucophage SR 750mg tablets (Merck Serono Ltd) (product)
13756411000001100	Metformin 500mg tablets (Tillomed Laboratories Ltd) (product)
13772611000001100	Glucophage SR 500mg tablets (Waymade Healthcare Plc) (product)
13823711000001100	Actos 15mg tablets (Doncaster Pharmaceuticals Ltd) (product)
13823911000001100	Actos 30mg tablets (Doncaster Pharmaceuticals Ltd) (product)
13833311000001100	Avandamet 2mg/1000mg tablets (Doncaster Pharmaceuticals Ltd) (product)
13833511000001100	Avandamet 4mg/1000mg tablets (Doncaster Pharmaceuticals Ltd) (product)
13859411000001100	Insulin human 100units/ml solution for injection 10ml vials (product)
13864511000001100	Diamicron 30mg MR tablets (Doncaster Pharmaceuticals Ltd) (product)
13877811000001100	Humalog KwikPen 100units/ml solution for injection 3ml pre-filled pens (Eli Lilly and Company Ltd) (product)
13884911000001100	Humalog Mix50 KwikPen 100units/ml suspension for injection 3ml pre-filled pens (Eli Lilly and Company Ltd) (product)
14040211000001100	Actos 15mg tablets (Sigma Pharmaceuticals Plc) (product)
14055711000001100	Amaryl 4mg tablets (Sigma Pharmaceuticals Plc) (product)
14056111000001100	Amaryl 1mg tablets (Sigma Pharmaceuticals Plc) (product)
14061411000001100	Amaryl 3mg tablets (Sigma Pharmaceuticals Plc) (product)
14122111000001100	Glimepiride 1mg tablets (Consilient Health Ltd) (product)
14122511000001100	Glimepiride 3mg tablets (Consilient Health Ltd) (product)
14190111000001100	Humulin S 100units/ml solution for injection 10ml vials (Waymade Healthcare Plc) (product)
14190311000001100	Humulin M3 100units/ml suspension for injection 3ml cartridges (Waymade Healthcare Plc) (product)
14200411000001100	Avandamet 2mg/500mg tablets (Sigma Pharmaceuticals Plc) (product)
14200811000001100	Avandia 4mg tablets (Sigma Pharmaceuticals Plc) (product)
14201211000001100	Avandia 8mg tablets (Sigma Pharmaceuticals Plc) (product)
14247811000001100	Humalog 100units/ml solution for injection 3ml cartridges (Sigma Pharmaceuticals Plc) (product)
14248011000001100	Humalog Mix25 100units/ml suspension for injection 3ml cartridges (Sigma Pharmaceuticals Plc) (product)
14248811000001100	Humalog Mix50 Pen 100units/ml suspension for injection 3ml pre-filled pens (Sigma Pharmaceuticals Plc) (product)
14380511000001100	NovoMix 30 FlexPen 100units/ml suspension for injection 3ml pre-filled pens (Sigma Pharmaceuticals Plc) (product)
14386011000001100	NovoMix 30 Penfill 100units/ml suspension for injection 3ml cartridges (Sigma Pharmaceuticals Plc) (product)
14389911000001100	NovoRapid FlexPen 100units/ml solution for injection 3ml pre-filled pens (Sigma Pharmaceuticals Plc) (product)
14664711000001100	NovoRapid Penfill 100units/ml solution for injection 3ml cartridges (Sigma Pharmaceuticals Plc) (product)
14712111000001100	Mixtard 30 Penfill 100units/ml suspension for injection 3ml cartridges (Sigma Pharmaceuticals Plc) (product)
14723311000001100	Prandin 1mg tablets (Sigma Pharmaceuticals Plc) (product)
14723511000001100	Prandin 2mg tablets (Sigma Pharmaceuticals Plc) (product)
14753811000001100	Prandin 0.5mg tablets (Sigma Pharmaceuticals Plc) (product)
14760811000001100	Lantus 100units/ml solution for injection 3ml OptiClik cartridges (Sigma Pharmaceuticals Plc) (product)
14762411000001100	Lantus 100units/ml solution for injection 10ml vials (Sigma Pharmaceuticals Plc) (product)
14763111000001100	Lantus 100units/ml solution for injection 3ml pre-filled OptiSet pens (Sigma Pharmaceuticals Plc) (product)
14774311000001100	Metformin 850mg tablets (LPC Medical (UK) Ltd) (product)
147811000001105	Tolbutamide 500mg tablets (Kent Pharmaceuticals Ltd) (product)
15019811000001100	Metformin 500mg modified-release tablets (Kent Pharmaceuticals Ltd) (product)
15091011000001100	Glimepiride 1mg tablets (Sigma Pharmaceuticals Plc) (product)
15091411000001100	Glimepiride 3mg tablets (Sigma Pharmaceuticals Plc) (product)
15100211000001100	Nazdol MR 30mg tablets (Teva UK Ltd) (product)
15100511000001100	Nazdol MR 30mg tablets (Generics (UK) Ltd) (product)
152311000001101	NovoNorm 500microgram tablets (Novo Nordisk Pharmaceuticals Ltd) (product)
15374311000001100	Glucophage 1000mg oral powder sachets (Merck Serono Ltd) (product)
15411211000001100	Metformin 1g oral powder sachets sugar free (product)
15603111000001100	Humulin R 500units/ml solution for injection 20ml vials (Imported (United States)) (product)
15858611000001100	Victoza 6mg/ml solution for injection 3ml pre-filled pens (Novo Nordisk Ltd) (product)

15871011000001100	Insulin human 100units/ml solution for injection 10ml vials (Special Order) (product)
15973711000001100	Metformin 850mg tablets (Zanza Laboratories Ltd) (product)
16053711000001100	Glimepiride 2mg tablets (Bristol Laboratories Ltd) (product)
16054111000001100	Glimepiride 4mg tablets (Bristol Laboratories Ltd) (product)
16061411000001100	Metformin 500mg tablets (Bristol Laboratories Ltd) (product)
16125411000001100	Glimepiride 2mg/5ml oral solution (Special Order) (product)
16130611000001100	Glimepiride 2mg/5ml oral solution (product)
16130711000001100	Glimepiride 2mg/5ml oral suspension (product)
16133711000001100	Actos 30mg tablets (Lexon (UK) Ltd) (product)
16137211000001100	Amaryl 1mg tablets (Lexon (UK) Ltd) (product)
16137411000001100	Amaryl 2mg tablets (Lexon (UK) Ltd) (product)
16152911000001100	Competact 15mg/850mg tablets (Lexon (UK) Ltd) (product)
16187411000001100	Glucophage SR 500mg tablets (Lexon (UK) Ltd) (product)
16225011000001100	Prandin 0.5mg tablets (Lexon (UK) Ltd) (product)
16256311000001100	Actos 30mg tablets (Mawdsley-Brooks & Company Ltd) (product)
16259811000001100	Amaryl 1mg tablets (Mawdsley-Brooks & Company Ltd) (product)
16271611000001100	Avandamet 2mg/1000mg tablets (Mawdsley-Brooks & Company Ltd) (product)
16272811000001100	Avandia 4mg tablets (Mawdsley-Brooks & Company Ltd) (product)
16517211000001100	Humalog Pen 100units/ml solution for injection 3ml pre-filled pens (Mawdsley-Brooks & Company Ltd) (product)
16517411000001100	Competact 15mg/850mg tablets (Mawdsley-Brooks & Company Ltd) (product)
16517711000001100	Humalog Mix25 100units/ml suspension for injection 3ml cartridges (Mawdsley-Brooks & Company Ltd) (product)
16518911000001100	Humalog Mix50 100units/ml suspension for injection 3ml cartridges (Mawdsley-Brooks & Company Ltd) (product)
16519611000001100	Humalog Mix50 Pen 100units/ml suspension for injection 3ml pre-filled pens (Mawdsley-Brooks & Company Ltd) (product)
16520011000001100	Humulin I 100units/ml suspension for injection 3ml cartridges (Mawdsley-Brooks & Company Ltd) (product)
16520211000001100	Humulin I 100units/ml suspension for injection 10ml vials (Mawdsley-Brooks & Company Ltd) (product)
16521011000001100	Humulin M3 100units/ml suspension for injection 3ml cartridges (Mawdsley-Brooks & Company Ltd) (product)
16521311000001100	Humulin S 100units/ml solution for injection 3ml cartridges (Mawdsley-Brooks & Company Ltd) (product)
16523211000001100	NovoMix 30 FlexPen 100units/ml suspension for injection 3ml pre-filled pens (Mawdsley-Brooks & Company Ltd) (product)
16523811000001100	NovoMix 30 Penfill 100units/ml suspension for injection 3ml cartridges (Mawdsley-Brooks & Company Ltd) (product)
16530311000001100	Humalog Mix25 100units/ml suspension for injection 10ml vials (Eli Lilly and Company Ltd) (product)
16533211000001100	Januvia 100mg tablets (Mawdsley-Brooks & Company Ltd) (product)
16536211000001100	Dacadis MR 30mg tablets (Generics (UK) Ltd) (product)
16548811000001100	Insulin lispro biphasic 25/75 100units/ml suspension for injection 10ml vials (product)
16677511000001100	Zicron 40mg tablets (Bristol Laboratories Ltd) (product)
16701111000001100	Metformin 500mg tablets (Pfizer Ltd) (product)
17060511000001100	Janumet 50mg/1000mg tablets (Merck Sharp & Dohme Ltd) (product)
17071811000001100	Metformin 1g / Sitagliptin 50mg tablets (product)
17420011000001100	Prandin 0.5mg tablets (Mawdsley-Brooks & Company Ltd) (product)
17455811000001100	Starlix 60mg tablets (Mawdsley-Brooks & Company Ltd) (product)
17510111000001100	Humalog 100units/ml solution for injection 10ml vials (Mawdsley-Brooks & Company Ltd) (product)
17510311000001100	Insulatard InnoLet 100units/ml suspension for injection 3ml pre-filled pens (Mawdsley-Brooks & Company Ltd) (product)
17510511000001100	Lantus 100units/ml solution for injection 3ml cartridges (Mawdsley-Brooks & Company Ltd) (product)
17510711000001100	Lantus 100units/ml solution for injection 3ml OptiClik cartridges (Mawdsley-Brooks & Company Ltd) (product)
17536111000001100	Competact 15mg/850mg tablets (Sigma Pharmaceuticals Plc) (product)
17559311000001100	Humulin I 100units/ml suspension for injection 10ml vials (Sigma Pharmaceuticals Plc) (product)
17572311000001100	Actos 15mg tablets (Necessity Supplies Ltd) (product)
17572711000001100	Actos 30mg tablets (Necessity Supplies Ltd) (product)
17582511000001100	Amaryl 3mg tablets (Necessity Supplies Ltd) (product)
17595011000001100	Avandia 8mg tablets (Necessity Supplies Ltd) (product)
17602211000001100	NovoRapid Penfill 100units/ml solution for injection 3ml cartridges (Necessity Supplies Ltd) (product)
17608511000001100	Humulin I KwikPen 100units/ml suspension for injection 3ml pre-filled pens (Eli Lilly and Company Ltd) (product)
17622211000001100	Prandin 1mg tablets (Necessity Supplies Ltd) (product)
17622711000001100	Prandin 2mg tablets (Necessity Supplies Ltd) (product)
17895211000001100	Actos 45mg tablets (Sigma Pharmaceuticals Plc) (product)

17915111000001100	Glibenclamide 5mg tablets (Phoenix Healthcare Distribution Ltd) (product)
17916311000001100	Glimepiride 1mg tablets (Phoenix Healthcare Distribution Ltd) (product)
17916711000001100	Glimepiride 2mg tablets (Phoenix Healthcare Distribution Ltd) (product)
17917111000001100	Glimepiride 3mg tablets (Phoenix Healthcare Distribution Ltd) (product)
17917511000001100	Glimepiride 4mg tablets (Phoenix Healthcare Distribution Ltd) (product)
17940411000001100	Metformin 850mg tablets (Phoenix Healthcare Distribution Ltd) (product)
18048711000001100	Humalog Mix25 KwikPen 100units/ml suspension for injection 3ml pre-filled pens (Sigma Pharmaceuticals Plc) (product)
18048911000001100	Humulin M3 100units/ml suspension for injection 10ml vials (Sigma Pharmaceuticals Plc) (product)
18049911000001100	Actos 15mg tablets (Lexon (UK) Ltd) (product)
18061311000001100	Januvia 100mg tablets (Lexon (UK) Ltd) (product)
18083911000001100	Insulin human 100units/ml solution for injection 3.15ml cartridges (product)
18141911000001100	Metabet SR 500mg tablets (Morningside Healthcare Ltd) (product)
18251011000001100	Diamicron 30mg MR tablets (Necessity Supplies Ltd) (product)
18265411000001100	Glucobay 50mg tablets (Necessity Supplies Ltd) (product)
18266611000001100	Humalog 100units/ml solution for injection 3ml cartridges (Necessity Supplies Ltd) (product)
18266811000001100	Humalog 100units/ml solution for injection 10ml vials (Necessity Supplies Ltd) (product)
18286111000001100	Humalog Mix50 100units/ml suspension for injection 3ml cartridges (Waymade Healthcare Plc) (product)
18309611000001100	Acarbose 50mg tablets (A A H Pharmaceuticals Ltd) (product)
18462411000001100	Glimepiride 1mg tablets (Accord Healthcare Ltd) (product)
18490511000001100	Lantus 100units/ml solution for injection 3ml cartridges (Necessity Supplies Ltd) (product)
18490711000001100	Lantus 100units/ml solution for injection 3ml OptiClik cartridges (Necessity Supplies Ltd) (product)
18491611000001100	Lantus 100units/ml solution for injection 3ml pre-filled SoloStar pens (Necessity Supplies Ltd) (product)
18491811000001100	Lantus 100units/ml solution for injection 10ml vials (Necessity Supplies Ltd) (product)
18544011000001100	Actos 45mg tablets (Doncaster Pharmaceuticals Ltd) (product)
18553711000001100	Humalog KwikPen 100units/ml solution for injection 3ml pre-filled pens (Waymade Healthcare Plc) (product)
18553911000001100	Humalog Mix25 KwikPen 100units/ml suspension for injection 3ml pre-filled pens (Waymade Healthcare Plc) (product)
18554111000001100	Humalog Mix50 KwikPen 100units/ml suspension for injection 3ml pre-filled pens (Waymade Healthcare Plc) (product)
18570911000001100	Competact 15mg/850mg tablets (Doncaster Pharmaceuticals Ltd) (product)
18586911000001100	Januvia 100mg tablets (Doncaster Pharmaceuticals Ltd) (product)
18596311000001100	Onglyza 2.5mg tablets (Bristol-Myers Squibb Pharmaceuticals Ltd) (product)
18624411000001100	Acarbose 100mg tablets (Phoenix Healthcare Distribution Ltd) (product)
18630911000001100	Lantus 100units/ml solution for injection 3ml pre-filled OptiSet pens (Mawdsley-Brooks & Company Ltd) (product)
18885611000001100	Metabet SR 1000mg tablets (Morningside Healthcare Ltd) (product)
19179211000001100	Repaglinide 500microgram tablets (Teva UK Ltd) (product)
19179511000001100	Repaglinide 1mg tablets (Teva UK Ltd) (product)
19179811000001100	Repaglinide 2mg tablets (Teva UK Ltd) (product)
19276711000001100	Repaglinide 2mg tablets (Aspire Pharma Ltd) (product)
19299911000001100	Repaglinide 500microgram tablets (Actavis UK Ltd) (product)
19300811000001100	Repaglinide 2mg tablets (Actavis UK Ltd) (product)
19306711000001100	Enyglid 0.5mg tablets (Consilient Health Ltd) (product)
19308111000001100	Glucient SR 500mg tablets (Consilient Health Ltd) (product)
19360311000001100	Repaglinide 500microgram tablets (Alliance Healthcare (Distribution) Ltd) (product)
19360811000001100	Repaglinide 1mg tablets (Alliance Healthcare (Distribution) Ltd) (product)
19361511000001100	Pioglitazone 30mg tablets (A A H Pharmaceuticals Ltd) (product)
19469211000001100	Pioglitazone 15mg tablets (Alliance Healthcare (Distribution) Ltd) (product)
19469411000001100	Pioglitazone 30mg tablets (Alliance Healthcare (Distribution) Ltd) (product)
19476211000001100	Pioglitazone 15mg tablets (Consilient Health Ltd) (product)
19489311000001100	Acarbose 50mg tablets (Alliance Healthcare (Distribution) Ltd) (product)
19492811000001100	Trajenta 5mg tablets (Boehringer Ingelheim Ltd) (product)
19525211000001100	Linagliptin 5mg tablets (product)
19573011000001100	Metformin 1g modified-release tablets (A A H Pharmaceuticals Ltd) (product)
19592911000001100	Pioglitazone 15mg tablets (Dr Reddy's Laboratories (UK) Ltd) (product)
19593111000001100	Pioglitazone 30mg tablets (Dr Reddy's Laboratories (UK) Ltd) (product)
19612811000001100	Galvus 50mg tablets (Doncaster Pharmaceuticals Ltd) (product)
19613111000001100	Humalog KwikPen 100units/ml solution for injection 3ml pre-filled pens (DE Pharmaceuticals) (product)
19613311000001100	Humalog Mix25 KwikPen 100units/ml suspension for injection 3ml pre-filled pens (DE Pharmaceuticals) (product)

19613711000001100	Humalog Mix25 100units/ml suspension for injection 3ml cartridges (Doncaster Pharmaceuticals Ltd) (product)
19613911000001100	Humalog Mix50 100units/ml suspension for injection 3ml cartridges (Doncaster Pharmaceuticals Ltd) (product)
19614311000001100	Humulin I 100units/ml suspension for injection 10ml vials (Doncaster Pharmaceuticals Ltd) (product)
19700011000001100	Acarbose 100mg tablets (Doncaster Pharmaceuticals Ltd) (product)
19700511000001100	Acarbose 50mg tablets (Doncaster Pharmaceuticals Ltd) (product)
19873911000001100	Eucreas 50mg/1000mg tablets (Doncaster Pharmaceuticals Ltd) (product)
19958011000001100	Glizofar 30mg tablets (Teva UK Ltd) (product)
19958211000001100	Glizofar 45mg tablets (Teva UK Ltd) (product)
20115111000001100	Januvia 25mg Tablets (Merck Sharp & Dohme Ltd) (product)
20133211000001100	Januvia 100mg tablets (Waymade Healthcare Plc) (product)
20163311000001100	NovoMix 30 Penfill 100units/ml suspension for injection 3ml cartridges (Doncaster Pharmaceuticals Ltd) (product)
20166811000001100	Glimepiride 2mg tablets (Accord Healthcare Ltd) (product)
202611000001102	Metformin 500mg tablets (A A H Pharmaceuticals Ltd) (product)
20357411000001100	Pioglitazone 15mg tablets (Actavis UK Ltd) (product)
20357811000001100	Pioglitazone 45mg tablets (Actavis UK Ltd) (product)
20552511000001100	Diagemet XL 500mg tablets (Genus Pharmaceuticals Ltd) (product)
20566511000001100	Repaglinide 1mg tablets (Creo Pharma Ltd) (product)
20777611000001100	Repaglinide 1mg tablets (Accord Healthcare Ltd) (product)
20916911000001100	Pioglitazone 15mg tablets (Zentiva) (product)
20917111000001100	Pioglitazone 30mg tablets (Zentiva) (product)
20917311000001100	Pioglitazone 45mg tablets (Zentiva) (product)
20946611000001100	Gliclazide 30mg modified-release tablets (Actavis UK Ltd) (product)
21022811000001100	Janumet 50mg/1000mg tablets (Waymade Healthcare Plc) (product)
21027911000001100	Acarbose 50mg tablets (Arrow Generics Ltd) (product)
21112511000001100	Acarbose 100mg tablets (Actavis UK Ltd) (product)
21203911000001100	Lantus 100units/ml solution for injection 3ml pre-filled SoloStar pens (Waymade Healthcare Plc) (product)
21324011000001100	Glimepiride 6mg/5ml oral suspension (Special Order) (product)
21609511000001100	Forxiga 5mg tablets (Bristol-Myers Squibb Pharmaceuticals Ltd) (product)
21609811000001100	Forxiga 10mg tablets (Bristol-Myers Squibb Pharmaceuticals Ltd) (product)
21734711000001100	Acarbose 50mg tablets (Waymade Healthcare Plc) (product)
21792811000001100	Metformin 500mg modified-release tablets (Waymade Healthcare Plc) (product)
21880311000001100	Repaglinide 1mg tablets (Waymade Healthcare Plc) (product)
21884111000001100	Tolbutamide 500mg tablets (Waymade Healthcare Plc) (product)
21928511000001100	Tresiba Penfill 100units/ml solution for injection 3ml cartridges (Novo Nordisk Ltd) (product)
21939511000001100	Insulin degludec 100units/ml solution for injection 3ml cartridges (product)
21941011000001100	Lyxumia 20micrograms/0.2ml solution for injection 3ml pre-filled pens (Sanofi) (product)
21953711000001100	Lyxumia 10micrograms/0.2ml solution for injection 3ml pre-filled pen and Lyxumia 20micrograms/0.2ml solution for injection 3ml pre-filled pen (Sanofi) (product)
21994611000001100	Lixisenatide 10micrograms/0.2ml solution for injection 3ml pre-filled disposable devices (product)
21994711000001100	Lixisenatide 10micrograms/0.2ml solution for injection 3ml pre-filled disposable devices and Lixisenatide 20micrograms/0.2ml solution for injection 3ml pre-filled disposable devices (product)
22026311000001100	Glibenclamide 5mg tablets (Waymade Healthcare Plc) (product)
22027111000001100	Glimepiride 1mg tablets (Waymade Healthcare Plc) (product)
22027411000001100	Glimepiride 2mg tablets (Waymade Healthcare Plc) (product)
22028211000001100	Glimepiride 4mg tablets (Waymade Healthcare Plc) (product)
22028411000001100	Glipizide 5mg tablets (Waymade Healthcare Plc) (product)
22105211000001100	Metformin 500mg/5ml oral solution sugar free (Zentiva) (product)
22225011000001100	Laaglyda MR 60 mg tablets (Consilient Health Ltd) (product)
22226111000001100	Gliclazide 60mg modified-release tablets (product)
22349811000001100	Metformin 500mg tablets (Aurobindo Pharma Ltd) (product)
22393411000001100	Metformin 500mg/5ml oral solution sugar free (Waymade Healthcare Plc) (product)
22879311000001100	Januvia 50mg tablets (Waymade Healthcare Plc) (product)
22949011000001100	Pioglitazone 15mg tablets (Ranbaxy (UK) Ltd) (product)
22949311000001100	Pioglitazone 30mg tablets (Ranbaxy (UK) Ltd) (product)
22949511000001100	Pioglitazone 45mg tablets (Ranbaxy (UK) Ltd) (product)
233411000001102	Euglucon 2.5mg tablets (Aventis Pharma) (product)
23372211000001100	Glidipion 45mg tablets (Actavis UK Ltd) (product)
234011000001108	Daonil 5mg tablets (Aventis Pharma) (product)
23487611000001100	Pioglitazone 30mg tablets (Accord Healthcare Ltd) (product)
23632611000001100	Vipdomet 12.5mg/1000mg tablets (Takeda UK Ltd) (product)
23634111000001100	Vipidia 6.25mg tablets (Takeda UK Ltd) (product)

23636011000001100	Vipidia 12.5mg tablets (Takeda UK Ltd) (product)
23636311000001100	Vipidia 25mg tablets (Takeda UK Ltd) (product)
23637211000001100	Alogliptin 12.5mg / Metformin 1g tablets (product)
23637411000001100	Alogliptin 25mg tablets (product)
23637511000001100	Alogliptin 6.25mg tablets (product)
23677111000001100	Januvia 25mg tablets (Waymade Healthcare Plc) (product)
23920511000001100	Gliclazide 30mg modified-release tablets (Phoenix Healthcare Distribution Ltd) (product)
23943411000001100	Glibenclamide 5mg tablets (Doncaster Pharmaceuticals Ltd) (product)
24054611000001100	Dapagliflozin 5mg / Metformin 1g tablets (product)
24104511000001100	Canagliflozin 300mg tablets (product)
24106311000001100	Glimepiride 1mg tablets (DE Pharmaceuticals) (product)
24106511000001100	Glimepiride 2mg tablets (DE Pharmaceuticals) (product)
24130611000001100	Acarbose 50mg tablets (Morningside Healthcare Ltd) (product)
24130811000001100	Acarbose 100mg tablets (Morningside Healthcare Ltd) (product)
24135111000001100	Pioglitazone 45mg tablets (Morningside Healthcare Ltd) (product)
24380011000001100	Metformin 500mg tablets (DE Pharmaceuticals) (product)
24380511000001100	Metformin 850mg tablets (DE Pharmaceuticals) (product)
24554311000001100	Metformin 500mg modified-release tablets (Actavis UK Ltd) (product)
24568211000001100	Sukkarto SR 1000mg tablets (Morningside Healthcare Ltd) (product)
25290611000001100	Empagliflozin 25mg tablets (product)
257011000001104	Metformin 500mg tablets (Generics (UK) Ltd) (product)
259611000001101	Amaryl 4mg tablets (Aventis Pharma) (product)
26209611000001100	NovoRapid PumpCart 100units/ml solution for injection 1.6ml cartridges (Novo Nordisk Ltd) (product)
26655811000001100	Insulin aspart 100units/ml solution for injection 1.6ml cartridges (product)
270611000001105	Avandia 8mg tablets (GlaxoSmithKline) (product)
27990411000001100	Forxiga 10mg tablets (Waymade Healthcare Plc) (product)
27990611000001100	Galvus 50mg tablets (Waymade Healthcare Plc) (product)
28044911000001100	Insulin human 500units/ml solution for injection 20ml vials (Special Order) (product)
28054311000001100	Xultophy 100units/ml / 3.6mg/ml solution for injection 3ml pre-filled pens (Novo Nordisk Ltd) (product)
28277811000001100	Victoza 6mg/ml solution for injection 3ml pre-filled pens (Waymade Healthcare Plc) (product)
28415811000001100	Metformin 500mg/5ml oral solution sugar free (Focus Pharmaceuticals Ltd) (product)
28420711000001100	Vamju 30mg modified-release tablets (AMCo) (product)
28426011000001100	Bydureon 2mg powder and solvent for prolonged-release suspension for injection pre-filled pens (AstraZeneca UK Ltd) (product)
28461011000001100	Trulicity 0.75mg/0.5ml solution for injection pre-filled pens (Eli Lilly and Company Ltd) (product)
28775211000001100	Canagliflozin 100mg tablets (Colorama Pharmaceuticals Ltd) (product)
28776011000001100	Liraglutide 6mg/ml solution for injection 3ml pre-filled disposable devices (Colorama Pharmaceuticals Ltd) (product)
28785111000001100	Metformin 1g oral powder sachets sugar free (J M McGill Ltd) (product)
28789611000001100	Dulaglutide 0.75mg/0.5ml solution for injection pre-filled disposable devices (product)
28989711000001100	Insulin lispro 200units/ml solution for injection 3ml pre-filled disposable devices (product)
29699911000001100	Canagliflozin 100mg tablets (J M McGill Ltd) (product)
29742811000001100	Diabiom 15mg tablets (Tillomed Laboratories Ltd) (product)
29754211000001100	Acarbose 100mg tablets (Sigma Pharmaceuticals Plc) (product)
29854711000001100	Liraglutide 6mg/ml solution for injection 3ml pre-filled disposable devices (Niche Pharma Ltd) (product)
29869311000001100	Glibenclamide 2.5mg tablets (Sigma Pharmaceuticals Plc) (product)
29869511000001100	Glibenclamide 5mg tablets (Sigma Pharmaceuticals Plc) (product)
29906411000001100	Metformin 850mg tablets (Sigma Pharmaceuticals Plc) (product)
29918411000001100	Metformin 500mg/5ml oral solution sugar free (Sigma Pharmaceuticals Plc) (product)
29960011000001100	Apidra 100units/ml solution for injection 3ml pre-filled SoloStar pens (Lexon (UK) Ltd) (product)
29985811000001100	Pioglitazone 15mg tablets (Sigma Pharmaceuticals Plc) (product)
29986011000001100	Pioglitazone 30mg tablets (Sigma Pharmaceuticals Plc) (product)
30012111000001100	Glucient SR 750mg tablets (Consilient Health Ltd) (product)
30012311000001100	Glucient SR 1000mg tablets (Consilient Health Ltd) (product)
30077211000001100	Pioglitazone 15mg tablets (DE Pharmaceuticals) (product)
30100711000001100	Tolbutamide 500mg tablets (DE Pharmaceuticals) (product)
30112111000001100	Pioglitazone 30mg tablets (DE Pharmaceuticals) (product)
30134511000001100	Glibenclamide 2.5mg tablets (Mawdsley-Brooks & Company Ltd) (product)
30134711000001100	Glibenclamide 5mg tablets (Mawdsley-Brooks & Company Ltd) (product)
30137811000001100	Glimepiride 2mg tablets (Mawdsley-Brooks & Company Ltd) (product)
30138311000001100	Glimepiride 3mg tablets (Mawdsley-Brooks & Company Ltd) (product)
30138511000001100	Glimepiride 4mg tablets (Mawdsley-Brooks & Company Ltd) (product)

30173411000001100	Synjardy 5mg/850mg tablets (Boehringer Ingelheim Ltd) (product)
30208411000001100	Eucreas 50mg/850mg tablets (Waymade Healthcare Plc) (product)
30214811000001100	Vipidia 25mg tablets (Waymade Healthcare Plc) (product)
30268411000001100	Apidra 100units/ml solution for injection 3ml pre-filled SoloStar pens (Waymade Healthcare Plc) (product)
30318111000001100	Empagliflozin 12.5mg / Metformin 1g tablets (product)
30829111000001100	Repaglinide 500microgram tablets (DE Pharmaceuticals) (product)
30857011000001100	Pioglitazone 15mg tablets (Mawdsley-Brooks & Company Ltd) (product)
30875411000001100	Repaglinide 500microgram tablets (Mawdsley-Brooks & Company Ltd) (product)
30875711000001100	Repaglinide 1mg tablets (Mawdsley-Brooks & Company Ltd) (product)
30876011000001100	Repaglinide 2mg tablets (Mawdsley-Brooks & Company Ltd) (product)
30928411000001100	Liraglutide 6mg/ml solution for injection 3ml pre-filled disposable devices (DE Pharmaceuticals) (product)
30983711000001100	Bilxona 60mg modified-release tablets (Actavis UK Ltd) (product)
31351911000001100	Alogliptin 12.5mg tablets (J M McGill Ltd) (product)
31352311000001100	Alogliptin 6.25mg tablets (J M McGill Ltd) (product)
322511000001103	Euglucon 5mg tablets (Aventis Pharma) (product)
32413911000001100	Gliclazide 80mg tablets (Genesis Pharmaceuticals Ltd) (product)
32431711000001100	Alogliptin 12.5mg / Metformin 1g tablets (Colorama Pharmaceuticals Ltd) (product)
32431911000001100	Alogliptin 12.5mg tablets (Colorama Pharmaceuticals Ltd) (product)
32492811000001100	Pioglitazone 15mg tablets (Brown & Burk UK Ltd) (product)
32493011000001100	Pioglitazone 30mg tablets (Brown & Burk UK Ltd) (product)
32493211000001100	Pioglitazone 45mg tablets (Brown & Burk UK Ltd) (product)
32493811000001100	Glimepiride 3mg tablets (Brown & Burk UK Ltd) (product)
325218001	Product containing precisely glibenclamide 2.5 milligram/1 each conventional release oral tablet (clinical drug)
325242002	Product containing precisely gliclazide 80 milligram/1 each conventional release oral tablet (clinical drug)
325243007	Product containing precisely glipizide 5 milligram/1 each conventional release oral tablet (clinical drug)
325248003	Product containing precisely glipizide 2.5 milligram/1 each conventional release oral tablet (clinical drug)
325262005	Product containing precisely glimepiride 3 milligram/1 each conventional release oral tablet (clinical drug)
325263000	Product containing precisely glimepiride 4 milligram/1 each conventional release oral tablet (clinical drug)
325279004	Product containing precisely metformin hydrochloride 850 milligram/1 each conventional release oral tablet (clinical drug)
3255911000001100	Humulin Isophane 100units/ml suspension for injection 10ml vials (Eli Lilly & Co Ltd) (product)
3258411000001100	Insuman Basal 100units/ml suspension for injection 5ml vials (Aventis Pharma) (product)
326037007	Product containing precisely acarbose 50 milligram/1 each conventional release oral tablet (clinical drug)
326048000	Product containing precisely repaglinide 1 milligram/1 each conventional release oral tablet (clinical drug)
326049008	Product containing precisely repaglinide 2 milligram/1 each conventional release oral tablet (clinical drug)
326056002	Product containing precisely rosiglitazone (as rosiglitazone maleate) 8 milligram/1 each conventional release oral tablet (clinical drug)
326057006	Product containing precisely rosiglitazone (as rosiglitazone maleate) 4 milligram/1 each conventional release oral tablet (clinical drug)
3260611000001100	Insuman Basal 100units/ml suspension for injection 3ml pre-filled OptiSet pens (Sanofi) (product)
326062007	Product containing precisely pioglitazone (as pioglitazone hydrochloride) 15 milligram/1 each conventional release oral tablet (clinical drug)
3262011000001100	Insulatard FlexPen 100units/ml suspension for injection (Novo Nordisk Pharmaceuticals Ltd) (product)
3262511000001100	Insulatard NovoLet 100units/ml suspension for injection (Novo Nordisk Pharmaceuticals Ltd) (product)
32625911000001100	Sitagliptin 50mg/5ml oral solution (Special Order) (product)
3263711000001100	Insulatard Penfill 100units/ml suspension for injection 3ml cartridges (Novo Nordisk Pharmaceuticals Ltd) (product)
3264111000001100	Insuman Comb 25 100units/ml suspension for injection 3ml cartridges (Aventis Pharma) (product)
3264711000001100	Mixtard 10 Penfill 100units/ml suspension for injection 3ml cartridges (Novo Nordisk Pharmaceuticals Ltd) (product)
3265011000001100	Insuman Basal 100units/ml suspension for injection 3ml cartridges (Aventis Pharma) (product)
3266111000001100	Mixtard 20 NovoLet 100units/ml suspension for injection (Novo Nordisk Pharmaceuticals Ltd) (product)
3266811000001100	Hypurin Porcine 30/70 Mix 100units/ml suspension for injection 10ml vials (C P Pharmaceuticals Ltd) (product)

3267811000001100	Human Mixtard 20 Penfill 100units/ml suspension for injection 1.5ml cartridges (Novo Nordisk Pharmaceuticals Ltd) (product)
3267911000001100	Pork Mixtard 30 100units/ml suspension for injection 10ml vials (Novo Nordisk Pharmaceuticals Ltd) (product)
3270511000001100	Mixtard 30 NovoLet 100units/ml suspension for injection (Novo Nordisk Pharmaceuticals Ltd) (product)
3271011000001100	Humulin M5 100units/ml suspension for injection 10ml vials (Eli Lilly & Co Ltd) (product)
3272011000001100	Mixtard 40 NovoLet 100units/ml suspension for injection (Novo Nordisk Pharmaceuticals Ltd) (product)
3272811000001100	Mixtard 50 Penfill 100units/ml suspension for injection 3ml cartridges (Novo Nordisk Pharmaceuticals Ltd) (product)
3273611000001100	Humulin M3 100units/ml suspension for injection 3ml cartridges (Eli Lilly & Co Ltd) (product)
3274511000001100	Insuman Comb 50 100units/ml suspension for injection 5ml vials (Aventis Pharma) (product)
3275011000001100	Mixtard 30 ge 100units/ml suspension for injection 10ml vials (Novo Nordisk Pharmaceuticals Ltd) (product)
3276911000001100	Humalog Mix50 Pen 100units/ml suspension for injection 3ml pre-filled pens (Eli Lilly and Company Ltd) (product)
3277211000001100	NovoMix 30 FlexPen 100units/ml suspension for injection 3ml pre-filled pens (Novo Nordisk Ltd) (product)
3277711000001100	Hypurin Porcine 30/70 Mix 100units/ml suspension for injection 1.5ml cartridges (C P Pharmaceuticals Ltd) (product)
3278311000001100	Mixtard 50 NovoLet 100units/ml suspension for injection (Novo Nordisk Pharmaceuticals Ltd) (product)
3278511000001100	Humalog Pen 100units/ml solution for injection 3ml pre-filled pens (Eli Lilly and Company Ltd) (product)
3280111000001100	Humalog 100units/ml solution for injection 10ml vials (Eli Lilly & Co Ltd) (product)
3281211000001100	Mixtard 40 Penfill 100units/ml suspension for injection 3ml cartridges (Novo Nordisk Pharmaceuticals Ltd) (product)
3281611000001100	NovoRapid Novolet 100units/ml solution for injection (Novo Nordisk Pharmaceuticals Ltd) (product)
3282711000001100	Humalog 100units/ml solution for injection 1.5ml cartridges (Eli Lilly & Co Ltd) (product)
3283211000001100	Lantus 100units/ml solution for injection 3ml pre-filled OptiSet pens (Sanofi) (product)
3283411000001100	Hypurin Porcine Neutral 100units/ml suspension for injection 1.5ml cartridges (C P Pharmaceuticals Ltd) (product)
3284911000001100	Humulin Lente 100units/ml suspension for injection 10ml vials (Eli Lilly & Co Ltd) (product)
3285011000001100	Hypurin Porcine Neutral 100units/ml solution for injection 10ml vials (C P Pharmaceuticals Ltd) (product)
32857511000001100	Sitagliptin 50mg/5ml oral suspension (Special Order) (product)
32879911000001100	Sitagliptin 50mg/5ml oral suspension (product)
3288511000001100	Actrapid NovoLet 100units/ml solution for injection (Novo Nordisk Pharmaceuticals Ltd) (product)
3290411000001100	HumaJect S Pen 100units/ml solution for injection (Eli Lilly & Co Ltd) (product)
3294911000001100	Insuman Rapid 100units/ml solution for injection 5ml vials (Aventis Pharma) (product)
3309511000001100	Human Actrapid Penfill 100units/ml solution for injection 1.5ml cartridges (Novo Nordisk Pharmaceuticals Ltd) (product)
3310711000001100	Actrapid Penfill 100units/ml solution for injection 3ml cartridges (Novo Nordisk Pharmaceuticals Ltd) (product)
3312611000001100	Humulin S 100units/ml solution for injection 10ml vials (Eli Lilly & Co Ltd) (product)
33547711000001100	Metformin 850mg/5ml oral solution sugar free (Colonis Pharma Ltd) (product)
33548911000001100	Metformin 500mg/5ml oral solution sugar free (Colonis Pharma Ltd) (product)
33549511000001100	Acarbose 50mg tablets (Mylan Ltd) (product)
33549711000001100	Acarbose 100mg tablets (Mylan Ltd) (product)
33550811000001100	Metformin 1g/5ml oral solution sugar free (product)
33599111000001100	Pioglitazone 15mg tablets (Mylan Ltd) (product)
33619611000001100	Repaglinide 2mg tablets (Mylan Ltd) (product)
3468611000001100	Insulin aspart 100units/ml solution for injection 3ml cartridges (product)
3469811000001100	Insulin isophane biphasic human 15/85 100units/ml suspension for injection 5ml vials (product)
3470111000001100	Insulin isophane biphasic human 20/80 100units/ml suspension for injection 3ml pre-filled disposable devices (product)
3470211000001100	Insulin isophane biphasic human 25/75 100units/ml suspension for injection 3ml cartridges (product)
3470711000001100	Insulin isophane biphasic human 30/70 100units/ml suspension for injection 10ml vials (product)
3471011000001100	Insulin isophane biphasic human 30/70 100units/ml suspension for injection 3ml pre-filled disposable devices (product)
3471311000001100	Insulin isophane biphasic human 40/60 100units/ml suspension for injection 3ml pre-filled disposable devices (product)

3471711000001100	Insulin isophane biphasic human 50/50 100units/ml suspension for injection 3ml pre-filled disposable devices (product)
3472011000001100	Insulin isophane biphasic porcine 30/70 100units/ml suspension for injection 10ml vials (product)
3472411000001100	Insulin isophane human 100units/ml suspension for injection 1.5ml cartridges (product)
3472511000001100	Insulin isophane human 100units/ml suspension for injection 10ml vials (product)
3472811000001100	Insulin isophane human 100units/ml suspension for injection 5ml vials (product)
3473011000001100	Insulin isophane porcine 100units/ml suspension for injection 10ml vials (product)
3474611000001100	Insulin soluble porcine 100units/ml suspension for injection 1.5ml cartridges (product)
363211000001102	Glucophage 500mg tablets (Merck Pharmaceuticals) (product)
365111000001109	Glucophage 850mg tablets (Merck Pharmaceuticals) (product)
3651211000001100	Starlix 120mg tablets (Novartis Pharmaceuticals UK Ltd) (product)
3763311000001100	Glurenorm 30mg tablets (Sanofi-Synthelabo Ltd) (product)
378911000001109	Gliclazide 80mg tablets (A A H Pharmaceuticals Ltd) (product)
386011000001102	Glibenclamide 2.5mg tablets (Generics (UK) Ltd) (product)
3883511000001100	Starlix 180mg tablets (Novartis Pharmaceuticals UK Ltd) (product)
393311000001107	Tolbutamide 500mg tablets (Unichem Plc) (product)
4029411000001100	Hypurin Porcine 30/70 Mix 100units/ml suspension for injection 3ml cartridges (C P Pharmaceuticals Ltd) (product)
4033311000001100	Insulin soluble bovine 100units/ml solution for injection 3ml cartridges (product)
4034911000001100	Hypurin Porcine Neutral 100units/ml suspension for injection 3ml cartridges (C P Pharmaceuticals Ltd) (product)
4053711000001100	Insulin soluble porcine 100units/ml suspension for injection 3ml cartridges (product)
409121008	Product containing precisely metformin hydrochloride 500 milligram and rosiglitazone (as rosiglitazone maleate) 1 milligram/1 each conventional release oral tablet (clinical drug)
409125004	Product containing precisely metformin hydrochloride 1 gram and rosiglitazone (as rosiglitazone maleate) 4 milligram/1 each conventional release oral tablet (clinical drug)
409361001	Metformin hydrochloride 750mg m/r tablet (product)
411533003	Metformin hydrochloride 1g m/r tablet (product)
419873003	Product containing precisely metformin hydrochloride 850 milligram and pioglitazone (as pioglitazone hydrochloride) 15 milligram/1 each conventional release oral tablet (clinical drug)
424345005	Product containing precisely sitagliptin (as sitagliptin phosphate) 100 milligram/1 each conventional release oral tablet (clinical drug)
443067000	Product containing precisely saxagliptin 2.5 milligram/1 each conventional release oral tablet (clinical drug)
443713000	Product containing precisely saxagliptin 5 milligram/1 each conventional release oral tablet (clinical drug)
4470711000001100	Tolbutamide 500mg tablets (Generics (UK) Ltd) (product)
464111000001100	Glipizide 5mg tablets (Generics (UK) Ltd) (product)
49111000001103	Diaglyk 80mg tablets (Ashbourne Pharmaceuticals Ltd) (product)
5268311000001100	Humalog 100units/ml solution for injection 3ml cartridges (PI) (Waymade Ltd) (product)
5268611000001100	Humalog Mix25 Pen 100units/ml suspension for injection 3ml pre-filled pens (Waymade Healthcare Plc) (product)
5269111000001100	Humalog Mix50 Pen 100units/ml suspension for injection 3ml pre-filled pens (Waymade Healthcare Plc) (product)
5270511000001100	NovoRapid Penfill 100units/ml solution for injection 3ml cartridges (PI) (Waymade Ltd) (product)
5295811000001100	Humalog 100units/ml solution for injection 1.5ml cartridges (PI) (Dowelhurst Ltd) (product)
5296011000001100	Humalog 100units/ml solution for injection 10ml vials (PI) (Dowelhurst Ltd) (product)
5302911000001100	Avandamet 1mg/500mg tablets (GlaxoSmithKline) (product)
5303611000001100	Avandamet 2mg/500mg tablets (GlaxoSmithKline) (product)
5330011000001100	Actos 30mg tablets (PI) (Waymade Ltd) (product)
5331011000001100	Amaryl 4mg tablets (PI) (Waymade Ltd) (product)
533211000001108	Gliclazide 80mg tablets (Generics (UK) Ltd) (product)
5336711000001100	Avandia 4mg tablets (PI) (Waymade Ltd) (product)
5355711000001100	Daonil 5mg tablets (PI) (Waymade Ltd) (product)
5369511000001100	Glibenese 5mg tablets (PI) (Waymade Ltd) (product)
5371611000001100	Glucobay 50 tablets (PI) (Waymade Ltd) (product)
5395711000001100	NovoNorm 500microgram tablets (PI) (Waymade Ltd) (product)
5396211000001100	NovoNorm 2mg tablets (PI) (Waymade Ltd) (product)
5415211000001100	Starlix 180mg tablets (PI) (Waymade Ltd) (product)
543011000001102	Glyformin 500mg tablets (Dr Reddy's Labs) (product)
5441111000001100	Acarbose 100mg tablets (PI) (Dowelhurst Ltd) (product)
5448611000001100	Glimepiride 1mg tablets (PI) (Dowelhurst Ltd) (product)
5481211000001100	Minodiab 5mg tablets (PI) (Waymade Ltd) (product)
5520811000001100	Amaryl 3mg tablets (PI) (Dowelhurst Ltd) (product)

622811000001108	Gliclazide 80mg tablets (C P Pharmaceuticals Ltd) (product)
639511000001103	Tolbutamide 500mg tablets (Approved Prescription Services) (product)
648011000001102	Glibenclamide 2.5mg tablets (A A H Pharmaceuticals Ltd) (product)
652411000001107	Minodiab 5mg tablets (Pfizer Ltd) (product)
656211000001109	Glibenclamide 2.5mg tablets (Unichem Plc) (product)
690811000001103	Metformin 500mg tablets (Kent Pharmaceuticals Ltd) (product)
695711000001101	Glibenclamide 5mg tablets (Alpharma Limited) (product)
697811000001104	Glipizide 5mg tablets (IVAX Pharmaceuticals UK Ltd) (product)
698511000001103	Glipizide 5mg tablets (Sandoz Ltd) (product)
7016011000001100	Metformin 500mg tablets (IVAX Pharmaceuticals UK Ltd) (product)
703682001	Product containing precisely canagliflozin 100 milligram/1 each conventional release oral tablet (clinical drug)
731211000001105	Glipizide 5mg tablets (A A H Pharmaceuticals Ltd) (product)
745211000001107	Glibenclamide 5mg tablets (A A H Pharmaceuticals Ltd) (product)
745911000001103	Diamicron 80mg tablets (Servier Laboratories Limited) (product)
7461611000001100	Metformin 500mg tablets (Ranbaxy (UK) Ltd) (product)
756411000001104	Glipizide 5mg tablets (Alpharma Limited) (product)
7589911000001100	Levemir Penfill 100units/ml solution for injection 3ml cartridges (Novo Nordisk Pharmaceuticals Ltd) (product)
7594211000001100	Insulin detemir 100units/ml solution for injection 3ml pre-filled disposable devices (product)
7597611000001100	Insulin detemir 100units/ml solution for injection 3ml cartridges (product)
788811000001108	Glipizide 5mg tablets (Unichem Plc) (product)
795311000001107	Metformin 850mg tablets (Generics (UK) Ltd) (product)
808711000001104	Minodiab 2.5mg tablets (Pfizer Ltd) (product)
8176311000001100	Avandamet 2mg/1000mg tablets (GlaxoSmithKline) (product)
822911000001107	Glibenclamide 5mg tablets (Kent Pharmaceuticals Ltd) (product)
8495311000001100	Glibenclamide 7.5mg/5ml oral solution (Special Order) (product)
8495611000001100	Glibenclamide 5mg/5ml oral suspension (Special Order) (product)
8495911000001100	Glibenclamide 7.5mg/5ml oral suspension (Special Order) (product)
8496611000001100	Gliclazide 160mg/5ml oral suspension (Special Order) (product)
8523711000001100	Glibenclamide 5mg/5ml oral solution (product)
8524011000001100	Glibenclamide 7.5mg/5ml oral suspension (product)
8524311000001100	Gliclazide 80mg/5ml oral suspension (product)
85711000001106	Metformin 850mg tablets (Unichem Plc) (product)
8614411000001100	Metformin 250mg/5ml oral solution (Special Order) (product)
8614811000001100	Metformin 425mg/5ml oral suspension (Special Order) (product)
8615611000001100	Metformin 250mg/5ml oral suspension (Special Order) (product)
8664211000001100	Metformin 425mg/5ml oral suspension (product)
868111000001101	Vivazide 80mg tablets (Lexon UK Ltd) (product)
882811000001109	Metformin 500mg tablets (Alpharma Limited) (product)
887211000001106	Glibenclamide 5mg tablets (Generics (UK) Ltd) (product)
903011000001101	Glibenclamide 5mg tablets (IVAX Pharmaceuticals UK Ltd) (product)
909311000001100	Metformin 850mg tablets (Sandoz Ltd) (product)
922211000001106	Avandia 4mg tablets (GlaxoSmithKline) (product)
929811000001102	Gliclazide 80mg tablets (Sandoz Ltd) (product)
9528811000001100	Apidra 100units/ml solution for injection 10ml vials (sanofi-aventis) (product)
9555911000001100	Metformin 500mg tablets (Wockhardt UK Ltd) (product)
9556111000001100	Metformin 850mg tablets (Wockhardt UK Ltd) (product)
9763311000001100	Metformin 850mg tablets (The Boots Company) (product)
9798211000001100	Metformin 850mg tablets (Almus Pharmaceutical Ltd) (product)
9801011000001100	Tolbutamide 500mg tablets (Almus Pharmaceutical Ltd) (product)
9801911000001100	Gliclazide 80mg tablets (Almus Pharmaceutical Ltd) (product)
34043411000001100	Fiasp 100units/ml solution for injection 10ml vials (Novo Nordisk Ltd) (product)
34188011000001100	Metformin 850mg/5ml oral solution sugar free (A A H Pharmaceuticals Ltd) (product)
34552811000001100	Meijumet 750mg modified-release tablets (Medreich Plc) (product)
34823611000001100	Gliclazide 40mg tablets (Teva UK Ltd) (product)
34956911000001100	Gliclazide 40mg tablets (Almus Pharmaceuticals Ltd) (product)
11119511000001100	Hypurin Bovine Isophane 100units/ml suspension for injection 10ml vials (Waymade Ltd) (product)
11119811000001100	Hypurin Bovine Isophane 100units/ml suspension for injection 1.5ml cartridges (Waymade Ltd) (product)
11982811000001100	Chlorpropamide 250mg/5ml oral suspension (Special Order) (product)
27692911000001100	Chlorpropamide 100mg tablets (Special Order) (product)
325213005	Product containing precisely chlorpropamide 100 milligram/1 each conventional release oral tablet (clinical drug)
325258004	Product containing precisely tolazamide 250 milligram/1 each conventional release oral tablet (clinical drug)
3279311000001100	Hypurin Bovine Isophane 100units/ml suspension for injection 1.5ml cartridges (C P Pharmaceuticals Ltd) (product)

3280011000001100	Hypurin Bovine Isophane 100units/ml suspension for injection 10ml vials (C P Pharmaceuticals Ltd) (product)
3472311000001100	Insulin isophane bovine 100units/ml suspension for injection 10ml vials (product)
374078008	Product containing precisely tolazamide 500 milligram/1 each conventional release oral tablet (clinical drug)
3949611000001100	Chlorpropamide 100mg tablets (The Boots Company) (product)
35214311000001100	Humulin R KwikPen 500units/ml solution for injection 3ml pre-filled pens (Imported (United States)) (product)
35215111000001100	Insulin human 500units/ml solution for injection 3ml pre-filled disposable devices (product)
30933011000001100	Alogliptin 12.5mg / Metformin 1g tablets (Ennogen Healthcare Ltd) (product)
30933211000001100	Alogliptin 12.5mg tablets (Ennogen Healthcare Ltd) (product)
30933611000001100	Alogliptin 6.25mg tablets (Ennogen Healthcare Ltd) (product)
32182811000001100	Alogliptin 25mg tablets (Niche Pharma Ltd) (product)
32183011000001100	Alogliptin 6.25mg tablets (Niche Pharma Ltd) (product)
35316811000001100	Gliclazide 40mg tablets (NorthStar Healthcare Unlimited Company) (product)
35547511000001100	Yaltormin SR 500mg tablets (Wockhardt UK Ltd) (product)
35548011000001100	Yaltormin SR 750mg tablets (Wockhardt UK Ltd) (product)
35563311000001100	Insulin lispro 100units/ml solution for injection 3ml cartridges (Sanofi Pasteur) (product)
35593311000001100	Metformin 500mg tablets (RX Farma) (product)
35593611000001100	Metformin 850mg tablets (RX Farma) (product)
35653211000001100	Pioglitazone 15mg / Metformin 850mg tablets (Teva UK Ltd) (product)
35672911000001100	Pioglitazone 15mg / Metformin 850mg tablets (A A H Pharmaceuticals Ltd) (product)
35776511000001100	Insulin lispro Sanofi 100units/ml solution for injection 3ml cartridges (Sanofi) (product)
35849011000001100	Metuxtan SR 500mg tablets (Accord Healthcare Ltd) (product)
36047011000001100	Insulin aspart 100units/ml solution for injection 10ml vials (product)
36047111000001100	Insulin glargine 100units/ml solution for injection 10ml vials (product)
36047211000001100	Insulin glulisine 100units/ml solution for injection 10ml vials (product)
36047711000001100	Insulin lispro 100units/ml solution for injection 3ml cartridges (product)
36047811000001100	Insulin lispro 100units/ml solution for injection 3ml pre-filled disposable devices (product)
36048011000001100	Insulin soluble bovine 100units/ml solution for injection 1.5ml cartridges (product)
36048211000001100	Insulin soluble human 100units/ml solution for injection 1.5ml cartridges (product)
36048311000001100	Insulin soluble human 100units/ml solution for injection 10ml vials (product)
36048711000001100	Insulin soluble porcine 100units/ml solution for injection 10ml vials (product)
36048911000001100	Insulin zinc mixed human 100units/ml suspension for injection 10ml vials (product)
36082811000001100	Semglee 100units/ml solution for injection 3ml pre-filled pens (Mylan) (product)
36468511000001100	Pioglitazone 15mg / Metformin 850mg tablets (Alliance Healthcare (Distribution) Ltd) (product)
36739111000001100	Gliclazide 40mg tablets (Flamingo Pharma (UK) Ltd) (product)
36763511000001100	Byetta 10micrograms/0.04ml solution for injection 2.4ml pre-filled pens (Originalis B.V.) (product)
36804511000001100	Jardiance 10mg tablets (Originalis B.V.) (product)
36832811000001100	Pioglitazone 15mg / Metformin 850mg tablets (Torrent Pharma (UK) Ltd) (product)
36856611000001100	Pioglitazone 15mg tablets (Torrent Pharma (UK) Ltd) (product)
36857111000001100	Pioglitazone 30mg tablets (Torrent Pharma (UK) Ltd) (product)
36893111000001100	Forxiga 10mg tablets (Pharmaram Ltd) (product)
36906311000001100	Vipidia 6.25mg tablets (Originalis B.V.) (product)
36911311000001100	Toujeo 300units/ml solution for injection 3ml pre-filled DoubleStar pens (Sanofi) (product)
36914911000001100	Gliclazide 160mg tablets (product)
36925911000001100	Gliclazide 80mg tablets (Relonchem Ltd) (product)
36931811000001100	Insulin glargine 300units/ml solution for injection 3ml pre-filled disposable devices (product)
37069611000001100	Glimepiride 1mg tablets (Mawdsley-Brooks & Company Ltd) (product)
37120411000001100	Metformin 500mg tablets (Mawdsley-Brooks & Company Ltd) (product)
36630511000001100	Insulin glargine 100units/ml / Lixisenatide 33micrograms/ml solution for injection 3ml pre-filled disposable devices (product)
36630611000001100	Insulin glargine 100units/ml / Lixisenatide 50micrograms/ml solution for injection 3ml pre-filled disposable devices (product)
37337011000001100	Amglidia 0.6mg/ml oral suspension with 1ml oral syringe (Amring Pharmaceuticals Ltd) (product)
37337211000001100	Amglidia 0.6mg/ml oral suspension with 5ml oral syringe (Amring Pharmaceuticals Ltd) (product)
37337711000001100	Amglidia 6mg/ml oral suspension with 5ml oral syringe (Amring Pharmaceuticals Ltd) (product)
37355411000001100	Byetta 10micrograms/0.04ml solution for injection 2.4ml pre-filled pens (Mawdsley-Brooks & Company Ltd) (product)
37387311000001100	Gliclazide 40mg tablets (DE Pharmaceuticals) (product)
37405911000001100	Glibenclamide 600micrograms/ml oral suspension sugar free (product)

37417011000001100	Humulin I 100units/ml suspension for injection 3ml cartridges (CST Pharma Ltd) (product)
37417211000001100	Humulin M3 100units/ml suspension for injection 3ml cartridges (CST Pharma Ltd) (product)
37420411000001100	Humulin I 100units/ml suspension for injection 10ml vials (CST Pharma Ltd) (product)
37428011000001100	Actos 15mg tablets (CST Pharma Ltd) (product)
37428411000001100	Actos 45mg tablets (CST Pharma Ltd) (product)
37428811000001100	Apidra 100units/ml solution for injection 3ml pre-filled SoloStar pens (CST Pharma Ltd) (product)
37438411000001100	Galvus 50mg tablets (CST Pharma Ltd) (product)
37439111000001100	Humalog KwikPen 100units/ml solution for injection 3ml pre-filled pens (CST Pharma Ltd) (product)
37440411000001100	Januvia 25mg tablets (CST Pharma Ltd) (product)
37440811000001100	Jardiance 10mg tablets (CST Pharma Ltd) (product)
37441111000001100	Jardiance 25mg tablets (CST Pharma Ltd) (product)
37508611000001100	Glucophage SR 750mg tablets (Mawdsley-Brooks & Company Ltd) (product)
37508911000001100	Glucophage 500mg tablets (Mawdsley-Brooks & Company Ltd) (product)
37512111000001100	Apidra 100units/ml solution for injection 3ml pre-filled SoloStar pens (Mawdsley-Brooks & Company Ltd) (product)
37512611000001100	Invokana 100mg tablets (Mawdsley-Brooks & Company Ltd) (product)
37525711000001100	Januvia 50mg tablets (Mawdsley-Brooks & Company Ltd) (product)
37527211000001100	Onglyza 2.5mg tablets (CST Pharma Ltd) (product)
37527411000001100	Onglyza 5mg tablets (CST Pharma Ltd) (product)
37537911000001100	Vipidia 12.5mg tablets (CST Pharma Ltd) (product)
37538511000001100	Xigduo 5mg/1000mg tablets (CST Pharma Ltd) (product)
37550411000001100	Humalog Mix50 KwikPen 100units/ml suspension for injection 3ml pre-filled pens (CST Pharma Ltd) (product)
37550911000001100	Humalog Mix25 100units/ml suspension for injection 3ml cartridges (CST Pharma Ltd) (product)
37665511000001100	Forxiga 10mg tablets (Pilsco Ltd) (product)
37666211000001100	Humalog Mix25 100units/ml suspension for injection 3ml cartridges (Pilsco Ltd) (product)
37667011000001100	Invokana 100mg tablets (Pilsco Ltd) (product)
37667311000001100	Janumet 50mg/1000mg tablets (Pilsco Ltd) (product)
37667611000001100	Januvia 100mg tablets (Pilsco Ltd) (product)
37668011000001100	Januvia 25mg tablets (Pilsco Ltd) (product)
37668211000001100	Januvia 50mg tablets (Pilsco Ltd) (product)
37668511000001100	Jardiance 10mg tablets (Pilsco Ltd) (product)
37672211000001100	Lantus 100units/ml solution for injection 3ml cartridges (Pilsco Ltd) (product)
37695011000001100	NovoMix 30 Penfill 100units/ml suspension for injection 3ml cartridges (Pilsco Ltd) (product)
37696411000001100	Onglyza 5mg tablets (Pilsco Ltd) (product)
37708911000001100	Victoza 6mg/ml solution for injection 3ml pre-filled pens (Pilsco Ltd) (product)
37709511000001100	Vipidia 25mg tablets (Pilsco Ltd) (product)
37719711000001100	Humulin M3 100units/ml suspension for injection 10ml vials (Pilsco Ltd) (product)
37747911000001100	Jentadueto 2.5mg/1000mg tablets (CST Pharma Ltd) (product)
37751111000001100	Tresiba FlexTouch 200units/ml solution for injection 3ml pre-filled pens (CST Pharma Ltd) (product)
37775311000001100	Pioglitazone 15mg / Metformin 850mg tablets (DE Pharmaceuticals) (product)
10925511000001100	Metformin 850mg / Pioglitazone 15mg tablets (product)
11473511000001100	Sitagliptin 100mg tablets (product)
16037911000001100	Saxagliptin 5mg tablets (product)
325063005	Insulin soluble human 100units/mL injection solution 3mL cartridge (product)
325064004	Insulin soluble human 100units/mL injection solution 10mL vial (product)
325067006	Insulin lispro 100units/mL injection solution 3mL cartridge (product)
325070005	Insulin lispro 100units/mL injection solution 3mL prefilled pen (product)
325076004	Insulin aspart 100units/mL injection solution 10mL vial (product)
325111000	Insulin isophane human 100units/mL injection suspension 3mL cartridge (product)
3469111000001100	Insulin glargine 100units/ml solution for injection 3ml cartridges (product)
353987005	Insulin soluble human 100units/mL injection solution 3mL prefilled pen (product)
353988000	Insulin isophane human 100units/mL injection suspension 3mL prefilled pen (product)
371418007	Insulin soluble bovine 100u/mL injection solution 10mL vial (product)
371419004	Insulin soluble bovine 100u/mL injection solution 1.5mL cartridge (product)
371430001	Insulin soluble porcine 100u/mL injection solution 10mL vial (product)
371483009	Insulin soluble human 100u/mL injection solution 5mL vial (product)
371496005	Insulin lispro 100u/mL injection solution 1.5mL cartridge (product)
37853211000001100	Synjardy 12.5mg/1000mg tablets (CST Pharma Ltd) (product)
412448008	Insulin glulisine 100units/mL injection 10mL vial (product)
5322611000001100	Metformin 500mg / Rosiglitazone 1mg tablets (product)
8175511000001100	Metformin 1g / Rosiglitazone 4mg tablets (product)
8285611000001100	Metformin 1g / Rosiglitazone 2mg tablets (product)
37972011000001100	Humalog 100units/ml solution for injection 10ml vials (Pharmaram Ltd) (product)
37972211000001100	Humalog Mix25 100units/ml suspension for injection 3ml cartridges (Pharmaram Ltd) (product)

37973311000001100	NovoRapid Penfill 100units/ml solution for injection 3ml cartridges (Pharmaram Ltd) (product)
37974511000001100	Vipidia 12.5mg tablets (Pharmaram Ltd) (product)
37989611000001100	Invokana 100mg tablets (Pharmaram Ltd) (product)
37989911000001100	Janumet 50mg/1000mg tablets (Pharmaram Ltd) (product)
37990211000001100	Januvia 100mg tablets (Pharmaram Ltd) (product)
38060511000001100	Bydureon BCise 2mg/0.85ml prolonged-release suspension for injection pre-filled pens (AstraZeneca UK Ltd) (product)
38067111000001100	Insulin soluble human 1unit/ml solution for infusion 30ml pre-filled syringes (Special Order) (product)
38067711000001100	Insulin soluble human 1unit/ml solution for infusion 30ml pre-filled syringes (product)
38116611000001100	Apidra 100units/ml solution for injection 3ml pre-filled SoloStar pens (DE Pharmaceuticals) (product)
38123611000001100	Byetta 10micrograms/0.04ml solution for injection 2.4ml pre-filled pens (DE Pharmaceuticals) (product)
38135711000001100	Forxiga 10mg tablets (DE Pharmaceuticals) (product)
38138111000001100	Glucophage SR 750mg tablets (DE Pharmaceuticals) (product)
38138411000001100	Glucophage SR 1000mg tablets (DE Pharmaceuticals) (product)
38139711000001100	Humulin M3 100units/ml suspension for injection 3ml cartridges (DE Pharmaceuticals) (product)
38140911000001100	Invokana 100mg tablets (DE Pharmaceuticals) (product)
38141511000001100	Janumet 50mg/1000mg tablets (DE Pharmaceuticals) (product)
38141911000001100	Januvia 50mg tablets (DE Pharmaceuticals) (product)
38143211000001100	Jentadueto 2.5mg/1000mg tablets (DE Pharmaceuticals) (product)
38157811000001100	Onglyza 2.5mg tablets (DE Pharmaceuticals) (product)
38170511000001100	Toujeo 300units/ml solution for injection 1.5ml pre-filled SoloStar pens (DE Pharmaceuticals) (product)
38170711000001100	Trajenta 5mg tablets (DE Pharmaceuticals) (product)
38171211000001100	Tresiba FlexTouch 200units/ml solution for injection 3ml pre-filled pens (DE Pharmaceuticals) (product)
38174811000001100	Vipidia 6.25mg tablets (DE Pharmaceuticals) (product)
38175011000001100	Vipidia 12.5mg tablets (DE Pharmaceuticals) (product)
38176411000001100	Xigduo 5mg/1000mg tablets (DE Pharmaceuticals) (product)
38238111000001100	Sukkarto SR 750mg tablets (Morningside Healthcare Ltd) (product)
38292411000001100	Acarbose 50mg tablets (Rivopharm (UK) Ltd) (product)
38379411000001100	Saxenda 6mg/ml solution for injection 3ml pre-filled pens (CST Pharma Ltd) (product)
38533711000001100	Metformin 750mg modified-release tablets (Morningside Healthcare Ltd) (product)
10093311000001100	Apidra 100units/ml solution for injection 3ml pre-filled OptiSet pens (Sanofi) (product)
10097211000001100	Insulin glulisine 100units/ml solution for injection 3ml pre-filled disposable devices (product)
10250211000001100	Lantus OptiClik 100units/ml solution for injection 3ml cartridges (sanofi-aventis) (product)
10272911000001100	Glimepiride 4mg tablets (Unichem Plc) (product)
10274011000001100	Glimepiride 2mg tablets (A A H Pharmaceuticals Ltd) (product)
10292611000001100	Metformin 500mg tablets (Relonchem Ltd) (product)
10344911000001100	Humalog Mix50 100units/ml suspension for injection 3ml cartridges (Eli Lilly & Co Ltd) (product)
10352711000001100	Insulin lispro biphasic 50/50 100units/ml suspension for injection 3ml cartridges (product)
10445111000001100	Glimepiride 1mg tablets (Niche Generics Ltd) (product)
10445311000001100	Glimepiride 2mg tablets (Niche Generics Ltd) (product)
10445511000001100	Glimepiride 3mg tablets (Niche Generics Ltd) (product)
10459011000001100	Actos 30mg tablets (PI) (Dowelhurst Ltd) (product)
10464711000001100	Avandia 4mg tablets (PI) (Dowelhurst Ltd) (product)
10470611000001100	Avandamet 2mg/500mg tablets (PI) (Dowelhurst Ltd) (product)
10486711000001100	Avandamet 1mg/500mg tablets (PI) (Waymade Ltd) (product)
10486811000001100	NovoNorm 1mg tablets (PI) (Dowelhurst Ltd) (product)
10487411000001100	Avandamet 2mg/1000mg tablets (PI) (Waymade Ltd) (product)
10487711000001100	Avandamet 2mg/500mg tablets (PI) (Waymade Ltd) (product)
10508511000001100	Glibenese 5mg tablets (PI) (Dowelhurst Ltd) (product)
10517911000001100	Glimepiride 2mg tablets (Winthrop Pharmaceuticals UK Ltd) (product)
10529511000001100	Diamicron 30mg MR tablets (PI) (Waymade Ltd) (product)
10534811000001100	NovoRapid 100units/ml solution for injection 10ml vials (PI) (Waymade Ltd) (product)
10542211000001100	Humalog Pen 100units/ml solution for injection 3ml pre-filled pens (Waymade Healthcare Plc) (product)
10542911000001100	NovoMix 30 Penfill 100units/ml suspension for injection 3ml cartridges (PI) (Waymade Ltd) (product)
10617311000001100	Glimepiride 1mg tablets (Somex Pharma) (product)
10617811000001100	Glimepiride 3mg tablets (Somex Pharma) (product)
10618111000001100	Glimepiride 4mg tablets (Somex Pharma) (product)
10688811000001100	Glimepiride 1mg tablets (Pliva Pharma Ltd) (product)
10690511000001100	Exubera 1mg inhalation powder blisters (Pfizer Ltd) (product)
10690811000001100	Exubera 3mg inhalation powder blisters (Pfizer Ltd) (product)
10703011000001100	Insulin human 1mg inhalation powder blisters (product)

10703111000001100	Insulin human 3mg inhalation powder blisters (product)
107311000001107	Glibenclamide 2.5mg tablets (Kent Pharmaceuticals Ltd) (product)
10952411000001100	Prandin 0.5mg tablets (Novo Nordisk Ltd) (product)
10957811000001100	Prandin 2mg tablets (Novo Nordisk Ltd) (product)
11018611000001100	Glimepiride 3mg tablets (Dr Reddy's Labs) (product)
11113611000001100	Pork Actrapid 100units/ml solution for injection 10ml vials (Waymade Ltd) (product)
11114411000001100	Pork Insulatard 100units/ml suspension for injection 10ml vials (Waymade Ltd) (product)
11115011000001100	Levemir FlexPen 100units/ml solution for injection 3ml pre-filled pens (Waymade Healthcare Plc) (product)
11116811000001100	Hypurin Porcine Neutral 100units/ml solution for injection 10ml vials (Waymade Ltd) (product)
11117211000001100	Hypurin Porcine Neutral 100units/ml solution for injection 1.5ml cartridges (Waymade Ltd) (product)
11118411000001100	Hypurin Porcine Isophane 100units/ml suspension for injection 10ml vials (Waymade Ltd) (product)
11119311000001100	Hypurin Bovine Lente 100units/ml suspension for injection 10ml vials (Waymade Ltd) (product)
11122611000001100	Mixtard 50 Penfill 100units/ml suspension for injection 3ml cartridges (Waymade Ltd) (product)
11123011000001100	Human Mixtard 50 Penfill 100units/ml suspension for injection 1.5ml cartridges (Waymade Ltd) (product)
11123311000001100	Mixtard 40 Penfill 100units/ml suspension for injection 3ml cartridges (Waymade Ltd) (product)
11123611000001100	Human Mixtard 40 Penfill 100units/ml suspension for injection 1.5ml cartridges (Waymade Ltd) (product)
11124211000001100	Mixtard 30 Penfill 100units/ml suspension for injection 3ml cartridges (Waymade Ltd) (product)
11124611000001100	Mixtard 20 Penfill 100units/ml suspension for injection 3ml cartridges (Waymade Ltd) (product)
11132611000001100	Actrapid 100units/ml solution for injection 10ml vials (Waymade Ltd) (product)
11133811000001100	Insulatard InnoLet 100units/ml suspension for injection 3ml pre-filled pens (Waymade Healthcare Plc) (product)
11134011000001100	Insulatard Penfill 100units/ml suspension for injection 3ml cartridges (Waymade Ltd) (product)
11135211000001100	Human Mixtard 10 Penfill 100units/ml suspension for injection 1.5ml cartridges (Waymade Ltd) (product)
11135411000001100	Human Mixtard 20 Penfill 100units/ml suspension for injection 1.5ml cartridges (Waymade Ltd) (product)
11149811000001100	Glimepiride 4mg tablets (Teva UK Ltd) (product)
11343211000001100	Glimepiride 2mg tablets (Sandoz Ltd) (product)
11344011000001100	Glimepiride 4mg tablets (Sandoz Ltd) (product)
11411000001101	Gliclazide 80mg tablets (Kent Pharmaceuticals Ltd) (product)
11494111000001100	Exenatide 10micrograms/0.04ml solution for injection 2.4ml pre-filled disposable devices (product)
11494211000001100	Exenatide 5micrograms/0.02ml solution for injection 1.2ml pre-filled disposable devices (product)
115811000001106	Metformin 850mg tablets (Alpharma Limited) (product)
11599411000001100	Metformin 500mg/5ml oral solution sugar free (Rosemont Pharmaceuticals Ltd) (product)
11765811000001100	Prandin 0.5mg tablets (Waymade Healthcare Plc) (product)
120811000001108	Glibenclamide 5mg tablets (C P Pharmaceuticals Ltd) (product)
12107211000001100	Glibenclamide 625micrograms/5ml oral suspension (Special Order) (product)
12107811000001100	Glibenclamide 15mg/5ml oral suspension (Special Order) (product)
12108111000001100	Glibenclamide 15mg/5ml oral solution (Special Order) (product)
12109011000001100	Gliclazide 100mg/5ml oral suspension (Special Order) (product)
12109311000001100	Gliclazide 120mg/5ml oral suspension (Special Order) (product)
12109611000001100	Gliclazide 20mg/5ml oral suspension (Special Order) (product)
12135311000001100	Glibenclamide 15mg/5ml oral solution (product)
12135711000001100	Glibenclamide 625micrograms/5ml oral solution (product)
12136011000001100	Gliclazide 120mg/5ml oral suspension (product)
12136411000001100	Gliclazide 60mg/5ml oral suspension (product)
12147111000001100	Glimepiride 1mg tablets (Kent Pharmaceuticals Ltd) (product)
12147511000001100	Glimepiride 3mg tablets (Kent Pharmaceuticals Ltd) (product)
12147711000001100	Glimepiride 4mg tablets (Kent Pharmaceuticals Ltd) (product)
12792711000001100	Metformin 1g/5ml oral solution (Special Order) (product)
12793011000001100	Metformin 5mg/5ml oral solution (Special Order) (product)
12813711000001100	Metformin 5mg/5ml oral solution (product)
129411000001101	Glipizide 5mg tablets (Pfizer Ltd) (product)
13100411000001100	Avandamet 2mg/1000mg tablets (Dowelhurst Ltd) (product)
13158411000001100	Diamicron 30mg MR tablets (Dowelhurst Ltd) (product)
13168311000001100	Januvia 100mg tablets (Dowelhurst Ltd) (product)
13180011000001100	NovoMix 30 FlexPen 100units/ml suspension for injection 3ml pre-filled pens (Dowelhurst Ltd) (product)
13181211000001100	NovoRapid FlexPen 100units/ml solution for injection 3ml pre-filled pens (Dowelhurst Ltd) (product)

13182011000001100	NovoRapid Penfill 100units/ml solution for injection 3ml cartridges (Dowelhurst Ltd) (product)
13205311000001100	Starlix 120mg tablets (Dowelhurst Ltd) (product)
13317511000001100	Tolbutamide 1g/5ml oral suspension (Special Order) (product)
13324311000001100	Tolbutamide 1g/5ml oral suspension (product)
13324411000001100	Tolbutamide 250mg/5ml oral suspension (product)
13412311000001100	Eucreas 50mg/1000mg tablets (Novartis Pharmaceuticals UK Ltd) (product)
13412611000001100	Eucreas 50mg/850mg tablets (Novartis Pharmaceuticals UK Ltd) (product)
13412911000001100	Vildagliptin 50mg tablets (product)
13413011000001100	Metformin 1g / Vildagliptin 50mg tablets (product)
13435011000001100	Pioglitazone 30mg/5ml oral suspension (Special Order) (product)
13440011000001100	Pioglitazone 30mg/5ml oral suspension (product)
134609007	Product containing precisely nateglinide 120 milligram/1 each conventional release oral tablet (clinical drug)
134610002	Product containing precisely nateglinide 60 milligram/1 each conventional release oral tablet (clinical drug)
13578511000001100	Glipizide 5mg tablets (Doncaster Pharmaceuticals Ltd) (product)
13588911000001100	Gliclazide 30mg modified-release tablets (UniChem Ltd) (product)
13626511000001100	Niddaryl 2mg tablets (Dee Pharmaceuticals Ltd) (product)
13626911000001100	Niddaryl 3mg tablets (Dee Pharmaceuticals Ltd) (product)
13720511000001100	Glimepiride 2mg tablets (Ranbaxy (UK) Ltd) (product)
13721211000001100	Glimepiride 4mg tablets (Ranbaxy (UK) Ltd) (product)
13757811000001100	Metformin 850mg tablets (Tillomed Laboratories Ltd) (product)
13772811000001100	Humulin I 100units/ml suspension for injection 3ml cartridges (Waymade Healthcare Plc) (product)
13773011000001100	Humulin S 100units/ml solution for injection 3ml cartridges (Waymade Healthcare Plc) (product)
13833111000001100	Avandamet 2mg/500mg tablets (Doncaster Pharmaceuticals Ltd) (product)
13833811000001100	Avandia 4mg tablets (Doncaster Pharmaceuticals Ltd) (product)
13834011000001100	Avandia 8mg tablets (Doncaster Pharmaceuticals Ltd) (product)
13850511000001100	Humulin R 100units/ml solution for injection 10ml vials (Imported (United States)) (product)
13883511000001100	Glucophage 500mg tablets (Doncaster Pharmaceuticals Ltd) (product)
13883911000001100	Glucophage 850mg tablets (Doncaster Pharmaceuticals Ltd) (product)
13884711000001100	Humalog Mix25 KwikPen 100units/ml suspension for injection 3ml pre-filled pens (Eli Lilly and Company Ltd) (product)
14040411000001100	Actos 30mg tablets (Sigma Pharmaceuticals Plc) (product)
14056511000001100	Amaryl 2mg tablets (Sigma Pharmaceuticals Plc) (product)
140711000001103	Semi-Daonil 2.5mg tablets (Aventis Pharma) (product)
14122311000001100	Glimepiride 2mg tablets (Consilient Health Ltd) (product)
14122711000001100	Glimepiride 4mg tablets (Consilient Health Ltd) (product)
14183911000001100	Bolamyn SR 500mg tablets (Teva UK Ltd) (product)
14199911000001100	Avandamet 2mg/1000mg tablets (Sigma Pharmaceuticals Plc) (product)
14200111000001100	Avandamet 4mg/1000mg tablets (Sigma Pharmaceuticals Plc) (product)
14248411000001100	Humalog Mix50 100units/ml suspension for injection 3ml cartridges (Sigma Pharmaceuticals Plc) (product)
14248611000001100	Humalog Mix25 Pen 100units/ml suspension for injection 3ml pre-filled pens (Sigma Pharmaceuticals Plc) (product)
14249011000001100	Humalog Pen 100units/ml solution for injection 3ml pre-filled pens (Sigma Pharmaceuticals Plc) (product)
14249211000001100	Humalog 100units/ml solution for injection 10ml vials (Sigma Pharmaceuticals Plc) (product)
14313211000001100	Diamicron 30mg MR tablets (Sigma Pharmaceuticals Plc) (product)
14400811000001100	Starlix 120mg tablets (Sigma Pharmaceuticals Plc) (product)
14401211000001100	Starlix 180mg tablets (Sigma Pharmaceuticals Plc) (product)
14584711000001100	Metformin 500mg modified-release tablets (A A H Pharmaceuticals Ltd) (product)
14592411000001100	Humulin I 100units/ml suspension for injection 10ml vials (Waymade Healthcare Plc) (product)
14665711000001100	NovoRapid 100units/ml solution for injection 10ml vials (Sigma Pharmaceuticals Plc) (product)
14711111000001100	Minodiab 5mg tablets (Sigma Pharmaceuticals Plc) (product)
14759411000001100	Lantus 100units/ml solution for injection 3ml cartridges (Sigma Pharmaceuticals Plc) (product)
14773811000001100	Metformin 500mg tablets (LPC Medical (UK) Ltd) (product)
15090711000001100	Gliclazide 30mg modified-release tablets (Sigma Pharmaceuticals Plc) (product)
15091211000001100	Glimepiride 2mg tablets (Sigma Pharmaceuticals Plc) (product)
15091611000001100	Glimepiride 4mg tablets (Sigma Pharmaceuticals Plc) (product)
15334511000001100	Edicil MR 30mg tablets (Ratiopharm UK Ltd) (product)
15359011000001100	Competact 15mg/850mg tablets (Waymade Healthcare Plc) (product)
15367811000001100	Glucophage SR 1000mg tablets (Merck Serono Ltd) (product)
15373711000001100	Glucophage 500mg oral powder sachets (Merck Serono Ltd) (product)
15411311000001100	Metformin 500mg oral powder sachets sugar free (product)
15611411000001100	Insulin human 500units/ml solution for injection 20ml vials (product)
157911000001103	Metformin 850mg tablets (Approved Prescription Services) (product)
15859111000001100	Liraglutide 18mg/3ml solution for injection pre-filled disposable devices (product)
15973311000001100	Metformin 500mg tablets (Zanza Laboratories Ltd) (product)
15984111000001100	Metformin 500mg/5ml oral solution sugar free (Actavis UK Ltd) (product)

15993011000001100	Onglyza 5mg tablets (Bristol-Myers Squibb Pharmaceuticals Ltd) (product)
16007611000001100	Pioglitazone 15mg/5ml oral suspension (Special Order) (product)
16036811000001100	Pioglitazone 15mg/5ml oral suspension (product)
16053211000001100	Gliclazide 80mg tablets (Bristol Laboratories Ltd) (product)
16053511000001100	Glimepiride 1mg tablets (Bristol Laboratories Ltd) (product)
16053911000001100	Glimepiride 3mg tablets (Bristol Laboratories Ltd) (product)
16061711000001100	Metformin 850mg tablets (Bristol Laboratories Ltd) (product)
16125711000001100	Glimepiride 2mg/5ml oral suspension (Special Order) (product)
16137611000001100	Amaryl 3mg tablets (Lexon (UK) Ltd) (product)
16137811000001100	Amaryl 4mg tablets (Lexon (UK) Ltd) (product)
16158211000001100	Diamicron 30mg MR tablets (Lexon (UK) Ltd) (product)
16187611000001100	Glucophage 850mg tablets (Lexon (UK) Ltd) (product)
16256011000001100	Actos 15mg tablets (Mawdsley-Brooks & Company Ltd) (product)
16256611000001100	Diamicron 30mg MR tablets (Mawdsley-Brooks & Company Ltd) (product)
16271111000001100	Avandamet 2mg/500mg tablets (Mawdsley-Brooks & Company Ltd) (product)
16272111000001100	Avandamet 4mg/1000mg tablets (Mawdsley-Brooks & Company Ltd) (product)
16273111000001100	Avandia 8mg tablets (Mawdsley-Brooks & Company Ltd) (product)
164511000001108	Amaryl 3mg tablets (Aventis Pharma) (product)
16503111000001100	Glucophage SR 500mg tablets (Mawdsley-Brooks & Company Ltd) (product)
16516311000001100	Humalog 100units/ml solution for injection 3ml cartridges (Mawdsley-Brooks & Company Ltd) (product)
16518411000001100	Humalog Mix25 Pen 100units/ml suspension for injection 3ml pre-filled pens (Mawdsley-Brooks & Company Ltd) (product)
16519811000001100	Mixtard 30 Penfill 100units/ml suspension for injection 3ml cartridges (Mawdsley-Brooks & Company Ltd) (product)
16520511000001100	Humulin M3 100units/ml suspension for injection 10ml vials (Mawdsley-Brooks & Company Ltd) (product)
16521911000001100	Humulin S 100units/ml solution for injection 10ml vials (Mawdsley-Brooks & Company Ltd) (product)
16522311000001100	NovoRapid FlexPen 100units/ml solution for injection 3ml pre-filled pens (Mawdsley-Brooks & Company Ltd) (product)
16522711000001100	NovoRapid Penfill 100units/ml solution for injection 3ml cartridges (Mawdsley-Brooks & Company Ltd) (product)
16701411000001100	Metformin 850mg tablets (Pfizer Ltd) (product)
16702011000001100	Gliclazide 40mg tablets (product)
16727911000001100	Levemir InnoLet 100units/ml solution for injection 3ml pre-filled pens (Waymade Healthcare Plc) (product)
17199311000001100	Gliclazide 80mg tablets (Sovereign Medical Ltd) (product)
17205711000001100	Tolbutamide 500mg tablets (Sovereign Medical Ltd) (product)
17496211000001100	Byetta 5micrograms/0.02ml solution for injection 1.2ml pre-filled pens (Mawdsley-Brooks & Company Ltd) (product)
17503511000001100	Eucreas 50mg/1000mg tablets (Mawdsley-Brooks & Company Ltd) (product)
17503711000001100	Eucreas 50mg/850mg tablets (Mawdsley-Brooks & Company Ltd) (product)
17506511000001100	Galvus 50mg tablets (Mawdsley-Brooks & Company Ltd) (product)
17509911000001100	Apidra 100units/ml solution for injection 3ml cartridges (Mawdsley-Brooks & Company Ltd) (product)
17510911000001100	Levemir Penfill 100units/ml solution for injection 3ml cartridges (Mawdsley-Brooks & Company Ltd) (product)
17511111000001100	Levemir FlexPen 100units/ml solution for injection 3ml pre-filled pens (Mawdsley-Brooks & Company Ltd) (product)
17558911000001100	Glucobay 100mg tablets (Sigma Pharmaceuticals Plc) (product)
17559111000001100	Humulin I 100units/ml suspension for injection 3ml cartridges (Sigma Pharmaceuticals Plc) (product)
17567211000001100	Januvia 100mg tablets (Sigma Pharmaceuticals Plc) (product)
17581511000001100	Amaryl 1mg tablets (Necessity Supplies Ltd) (product)
17582111000001100	Amaryl 2mg tablets (Necessity Supplies Ltd) (product)
17582711000001100	Amaryl 4mg tablets (Necessity Supplies Ltd) (product)
17592711000001100	Avandamet 2mg/1000mg tablets (Necessity Supplies Ltd) (product)
17593011000001100	Avandamet 2mg/500mg tablets (Necessity Supplies Ltd) (product)
17593811000001100	Avandamet 4mg/1000mg tablets (Necessity Supplies Ltd) (product)
17594311000001100	Avandia 4mg tablets (Necessity Supplies Ltd) (product)
17601411000001100	NovoMix 30 FlexPen 100units/ml suspension for injection 3ml pre-filled pens (Necessity Supplies Ltd) (product)
17601611000001100	NovoMix 30 Penfill 100units/ml suspension for injection 3ml cartridges (Necessity Supplies Ltd) (product)
17601911000001100	NovoRapid FlexPen 100units/ml solution for injection 3ml pre-filled pens (Necessity Supplies Ltd) (product)
17609511000001100	Humulin M3 KwikPen 100units/ml suspension for injection 3ml pre-filled pens (Eli Lilly and Company Ltd) (product)
17621611000001100	Prandin 0.5mg tablets (Necessity Supplies Ltd) (product)
17821311000001100	Tolbutamide 500mg tablets (Phoenix Healthcare Distribution Ltd) (product)
17880511000001100	Gliclazide 40mg tablets (A A H Pharmaceuticals Ltd) (product)
17899911000001100	Humulin M3 100units/ml suspension for injection 3ml cartridges (Sigma Pharmaceuticals Plc) (product)
17900111000001100	Onglyza 5mg tablets (Sigma Pharmaceuticals Plc) (product)
17914711000001100	Glibenclamide 2.5mg tablets (Phoenix Healthcare Distribution Ltd) (product)
17915611000001100	Gliclazide 80mg tablets (Phoenix Healthcare Distribution Ltd) (product)
17918011000001100	Glipizide 5mg tablets (Phoenix Healthcare Distribution Ltd) (product)

17940011000001100	Metformin 500mg tablets (Phoenix Healthcare Distribution Ltd) (product)
17970111000001100	Metformin 500mg/5ml oral solution sugar free (Almus Pharmaceuticals Ltd) (product)
17972811000001100	Glibenclamide 2.5mg tablets (Almus Pharmaceuticals Ltd) (product)
17973211000001100	Glibenclamide 5mg tablets (Almus Pharmaceuticals Ltd) (product)
18030311000001100	Insuman Infusat 100units/ml solution for injection 10ml vials (Imported (Germany)) (product)
18046311000001100	Insuman Infusat 100units/ml solution for injection 3.15ml cartridges (Imported (Germany)) (product)
18150311000001100	Insuman Comb 25 100units/ml suspension for injection 3ml pre-filled SoloStar pens (Sanofi) (product)
18155911000001100	Starlix 120mg tablets (Mawdsley-Brooks & Company Ltd) (product)
18212011000001100	Lantus 100units/ml solution for injection 10ml vials (Mawdsley-Brooks & Company Ltd) (product)
18224311000001100	Competact 15mg/850mg tablets (Necessity Supplies Ltd) (product)
18265111000001100	Glucobay 100mg tablets (Necessity Supplies Ltd) (product)
18309811000001100	Acarbose 100mg tablets (A A H Pharmaceuticals Ltd) (product)
18362011000001100	Metformin 500mg modified-release tablets (Mawdsley-Brooks & Company Ltd) (product)
18462111000001100	Gliclazide 80mg tablets (Accord Healthcare Ltd) (product)
18462611000001100	Glimepiride 3mg tablets (Accord Healthcare Ltd) (product)
18462811000001100	Glimepiride 4mg tablets (Accord Healthcare Ltd) (product)
184711000001100	Gliclazide 80mg tablets (Sterwin Medicines) (product)
18490911000001100	Lantus 100units/ml solution for injection 3ml pre-filled OptiSet pens (Necessity Supplies Ltd) (product)
18567011000001100	Pioglitazone 45mg/5ml oral suspension (Special Order) (product)
18580011000001100	Glucophage SR 500mg tablets (Doncaster Pharmaceuticals Ltd) (product)
18595311000001100	Pioglitazone 45mg/5ml oral suspension (product)
18624811000001100	Acarbose 50mg tablets (Phoenix Healthcare Distribution Ltd) (product)
18631911000001100	Onglyza 5mg tablets (Mawdsley-Brooks & Company Ltd) (product)
18678711000001100	Vitile XL 30mg tablets (Actavis UK Ltd) (product)
18745311000001100	Humalog KwikPen 100units/ml solution for injection 3ml pre-filled pens (Sigma Pharmaceuticals Plc) (product)
18745511000001100	Humalog Mix50 KwikPen 100units/ml suspension for injection 3ml pre-filled pens (Sigma Pharmaceuticals Plc) (product)
19274811000001100	Bydureon 2mg powder and solvent for suspension for injection vials (Eli Lilly and Company Ltd) (product)
19275411000001100	Exenatide 2mg powder and solvent for suspension for injection vials (product)
19276911000001100	Repaglinide 1mg tablets (Aspire Pharma Ltd) (product)
19277111000001100	Repaglinide 500microgram tablets (Aspire Pharma Ltd) (product)
19300511000001100	Repaglinide 1mg tablets (Actavis UK Ltd) (product)
19301211000001100	Metabet SR 1000mg tablets (Actavis UK Ltd) (product)
19306911000001100	Enyglid 1mg tablets (Consilient Health Ltd) (product)
19307311000001100	Enyglid 2mg tablets (Consilient Health Ltd) (product)
19354411000001100	Insuman Basal 100units/ml suspension for injection 3ml pre-filled SoloStar pens (Sanofi) (product)
19361111000001100	Repaglinide 2mg tablets (Alliance Healthcare (Distribution) Ltd) (product)
19361311000001100	Pioglitazone 15mg tablets (A A H Pharmaceuticals Ltd) (product)
19361711000001100	Repaglinide 500microgram tablets (A A H Pharmaceuticals Ltd) (product)
19362011000001100	Repaglinide 1mg tablets (A A H Pharmaceuticals Ltd) (product)
19362311000001100	Repaglinide 2mg tablets (A A H Pharmaceuticals Ltd) (product)
19469611000001100	Pioglitazone 45mg tablets (Alliance Healthcare (Distribution) Ltd) (product)
19473511000001100	Pioglitazone 45mg tablets (A A H Pharmaceuticals Ltd) (product)
19476411000001100	Pioglitazone 30mg tablets (Consilient Health Ltd) (product)
19476611000001100	Pioglitazone 45mg tablets (Consilient Health Ltd) (product)
19489511000001100	Acarbose 100mg tablets (Alliance Healthcare (Distribution) Ltd) (product)
19527011000001100	Actos 45mg tablets (Waymade Healthcare Plc) (product)
19570211000001100	NovoRapid FlexTouch 100units/ml solution for injection 3ml pre-filled pens (Novo Nordisk Ltd) (product)
19593311000001100	Pioglitazone 45mg tablets (Dr Reddy's Laboratories (UK) Ltd) (product)
19611511000001100	Eucreas 50mg/850mg tablets (Doncaster Pharmaceuticals Ltd) (product)
19613511000001100	Humalog Mix50 KwikPen 100units/ml suspension for injection 3ml pre-filled pens (DE Pharmaceuticals) (product)
19614111000001100	Humalog 100units/ml solution for injection 10ml vials (Doncaster Pharmaceuticals Ltd) (product)
19726511000001100	Actos 45mg tablets (Mawdsley-Brooks & Company Ltd) (product)
197511000001103	Metformin 500mg tablets (Unichem Plc) (product)
19818611000001100	Glipizide 5mg tablets (Almus Pharmaceuticals Ltd) (product)
19863911000001100	Actos 45mg tablets (Lexon (UK) Ltd) (product)
19957811000001100	Glizofar 15mg tablets (Teva UK Ltd) (product)
20022911000001100	Glizofar 15mg tablets (Arrow Generics Ltd) (product)
20023111000001100	Glizofar 30mg tablets (Arrow Generics Ltd) (product)
20023411000001100	Glizofar 45mg tablets (Arrow Generics Ltd) (product)
20114811000001100	Januvia 50mg Tablets (Merck Sharp & Dohme Ltd) (product)
20163611000001100	NovoRapid Penfill 100units/ml solution for injection 3ml cartridges (Doncaster Pharmaceuticals Ltd) (product)
20319611000001100	Pioglitazone 15mg tablets (Sandoz Ltd) (product)

20320211000001100	Pioglitazone 30mg tablets (Sandoz Ltd) (product)
20320611000001100	Pioglitazone 45mg tablets (Sandoz Ltd) (product)
20357611000001100	Pioglitazone 30mg tablets (Actavis UK Ltd) (product)
20566211000001100	Repaglinide 500microgram tablets (Creo Pharma Ltd) (product)
20566811000001100	Repaglinide 2mg tablets (Creo Pharma Ltd) (product)
20773611000001100	Repaglinide 500microgram tablets (Accord Healthcare Ltd) (product)
20778911000001100	Repaglinide 2mg tablets (Accord Healthcare Ltd) (product)
20925511000001100	Onglyza 5mg tablets (Waymade Healthcare Plc) (product)
21028111000001100	Acarbose 100mg tablets (Arrow Generics Ltd) (product)
21111411000001100	Acarbose 50mg tablets (Actavis UK Ltd) (product)
21208211000001100	Jentadueto 2.5mg/850mg tablets (Boehringer Ingelheim Ltd) (product)
21208511000001100	Jentadueto 2.5mg/1000mg tablets (Boehringer Ingelheim Ltd) (product)
21245011000001100	Linagliptin 2.5mg / Metformin 1g tablets (product)
21245111000001100	Linagliptin 2.5mg / Metformin 850mg tablets (product)
21366911000001100	Glimepiride 6mg/5ml oral suspension (product)
21409211000001100	Gliclazide 80mg tablets (Medreich Plc) (product)
21409811000001100	Metabet SR 500mg tablets (Actavis UK Ltd) (product)
21501311000001100	Glucophage SR 1000mg tablets (Waymade Healthcare Plc) (product)
21705311000001100	Komboglyze 2.5mg/850mg tablets (Bristol-Myers Squibb Pharmaceuticals Ltd) (product)
21705611000001100	Komboglyze 2.5mg/1000mg tablets (Bristol-Myers Squibb Pharmaceuticals Ltd) (product)
21711411000001100	Saxagliptin 2.5mg / Metformin 1g tablets (product)
21711511000001100	Saxagliptin 2.5mg / Metformin 850mg tablets (product)
21735211000001100	Acarbose 100mg tablets (Waymade Healthcare Plc) (product)
21763311000001100	Pioglitazone 15mg tablets (Teva UK Ltd) (product)
21764211000001100	Pioglitazone 30mg tablets (Teva UK Ltd) (product)
21764511000001100	Pioglitazone 45mg tablets (Teva UK Ltd) (product)
21793311000001100	Metformin 1g modified-release tablets (Waymade Healthcare Plc) (product)
21793711000001100	Metformin 500mg tablets (Waymade Healthcare Plc) (product)
21794511000001100	Metformin 850mg tablets (Waymade Healthcare Plc) (product)
21848111000001100	Pioglitazone 15mg tablets (Waymade Healthcare Plc) (product)
21848311000001100	Pioglitazone 30mg tablets (Waymade Healthcare Plc) (product)
21848511000001100	Pioglitazone 45mg tablets (Waymade Healthcare Plc) (product)
21880011000001100	Repaglinide 500microgram tablets (Waymade Healthcare Plc) (product)
21880611000001100	Repaglinide 2mg tablets (Waymade Healthcare Plc) (product)
21930011000001100	Tresiba FlexTouch 100units/ml solution for injection 3ml pre-filled pens (Novo Nordisk Ltd) (product)
21931911000001100	Tresiba FlexTouch 200units/ml solution for injection 3ml pre-filled pens (Novo Nordisk Ltd) (product)
21939611000001100	Insulin degludec 100units/ml solution for injection 3ml pre-filled disposable devices (product)
21939711000001100	Insulin degludec 200units/ml solution for injection 3ml pre-filled disposable devices (product)
21941511000001100	Lyxumia 10micrograms/0.2ml solution for injection 3ml pre-filled pens (Sanofi) (product)
21994811000001100	Lixisenatide 20micrograms/0.2ml solution for injection 3ml pre-filled disposable devices (product)
22026011000001100	Glibenclamide 2.5mg tablets (Waymade Healthcare Plc) (product)
22026511000001100	Gliclazide 40mg tablets (Waymade Healthcare Plc) (product)
22026711000001100	Gliclazide 80mg tablets (Waymade Healthcare Plc) (product)
22027911000001100	Glimepiride 3mg tablets (Waymade Healthcare Plc) (product)
22082511000001100	Gliclazide 30mg modified-release tablets (Waymade Healthcare Plc) (product)
22308811000001100	Bolamyn SR 1000mg tablets (Teva UK Ltd) (product)
22350011000001100	Metformin 850mg tablets (Aurobindo Pharma Ltd) (product)
22643511000001100	Lantus 100units/ml solution for injection 3ml OptiClik cartridges (Waymade Healthcare Plc) (product)
228111000001101	Amaryl 1mg tablets (Aventis Pharma) (product)
23369611000001100	Glidipion 30mg tablets (Actavis UK Ltd) (product)
23487411000001100	Pioglitazone 15mg tablets (Accord Healthcare Ltd) (product)
23487811000001100	Pioglitazone 45mg tablets (Accord Healthcare Ltd) (product)
23637311000001100	Alogliptin 12.5mg tablets (product)
23677911000001100	Sitagliptin 50mg tablets (Waymade Healthcare Plc) (product)
23678111000001100	Sitagliptin 100mg tablets (Waymade Healthcare Plc) (product)
23943211000001100	Glibenclamide 2.5mg tablets (Doncaster Pharmaceuticals Ltd) (product)
23943611000001100	Gliclazide 80mg tablets (Doncaster Pharmaceuticals Ltd) (product)
24018111000001100	Xigduo 5mg/850mg tablets (Bristol-Myers Squibb Pharmaceuticals Ltd) (product)
24018511000001100	Xigduo 5mg/1000mg tablets (Bristol-Myers Squibb Pharmaceuticals Ltd) (product)
24054711000001100	Dapagliflozin 5mg / Metformin 850mg tablets (product)
24088311000001100	Invokana 300mg tablets (Janssen-Cilag Ltd) (product)
24088611000001100	Invokana 100mg tablets (Janssen-Cilag Ltd) (product)
24106711000001100	Glimepiride 3mg tablets (DE Pharmaceuticals) (product)
24106911000001100	Glimepiride 4mg tablets (DE Pharmaceuticals) (product)
24134911000001100	Pioglitazone 30mg tablets (Morningside Healthcare Ltd) (product)
24137211000001100	Pioglitazone 15mg tablets (Morningside Healthcare Ltd) (product)
24380811000001100	Metformin 1g modified-release tablets (DE Pharmaceuticals) (product)
24381111000001100	Metformin 500mg modified-release tablets (DE Pharmaceuticals) (product)

24554511000001100	Metformin 1g modified-release tablets (Actavis UK Ltd) (product)
24568411000001100	Sukkarto SR 500mg tablets (Morningside Healthcare Ltd) (product)
25238811000001100	Jardiance 10mg tablets (Boehringer Ingelheim Ltd) (product)
25239711000001100	Jardiance 25mg tablets (Boehringer Ingelheim Ltd) (product)
25290511000001100	Empagliflozin 10mg tablets (product)
27117211000001100	Tolbutamide 500mg tablets (Genesis Pharmaceuticals Ltd) (product)
27879911000001100	Metformin 500mg/5ml oral solution sugar free (Pinewood Healthcare) (product)
27957011000001100	Ziclaseg 30mg modified-release tablets (Lupin (Europe) Ltd) (product)
28022511000001100	Vokanamet 50mg/850mg tablets (Janssen-Cilag Ltd) (product)
28024411000001100	Vokanamet 50mg/1000mg tablets (Janssen-Cilag Ltd) (product)
28049211000001100	Canagliflozin 50mg / Metformin 1g tablets (product)
28049311000001100	Canagliflozin 50mg / Metformin 850mg tablets (product)
28279611000001100	Insulin degludec 100units/ml / Liraglutide 3.6mg/ml solution for injection 3ml pre-filled disposable devices (product)
28420911000001100	Vamju 60mg modified-release tablets (AMCo) (product)
28425111000001100	Forxiga 5mg tablets (Waymade Healthcare Plc) (product)
28440211000001100	Exenatide 2mg powder and solvent for suspension for injection pre-filled disposable devices (product)
28462311000001100	Trulicity 1.5mg/0.5ml solution for injection pre-filled pens (Eli Lilly and Company Ltd) (product)
28775411000001100	Canagliflozin 300mg tablets (Colorama Pharmaceuticals Ltd) (product)
287811000001101	Diabetamide 5mg tablets (Ashbourne Pharmaceuticals Ltd) (product)
28789711000001100	Dulaglutide 1.5mg/0.5ml solution for injection pre-filled disposable devices (product)
28926811000001100	Humalog KwikPen 200units/ml solution for injection 3ml pre-filled pens (Eli Lilly and Company Ltd) (product)
28940811000001100	Glidipion 15mg tablets (Actavis UK Ltd) (product)
29700111000001100	Liraglutide 6mg/ml solution for injection 3ml pre-filled disposable devices (J M McGill Ltd) (product)
29737811000001100	Onglyza 2.5mg tablets (Waymade Healthcare Plc) (product)
29743011000001100	Diabiom 30mg tablets (Tillomed Laboratories Ltd) (product)
29743211000001100	Diabiom 45mg tablets (Tillomed Laboratories Ltd) (product)
29750011000001100	Sitagliptin 25mg tablets (Waymade Healthcare Plc) (product)
29754711000001100	Acarbose 50mg tablets (Sigma Pharmaceuticals Plc) (product)
29853611000001100	Canagliflozin 100mg tablets (Niche Pharma Ltd) (product)
29855111000001100	Metformin 1g oral powder sachets sugar free (Niche Pharma Ltd) (product)
29866811000001100	Toujeo 300units/ml solution for injection 1.5ml pre-filled SoloStar pens (Sanofi) (product)
29869711000001100	Gliclazide 80mg tablets (Sigma Pharmaceuticals Plc) (product)
29870011000001100	Glipizide 5mg tablets (Sigma Pharmaceuticals Plc) (product)
29903611000001100	Insulin glargine 300units/ml solution for injection 1.5ml pre-filled disposable devices (product)
29906011000001100	Metformin 500mg tablets (Sigma Pharmaceuticals Plc) (product)
29980811000001100	Byetta 10micrograms/0.04ml solution for injection 2.4ml pre-filled pens (Lexon (UK) Ltd) (product)
29981611000001100	Bydureon 2mg powder and solvent for suspension for injection vials (Lexon (UK) Ltd) (product)
29986211000001100	Pioglitazone 45mg tablets (Sigma Pharmaceuticals Plc) (product)
29988711000001100	Acarbose 50mg tablets (Mawdsley-Brooks & Company Ltd) (product)
29988911000001100	Acarbose 100mg tablets (Mawdsley-Brooks & Company Ltd) (product)
30056811000001100	Gliclazide 30mg modified-release tablets (DE Pharmaceuticals) (product)
30088811000001100	Repaglinide 1mg tablets (DE Pharmaceuticals) (product)
30089311000001100	Repaglinide 2mg tablets (DE Pharmaceuticals) (product)
30112311000001100	Pioglitazone 45mg tablets (DE Pharmaceuticals) (product)
30134911000001100	Gliclazide 80mg tablets (Mawdsley-Brooks & Company Ltd) (product)
30135211000001100	Gliclazide 30mg modified-release tablets (Mawdsley-Brooks & Company Ltd) (product)
30171811000001100	Abasaglar KwikPen 100units/ml solution for injection 3ml pre-filled pens (Eli Lilly and Company Ltd) (product)
30172211000001100	Abasaglar 100units/ml solution for injection 3ml cartridges (Eli Lilly and Company Ltd) (product)
30174111000001100	Synjardy 5mg/1000mg tablets (Boehringer Ingelheim Ltd) (product)
30175011000001100	Synjardy 12.5mg/850mg tablets (Boehringer Ingelheim Ltd) (product)
30175711000001100	Synjardy 12.5mg/1000mg tablets (Boehringer Ingelheim Ltd) (product)
30204011000001100	Glipizide 5mg tablets (Mawdsley-Brooks & Company Ltd) (product)
30208211000001100	Eucreas 50mg/1000mg tablets (Waymade Healthcare Plc) (product)
30214411000001100	Vipdomet 12.5mg/1000mg tablets (Waymade Healthcare Plc) (product)
30214611000001100	Vipidia 12.5mg tablets (Waymade Healthcare Plc) (product)
30269611000001100	Byetta 5micrograms/0.02ml solution for injection 1.2ml pre-filled pens (Waymade Healthcare Plc) (product)
30318211000001100	Empagliflozin 12.5mg / Metformin 850mg tablets (product)
30318311000001100	Empagliflozin 5mg / Metformin 1g tablets (product)
30318411000001100	Empagliflozin 5mg / Metformin 850mg tablets (product)
30763311000001100	Metformin 500mg modified-release tablets (Almus Pharmaceuticals Ltd) (product)
30828011000001100	Metformin 1g modified-release tablets (Mawdsley-Brooks & Company Ltd) (product)
30857211000001100	Pioglitazone 30mg tablets (Mawdsley-Brooks & Company Ltd) (product)

30857411000001100	Pioglitazone 45mg tablets (Mawdsley-Brooks & Company Ltd) (product)
30982411000001100	Bilxona 30mg modified-release tablets (Accord Healthcare Ltd) (product)
30989911000001100	Metformin 500mg modified-release tablets (Alliance Healthcare (Distribution) Ltd) (product)
30992711000001100	Insulin soluble human 1unit/ml solution for injection 50ml pre-filled syringes (product)
30993011000001100	Insulin soluble human 1unit/ml solution for injection 50ml pre-filled syringes (Special Order) (product)
31351711000001100	Alogliptin 12.5mg / Metformin 1g tablets (J M McGill Ltd) (product)
31352111000001100	Alogliptin 25mg tablets (J M McGill Ltd) (product)
32423011000001100	Metformin 1g modified-release tablets (Kent Pharmaceuticals Ltd) (product)
32432111000001100	Alogliptin 25mg tablets (Colorama Pharmaceuticals Ltd) (product)
32493411000001100	Glimepiride 1mg tablets (Brown & Burk UK Ltd) (product)
32493611000001100	Glimepiride 2mg tablets (Brown & Burk UK Ltd) (product)
32494011000001100	Glimepiride 4mg tablets (Brown & Burk UK Ltd) (product)
32498311000001100	Gliclazide 40mg tablets (Actavis UK Ltd) (product)
325011000001101	Metformin 850mg tablets (A A H Pharmaceuticals Ltd) (product)
325219009	Product containing precisely glibenclamide 5 milligram/1 each conventional release oral tablet (clinical drug)
325251005	Product containing precisely gliquidone 30 milligram/1 each conventional release oral tablet (clinical drug)
325259007	Product containing precisely glimepiride 2 milligram/1 each conventional release oral tablet (clinical drug)
325261003	Product containing precisely glimepiride 1 milligram/1 each conventional release oral tablet (clinical drug)
325267004	Product containing precisely tolbutamide 500 milligram/1 each conventional release oral tablet (clinical drug)
325278007	Product containing precisely metformin hydrochloride 500 milligram/1 each conventional release oral tablet (clinical drug)
3256111000001100	Insulatard ge 100units/ml suspension for injection 10ml vials (Novo Nordisk Pharmaceuticals Ltd) (product)
3259411000001100	Human Insulatard Penfill 100units/ml suspension for injection 1.5ml cartridges (Novo Nordisk Pharmaceuticals Ltd) (product)
3259811000001100	Insuman Comb 25 100units/ml suspension for injection 3ml pre-filled OptiSet pens (Sanofi) (product)
326038002	Product containing precisely acarbose 100 milligram/1 each conventional release oral tablet (clinical drug)
326047005	Product containing precisely repaglinide 500 microgram/1 each conventional release oral tablet (clinical drug)
326061000	Product containing precisely pioglitazone (as pioglitazone hydrochloride) 30 milligram/1 each conventional release oral tablet (clinical drug)
3260811000001100	Humulin I Pen 100units/ml suspension for injection 3ml pre-filled pens (Eli Lilly and Company Ltd) (product)
3261411000001100	Insulatard InnoLet 100units/ml suspension for injection 3ml pre-filled pens (Novo Nordisk Ltd) (product)
3263011000001100	Insuman Comb 25 100units/ml suspension for injection 5ml vials (Aventis Pharma) (product)
3263611000001100	Humulin M2 100units/ml suspension for injection 3ml cartridges (Eli Lilly & Co Ltd) (product)
32638311000001100	Sitagliptin 50mg/5ml oral solution (product)
3264211000001100	Humulin Isophane 100units/ml suspension for injection 3ml cartridges (Eli Lilly & Co Ltd) (product)
3264411000001100	Mixtard 20 Penfill 100units/ml suspension for injection 3ml cartridges (Novo Nordisk Pharmaceuticals Ltd) (product)
3268711000001100	Human Mixtard 10 Penfill 100units/ml suspension for injection 1.5ml cartridges (Novo Nordisk Pharmaceuticals Ltd) (product)
3269711000001100	Mixtard 10 NovoLet 100units/ml suspension for injection (Novo Nordisk Pharmaceuticals Ltd) (product)
3269911000001100	Hypurin Bovine Protamine Zinc 100units/ml suspension for injection 10ml vials (C P Pharmaceuticals Ltd) (product)
3270211000001100	Insuman Comb 15 100units/ml suspension for injection 3ml pre-filled OptiSet pens (Sanofi) (product)
3270611000001100	Human Mixtard 50 100units/ml suspension for injection 10ml vials (Novo Nordisk Pharmaceuticals Ltd) (product)
3271311000001100	Hypurin Bovine Neutral 100units/ml solution for injection 10ml vials (C P Pharmaceuticals Ltd) (product)
3271611000001100	Mixtard 30 InnoLet 100units/ml suspension for injection 3ml pre-filled pens (Novo Nordisk Ltd) (product)
3271711000001100	Insuman Comb 15 100units/ml suspension for injection 5ml vials (Aventis Pharma) (product)
3272211000001100	Humaject M3 Pen 100units/ml suspension for injection (Eli Lilly & Co Ltd) (product)
3272411000001100	Insuman Comb 50 100units/ml suspension for injection 3ml cartridges (Aventis Pharma) (product)
3273111000001100	Mixtard 30 Penfill 100units/ml suspension for injection 3ml cartridges (Novo Nordisk Pharmaceuticals Ltd) (product)
3273411000001100	Human Mixtard 50 Penfill 100units/ml suspension for injection 1.5ml cartridges (Novo Nordisk Pharmaceuticals Ltd) (product)

3273911000001100	Insuman Comb 15 100units/ml suspension for injection 3ml cartridges (Aventis Pharma) (product)
3274011000001100	Hypurin Bovine Neutral 100units/ml solution for injection 1.5ml cartridges (C P Pharmaceuticals Ltd) (product)
3274811000001100	Humulin M3 100units/ml suspension for injection 10ml vials (Eli Lilly & Co Ltd) (product)
3275311000001100	Human Mixtard 30 Penfill 100units/ml suspension for injection 1.5ml cartridges (Novo Nordisk Pharmaceuticals Ltd) (product)
3275711000001100	Humalog Mix25 100units/ml suspension for injection 3ml cartridges (Eli Lilly & Co Ltd) (product)
3276011000001100	Humalog Mix25 Pen 100units/ml suspension for injection 3ml pre-filled pens (Eli Lilly and Company Ltd) (product)
3277811000001100	NovoMix 30 Penfill 100units/ml suspension for injection 3ml cartridges (Novo Nordisk Pharmaceuticals Ltd) (product)
3278611000001100	Insuman Comb 50 100units/ml suspension for injection 3ml pre-filled OptiSet pens (Sanofi) (product)
3279211000001100	NovoRapid Penfill 100units/ml solution for injection 3ml cartridges (Novo Nordisk Pharmaceuticals Ltd) (product)
3280611000001100	Hypurin Bovine Lente 100units/ml suspension for injection 10ml vials (C P Pharmaceuticals Ltd) (product)
3280711000001100	NovoRapid 100units/ml solution for injection 10ml vials (Novo Nordisk Pharmaceuticals Ltd) (product)
3281811000001100	Humulin Zn 100units/ml suspension for injection 10ml vials (Eli Lilly & Co Ltd) (product)
3282211000001100	NovoRapid FlexPen 100units/ml solution for injection 3ml pre-filled pens (Novo Nordisk Ltd) (product)
3282311000001100	Ultratard 100units/ml suspension for injection 10ml vials (Novo Nordisk Pharmaceuticals Ltd) (product)
3282611000001100	Hypurin Porcine Isophane 100units/ml suspension for injection 1.5ml cartridges (C P Pharmaceuticals Ltd) (product)
3284011000001100	Hypurin Porcine Isophane 100units/ml suspension for injection 10ml vials (C P Pharmaceuticals Ltd) (product)
3284111000001100	Monotard 100units/ml suspension for injection 10ml vials (Novo Nordisk Pharmaceuticals Ltd) (product)
3284211000001100	Lantus 100units/ml solution for injection 3ml cartridges (Aventis Pharma) (product)
3284311000001100	Humalog 100units/ml solution for injection 3ml cartridges (Eli Lilly & Co Ltd) (product)
3285511000001100	Human Mixtard 40 Penfill 100units/ml suspension for injection 1.5ml cartridges (Novo Nordisk Pharmaceuticals Ltd) (product)
3285611000001100	Pork Actrapid 100units/ml solution for injection 10ml vials (Novo Nordisk Pharmaceuticals Ltd) (product)
3287911000001100	Lantus 100units/ml solution for injection 10ml vials (Aventis Pharma) (product)
3291711000001100	Insuman Rapid 100units/ml solution for injection 3ml pre-filled OptiSet pens (Sanofi) (product)
3311311000001100	Humulin S 100units/ml solution for injection 3ml cartridges (Eli Lilly & Co Ltd) (product)
3311611000001100	Insuman Rapid 100units/ml solution for injection 3ml cartridges (Aventis Pharma) (product)
3312111000001100	Actrapid 100units/ml solution for injection 10ml vials (Novo Nordisk Pharmaceuticals Ltd) (product)
3312411000001100	Velosulin 100units/ml solution for injection 10ml vials (Novo Nordisk Pharmaceuticals Ltd) (product)
3333111000001100	Pork Insulatard 100units/ml suspension for injection 10ml vials (Novo Nordisk Pharmaceuticals Ltd) (product)
33425611000001100	Metformin 750mg modified-release tablets (A A H Pharmaceuticals Ltd) (product)
33428211000001100	Gliclazide 40mg tablets (Alliance Healthcare (Distribution) Ltd) (product)
33548011000001100	Metformin 1g/5ml oral solution sugar free (Colonis Pharma Ltd) (product)
33550911000001100	Metformin 850mg/5ml oral solution sugar free (product)
33598711000001100	Pioglitazone 45mg tablets (Mylan Ltd) (product)
33598911000001100	Pioglitazone 30mg tablets (Mylan Ltd) (product)
33613711000001100	Repaglinide 500microgram tablets (Mylan Ltd) (product)
33614311000001100	Repaglinide 1mg tablets (Mylan Ltd) (product)
33747711000001100	Saxenda 6mg/ml solution for injection 3ml pre-filled pens (Novo Nordisk Ltd) (product)
33766211000001100	Zicron PR 30mg tablets (Bristol Laboratories Ltd) (product)
33769011000001100	Metformin 850mg/5ml oral solution sugar free (Alliance Healthcare (Distribution) Ltd) (product)
33769211000001100	Metformin 1g/5ml oral solution sugar free (Alliance Healthcare (Distribution) Ltd) (product)
3468711000001100	Insulin aspart 100units/ml solution for injection 3ml pre-filled disposable devices (product)
3468811000001100	Insulin aspart biphasic 30/70 100units/ml suspension for injection 3ml cartridges (product)
3468911000001100	Insulin aspart biphasic 30/70 100units/ml suspension for injection 3ml pre-filled disposable devices (product)
3469311000001100	Insulin isophane biphasic human 10/90 100units/ml suspension for injection 1.5ml cartridges (product)

3469411000001100	Insulin isophane biphasic human 10/90 100units/ml suspension for injection 3ml cartridges (product)
3469511000001100	Insulin isophane biphasic human 10/90 100units/ml suspension for injection 3ml pre-filled disposable devices (product)
3469611000001100	Insulin isophane biphasic human 15/85 100units/ml suspension for injection 3ml cartridges (product)
3469711000001100	Insulin isophane biphasic human 15/85 100units/ml suspension for injection 3ml pre-filled disposable devices (product)
3469911000001100	Insulin isophane biphasic human 20/80 100units/ml suspension for injection 1.5ml cartridges (product)
3470011000001100	Insulin isophane biphasic human 20/80 100units/ml suspension for injection 3ml cartridges (product)
3470311000001100	Insulin isophane biphasic human 25/75 100units/ml suspension for injection 3ml pre-filled disposable devices (product)
3470411000001100	Insulin isophane biphasic human 25/75 100units/ml suspension for injection 5ml vials (product)
3470511000001100	Insulin isophane biphasic human 30/70 100units/ml suspension for injection 1.5ml cartridges (product)
3470911000001100	Insulin isophane biphasic human 30/70 100units/ml suspension for injection 3ml cartridges (product)
3471111000001100	Insulin isophane biphasic human 40/60 100units/ml suspension for injection 1.5ml cartridges (product)
3471211000001100	Insulin isophane biphasic human 40/60 100units/ml suspension for injection 3ml cartridges (product)
3471411000001100	Insulin isophane biphasic human 50/50 100units/ml suspension for injection 1.5ml cartridges (product)
3471511000001100	Insulin isophane biphasic human 50/50 100units/ml suspension for injection 10ml vials (product)
3471611000001100	Insulin isophane biphasic human 50/50 100units/ml suspension for injection 3ml cartridges (product)
3471811000001100	Insulin isophane biphasic human 50/50 100units/ml suspension for injection 5ml vials (product)
3471911000001100	Insulin isophane biphasic porcine 30/70 100units/ml suspension for injection 1.5ml cartridges (product)
3472911000001100	Insulin isophane porcine 100units/ml suspension for injection 1.5ml cartridges (product)
3473511000001100	Insulin lispro biphasic 25/75 100units/ml suspension for injection 3ml pre-filled disposable devices (product)
3473611000001100	Insulin lispro biphasic 50/50 100units/ml suspension for injection 3ml pre-filled disposable devices (product)
3473711000001100	Insulin lispro biphasic 25/75 100units/ml suspension for injection 3ml cartridges (product)
3474911000001100	Insulin zinc mixed bovine 100units/ml suspension for injection 10ml vials (product)
3650611000001100	Starlix 60mg tablets (Novartis Pharmaceuticals UK Ltd) (product)
3661311000001100	Diamicron 30mg MR tablets (Servier Laboratories Limited) (product)
374897009	Product containing precisely pioglitazone (as pioglitazone hydrochloride) 45 milligram/1 each conventional release oral tablet (clinical drug)
386047000	Metformin hydrochloride 500mg m/r tablet (product)
400780006	Insulin glargine 100units/mL injection solution 3mL cartridge (product)
400877001	Insulin glargine 100units/mL injection solution 3mL prefilled disposable injection device (product)
4028811000001100	Hypurin Bovine Neutral 100units/ml solution for injection 3ml cartridges (C P Pharmaceuticals Ltd) (product)
4033111000001100	Insulin isophane biphasic porcine 30/70 100units/ml suspension for injection 3ml cartridges (product)
4034311000001100	Hypurin Porcine Isophane 100units/ml suspension for injection 3ml cartridges (C P Pharmaceuticals Ltd) (product)
4053611000001100	Insulin isophane porcine 100units/ml suspension for injection 3ml cartridges (product)
409122001	Product containing precisely metformin hydrochloride 500 milligram and rosiglitazone (as rosiglitazone maleate) 2 milligram/1 each conventional release oral tablet (clinical drug)
409124000	Product containing precisely metformin hydrochloride 1 gram and rosiglitazone (as rosiglitazone maleate) 2 milligram/1 each conventional release oral tablet (clinical drug)
409197000	Product containing precisely metformin hydrochloride 100 milligram/1 milliliter conventional release oral solution (clinical drug)
423962004	Product containing precisely sitagliptin (as sitagliptin phosphate) 25 milligram/1 each conventional release oral tablet (clinical drug)
424513004	Product containing precisely sitagliptin (as sitagliptin phosphate) 50 milligram/1 each conventional release oral tablet (clinical drug)
444311000001106	Glucobay 100 tablets (Bayer Plc) (product)
446711000001101	Actos 30mg tablets (Takeda UK Ltd) (product)
450011000001104	Tolbutamide 500mg tablets (A A H Pharmaceuticals Ltd) (product)
494111000001103	NovoNorm 1mg tablets (Novo Nordisk Pharmaceuticals Ltd) (product)
498911000001107	Gliclazide 80mg tablets (Alpharma Limited) (product)
515111000001108	Glibenese 5mg tablets (Pfizer Ltd) (product)
5199411000001100	Actos 45mg tablets (Takeda UK Ltd) (product)

5268911000001100	Humalog Mix25 100units/ml suspension for injection 3ml cartridges (PI) (Waymade Ltd) (product)
5270711000001100	NovoRapid Novolet 100units/ml solution for injection (PI) (Waymade Ltd) (product)
5283711000001100	NovoMix 30 FlexPen 100units/ml suspension for injection 3ml pre-filled pens (Waymade Healthcare Plc) (product)
5329011000001100	Actos 15mg tablets (PI) (Waymade Ltd) (product)
5330711000001100	Amaryl 2mg tablets (PI) (Waymade Ltd) (product)
5330811000001100	Amaryl 3mg tablets (PI) (Waymade Ltd) (product)
5335311000001100	Amaryl 1mg tablets (PI) (Waymade Ltd) (product)
5337611000001100	Avandia 8mg tablets (PI) (Waymade Ltd) (product)
535811000001108	Glibenclamide 5mg tablets (Approved Prescription Services) (product)
5372211000001100	Glucobay 100 tablets (PI) (Waymade Ltd) (product)
5396011000001100	NovoNorm 1mg tablets (PI) (Waymade Ltd) (product)
5414811000001100	Starlix 120mg tablets (PI) (Waymade Ltd) (product)
5440011000001100	Glucobay 100 tablets (PI) (Dowelhurst Ltd) (product)
5449611000001100	Glimepiride 3mg tablets (PI) (Dowelhurst Ltd) (product)
5456111000001100	Glibenclamide 5mg tablets (PI) (Dowelhurst Ltd) (product)
5465811000001100	Acarbose 50mg tablets (PI) (Dowelhurst Ltd) (product)
551111000001104	Gliclazide 80mg tablets (Unichem Plc) (product)
5519911000001100	Amaryl 1mg tablets (PI) (Dowelhurst Ltd) (product)
5520411000001100	Amaryl 2mg tablets (PI) (Dowelhurst Ltd) (product)
5521211000001100	Amaryl 4mg tablets (PI) (Dowelhurst Ltd) (product)
5532711000001100	Daonil 5mg tablets (PI) (Dowelhurst Ltd) (product)
5548011000001100	Glucobay 50 tablets (PI) (Dowelhurst Ltd) (product)
58011000001106	Actos 15mg tablets (Takeda UK Ltd) (product)
593911000001106	Metformin 500mg tablets (Sandoz Ltd) (product)
61911000001108	Glimil 80mg tablets (Milpharm Ltd) (product)
637811000001108	Metformin 850mg tablets (Sterwin Medicines) (product)
700011000001100	Glibenclamide 2.5mg tablets (Alpharma Limited) (product)
7034411000001100	Metformin 850mg tablets (IVAX Pharmaceuticals UK Ltd) (product)
703679006	Product containing precisely dapagliflozin propanediol 5 milligram/1 each conventional release oral tablet (clinical drug)
703680009	Product containing precisely dapagliflozin propanediol 10 milligram/1 each conventional release oral tablet (clinical drug)
718311000001102	Glibenclamide 5mg tablets (Unichem Plc) (product)
7463711000001100	Metformin 850mg tablets (Ranbaxy (UK) Ltd) (product)
7589411000001100	Levemir FlexPen 100units/ml solution for injection 3ml pre-filled pens (Novo Nordisk Ltd) (product)
781411000001104	Glucobay 50 tablets (Bayer Plc) (product)
785311000001108	Metformin 500mg tablets (Sterwin Medicines) (product)
8093711000001100	Glimepiride 2mg tablets (Unichem Plc) (product)
8093911000001100	Glimepiride 3mg tablets (Unichem Plc) (product)
811811000001103	Glipizide 5mg tablets (Kent Pharmaceuticals Ltd) (product)
8174611000001100	Avandamet 4mg/1000mg tablets (GlaxoSmithKline) (product)
840811000001100	NovoNorm 2mg tablets (Novo Nordisk Pharmaceuticals Ltd) (product)
8494911000001100	Glibenclamide 5mg/5ml oral solution (Special Order) (product)
8496711000001100	Gliclazide 80mg/5ml oral suspension (Special Order) (product)
8497111000001100	Gliclazide 40mg/5ml oral suspension (Special Order) (product)
8523811000001100	Glibenclamide 5mg/5ml oral suspension (product)
8523911000001100	Glibenclamide 7.5mg/5ml oral solution (product)
8524111000001100	Gliclazide 160mg/5ml oral suspension (product)
8524211000001100	Gliclazide 40mg/5ml oral suspension (product)
859811000001106	Metformin 850mg tablets (Kent Pharmaceuticals Ltd) (product)
860011000001104	Gliclazide 80mg tablets (IVAX Pharmaceuticals UK Ltd) (product)
8614211000001100	Metformin 425mg/5ml oral solution (Special Order) (product)
8615911000001100	Metformin 500mg/5ml oral solution (Special Order) (product)
8616711000001100	Metformin 500mg/5ml oral suspension (Special Order) (product)
8617611000001100	Metformin 850mg/5ml oral solution (Special Order) (product)
8618911000001100	Metformin 850mg/5ml oral suspension (Special Order) (product)
864711000001108	Tolbutamide 500mg tablets (Alpharma Limited) (product)
8663911000001100	Metformin 250mg/5ml oral solution (product)
8664011000001100	Metformin 250mg/5ml oral suspension (product)
8664111000001100	Metformin 425mg/5ml oral solution (product)
8664411000001100	Metformin 500mg/5ml oral suspension (product)
8664611000001100	Metformin 850mg/5ml oral solution (product)
8664711000001100	Metformin 850mg/5ml oral suspension (product)
8712011000001100	Tolbutamide 500mg/5ml oral suspension (Special Order) (product)
8724011000001100	Tolbutamide 500mg/5ml oral suspension (product)
8759611000001100	Metformin 850mg capsules (Special Order) (product)
8796711000001100	Metformin 850mg capsules (product)
889611000001103	Metformin 500mg tablets (Approved Prescription Services) (product)
892811000001108	Gliclazide 80mg tablets (Approved Prescription Services) (product)
894411000001104	Glibenclamide 2.5mg tablets (C P Pharmaceuticals Ltd) (product)
8990711000001100	Glucophage SR 500mg tablets (Merck Pharmaceuticals) (product)
918011000001106	Glibenclamide 2.5mg tablets (Approved Prescription Services) (product)
923911000001109	Glibenclamide 2.5mg tablets (IVAX Pharmaceuticals UK Ltd) (product)
925311000001109	Gliclazide 80mg tablets (Genus Pharmaceuticals) (product)

938211000001105	Amaryl 2mg tablets (Aventis Pharma) (product)
9437511000001100	Humulin M3 Pen 100units/ml suspension for injection 3ml pre-filled pens (Eli Lilly and Company Ltd) (product)
9528311000001100	Apidra 100units/ml solution for injection 3ml cartridges (sanofi-aventis) (product)
9532111000001100	Insulin glulisine 100units/ml solution for injection 3ml cartridges (product)
9745811000001100	Glipizide 5mg tablets (Teva UK Ltd) (product)
9752811000001100	Gliclazide 80mg tablets (Milpharm Ltd) (product)
9797311000001100	Metformin 500mg tablets (Almus Pharmaceutical Ltd) (product)
34043011000001100	Fiasp FlexTouch 100units/ml solution for injection 3ml pre-filled pens (Novo Nordisk Ltd) (product)
34043211000001100	Fiasp Penfill 100units/ml solution for injection 3ml cartridges (Novo Nordisk Ltd) (product)
34188211000001100	Metformin 1g/5ml oral solution sugar free (A A H Pharmaceuticals Ltd) (product)
34346811000001100	Metformin 850mg tablets (Crescent Pharma Ltd) (product)
34347311000001100	Metformin 500mg tablets (Crescent Pharma Ltd) (product)
34552411000001100	Meijumet 500mg modified-release tablets (Medreich Plc) (product)
34553311000001100	Meijumet 1000mg modified-release tablets (Medreich Plc) (product)
12019111000001100	Chlorpropamide 250mg/5ml oral suspension (product)
31014311000001100	Eperzan 30mg powder and solvent for solution for injection pre-filled pens (GlaxoSmithKline UK Ltd) (product)
31014611000001100	Eperzan 50mg powder and solvent for solution for injection pre-filled pens (GlaxoSmithKline UK Ltd) (product)
31015711000001100	Albiglutide 30mg powder and solvent for solution for injection pre-filled disposable devices (product)
31015811000001100	Albiglutide 50mg powder and solvent for solution for injection pre-filled disposable devices (product)
325214004	Product containing precisely chlorpropamide 250 milligram/1 each conventional release oral tablet (clinical drug)
32860911000001100	Tolazamide 250mg tablets (Imported (United States)) (product)
32861211000001100	Tolazamide 500mg tablets (Imported (United States)) (product)
3472211000001100	Insulin isophane bovine 100units/ml suspension for injection 1.5ml cartridges (product)
3950411000001100	Chlorpropamide 250mg tablets (The Boots Company) (product)
4028311000001100	Hypurin Bovine Isophane 100units/ml suspension for injection 3ml cartridges (C P Pharmaceuticals Ltd) (product)
4033211000001100	Insulin isophane bovine 100units/ml suspension for injection 3ml cartridges (product)
30933411000001100	Alogliptin 25mg tablets (Ennogen Healthcare Ltd) (product)
32181311000001100	Alogliptin 12.5mg / Metformin 1g tablets (Niche Pharma Ltd) (product)
32182511000001100	Alogliptin 12.5mg tablets (Niche Pharma Ltd) (product)
35216811000001100	Humalog Junior KwikPen 100units/ml solution for injection 3ml pre-filled pens (Eli Lilly and Company Ltd) (product)
35548311000001100	Yaltormin SR 1000mg tablets (Wockhardt UK Ltd) (product)
35563111000001100	Insulin lispro 100units/ml solution for injection 3ml pre-filled pen (Sanofi Pasteur) (product)
35563711000001100	Insulin lispro 100units/ml solution for injection 10ml vials (Sanofi Pasteur) (product)
35776411000001100	Insulin lispro Sanofi 100units/ml solution for injection 10ml vials (Sanofi) (product)
35776811000001100	Insulin lispro Sanofi 100units/ml solution for injection 3ml pre-filled pens (Sanofi) (product)
36047311000001100	Insulin isophane human 100units/ml suspension for injection 3ml cartridges (product)
36047411000001100	Insulin isophane human 100units/ml suspension for injection 3ml pre-filled disposable devices (product)
36047511000001100	Insulin lispro 100units/ml solution for injection 1.5ml cartridges (product)
36047611000001100	Insulin lispro 100units/ml solution for injection 10ml vials (product)
36047911000001100	Insulin protamine zinc bovine 100units/ml suspension for injection 10ml vials (product)
36048111000001100	Insulin soluble bovine 100units/ml solution for injection 10ml vials (product)
36048411000001100	Insulin soluble human 100units/ml solution for injection 3ml cartridges (product)
36048511000001100	Insulin soluble human 100units/ml solution for injection 3ml pre-filled disposable devices (product)
36048611000001100	Insulin soluble human 100units/ml solution for injection 5ml vials (product)
36048811000001100	Insulin zinc crystalline human 100units/ml suspension for injection 10ml vials (product)
36804811000001100	Jardiance 25mg tablets (Originalis B.V.) (product)
36857411000001100	Pioglitazone 45mg tablets (Torrent Pharma (UK) Ltd) (product)
36889711000001100	Januvia 25mg tablets (Pharmaram Ltd) (product)
36889911000001100	Januvia 50mg tablets (Pharmaram Ltd) (product)
36893611000001100	Trajenta 5mg tablets (Pharmaram Ltd) (product)
36904411000001100	Victoza 6mg/ml solution for injection 3ml pre-filled pens (Originalis B.V.) (product)
36910511000001100	Glydex 160mg tablets (Medreich Plc) (product)
37120611000001100	Metformin 850mg tablets (Mawdsley-Brooks & Company Ltd) (product)
36618311000001100	Suliqua 100units/ml / 50micrograms/ml solution for injection 3ml pre-filled SoloStar pens (Sanofi) (product)
36620611000001100	Suliqua 100units/ml / 33micrograms/ml solution for injection 3ml pre-filled SoloStar pens (Sanofi) (product)

37337511000001100	Amglidia 6mg/ml oral suspension with 1ml oral syringe (Amring Pharmaceuticals Ltd) (product)
37406011000001100	Glibenclamide 6mg/ml oral suspension sugar free (product)
37419911000001100	Humulin M3 100units/ml suspension for injection 10ml vials (CST Pharma Ltd) (product)
37428211000001100	Actos 30mg tablets (CST Pharma Ltd) (product)
37432611000001100	Byetta 10micrograms/0.04ml solution for injection 2.4ml pre-filled pens (CST Pharma Ltd) (product)
37436211000001100	Eucreas 50mg/1000mg tablets (CST Pharma Ltd) (product)
37437511000001100	Forxiga 10mg tablets (CST Pharma Ltd) (product)
37437811000001100	Forxiga 5mg tablets (CST Pharma Ltd) (product)
37439411000001100	Humalog Mix25 KwikPen 100units/ml suspension for injection 3ml pre-filled pens (CST Pharma Ltd) (product)
37440011000001100	Janumet 50mg/1000mg tablets (CST Pharma Ltd) (product)
37440211000001100	Januvia 100mg tablets (CST Pharma Ltd) (product)
37440611000001100	Januvia 50mg tablets (CST Pharma Ltd) (product)
37449011000001100	Metformin 500mg/5ml oral solution sugar free (DE Pharmaceuticals) (product)
37509111000001100	Glucophage 850mg tablets (Mawdsley-Brooks & Company Ltd) (product)
37512311000001100	Victoza 6mg/ml solution for injection 3ml pre-filled pens (Mawdsley-Brooks & Company Ltd) (product)
37512811000001100	Invokana 300mg tablets (Mawdsley-Brooks & Company Ltd) (product)
37523211000001100	Janumet 50mg/1000mg tablets (Mawdsley-Brooks & Company Ltd) (product)
37525111000001100	Januvia 25mg tablets (Mawdsley-Brooks & Company Ltd) (product)
37526411000001100	Jardiance 10mg tablets (Mawdsley-Brooks & Company Ltd) (product)
37526711000001100	Jardiance 25mg tablets (Mawdsley-Brooks & Company Ltd) (product)
37527011000001100	Jentadueto 2.5mg/1000mg tablets (Mawdsley-Brooks & Company Ltd) (product)
37528111000001100	Jentadueto 2.5mg/850mg tablets (Mawdsley-Brooks & Company Ltd) (product)
37528511000001100	Komboglyze 2.5mg/1000mg tablets (Mawdsley-Brooks & Company Ltd) (product)
37536011000001100	Trajenta 5mg tablets (CST Pharma Ltd) (product)
37538111000001100	Vipidia 6.25mg tablets (CST Pharma Ltd) (product)
37550311000001100	Onglyza 2.5mg tablets (Mawdsley-Brooks & Company Ltd) (product)
37590811000001100	Apidra 100units/ml solution for injection 3ml cartridges (CST Pharma Ltd) (product)
37591211000001100	Bydureon 2mg powder and solvent for prolonged-release suspension for injection pre-filled pens (CST Pharma Ltd) (product)
37618711000001100	Bilxona 60mg modified-release tablets (Accord Healthcare Ltd) (product)
37625811000001100	Competact 15mg/850mg tablets (Pilsco Ltd) (product)
37636511000001100	Eucreas 50mg/1000mg tablets (Pilsco Ltd) (product)
37672511000001100	Lantus 100units/ml solution for injection 3ml pre-filled SoloStar pens (Pilsco Ltd) (product)
37694811000001100	NovoMix 30 FlexPen 100units/ml suspension for injection 3ml pre-filled pens (Pilsco Ltd) (product)
37695311000001100	NovoRapid FlexPen 100units/ml solution for injection 3ml pre-filled pens (Pilsco Ltd) (product)
37695811000001100	NovoRapid Penfill 100units/ml solution for injection 3ml cartridges (Pilsco Ltd) (product)
37709111000001100	Vipidia 12.5mg tablets (Pilsco Ltd) (product)
37716411000001100	Byetta 10micrograms/0.04ml solution for injection 2.4ml pre-filled pens (Pilsco Ltd) (product)
37750911000001100	Tresiba Penfill 100units/ml solution for injection 3ml cartridges (CST Pharma Ltd) (product)
21635011000001100	Dapagliflozin 10mg tablets (product)
21635211000001100	Dapagliflozin 5mg tablets (product)
24104411000001100	Canagliflozin 100mg tablets (product)
325065003	Insulin lispro 100units/mL injection solution 10mL vial (product)
325100008	Insulin zinc extended release 100units/mL injection 10mL vial (product)
325143000	Insulin protamine zinc bovine 100units/mL injection 10mL vial (product)
3469211000001100	Insulin glargine 100units/ml solution for injection 3ml pre-filled disposable devices (product)
371485002	Insulin soluble human 100u/mL injection solution 1.5mL cartridge (product)
37852711000001100	Synjardy 5mg/1000mg tablets (CST Pharma Ltd) (product)
400844000	Insulin zinc suspension mixed human 100units/ml injection 10ml vial (product)
400847007	Insulin glargine 100units/mL injection solution 10mL vial (product)
5211211000001100	Pioglitazone 45mg tablets (product)
5322711000001100	Metformin 500mg / Rosiglitazone 2mg tablets (product)
8664311000001100	Metformin 500mg/5ml oral solution (product)
8991411000001100	Metformin 500mg modified-release tablets (product)
37971811000001100	Humalog 100units/ml solution for injection 3ml cartridges (Pharmaram Ltd) (product)
37973011000001100	NovoRapid FlexPen 100units/ml solution for injection 3ml pre-filled pens (Pharmaram Ltd) (product)
37974111000001100	Vipidia 6.25mg tablets (Pharmaram Ltd) (product)
37989011000001100	Eucreas 50mg/1000mg tablets (Pharmaram Ltd) (product)
37989211000001100	Forxiga 5mg tablets (Pharmaram Ltd) (product)
37992511000001100	NovoMix 30 FlexPen 100units/ml suspension for injection 3ml pre-filled pens (Pharmaram Ltd) (product)
37992711000001100	Onglyza 2.5mg tablets (Pharmaram Ltd) (product)
37992911000001100	Onglyza 5mg tablets (Pharmaram Ltd) (product)

	38018211000001100	Lamzarin 30mg modified-release tablets (Key Pharmaceuticals Ltd) (product)
	38018511000001100	Lamzarin 60mg modified-release tablets (Key Pharmaceuticals Ltd) (product)
	38074211000001100	Byetta 10micrograms/0.04ml solution for injection 2.4ml pre-filled pens (Pharmaram Ltd) (product)
	38074811000001100	Tresiba FlexTouch 200units/ml solution for injection 3ml pre-filled pens (Pharmaram Ltd) (product)
	38082811000001100	Exenatide 2mg/0.85ml prolonged-release suspension for injection pre-filled disposable devices (product)
	38123811000001100	Byetta 5micrograms/0.02ml solution for injection 1.2ml pre-filled pens (DE Pharmaceuticals) (product)
	38135511000001100	Forxiga 5mg tablets (DE Pharmaceuticals) (product)
	38139511000001100	Humulin I 100units/ml suspension for injection 3ml cartridges (DE Pharmaceuticals) (product)
	38139911000001100	Humulin M3 100units/ml suspension for injection 10ml vials (DE Pharmaceuticals) (product)
	38141711000001100	Januvia 25mg tablets (DE Pharmaceuticals) (product)
	38142111000001100	Jardiance 10mg tablets (DE Pharmaceuticals) (product)
	38142411000001100	Jardiance 25mg tablets (DE Pharmaceuticals) (product)
	38142711000001100	Jentadueto 2.5mg/850mg tablets (DE Pharmaceuticals) (product)
	38144011000001100	Komboglyze 2.5mg/1000mg tablets (DE Pharmaceuticals) (product)
	38144911000001100	Lantus 100units/ml solution for injection 3ml cartridges (DE Pharmaceuticals) (product)
	38145111000001100	Lantus 100units/ml solution for injection 10ml vials (DE Pharmaceuticals) (product)
	38145311000001100	Lantus 100units/ml solution for injection 3ml pre-filled SoloStar pens (DE Pharmaceuticals) (product)
	38158011000001100	Onglyza 5mg tablets (DE Pharmaceuticals) (product)
	38174611000001100	Vipdomet 12.5mg/1000mg tablets (DE Pharmaceuticals) (product)
	38175211000001100	Vipidia 25mg tablets (DE Pharmaceuticals) (product)
	38190911000001100	Repaglinide 1mg tablets (Rivopharm (UK) Ltd) (product)
	38191211000001100	Repaglinide 2mg tablets (Rivopharm (UK) Ltd) (product)
	38293311000001100	Acarbose 100mg tablets (Rivopharm (UK) Ltd) (product)
	38530511000001100	Metformin 500mg modified-release tablets (Morningside Healthcare Ltd) (product)
	38543111000001100	Metformin 1g modified-release tablets (Morningside Healthcare Ltd) (product)
	1066911000000100	Diabetes monitoring short message service text message first invitation (procedure)
	1066921000000100	Diabetes monitoring short message service text message second invitation (procedure)
	185756006	Diabetes monitoring first letter (procedure)
	185757002	Diabetes monitoring second letter (procedure)
	185758007	Diabetes monitoring third letter (procedure)
	185759004	Diabetes monitoring verbal invite (procedure)
	310425007	Diabetes monitoring invitation (procedure)
	1066931000000100	Diabetes monitoring short message service text message third invitation (procedure)
	1083111000000100	Diabetes monitoring invitation email (procedure)
	1109921000000100	Quality and Outcomes Framework quality indicator-related care invitation (procedure)
	1110921000000100	Quality and Outcomes Framework diabetes mellitus quality indicator-related care invitation (procedure)
	143401000000102	Quality and Outcomes Framework diabetes mellitus quality indicator-related care invitation using preferred method of communication (procedure)
	185760009	Diabetes monitoring telephone invite (procedure)
	705072004	Diabetes monitoring invitation by short message service text messaging (procedure)

Supplementary Table 5: Codelists for laboratory and clinically confirmed COVID-19 (see https://github.com/BHFDSC/Linked-EHR-England-2021/tree/main/Phenotypes)

Code type	Code	Descriptions
**ICD10 codes**	**U07.1 or U071**	COVID-19 has been confirmed by laboratory testing irrespective of severity of clinical signs or symptoms
	**U07.2 or U072**	COVID-19 is diagnosed clinically or epidemiologically but laboratory testing is inconclusive or not available
SNOMED CONCEPT IDS	**1300721000000109**	Coronavirus disease 19 caused by severe acute respiratory syndrome coronavirus 2 confirmed by laboratory test (situation)
	**1240751000000100**	Coronavirus disease 19 caused by severe acute respiratory syndrome coronavirus 2 (disorder)
	**1240581000000104**	Severe acute respiratory syndrome coronavirus 2 ribonucleic acid detected (finding)
	**1008541000000105**	Coronavirus ribonucleic acid detection assay (observable entity)
	**186747009**	Coronavirus infection (disorder)
	**1300731000000106**	Coronavirus disease 19 caused by severe acute respiratory syndrome coronavirus 2 confirmed using clinical diagnostic criteria (situation)
	**1240511000000106**	Detection of severe acute respiratory syndrome coronavirus 2 using polymerase chain reaction technique (procedure)
	**1240551000000105**	Pneumonia caused by severe acute respiratory syndrome coronavirus 2 (disorder)
	**1240741000000103**	Severe acute respiratory syndrome coronavirus 2 serology (observable entity)
	**1300681000000102**	Assessment using coronavirus disease 19 severity scale (procedure)
	**1300631000000101**	Coronavirus disease 19 severity score (observable entity)
	**1321541000000108**	Severe acute respiratory syndrome coronavirus 2 immunoglobulin G detected (finding)
	**1240541000000107**	Infection of upper respiratory tract caused by severe acute respiratory syndrome coronavirus 2 (disorder)
	**1029481000000103**	Coronavirus nucleic acid detection assay (observable entity)
	**1300671000000104**	Coronavirus disease 19 severity scale (assessment scale)
	**1321551000000106**	Severe acute respiratory syndrome coronavirus 2 immunoglobulin M detected (finding)
	**1240401000000105**	Antibody to severe acute respiratory syndrome coronavirus 2 (substance)
	**1240421000000101**	Serotype severe acute respiratory syndrome coronavirus 2 (qualifier value)
	**1240571000000101**	Gastroenteritis caused by severe acute respiratory syndrome coronavirus 2 (disorder)
	**1321331000000107**	Arbitrary concentration of severe acute respiratory syndrome coronavirus 2 total immunoglobulin in serum (observable entity)
	**1321761000000103**	Severe acute respiratory syndrome coronavirus 2 immunoglobulin A detected (finding)
	**1322871000000109**	Severe acute respiratory syndrome coronavirus 2 antibody detection result positive (finding)
	**1240391000000107**	Antigen of severe acute respiratory syndrome coronavirus 2 (substance)
	**1240531000000103**	Myocarditis caused by severe acute respiratory syndrome coronavirus 2 (disorder)
	**1321341000000103**	Arbitrary concentration of severe acute respiratory syndrome coronavirus 2 immunoglobulin G in serum (observable entity)
	**1322781000000102**	Severe acute respiratory syndrome coronavirus 2 antigen detection result positive (finding)
	**1240521000000100**	Otitis media caused by severe acute respiratory syndrome coronavirus 2 (disorder)
	**1321321000000105**	Severe acute respiratory syndrome coronavirus 2 immunoglobulin G qualitative existence in specimen (observable entity)
	**1240561000000108**	Encephalopathy caused by severe acute respiratory syndrome coronavirus 2 (disorder)
	**1321241000000105**	Cardiomyopathy caused by severe acute respiratory syndrome coronavirus 2 (disorder)
	**1321201000000107**	Coronavirus disease 19 caused by severe acute respiratory syndrome coronavirus 2 health issues simple reference set (foundation metadata concept)
	**1321301000000101**	Severe acute respiratory syndrome coronavirus 2 ribonucleic acid qualitative existence in specimen (observable entity)
	**1321311000000104**	Severe acute respiratory syndrome coronavirus 2 immunoglobulin M qualitative existence in specimen (observable entity)
	**120814005**	Coronavirus antibody (substance)
	**1321181000000108**	Coronavirus disease 19 caused by severe acute respiratory syndrome coronavirus 2 record extraction simple reference set (foundation metadata concept)
	**1321351000000100**	Arbitrary concentration of severe acute respiratory syndrome coronavirus 2 immunoglobulin M in serum (observable entity)
	**1321191000000105**	Coronavirus disease 19 caused by severe acute respiratory syndrome coronavirus 2 procedures simple reference set (foundation metadata concept)

Supplementary Table 6: Dataset data dictionary

Dataset	Product	Field Group	Database Field Name	Description
**Deaths**	**Civil Registration - Deaths**	**Civil Registration - Deaths**	**-**	**The Deaths table contains a combination of the Civil Registration of Deaths data and the DARS BIRD DEATHS data. This has been made patient linkable via pseudonomised NHS Number**
Deaths	Civil Registration - Deaths	Civil Registration - Deaths	pseudo_hesid	This field uniquely identifies a patient across all data years. It is generated by matching records for the same patient using a combination of NHS Number, local patient identifier, provider code, postcode, sex and date of birth.<br>Customers who request an extract will receive a unique version of the PSEUDO_HESID called the EXTRACT_HESID.
Deaths	Civil Registration - Deaths	Civil Registration - Deaths	dod	Date on which the patient died. Where 'Death Record Used' = 'MIX1', 'HES1' or 'HES2', the date of death from HES (discharge date of death episode) is used.
Deaths	Civil Registration - Deaths	Civil Registration - Deaths	dor	Date on which the death was registered
Deaths	Civil Registration - Deaths	Civil Registration - Deaths	resstha	This field contains the code for the strategic health authority (SHA) in which the patient lived immediately before last admission before the date of death.
Deaths	Civil Registration - Deaths	Civil Registration - Deaths	respct	This field contains the code for the primary care trust (PCT) in which the patient lived immediately before the last admission before the date of death.
Deaths	Civil Registration - Deaths	Civil Registration - Deaths	sex	Sex of patient
Deaths	Civil Registration - Deaths	Civil Registration - Deaths	communal_establishment	The communal establishment code is a five-digit code derived from the place of death as supplied on the death certificate.
Deaths	Civil Registration - Deaths	Civil Registration - Deaths	nhs_indicator	Indicates whether the communal establishment code refers to an NHS establishment, referring to the physical building rather than the service.
Deaths	Civil Registration - Deaths	Civil Registration - Deaths	cause_of_death	This is an ICD* code that identifies the medical condition judged to be the underlying cause of death.
Deaths	Civil Registration - Deaths	Civil Registration - Deaths	death_record_used	Indicates source of the linked death record, HES or ONS
Deaths	Civil Registration - Deaths	Civil Registration - Deaths	subsequent_activity	Indicates whether the patient has had any HES activity after the date of death
Deaths	Civil Registration - Deaths	Civil Registration - Deaths	DEC_SEX	Sex
Deaths	Civil Registration - Deaths	Civil Registration - Deaths	REG_DATE_OF_DEATH	Date of death
Deaths	Civil Registration - Deaths	Civil Registration - Deaths	REG_DATE	Date of Registration
Deaths	Civil Registration - Deaths	Civil Registration - Deaths	REG_DISTRICT_CODE	Reg district
Deaths	Civil Registration - Deaths	Civil Registration - Deaths	REG_SUB_DISTRICT_CODE	Reg sub-district
Deaths	Civil Registration - Deaths	Civil Registration - Deaths	COR_AREA_NAME	Coroners area (Inquest)
Deaths	Civil Registration - Deaths	Civil Registration - Deaths	COR_INQ_DATES	Date of Inquest text
Deaths	Civil Registration - Deaths	Civil Registration - Deaths	DEC_OCC_TYPE	Occupation type code
Deaths	Civil Registration - Deaths	Civil Registration - Deaths	OUT_SECCATDM	National Statistics Socio-econonmic Classification operational category for deceased or mother of deceased juvenile
Deaths	Civil Registration - Deaths	Civil Registration - Deaths	OUT_SECCATHF	National Statistics Socio-econonmic Classification operational category for husband or father of deceased juvenile
Deaths	Civil Registration - Deaths	Civil Registration - Deaths	SOC2KDM	2010 Standard Ocuupation Classification of deceased or mother of deceased juvenile
Deaths	Civil Registration - Deaths	Civil Registration - Deaths	SOC2KHF	2010 Standard Ocuupation Classification of husband or father of deceased juvenile
Deaths	Civil Registration - Deaths	Civil Registration - Deaths	DEC_RETIRED_IND	Retired indicator for deceased or mother
Deaths	Civil Registration - Deaths	Civil Registration - Deaths	DEC_BIRTH_COUNTRY_CODE	Country code of place of birth of deceased
Deaths	Civil Registration - Deaths	Civil Registration - Deaths	POD_CODE	Place of death code
Deaths	Civil Registration - Deaths	Civil Registration - Deaths	POD_NHS_ESTABLISHMENT	NHS Establishment indicator
Deaths	Civil Registration - Deaths	Civil Registration - Deaths	POD_ESTABLISHMENT_TYPE	Establishment type where death occurred
Deaths	Civil Registration - Deaths	Civil Registration - Deaths	S_INJURY_EXTERNAL	Nature of injury code where the underlying cause of death (ICD10U) is an external cause
Deaths	Civil Registration - Deaths	Civil Registration - Deaths	NEO_NATE_FLAG	Indicates whether COD is neonatal (0 or 1)
Deaths	Civil Registration - Deaths	Civil Registration - Deaths	MED_C_OF_D_FREE_FORMAT	Free format cause of death
Deaths	Civil Registration - Deaths	Civil Registration - Deaths	REG_DISTRICT_NAME	Reg district name
Deaths	Civil Registration - Deaths	Civil Registration - Deaths	REG_SUB_DISTRICT_NAME	Reg sub-district name
Deaths	Civil Registration - Deaths	Civil Registration - Deaths	S_UNDERLYING_COD_ICD10	Primary cause of death, ICD10 Code
Deaths	Civil Registration - Deaths	Civil Registration - Deaths	S_UNDERLYING_COD_ICD9	Primary cause of death, ICD9 Code
Deaths	DARS BIRD DEATHS	All fields	CCGPOD	Clinical commissioning group code for place of death of deceased
Deaths	DARS BIRD DEATHS	All fields	CCGR	Clinical commissioning group code of usual residence of deceased
Deaths	DARS BIRD DEATHS	All fields	PCDIND	Postcode imputation indicator
Deaths	DARS BIRD DEATHS	All fields	CTYDR	County district code of usual residence of deceased
Deaths	DARS BIRD DEATHS	All fields	CTYR	County code of usual residence of deceased
Deaths	DARS BIRD DEATHS	All fields	GORR	Government Office Region code of usual residence of deceased
Deaths	DARS BIRD DEATHS	All fields	HAUTPOD	Primary Care Organisation code for place of death
Deaths	DARS BIRD DEATHS	All fields	HAUTR	PCT of residence
Deaths	DARS BIRD DEATHS	All fields	HROR	SHA of residence
Deaths	DARS BIRD DEATHS	All fields	LSOAR	Lower super output area of residence
Deaths	DARS BIRD DEATHS	All fields	UTLA9POD	Upper Tier Local Authority code for place of death
Deaths	DARS BIRD DEATHS	All fields	UTLA9R	Upper Tier Local Authority code of residence
Deaths	DARS BIRD DEATHS	All fields	WARDR	Ward code of usual residence of deceased
**GDPPR**	**GPES Data for Pandemic Planning and Research (COVID-19)**	**-**	**-**	**This is a GPES extract made for research during the COVID pandemic. Information can be found here: https://digital.nhs.uk/coronavirus/gpes-data-for-pandemic-planning-and-research/guide-for-analysts-and-users-of-the-data**
GDPPR	GPES Data for Pandemic Planning and Research (COVID-19)	General	GP_SYSTEM_SUPPLIER	Name of GP system supplier of the source patient record
GDPPR	GPES Data for Pandemic Planning and Research (COVID-19)	General	PROCESSED_TIMESTAMP	Date that the extract was processed
GDPPR	GPES Data for Pandemic Planning and Research (COVID-19)	General	REPORTING_PERIOD_END_DATE	Date that the extract was performed<br>on the source patient record
GDPPR	GPES Data for Pandemic Planning and Research (COVID-19)	Journal	CODE	SNOMED code value indicating the nature of the characteristic, event or intervention recorded
GDPPR	GPES Data for Pandemic Planning and Research (COVID-19)	Journal	DATE	Date to which journal item applies (For medication date of issue<br>(if EPISODE A or I) or authorisation (if EPISODE R))
GDPPR	GPES Data for Pandemic Planning and Research (COVID-19)	Journal	EPISODE_CONDITION	Whether first or subsequent episode of the coded condition<br>F First<br>N New<br>O Other<br>D Cause of Death
GDPPR	GPES Data for Pandemic Planning and Research (COVID-19)	Journal	EPISODE_PRESCRIPTION	Whether a prescription for the coded item is repeat or acute<br>A Acute (one-off issue)<br>I Issue of repeat<br>R Repeat authorisation
GDPPR	GPES Data for Pandemic Planning and Research (COVID-19)	Journal	LINKS	A list of zero, one or many link numbers.<br>A link number is shared by records for the same patient that have been explicitly linked in some way by the use. For example, problem-linkage, links between disorders and associated treatments and outcomes.
GDPPR	GPES Data for Pandemic Planning and Research (COVID-19)	Journal	PRACTICE	National practice code used to identify the GP practice
GDPPR	GPES Data for Pandemic Planning and Research (COVID-19)	Journal	RECORD_DATE	Date on which recorded<br>(For example, if an entry is made during a consultation<br>on 3-Jan-1998 noting a past history of appendectomy on 1-Nov-1978:<br>RECORD_DATE="1998-01-03", DATE="1978-11-01")
GDPPR	GPES Data for Pandemic Planning and Research (COVID-19)	Journal	VALUE1_CONDITION	First numeric value (in a record with CODE for investigation, measurement or result, e.g,<br>With CODE for weight VALUE1 is the weight in Kg,<br>with CODE for blood pressure VALUE1 is the systolic pressure in mmHg)
GDPPR	GPES Data for Pandemic Planning and Research (COVID-19)	Journal	VALUE1_PRESCRIPTION	Amount prescribed as number of tablets, capsules, etc. or volume of liquid in ml.
GDPPR	GPES Data for Pandemic Planning and Research (COVID-19)	Journal	VALUE2_CONDITION	Second numeric value (in a record with CODE for blood pressure e.g. diastolic pressure)
GDPPR	GPES Data for Pandemic Planning and Research (COVID-19)	Journal	VALUE2_PRESCRIPTION	Daily dose prescribed as number of tablets, capsules, etc. or volume of liquid in ml.
GDPPR	GPES Data for Pandemic Planning and Research (COVID-19)	Patient	ETHNIC	Ethnic origin (as per the code set within the NHS Data Dictionary)
GDPPR	GPES Data for Pandemic Planning and Research (COVID-19)	Patient	LSOA	The Lower Layer Super Output Area<br>(LSOA) derived from postcode and obtained<br>by lookup of an appropriate mapping resource e.g.<br>https://digital.nhs.uk/services/organisation-data-service/data-downloads/office-for-national-statistics-data)
GDPPR	GPES Data for Pandemic Planning and Research (COVID-19)	Patient	PRACTICE	National practice code used to identify the GP practice
GDPPR	GPES Data for Pandemic Planning and Research (COVID-19)	Patient	SEX	Sex of patient:<br>0 Not Known<br>1 Male<br>2 Female<br>9 Not specified
GDPPR	GPES Data for Pandemic Planning and Research (COVID-19)	Patient	YEAR_OF_DEATH	Year of death of patient
GDPPR	GPES Data for Pandemic Planning and Research (COVID-19)	Patient	YEAR_OF_BIRTH	Year of birth of patient

**HES AE**	**Hospital Episode Statistics Accident and Emergency**	**-**	**-**	**Hospital Episodes Statistics Accident and Emergency data. Derived from SUS and made person linkable on pseudonomised NHS number.**
HES AE	Hospital Episode Statistics Accident and Emergency	Attendances	AEARRIVALMODE	The mode by which a patient arrived at an A&E department.
HES AE	Hospital Episode Statistics Accident and Emergency	Attendances	AEATTEND_EXC_PLANNED	A "yes/no" flag, derived from AEATTENDCAT, enabling a count of unplanned attendances to be made.
HES AE	Hospital Episode Statistics Accident and Emergency	Attendances	AEATTENDCAT	An indication of whether a patient is making an initial or follow-up attendance within a particular A&E Department. The first attendance (1) is the first in a series, or the only attendance, in a particular A&E Episode. A planned follow-up A&E attendance (2) is a subsequent planned attendance at the same department, and for the same incident as the first attendance. An unplanned follow-up A&E attendance (3) is a subsequent unplanned attendance at the same department, and for the same incident as the first attendance.
HES AE	Hospital Episode Statistics Accident and Emergency	Attendances	AEATTENDDISP	The way in which an A&E attendance might end.
HES AE	Hospital Episode Statistics Accident and Emergency	Attendances	AEDEPTTYPE	A classification of A&E department type according to the activity carried out.
HES AE	Hospital Episode Statistics Accident and Emergency	Attendances	AEINCLOCTYPE	Classification of the place where the incident occurred that led to an A&E episode.
HES AE	Hospital Episode Statistics Accident and Emergency	Attendances	AEPATGROUP	A coded classification to identify the reason for an Accident and Emergency Episode.
HES AE	Hospital Episode Statistics Accident and Emergency	Attendances	AEREFSOURCE	The source of referral for each A&E episode.
HES AE	Hospital Episode Statistics Accident and Emergency	Attendances	ARRIVALDATE	The arrival date of a patient in the A&E department.
HES AE	Hospital Episode Statistics Accident and Emergency	Attendances	ARRIVALTIME	The time the patient self presented at the A&E Department or arrived in an ambulance at the A&E Department.
HES AE	Hospital Episode Statistics Accident and Emergency	Attendances	CONCLDUR	The time (expressed as a whole number of minutes) between the patient's arrival and conclusion of their attendance or treatment (whichever is later).
HES AE	Hospital Episode Statistics Accident and Emergency	Attendances	CONCLTIME	The time that a patient's A&E attendance ends or when treatment in A&E is completed (whichever is the later).
HES AE	Hospital Episode Statistics Accident and Emergency	Attendances	DEPDUR	The time (expressed as a whole number of minutes) between the patient's arrival, and the time the A&E attendance has concluded and the department is no longer responsible for the care of the patient.
HES AE	Hospital Episode Statistics Accident and Emergency	Attendances	DEPTIME	A&E departure time is the time that a patient leaves the A&E department after an A&E attendance has concluded and the department is no longer responsible for the care of the patient. Temporary absences from the A&E department are ignored. For example, during an A&E attendance the patient may leave the department for a short time for an X-ray, but they remain under the care of an A&E consultant. Note that A&E Attendance Conclusion time and A&E Departure times will be different for patients who wait for transport or who are lodged patients prior to admission to a ward.
HES AE	Hospital Episode Statistics Accident and Emergency	Attendances	INITDUR	The time (expressed as a whole number of minutes) between the patient's arrival and their initial assessment.
HES AE	Hospital Episode Statistics Accident and Emergency	Attendances	INITTIME	The time when a patient is assessed by medical or nursing staff in an A&E department to determine priority for treatment. The assessment should be conducted by medical or nursing staff who have received appropriate training in triage.
HES AE	Hospital Episode Statistics Accident and Emergency	Attendances	PARTYEAR	This field represents the year and month of the HES dataset in the format yyyymm. For example, 201601 represents the month 1 2016-17 HES dataset; 201602 represents the month 2 2016-17 HES dataset etc.
HES AE	Hospital Episode Statistics Accident and Emergency	Attendances	TRETDUR	The time (expressed as a whole number of minutes) between the patient's arrival (arrivaltime) and the start of their treatment (trettime).
HES AE	Hospital Episode Statistics Accident and Emergency	Attendances	TRETTIME	The time, recorded using a 24 hour clock, that the patient is seen by a health professional to diagnose the problem and arrange or start tests and start definite treatment as necessary.
HES AE	Hospital Episode Statistics Accident and Emergency	Clinical Diagnoses	DIAGSCHEME	The Coding Scheme basis of the Diagnosis.
HES AE	Hospital Episode Statistics Accident and Emergency	Attendances	FYEAR	Fiscal Year of data "yyYY" from 1st April yyyy to 31st March YYYY (i.e. for the April 2019 to March 2020 financial year, FYEAR is "1920")
HES AE	Hospital Episode Statistics Accident and Emergency	General fields	MATCH_RANK	User derived field - Not in core data
HES AE	Hospital Episode Statistics Accident and Emergency	All fields	INVESTSCHEME	INVESTSCHEME
HES AE	Hospital Episode Statistics Accident and Emergency	All fields	PROCSCHEME	The code scheme basis of a procedure.
HES AE	Hospital Episode Statistics Accident and Emergency	All fields	SUSLDDATE_HIS	SUSLDDATE_HIS
HES AE	Hospital Episode Statistics Accident and Emergency	All fields	TREATSCHEME	The clincial coding scheme used to describe the treatment
HES AE	Hospital Episode Statistics Accident and Emergency	Geographical	AT_GP_PRACTICE	This derived field contains the code for the Area Team (AT) where the patient's GP practice is registered. It is derived from Code of GP practice (GPPRAC).
HES AE	Hospital Episode Statistics Accident and Emergency	Geographical	AT_RESIDENCE	This derived field contains the code for the Area Team (AT) where the patient lives. It is derived from the CCG of residence.
HES AE	Hospital Episode Statistics Accident and Emergency	Geographical	AT_TREATMENT	This derived field contains the code for the Area Team (AT) where the patient was treated. It is derived from the CCG of Treatment.
HES AE	Hospital Episode Statistics Accident and Emergency	Geographical	CANNET	This field, which is derived from the patient's postcode in the field HOMEADD, contains a code which defines the former cancer network area that the postcode falls within.
HES AE	Hospital Episode Statistics Accident and Emergency	Geographical	CANREG	This field, which is derived from the patient's postcode in the field HOMEADD, contains a code which defines the cancer registry area that the postcode falls within.
HES AE	Hospital Episode Statistics Accident and Emergency	Geographical	CCG_GP_PRACTICE	This derived field contains the code for the Clinical Commissioning Group (CCG) where the patient's GP practice is registered. It is derived from GP Pracrice (GGPRAC).
HES AE	Hospital Episode Statistics Accident and Emergency	Geographical	CCG_RESIDENCE	This derived field contains the code for the Clinical Commissioning Group (CCG) where the patient lived immediately before admission. It is derived from post code (HOMEADD).
HES AE	Hospital Episode Statistics Accident and Emergency	Geographical	CCG_RESPONSIBILITY	This derived field contains the code for the most suitable Clinical Commissioning Group (CCG) of responsibility. It is derived firstly from the patient's GP practice but if not available the patient's CCG of residence then the CCG of treatment is used.
HES AE	Hospital Episode Statistics Accident and Emergency	Geographical	CCG_RESPONSIBILITY_ORIGIN	This derived field indicates the basis on which the CCG of Responsibility was assigned.
HES AE	Hospital Episode Statistics Accident and Emergency	Geographical	CCG_TREATMENT	This derived field contains the code for the Clinical Commissioning Group (CCG) where the patient was treated. It is derived from the postcode of the Site of Treatment firstly, but where not available the postcode of the Provider is used.
HES AE	Hospital Episode Statistics Accident and Emergency	Geographical	CCG_TREATMENT_ORIGIN	This derived field indicates the basis on which the CCG of Treatment was assigned.
HES AE	Hospital Episode Statistics Accident and Emergency	Geographical	CR_GP_PRACTICE	This derived field contains the code for the Commissioning Region (CR) where the patient's GP practice is registered. Where not available, the code for the Area Team is used. It is derived from gpprac.
HES AE	Hospital Episode Statistics Accident and Emergency	Geographical	CR_RESIDENCE	This derived field contains the code for the Commissioning Region (CR) where the patient lived immediately before admission. Where not available, the code for the Area Team is used. It is derived from the CCG of Residence.
HES AE	Hospital Episode Statistics Accident and Emergency	Geographical	CR_TREATMENT	This derived field contains the code for the Commissioning Region (CR) where the patient was treated. Where not available, the code for the Area Team is used. It is derived from the CCG of Treatment.
HES AE	Hospital Episode Statistics Accident and Emergency	Geographical	GORTREAT	Government Office Region (GOR) of treatment. This field is derived from the hospital provider code (procode). It indicates the GOR area within which the treatment took place.
HES AE	Hospital Episode Statistics Accident and Emergency	Geographical	LSOA01	This field, which is derived from the patient's postcode in the field HOMEADD, represents the 2001 Census Lower Layer SOA code for England and Wales, SOA code for Northern Ireland and data zone code for Scotland. Pseudo codes are included for Channel Islands and Isle of Man. The field will otherwise be blank for postcodes with no grid reference. The first character is either E for England or W for Wales The next two characters are 01 for Lower Super Output Area and the remaining six characters make up the unique 6-digit tag for each zone.
HES AE	Hospital Episode Statistics Accident and Emergency	Geographical	LSOA11	This field, which is derived from the patient's postcode in the field HOMEADD, represents the 2011 Census lower layer SOA code for England and Wales, SOA code for Northern Ireland and data zone code for Scotland. Pseudo codes are included for Channel Islands and Isle of Man. The field will otherwise be blank for postcodes with no grid reference. N.B. this field remains blank for Scottish postcodes until this information is released. The first character is either E for England or W for Wales The next two characters are 01 for Lower Super Output Area and the remaining six characters make up the unique 6-digit tag for each zone.
HES AE	Hospital Episode Statistics Accident and Emergency	Geographical	MSOA01	This field, which is derived from the patient's postcode in the field HOMEADD, represents the 2001 Census Middle Layer SOA (MSOA) code for England and Wales and intermediate zone for Scotland. Pseudo codes are included for Northern Ireland, Channel Islands and Isle of Man. The field will otherwise be blank for postcodes with no grid reference. The first character is either E for England or W for Wales. The next two characters are 02 for Middle Layer and the remaining six characters make up the unique 6-digit tag for each zone.

HES AE	Hospital Episode Statistics Accident and Emergency	Geographical	MSOA11	This field, which is derived from the patient's postcode in the field HOMEADD, represents the 2011 Census middle layer SOA (MSOA) code for England and Wales and intermediate zone for Scotland. Pseudo codes are included for Northern Ireland, Channel Islands and Isle of Man. The field will otherwise be blank for postcodes with no grid reference. N.B. this field remains blank for Scottish postcodes until this information is released. The first character is either E for England or W for Wales. The next two characters are 02 for Middle Layer and the remaining six characters make up the unique 6-digit tag for each zone.
HES AE	Hospital Episode Statistics Accident and Emergency	Geographical	PCON	For activity from 2011-12 onwards, HES users are advised to use the field PCON_ONS instead of PCON.<br><br>This field, which is derived from the patient's postcode in the field HOMEADD, defines the code for the English Westminster Parliamentary Constituency where the patient resides. Note that this field uses old-style geographical codes that were superseded in 2011 and are now no longer supported by ONS - the new-style code can be found in the field PCON_ONS.<br>This field will no longer be used from April 2021 onwards
HES AE	Hospital Episode Statistics Accident and Emergency	Geographical	PCON_ONS	This field, which is derived from the patient's postcode in the field HOMEADD, defines the code for the English Westminster Parliamentary Constituency where the patient resides.
HES AE	Hospital Episode Statistics Accident and Emergency	Geographical	PCTTREAT	This field is derived from the hospital provider code (procode). It indicates the PCT area of the main provider of treatment. Note that the PCT itself may be the provider of treatment.<br>Note: This field was formerly known as "Primary care trust area of treatment".<br><br>In 2013 following a reorganisation of health areas, Primary Care Trusts were abolished. This field has continued to be derived after this date as a 'frozen' geography.<br>This field will no longer be used from April 2021 onwards
HES AE	Hospital Episode Statistics Accident and Emergency	Geographical	RESCTY	For activity from 2011-12 onwards, HES users are advised to use the field RESCTY_ONS instead of RESCTY.<br><br>This field contains a code that defines the county of residence of the patient. It is derived from the patient's postcode in the field homeadd (postcode of patient).<br><br>Note that this field uses old-style geographical codes that were superseded in 2011 and are now no longer supported by ONS. The field RESCTY_ONS represents the county of residence using codes from the current ONS geographical coding system.<br><br>This field will no longer be used from April 2021 onwards
HES AE	Hospital Episode Statistics Accident and Emergency	Geographical	RESCTY_ONS	This field contains a code that defines the county of residence of the patient. It is derived from the patient's postcode in the field homeadd.
HES AE	Hospital Episode Statistics Accident and Emergency	Geographical	RESGOR	For activity from 2011-12 onwards, HES users are advised to use the field RESGOR_ONS instead of RESGOR<br><br>The Government Office Region of residence field contains a code that defines the Government Office Region of residence of the patient. It is derived from the patient's postcode in the field homeadd. The Government Office Regions closed on 31 March 2011, but this regional geography is maintained for statistical purposes and after this date referred to simply as 'regions'.<br><br>Note that this field uses old-style geographical codes that were superseded in 2011 and are now no longer supported by ONS. The field RESGOR_ONS represents the Government Office Region of residence using codes from the current ONS geographical coding system.<br>This field will no longer be used from April 2021 onwards
HES AE	Hospital Episode Statistics Accident and Emergency	Geographical	RESGOR_ONS	The Government Office Region of residence field contains a code that defines the Government Office Region of residence of the patient. It is derived from the patient's postcode in the field homeadd. The Government Office Regions closed on 31 March 2011, but this regional geography is maintained for statistical purposes and after this date referred to simply as 'regions'.
HES AE	Hospital Episode Statistics Accident and Emergency	Geographical	RESLADST	For activity from 2011-12 onwards, HES users are advised to use the field RESLADST_ONS instead of RESLADST.<br><br>This field contains a code that defines the county (first two characters) and local authority district (last two characters) of residence of the patient. It is derived from the patient's postcode in the field homeadd. Note that this field uses old-style geographical codes that were superseded in 2011 and are now no longer supported by ONS. The field RESLADST_ONS represents the local authority district of residence using codes from the current ONS geographical coding system.<br><br>RESLADST is used in conjunction with CURRWARD (current electoral ward) to produce a unique value indicating the ward within a given district where the patient resided (ie because identical CURRWARD codes are allocated to many local authority districts, CURRWARD is meaningless in isolation). If the patient is resident within a Unitary Authority, the first two characters will be 00 (zero, zero) and the local authority component may not be useable.<br>This field will no longer be used from April 2021 onwards
HES AE	Hospital Episode Statistics Accident and Emergency	Geographical	RESLADST_ONS	This derived field contains a code which defines the current local authority district of residence of the patient. It is derived from the patient's postcode in the field homeadd.
HES AE	Hospital Episode Statistics Accident and Emergency	Geographical	STHATRET	This field contains a code that defines the Strategic Health Authority (SHA) area where the treatment took place. It is derived from the hospital provider code in the field procode. Note: (1) for NHS trusts, care provided at subsidiary sites will be attributed to the main trust location.<br><br>SHAs were abolished in 2013. This field has continued to be derived after this date as a 'frozen' geography.<br><br>This field will no longer be used from April 2021 onwards
HES AE	Hospital Episode Statistics Accident and Emergency	Healthcare resource groups (HRG) data	SUSHRG	The SUS PbR derived healthcare resource group (HRG) code.
HES AE	Hospital Episode Statistics Accident and Emergency	Organisation	PCTCODE_HIS	The PCTCODE depending on the datayear. Where the data year is equal to or later than 2006, this field contains the code from the field PCTCODE06, otherwise it contains the code from the field PCTCODE02.<br><br>In 2013 following a reorganisation of health areas, Primary Care Trusts were abolished. This field has continued to be derived after this date as a 'frozen' geography.<br>This field will no longer be used from April 2021 onwards
HES AE	Hospital Episode Statistics Accident and Emergency	Organisation	PCTORIG_HIS	The origin of the primary care trust of responsibility dependent on the datayear. Where the data year is equal to or later than 2006, this field contains the code from the field PCTORIG06, otherwise it contains the code from the field PCTORIG02.<br>This field will no longer be used from April 2021 onwards
HES AE	Hospital Episode Statistics Accident and Emergency	Organisation	PROCODE	The organisation code of the organisation acting as the health care provider.<br><br>NHS organisations are allocated a main 3-character code, however this field may contain the 3-character organisation code or a full 5-character code (e.g. a site code, a HQ code ending 01 etc.). Independent providers will always be represented in this field by a 5-character code. Users wishing to group data at 3-character level (which will identify an individual organisation - e.g. NHS Trust) may wish to use the field PROCODE3.<br><br>Note that details of the site code can be found in the field 'site code of treatment' (SITETRET).

HES AE	Hospital Episode Statistics Accident and Emergency	Organisation	RESPCT_HIS	The Primary Care Trust (PCT) of residence dependent on the data year.<br><br>If the HES data year is 2006 or later, it will return the value from the field RESPCT06. If the data year is earlier than 2006, it will return the value from the field RESPCT02.<br><br>In 2013 following a reorganisation of health areas, Primary Care Trusts were abolished. This field has continued to be derived after this date as a 'frozen' geography.<br>This field will no longer be used from April 2021 onwards
HES AE	Hospital Episode Statistics Accident and Emergency	Organisation	RESSTHA_HIS	The Strategic Health Authority (SHA) of residence dependent on the data year.<br><br>If the HES data year is 2006 or later, it will return the value from the field RESSTHA06. If the data year is earlier than 2006, it will return the value from the field RESSTHA02.<br><br>In 2013 following a reorganisation of health areas, Strategic Health Authorities were abolished. This field has continued to be derived after this date as a 'frozen' geography.<br><br>This field will no longer be used from April 2021 onwards
HES AE	Hospital Episode Statistics Accident and Emergency	Organisation data	PROCODE3	The organisation code of the organisation acting as the health care provider. This field contains the first 3-characters of the provider code (PROCODE) field, which can be used to identify an individual provider (e.g. NHS Trust or PCT* (*before April 2013)).
HES AE	Hospital Episode Statistics Accident and Emergency	Organisation data	PROCODE5	The organisation code of the organisation acting as the health care provider. This field contains the provider code (PROCODE) to 5-characters. Where the field PROCODE contains a 3-character code, it will be padded to the right with additional zeros in this field.<br><br>Note that details of the site code can be found in the field 'site code of treatment' (SITETRET).
HES AE	Hospital Episode Statistics Accident and Emergency	Organisation data	PROCODET	This field gives a combination of 3-character and 5-character provider codes. Procodet enables you to view a combined list of codes, and related data, from:<br>1. Primary care trusts (3 character, beginning with 5)<br>2. NHS trusts (3 character, beginning with R or T. Trusts with associated treatment centres will have an '- X' following their code)<br>3. NHS trust treatment centres (5 character; listed separately to the NHS trusts)<br>4. Independent providers (5 character, beginning with 8)<br>5. Independent sector healthcare providers (5 character, beginning with N or A)<br>For 3-character codes only see procode (provider code (3 character)) and for 5-character codes only see procode5 (provider code (5 character)).
HES AE	Hospital Episode Statistics Accident and Emergency	Organisation data	PURCODE	This field contains a code for the organisation commissioning the patient's health care.
HES AE	Hospital Episode Statistics Accident and Emergency	Patient Data	ARRIVALAGE	This field is derived in HES as the patient's age in whole completed years, calculated from the arrival date (arrivaldate) and date of birth (dob).<br><br>For patients under 1 year old, special codes in the range 7001 to 7007 apply.
HES AE	Hospital Episode Statistics Accident and Emergency	Patient Data	ARRIVALAGE_CALC	Arrival age (ARRIVALAGE), with decimalised values for babies.
HES AE	Hospital Episode Statistics Accident and Emergency	Patient Data	ETHNOS	The ethnicity of the patient, as specified by the patient. This field uses the ethnic data categories as defined in the 2001 census.<br><br>Note that ETHNOS is very poorly populated on activity prior to the 2011-12 data year.
HES AE	Hospital Episode Statistics Accident and Emergency	Patient Data	MYDOB	Month and year of date of birth only. Day is not made available
HES AE	Hospital Episode Statistics Accident and Emergency	Patient Data	PSEUDO_HESID	This field contains a unique identifier for each individual patient. This allows an individual's care to be tracked across years and continuous periods to be identified. This is a pseudonymised version of the HES ID field based on an updated matching algorithm, which supersedes and is compatible with the original HES ID, which is no longer available.
HES AE	Hospital Episode Statistics Accident and Emergency	Patient Data	SEX	Defines the sex of the patient. The classification is phenotypical rather than genotypical, i.e. it does not provide codes for medical or scientific purposes.<br>Notes:<br>• National Code 'Not Known' means that the sex of a person has not been recorded<br>• National Code 'Not Specified' means indeterminate, i.e. unable to be classified as either male or female.
HES AE	Hospital Episode Statistics Accident and Emergency	Patient Pathway	WAITDAYS	This field is derived in HES to calculate the number of days that a patient waited in a referral to treatment period - this is the difference between the Referral to Treatment Start Date (RTTPERSTART) and Referral to Treatment End Date (RTTPEREND).
HES AE	Hospital Episode Statistics Accident and Emergency	Practitioner data	GPPRAC	This field contains a code which defines the practice that the patient is registered with.
HES AE	Hospital Episode Statistics Accident and Emergency	System Data	AEKEY	This is a record identifier that is created by the HES system. The digits store a decimal number. This is commonly eight or nine digits but can be up to 14.
HES AE	Hospital Episode Statistics Accident and Emergency	System Data	AEKEY_FLAG	Flags whether there is an AEKEY present on the record. This can be used to sum the number of A&E attendances.
HES AE	Hospital Episode Statistics Accident and Emergency	System Data	CDSEXTDATE	This is a system field that represents the date of the update event that resulted in the need to exchange the data with the Secondary Uses Service (SUS) database. This field is present on records submitted via the Bulk Update Protocol method only, and is used to apply records correctly to SUS.
HES AE	Hospital Episode Statistics Accident and Emergency	System Data	EPIKEY	This field is present on A&E and APC linked records only. Linkage between these two datasets enables patient pathways to be followed and provides additional information beyond what is available from the standalone datasets.<br><br>EPIKEY represents the record identifier of the APC episode that the A&E attendance has been linked to.<br><br>Full details of the methodology used to link A&E and APC records can be found on the following page of our website: http://content.digital.nhs.uk/article/1824/How-do-we-collect-and-process-HES-data
HES AE	Hospital Episode Statistics Accident and Emergency	System Data	SUBDATE	Date on which the data used to generate the HES record was received by the Secondary Uses Service.
HES AE	Hospital Episode Statistics Accident and Emergency	System Data	SUSLDDATE	The date that the record was loaded into the SUS staging system.
HES AE	Hospital Episode Statistics Accident and Emergency	System Data	SUSRECID	SUS (Secondary Uses Service)-generated record identifier.
HES AE	Hospital Episode Statistics Accident and Emergency	All fields	DIAG2_01	Primary AE diagnosis. The A&E diagnosis code recorded for an A&E attendance. The CDS allows an unlimited number of diagnoses to be submitted, however, only the first 12 diagnoses are available within HES. The A&E diagnosis is a six character code made up of, diagnosis condition (n2), sub-analysis (n1), anatomical area (n2) and anatomical side (an1). Only certain diagnoses contain a sub-analysis.<br><br>The A&E diagnosis description at 2-character level covering the diagnosis condition. This field contains a description based on the diagnosis condition (first 2 characters) of the A&E diagnosis and only displays a code where it is unclassifiable against the A&E Diagnosis classification.
HES AE	Hospital Episode Statistics Accident and Emergency	All fields	DIAG2_02 to 12	A secondary AE diagnosis. The A&E diagnosis code recorded for an A&E attendance. The CDS allows an unlimited number of diagnoses to be submitted, however, only the first 12 diagnoses are available within HES. The A&E diagnosis is a six character code made up of, diagnosis condition (n2), sub-analysis (n1), anatomical area (n2) and anatomical side (an1). Only certain diagnoses contain a sub-analysis.<br><br>The A&E diagnosis description at 2-character level covering the diagnosis condition. This field contains a description based on the diagnosis condition (first 2 characters) of the A&E diagnosis and only displays a code where it is unclassifiable against the A&E Diagnosis classification.

HES AE	Hospital Episode Statistics Accident and Emergency	All fields	DIAG3_01 to 12	A secondary AE diagnosis. The A&E diagnosis code recorded for an A&E attendance. The CDS allows an unlimited number of diagnoses to be submitted, however, only the first 12 diagnoses are available within HES. The A&E diagnosis is a six character code made up of, diagnosis condition (n2), sub-analysis (n1), anatomical area (n2) and anatomical side (an1). Only certain diagnoses contain a sub-analysis.<br><br>The A&E diagnosis description at 2-character level covering the diagnosis condition. This field contains a description based on the diagnosis condition (first 2 characters) of the A&E diagnosis and only displays a code where it is unclassifiable against the A&E Diagnosis classification.
HES AE	Hospital Episode Statistics Accident and Emergency	All fields	DIAGA_01 to 12	A secondary AE diagnosis. The A&E diagnosis code recorded for an A&E attendance. The CDS allows an unlimited number of diagnoses to be submitted, however, only the first 12 diagnoses are available within HES. The A&E diagnosis is a six character code made up of, diagnosis condition (n2), sub-analysis (n1), anatomical area (n2) and anatomical side (an1). Only certain diagnoses contain a sub-analysis.<br><br>The A&E diagnosis description at 2-character level covering the diagnosis condition. This field contains a description based on the diagnosis condition (first 2 characters) of the A&E diagnosis and only displays a code where it is unclassifiable against the A&E Diagnosis classification.
HES AE	Hospital Episode Statistics Accident and Emergency	All fields	DIAGS_01 to 12	A secondary AE diagnosis. The A&E diagnosis code recorded for an A&E attendance. The CDS allows an unlimited number of diagnoses to be submitted, however, only the first 12 diagnoses are available within HES. The A&E diagnosis is a six character code made up of, diagnosis condition (n2), sub-analysis (n1), anatomical area (n2) and anatomical side (an1). Only certain diagnoses contain a sub-analysis.<br><br>The A&E diagnosis description at 2-character level covering the diagnosis condition. This field contains a description based on the diagnosis condition (first 2 characters) of the A&E diagnosis and only displays a code where it is unclassifiable against the A&E Diagnosis classification.
HES AE	Hospital Episode Statistics Accident and Emergency	All fields	INVEST2_01 to 12	The A&E investigation recorded for an A&E attendance. The CDS allows an unlimited number of investigations to be submitted, however, only the first 12 investigations are available within HES. The A&E investigation is a six character code made up of, investigation (n2) and local sub-analysis (up to an4). As the sub-analysis is for local use it cannot be classified.<br><br>This field contains a description based upon the investigation (first 2 characters) of the A&E investigation code and only displays a code where it is unclassifiable against the A&E Investigation classification.
HES AE	Hospital Episode Statistics Accident and Emergency	All fields	NER_GP_PRACTICE	This derived field contains the code for the NHS England region where the patient's GP practice code is registered. It is derived from GPPRAC.
HES AE	Hospital Episode Statistics Accident and Emergency	All fields	NER_RESIDENCE	This derived field contains the code for the NHS England region where the patient lives.
HES AE	Hospital Episode Statistics Accident and Emergency	All fields	NER_TREATMENT	This derived field contains the code for the NHS England region where the patient was treated. It is derived from the CCG of treatment.
HES AE	Hospital Episode Statistics Accident and Emergency	All fields	PERSON_ID_DEID	Linkable patient identifer produced within NHS Digital by matching the HESID to MPS
HES AE	Hospital Episode Statistics Accident and Emergency	All fields	PROVDIST	Provides the distance in kilometers between the LSOA centroid and the site of treatment. Site of treatment is defined as PROCODE5 (if populated) or PROCODE3 (if PROCODE5 is not populated or the last two characters of PROCODE5 are 00).
HES AE	Hospital Episode Statistics Accident and Emergency	All fields	PROVDIST_FLAG	Flag to indicate if the PROVDIST field was calculated using PROCODE5 or PROCODE3 (if PROCODE5 was not populated or last two characters were 00).
HES AE	Hospital Episode Statistics Accident and Emergency	All fields	RANK_ORDER	This field is present on A&E and APC linked records only. Linkage between these two datasets enables patient pathways to be followed and provides additional information beyond what is available from the standalone datasets.<br><br>A score of one to four is applied to each linked record, with a score of one meaning a very good link and a score of four meaning a poor link. Records that score poor in quality, rating either three or four, are excluded from the final output of linked data available to users.<br><br>Full details of the methodology used to link A&E and APC records, and the filters used to assess linkage quality, can be found on the following page of our website: http://content.digital.nhs.uk/article/1824/How-do-we-collect-and-process-HES-data
HES AE	Hospital Episode Statistics Accident and Emergency	All fields	SITEDIST	Provides the distance in kilometers between the LSOA centroid and the site of treatment. Site of treatment is defined as first five characters of SITETRET or first 3 characters of SITETRET (if SITETRET is not populated with 5 characters)
HES AE	Hospital Episode Statistics Accident and Emergency	All fields	SITEDIST_FLAG	Flag to indicate if the SITETRET field had 3 or 5 characters to use in deriviation of SITEDIST
HES AE	Hospital Episode Statistics Accident and Emergency	All fields	SITETRET	This field contains a code that defines the site on which the patient was treated within an organisation. The code recorded should be the one of the Health Care Provider actually carrying out the work.<br>It contains the first 3 digit of the Provider Code with the last two digits being the site identifier.
HES AE	Hospital Episode Statistics Accident and Emergency	All fields	TREAT2_01 to 12	The first 2 characters of the A&E treatment code (TREAT_N) field, covering the treatment. The Commissioning Dataset allows an unlimited number of treatments to be submitted, however, only the first 12 treatments are available within HES. The A&E treatment is a six character code made up of: treatment (n2), sub-analysis (n1) and a local use section (up to an3). As the local use section is used for local codes it cannot be classified.<br><br>A truncated version of the code from this field representing the first 2 and 3 characters can be found in the fields TREAT2_N and TREAT3_N,
HES AE	Hospital Episode Statistics Accident and Emergency	All fields	TREAT3_01 to 12	The first 3 characters of the A&E treatment code (TREAT_N) field, covering the treatment and the sub-analysis. Note that if no sub-analysis has been provided, or is not applicable, then the 2-character description will be displayed if available. The Commissioning Dataset allows an unlimited number of treatments to be submitted, however, only the first 12 treatments are available within HES. The A&E treatment is a six character code made up of: treatment (n2), sub-analysis (n1) and a local use section (up to an3). As the local use section is used for local codes it cannot be classified.<br><br>A truncated version of the code from this field representing the first 2 and 3 characters can be found in the fields TREAT2_N and TREAT3_N,
**HES APC**	**Hospital Episode Statistics Admitted Patient Care**	-	-	**Hospital Episodes Statistics Admitted Patient Care data. Secondary Care. Derived from SUS and made person linkable on pseudonomised NHS number.**
HES APC	Hospital Episode Statistics Admitted Patient Care	Admissions ; Period of care	ADMIDATE	This field contains the date the patient was admitted to hospital at the start of a hospital spell. Admidate is recorded on all episodes within a spell.
HES APC	Hospital Episode Statistics Admitted Patient Care	Admissions ; Period of care	ADMIMETH	This field contains a code which identifies how the patient was admitted to hospital. Admimeth is recorded on the first and also all subsequent episodes within the spell (ie where the spell is made up of more than one episode).
HES APC	Hospital Episode Statistics Admitted Patient Care	Admissions ; Period of care	ADMISORC	This field contains a code which identifies where the patient was immediately prior to admission. Most patients are admitted from home, but there are some significant exceptions. In particular, this field differentiates between patients admitted from home and patients transferred from another hospital provider or institution.
HES APC	Hospital Episode Statistics Admitted Patient Care	Admissions ; Period of care	ELECDATE	This field contains the date on which a consultant, or another member of the clinical staff, decided to admit the patient to a hospital. The patient may or may not be admitted immediately. The time between elecdate and admidate (admission date) is known as the waiting time.
HES APC	Hospital Episode Statistics Admitted Patient Care	Admissions ; Period of care	ELECDUR	This submitted field contains the difference in days between the date on which it was decided to admit the patient (elecdate) and the actual admission date (admidate). Elecdur is only applicable where an elective admission (ie the admission method is 11, 12 or 13) was scheduled and took place.
HES APC	Hospital Episode Statistics Admitted Patient Care	Admissions ; Period of care	ELECDUR_CALC	This field returns the elecdurd but excludes non-elective and planned admissions - so only includes where the method of admission is 11 or 12. This is only calculated on the first episode in a hospital provider spell, and regular admissions are also excluded.<br><br>If classpat in ('1','2','5') and epiorder = 1 and admimeth in ('11','12')<br>then elecdur_calc = elecdurd

HES APC	Hospital Episode Statistics Admitted Patient Care	Clinical	CAUSE_3	This item is a copy of the initial 3 characters of the first diagnosis code that represents an external cause, eg accidents or poisoning.
HES APC	Hospital Episode Statistics Admitted Patient Care	Clinical	CAUSE_4	This item is a copy of the initial 4 characters of the first diagnosis code that represents an external cause, eg accidents or poisoning.
HES APC	Hospital Episode Statistics Admitted Patient Care	Clinical	DIAG_3_CONCAT	Provides a concatenated string of all diagnoses from the record at a 3 character level, separated by a comma with no spaces. This enables the user to search across the full list of diagnoses to look at mentions and pairs of diagnosis codes.
HES APC	Hospital Episode Statistics Admitted Patient Care	Clinical	Diag_3_01 to 12	This provides the first three characters of the diagnosis code. There are twelve fields (two before April 2007), diag_01 to diag_12, which contain information about a patient's illness or condition. The field diag_01 contains the primary diagnosis. The other fields contain secondary/subsidiary diagnoses. The codes are defined in the International Statistical Classification of Diseases, Injuries and Causes of Death. HES records currently use the tenth revision (ICD-10). Diagnosis codes start with a letter and are followed by two or three digits. The third digit identifies variations on a main diagnosis code containing two digits. The third digit is preceded by a full stop in ICD-10, but this is not stored in the field. This is the **PRIMARY** diagnosis.
HES APC	Hospital Episode Statistics Admitted Patient Care	Clinical	DIAG_4_CONCAT	Provides a concatenated string of all diagnoses from the record at a 4 character level, separated by a comma with no spaces. This enables the user to search across the full list of diagnoses to look at mentions and pairs of diagnosis codes.
HES APC	Hospital Episode Statistics Admitted Patient Care	Clinical	OPERTN_3_CONCAT	Provides a concatenated string of all procedures from the record at a 3 character level, separated by a comma with no spaces. This enables the user to search across the full list of procedures to look at mentions and pairs of procedure codes.
HES APC	Hospital Episode Statistics Admitted Patient Care	Clinical	OPERTN_3_01 to 24	The three character version of the OPERTN field. There are twenty-four fields (twelve before April 2007), oper_01 to oper_24, which contain information about a patient's operations. The field oper_01 contains the main (ie most resource intensive) procedure. The other fields contain secondary procedures. The codes are defined in the Tabular List of the Classification of Surgical Operations and Procedures. The current version is OPCS4. Procedure codes start with a letter and are followed by two or three digits. The third digit identifies variations on a main procedure code containing two digits. The third digit is preceded by a full stop in OPCS4, but this is not stored in the field. A single operation may contain more than one procedure.
HES APC	Hospital Episode Statistics Admitted Patient Care	Clinical	OPERTN_4_CONCAT	Provides a concatenated string of all procedures from the record at a 4 character level, separated by a comma with no spaces. This enables the user to search across the full list of procedures to look at mentions and pairs of procedure codes.
HES APC	Hospital Episode Statistics Admitted Patient Care	Clinical	OPERTN_COUNT	The total number of procedure codes present on the record (maximum of 24)
HES APC	Hospital Episode Statistics Admitted Patient Care	Clinical	Diag_4_01 to 12	This provides the first four characters of the diagnosis code. There are twelve fields (two before April 2007), diag_01 to diag_12, which contain information about a patient's illness or condition. The field diag_01 contains the primary diagnosis. The other fields contain secondary/subsidiary diagnoses. The codes are defined in the International Statistical Classification of Diseases, Injuries and Causes of Death. HES records currently use the tenth revision (ICD-10). Diagnosis codes start with a letter and are followed by two or three digits. The third digit identifies variations on a main diagnosis code containing two digits. The third digit is preceded by a full stop in ICD-10, but this is not stored in the field. This is the **PRIMARY** diagnosis.
HES APC	Hospital Episode Statistics Admitted Patient Care	Clinical	OPERTN_4_01 to 24	The four character version of the OPERTN field. There are twenty-four fields (twelve before April 2007), oper_01 to oper_24, which contain information about a patient's operations. The field oper_01 contains the main (ie most resource intensive) procedure. The other fields contain secondary procedures. The codes are defined in the Tabular List of the Classification of Surgical Operations and Procedures. The current version is OPCS4. Procedure codes start with a letter and are followed by two or three digits. The third digit identifies variations on a main procedure code containing two digits. The third digit is preceded by a full stop in OPCS4, but this is not stored in the field. A single operation may contain more than one procedure.
HES APC	Hospital Episode Statistics Admitted Patient Care	Clinical ; Period of care	CLASSPAT	This field identifies day cases, ordinary admissions, regular day and regular night attenders, and the special case of mothers and babies using only delivery facilities. Data about regular day and regular night attenders are not available for analysis prior to 2002-03. Since the introduction of the NHS wide clearing service in April 1996, this field has been derived from related items in the Commissioning Data Set (eg intended management).
HES APC	Hospital Episode Statistics Admitted Patient Care	Clinical ; Period of care	MAINSPEF	This field contains a code that defines the specialty under which the consultant is contracted. It can be compared with tretspef, the specialty under which the consultant worked.
HES APC	Hospital Episode Statistics Admitted Patient Care	Clinical ; Period of care	TRETSPEF	This field contains a code that defines the specialty in which the consultant was working during the period of care. It can be compared with mainspef, the specialty under which the consultant is contracted. Prior to 1 April 1996, this data item contained the code for the sub-specialty (subspef). From April 2004 a new list of treatment specialities was introduced (see below). The new list describes the specialised service within which the patient was treated.
HES APC	Hospital Episode Statistics Admitted Patient Care	Healthcare resource groups (HRG) data	HRG40	This derived field contains healthcare resource group (HRG) values. HES adds the two most recent versions of HRG codes to records. For example, a record for 2004-05 will have codes for HRG version 3.1 and HRG version 3.5.
HES APC	Hospital Episode Statistics Admitted Patient Care	Healthcare resource groups (HRG) data	HRGLATE35	This derived field contains healthcare resource group (HRG) values. HES adds the two most recent versions of HRG codes to records. For example, a record for 2004-05 will have codes for HRG version 3.1 and HRG version 3.5.
HES APC	Hospital Episode Statistics Admitted Patient Care	System Data	CDSVERSION	The version of the commissioning data set (CDS) being used.
HES APC	Hospital Episode Statistics Admitted Patient Care	Diagnosis	ALCNRWFRAC	Indicates the highest alcohol attributable fraction within an episode based on the narrow definition (primary diagnosis and external causes of injury) alcohol related diagnosis.
HES APC	Hospital Episode Statistics Admitted Patient Care	Diagnosis	ALCNRWDIAG	Provides the narrow (primary diagnosis and external causes of injury) alcohol related diagnosis.
HES APC	Hospital Episode Statistics Admitted Patient Care	Diagnosis	ALCBRDFRAC	Indicates the highest alcohol attributable fraction within an episode based on the broad definition (primary and secondary) alcohol related diagnosis.
HES APC	Hospital Episode Statistics Admitted Patient Care	Diagnosis	ALCBRDDIAG	Provides the broad definition (primary and secondary) alcohol related diagnosis.
HES APC	Hospital Episode Statistics Admitted Patient Care	Diagnosis	ALCDIAG_4	This provides the first four characters of the principle alcohol related diagnosis (ALCDIAG).
HES APC	Hospital Episode Statistics Admitted Patient Care	Diagnosis	ALCFRAC	Indicates the highest alcohol attributable fraction within the episode based on the principal alcohol related diagnosis. See "HES APC Data Dicitonary - Supplementary Table" at http://content.digital.nhs.uk/hesdatadictionary
HES APC	Hospital Episode Statistics Admitted Patient Care	Diagnosis	DIAG_COUNT	The total number of diagnosis codes present on the record (maximum of 20)
HES APC	Hospital Episode Statistics Admitted Patient Care	Discharges ; Period of care	DISDATE	This field contains the date on which the patient was discharged from hospital. ; It is only present in the record for the last episode of a spell.
HES APC	Hospital Episode Statistics Admitted Patient Care	Discharges ; Period of care	DISDEST	This field contains a code which identifies where the patient was due to go on leaving hospital. In most cases they return home. For many patients discharge destination is the same as source of admission (admisorc).
HES APC	Hospital Episode Statistics Admitted Patient Care	Discharges ; Period of care	DISMETH	This field contains a code which defines the circumstances under which a patient left hospital. For the majority of patients this is when they are discharged by the consultant. This field is only completed for the last episode in a spell.
HES APC	Hospital Episode Statistics Admitted Patient Care	Episodes and spells ; Period of care	EPIDUR	This field contains the difference in days between the episode start date (epistart) and the episode end date (epiend).<br><br>This is the full duration of a finished episode only. Episode duration is not applicable for 'other maternity events' and shall therefore remain null.
HES APC	Hospital Episode Statistics Admitted Patient Care	Episodes and spells ; Period of care	EPIEND	This field contains the date on which a patient left the care of a particular consultant, for one of the following reasons: Patient discharged from hospital (includes transfers) or moved to the care of another consultant. A null entry either indicates that the episode was unfinished at the end of the data year, or the date was unknown.
HES APC	Hospital Episode Statistics Admitted Patient Care	Episodes and spells ; Period of care	EPIORDER	This field contains the number of the episode within the current spell. All spells start with an episode where epiorder is 01. Many spells finish with this episode, but if the patient moves to the care of another consultant, a new episode begins. Episode numbers increase by 1 for each new episode until the patient is discharged (this includes transfers to another NHS trust or primary care trust - ie the first episode in the new trust will have epiorder 01). If the same patient returns for a different spell in hospital, epiorder is again set to 01. Admissions are calculated by counting the number of times epiorder is 01. When studying long term care, remember that it is not unusual to transfer psychiatric patients from one hospital to another.

HES APC	Hospital Episode Statistics Admitted Patient Care	Episodes and spells ; Period of care	EPISTART	This field contains the date on which a patient was under the care of a particular consultant. If a patient has more than one episode in a spell, for each new episode there is a new value of epistart. However, the admission date which is copied to each new episode in a spell will remain unchanged and will be equal to the episode start date of the first episode in hospital.
HES APC	Hospital Episode Statistics Admitted Patient Care	Episodes and spells ; Period of care	EPISTAT	This field tells you whether the episode had finished before the end of the HES data-year (ie whether the episode was still 'live' at midnight on 31 March). For example, if a patient was admitted on 25 March 2005 and was not discharged (or transferred to the care of another consultant) until 4 April 2005, there will be a record describing the unfinished episode (episode status = 1) in the 2004-05 data, and a separate record describing the finished episode (episode status = 3) in the 2005-06 data. Because hospital providers are advised not to include clinical data (diagnosis and operation codes) in unfinished records, these are normally excluded from analyses. Also, if unfinished episodes are included in time series analyses - where data for more than one year is involved - there is a danger of counting the same episode twice.
HES APC	Hospital Episode Statistics Admitted Patient Care	Episodes and spells ; Period of care	EPITYPE	This field contains a code that defines the type of episode, so that groups of similar episodes can be formed.
HES APC	Hospital Episode Statistics Admitted Patient Care	Episodes and spells ; Period of care	FAE	This indicates whether the episode is a finished admission episode (the first episode in a hospital provider spell). FAE is derived in HES, and is set to a value of 1 for finished admission episodes (where the Episode Status is 3 and Episode Order = 1 and Patient Classification is 1, 2 or 5).
HES APC	Hospital Episode Statistics Admitted Patient Care	Episodes and spells ; Period of care	FAE_EMERGENCY	Finished Admission Episode Flag where admission to hospital is from an emergency admission. This is set to a value of 1 for the admission episode where Patient Classification is 1,2 or 5 AND admission method begins with a 2*
HES APC	Hospital Episode Statistics Admitted Patient Care	Episodes and spells ; Period of care	FCE	Finished consultant episode (FCE) is a continuous period of admitted patient care under one consultant within one healthcare provider. FCEs do not represent the number of in-patients, as a person may have more than one period of care within the year. This field is derived in HES and it is set to a value of 1 for finished episodes i.e. where the Episode Status is 3 and the Patient Classification is 1, 2 or 5 (Ordinary admission, day case admission or mothers and babies using only delivery facilities).
HES APC	Hospital Episode Statistics Admitted Patient Care	Episodes and spells ; Period of care	FDE	This indicates whether the episode is a finished discharge episode (whether the patient was discharged at the end of the episode). FDE is derived in HES, and is set to a value of 1 for finished discharge episodes (where the Episode Status is 3 and Discharge Method is 1-5 and Patient Classification is 1, 2 or 5,).
HES APC	Hospital Episode Statistics Admitted Patient Care	Episodes and spells ; Period of care	FYEAR	Financial year of the HES data i.e. 2021 - 2020 - 2021
HES APC	Hospital Episode Statistics Admitted Patient Care	Episodes and spells ; Period of care	PARTYEAR	This field represents the year and month of the HES dataset in the format yyyymm. For example, 201601 represents the month 1 2016-17 HES dataset; 201602 represents the month 2 2016-17 HES dataset etc.
HES APC	Hospital Episode Statistics Admitted Patient Care	Episodes and spells ; Period of care	SPELBGIN	This derived field contains a code that defines whether the episode is the first of a spell, and whether the spell started in the current year or a previous year. Other maternity events are excluded.
HES APC	Hospital Episode Statistics Admitted Patient Care	Episodes and spells ; Period of care	SPELDUR	This derived field gives the duration of the spell in days. It contains the difference in days between the admission date (admidate) and the discharge date (epiend) provided the discharge method (dismeth) confirms that the spell has finished. If the episode has not finished it is calculated from the end of the year and admidate.
HES APC	Hospital Episode Statistics Admitted Patient Care	Episodes and spells ; Period of care	SPELEND	This derived field contains a code that defines whether the episode is the last of a spell. It is set on finished general, delivery and birth episodes (where epistat = 3 and epitype = 1, 2 or 3), provided the discharge method (dismeth) confirms that the spell has finished.
HES APC	Hospital Episode Statistics Admitted Patient Care	Geographical	AT_GP_PRACTICE	This derived field contains the code for the Area Team (AT) where the patient’s GP practice is registered. It is derived from Code of GP practice (GPPRAC).<br><br>In 2015 following an NHS reorganisation, Area Teams were replaced by NHS England regions. This field has continued to be derived after this date as a ‘frozen’ geography. The NHS England region of the GP practice can be found in the field NER_GP_PRACTICE. This field will no longer be used from April 2021 onwards
HES APC	Hospital Episode Statistics Admitted Patient Care	Geographical	AT_RESIDENCE	This derived field contains the code for the Area Team (AT) where the patient lives. It is derived from the CCG of residence.<br><br>In 2015 following an NHS reorganisation, Area Teams were replaced by NHS England regions. This field has continued to be derived after this date as a ‘frozen’ geography. The NHS Region of residence can be found in the field NER_RESIDENCE. This field will no longer be used from April 2021 onwards
HES APC	Hospital Episode Statistics Admitted Patient Care	Geographical	AT_TREATMENT	This derived field contains the code for the Area Team (AT) where the patient was treated. It is derived from the CCG of Treatment.<br><br>In 2015 following an NHS reorganisation, Area Teams were replaced by NHS England regions. This field has continued to be derived after this date as a ‘frozen’ geography. The NHS England region of treatment can be found in the field NER_TREATMENT. This field will no longer be used from April 2021 onwards
HES APC	Hospital Episode Statistics Admitted Patient Care	Geographical	CANNET	This field, which is derived from the patient's postcode in the field HOMEADD, contains a code which defines the former cancer network area that the postcode falls within.<br><br>Cancer networks were abolished in 2013. This field has continued to be derived after this date as a 'frozen' geography. This field will no longer be used from April 2021 onwards
HES APC	Hospital Episode Statistics Admitted Patient Care	Geographical	CANREG	This field, which is derived from the patient's postcode in the field HOMEADD, contains a code which defines the cancer registry area that the postcode falls within.
HES APC	Hospital Episode Statistics Admitted Patient Care	Geographical	CCG_GP_PRACTICE	This derived field contains the code for the Clinical Commissioning Group (CCG) where the patient’s GP practice is registered. It is derived from GP Pracrice (GGPRAC).
HES APC	Hospital Episode Statistics Admitted Patient Care	Geographical	CCG_RESIDENCE	This derived field contains the code for the Clinical Commissioning Group (CCG) where the patient lived immediately before admission. It is derived from post code (HOMEADD).
HES APC	Hospital Episode Statistics Admitted Patient Care	Geographical	CCG_RESPONSIBILITY	This derived field contains the code for the most suitable Clinical Commissioning Group (CCG) of responsibility. It is derived firstly from the patient’s GP practice but if not available the patient’s CCG of residence then the CCG of treatment is used.
HES APC	Hospital Episode Statistics Admitted Patient Care	Geographical	CCG_RESPONSIBILITY_ORIGIN	This derived field indicates the basis on which the CCG of Responsibility was assigned.
HES APC	Hospital Episode Statistics Admitted Patient Care	Geographical	CCG_TREATMENT	This derived field contains the code for the Clinical Commissioning Group (CCG) where the patient was treated. It is derived from the postcode of the Site of Treatment firstly, but where not available the postcode of the Provider is used.
HES APC	Hospital Episode Statistics Admitted Patient Care	Geographical	CCG_TREATMENT_ORIGIN	This derived field indicates the basis on which the CCG of Treatment was assigned.
HES APC	Hospital Episode Statistics Admitted Patient Care	Geographical	CR_GP_PRACTICE	This derived field contains the code for the Commissioning Region (CR) where the patient’s GP practice is registered. Where not available, the code for the Area Team is used. It is derived from gpprac.
HES APC	Hospital Episode Statistics Admitted Patient Care	Geographical	CR_RESIDENCE	This derived field contains the code for the Commissioning Region (CR) where the patient lived immediately before admission. Where not available, the code for the Area Team is used. It is derived from the CCG of Residence.
HES APC	Hospital Episode Statistics Admitted Patient Care	Geographical	CR_TREATMENT	This derived field contains the code for the Commissioning Region (CR) where the patient was treated. Where not available, the code for the Area Team is used. It is derived from the CCG of Treatment.
HES APC	Hospital Episode Statistics Admitted Patient Care	Geographical	GORTREAT	Government Office Region (GOR) of treatment. This field is derived from the hospital provider code (procode). It indicates the GOR area within which the treatment took place.
HES APC	Hospital Episode Statistics Admitted Patient Care	Geographical	LSOA01	This field, which is derived from the patient's postcode in the field HOMEADD, represents the 2001 Census Lower Layer SOA code for England and Wales, SOA code for Northern Ireland and data zone code for Scotland. Pseudo codes are included for Channel Islands and Isle of Man. The field will otherwise be blank for postcodes with no grid reference. The first character is either E for England or W for Wales The next two characters are 01 for Lower Super Output Area and the remaining six characters make up the unique 6-digit tag for each zone.

HES APC	Hospital Episode Statistics Admitted Patient Care	Geographical	LSOA11	This field, which is derived from the patient's postcode in the field HOMEADD, represents the 2011 Census lower layer SOA code for England and Wales, SOA code for Northern Ireland and data zone code for Scotland. Pseudo codes are included for Channel Islands and Isle of Man. The field will otherwise be blank for postcodes with no grid reference. N.B. this field remains blank for Scottish postcodes until this information is released. The first character is either E for England or W for Wales The next two characters are 01 for Lower Super Output Area and the remaining six characters make up the unique 6-digit tag for each zone.
HES APC	Hospital Episode Statistics Admitted Patient Care	Geographical	MSOA01	This field, which is derived from the patient's postcode in the field HOMEADD, represents the 2001 Census Middle Layer SOA (MSOA) code for England and Wales and intermediate zone for Scotland. Pseudo codes are included for Northern Ireland, Channel Islands and Isle of Man. The field will otherwise be blank for postcodes with no grid reference. The first character is either E for England or W for Wales. The next two characters are 02 for Middle Layer and the remaining six characters make up the unique 6-digit tag for each zone.
HES APC	Hospital Episode Statistics Admitted Patient Care	Geographical	MSOA11	This field, which is derived from the patient's postcode in the field HOMEADD, represents the 2011 Census middle layer SOA (MSOA) code for England and Wales and intermediate zone for Scotland. Pseudo codes are included for Northern Ireland, Channel Islands and Isle of Man. The field will otherwise be blank for postcodes with no grid reference. N.B. this field remains blank for Scottish postcodes until this information is released. The first character is either E for England or W for Wales. The next two characters are 02 for Middle Layer and the remaining six characters make up the unique 6-digit tag for each zone.
HES APC	Hospital Episode Statistics Admitted Patient Care	Geographical	PCON	For activity from 2011-12 onwards, HES users are advised to use the field PCON_ONS instead of PCON.<br><br>This field, which is derived from the patient's postcode in the field HOMEADD, defines the code for the English Westminster Parliamentary Constituency where the patient resides. Note that this field uses old-style geographical codes that were superseded in 2011 and are now no longer supported by ONS - the new-style code can be found in the field PCON_ONS.<br>This field will no longer be used from April 2021 onwards
HES APC	Hospital Episode Statistics Admitted Patient Care	Geographical	PCON_ONS	This field, which is derived from the patient's postcode in the field HOMEADD, defines the code for the English Westminster Parliamentary Constituency where the patient resides.
HES APC	Hospital Episode Statistics Admitted Patient Care	Geographical	PCTTREAT	This field is derived from the hospital provider code (procode). It indicates the PCT area of the main provider of treatment. Note that the PCT itself may be the provider of treatment.<br>Note: This field was formerly known as "Primary care trust area of treatment".<br><br>In 2013 following a reorganisation of health areas, Primary Care Trusts were abolished. This field has continued to be derived after this date as a 'frozen' geography.<br>This field will no longer be used from April 2021 onwards
HES APC	Hospital Episode Statistics Admitted Patient Care	Geographical	RESCTY	For activity from 2011-12 onwards, HES users are advised to use the field RESCTY_ONS instead of RESCTY.<br><br>This field contains a code that defines the county of residence of the patient. It is derived from the patient's postcode in the field homeadd (postcode of patient).<br><br>Note that this field uses old-style geographical codes that were superseded in 2011 and are now no longer supported by ONS. The field RESCTY_ONS represents the county of residence using codes from the current ONS geographical coding system.<br><br>This field will no longer be used from April 2021 onwards
HES APC	Hospital Episode Statistics Admitted Patient Care	Geographical	RESCTY_ONS	This field contains a code that defines the county of residence of the patient. It is derived from the patient's postcode in the field homeadd.
HES APC	Hospital Episode Statistics Admitted Patient Care	Geographical	RESGOR	For activity from 2011-12 onwards, HES users are advised to use the field RESGOR_ONS instead of RESGOR<br><br>The Government Office Region of residence field contains a code that defines the Government Office Region of residence of the patient. It is derived from the patient's postcode in the field homeadd. The Government Office Regions closed on 31 March 2011, but this regional geography is maintained for statistical purposes and after this date referred to simply as 'regions'.<br><br>Note that this field uses old-style geographical codes that were superseded in 2011 and are now no longer supported by ONS. The field RESGOR_ONS represents the Government Office Region of residence using codes from the current ONS geographical coding system.<br>This field will no longer be used from April 2021 onwards
HES APC	Hospital Episode Statistics Admitted Patient Care	Geographical	RESGOR_ONS	The Government Office Region of residence field contains a code that defines the Government Office Region of residence of the patient. It is derived from the patient's postcode in the field homeadd. The Government Office Regions closed on 31 March 2011, but this regional geography is maintained for statistical purposes and after this date referred to simply as 'regions'.
HES APC	Hospital Episode Statistics Admitted Patient Care	Geographical	RESLADST	For activity from 2011-12 onwards, HES users are advised to use the field RESLADST_ONS instead of RESLADST.<br><br>This field contains a code that defines the county (first two characters) and local authority district (last two characters) of residence of the patient. It is derived from the patient's postcode in the field homeadd. Note that this field uses old-style geographical codes that were superseded in 2011 and are now no longer supported by ONS. The field RESLADST_ONS represents the local authority district of residence using codes from the current ONS geographical coding system.<br><br>RESLADST is used in conjunction with CURRWARD (current electoral ward) to produce a unique value indicating the ward within a given district where the patient resided (ie because identical CURRWARD codes are allocated to many local authority districts, CURRWARD is meaningless in isolation). If the patient is resident within a Unitary Authority, the first two characters will be 00 (zero, zero) and the local authority component may not be useable.<br>This field will no longer be used from April 2021 onwards
HES APC	Hospital Episode Statistics Admitted Patient Care	Geographical	RESLADST_ONS	This derived field contains a code which defines the current local authority district of residence of the patient. It is derived from the patient's postcode in the field homeadd.
HES APC	Hospital Episode Statistics Admitted Patient Care	Geographical	RESPCT_HIS	The Primary Care Trust (PCT) of residence dependent on the data year.<br><br>If the HES data year is 2006 or later, it will return the value from the field RESPCT06. If the data year is earlier than 2006, it will return the value from the field RESPCT02.<br><br>In 2013 following a reorganisation of health areas, Primary Care Trusts were abolished. This field has continued to be derived after this date as a 'frozen' geography.<br>This field will no longer be used from April 2021 onwards
HES APC	Hospital Episode Statistics Admitted Patient Care	Geographical	RESSTHA	This derived field contains the code for the strategic health authority (SHA) in which the patient lived immediately before admission. It is derived from the patient's postcode in the field homeadd.<br><br>In 2013 following a reorganisation of health areas, Strategic Health Authorities were abolished. This field has continued to be derived after this date as a 'frozen' geography.
HES APC	Hospital Episode Statistics Admitted Patient Care	Geographical	SITETRET	This field contains a code that defines the site on which the patient was treated within an organisation. The code recorded should be the one of the Health Care Provider actually carrying out the work.<br>It contains the first 3 digit of the Provider Code with the last two digits being the site identifier.

HES APC	Hospital Episode Statistics Admitted Patient Care	Geographical	STHATRET	This field contains a code that defines the Strategic Health Authority (SHA) area where the treatment took place. It is derived from the hospital provider code in the field procode. Note: (1) for NHS trusts, care provided at subsidiary sites will be attributed to the main trust location.<br><br>SHAs were abolished in 2013. This field has continued to be derived after this date as a 'frozen' geography.<br><br>This field will no longer be used from April 2021 onwards
HES APC	Hospital Episode Statistics Admitted Patient Care	Healthcare resource groups (HRG) data	SUSHRG	The SUS PbR derived healthcare resource group (HRG) code.
HES APC	Hospital Episode Statistics Admitted Patient Care	Maternity	NEODUR	This field contains the age in days of a baby admitted as a patient. It is derived from admission date (admidate) and date of birth (dob). If the baby is older than 27 days, neodur is not calculated.
HES APC	Hospital Episode Statistics Admitted Patient Care	Organisation	PROCODE3	The organisation code of the organisation acting as the health care provider. This field contains the first 3-characters of the provider code (PROCODE) field, which can be used to identify an individual provider (e.g. NHS Trust or PCT* (*before April 2013)).
HES APC	Hospital Episode Statistics Admitted Patient Care	Organisation	PROCODE5	The organisation code of the organisation acting as the health care provider. This field contains the provider code (PROCODE) to 5-characters. Where the field PROCODE contains a 3-character code, it will be padded to the right with additional zeros in this field.<br><br>Note that details of the site code can be found in the field 'site code of treatment' (SITETRET).
HES APC	Hospital Episode Statistics Admitted Patient Care	Organisation	PROCODET	This field gives a combination of 3-character and 5-character provider codes. Procodet enables you to view a combined list of codes, and related data, from:<br>1. Primary care trusts (3 character, beginning with 5)<br>2. NHS trusts (3 character, beginning with R or T. Trusts with associated treatment centres will have an '- X' following their code)<br>3. NHS trust treatment centres (5 character; listed separately to the NHS trusts)<br>4. Independent providers (5 character, beginning with 8)<br>5. Independent sector healthcare providers (5 character, beginning with N or A)<br>For 3-character codes only see procode (provider code (3 character)) and for 5-character codes only see procode5 (provider code (5 character)).
HES APC	Hospital Episode Statistics Admitted Patient Care	Organisation	PURCODE	This field contains a code for the organisation commissioning the patient's health care.
HES APC	Hospital Episode Statistics Admitted Patient Care	Organisation	PROCODE	The organisation code of the organisation acting as the health care provider.<br><br>NHS organisations are allocated a main 3-character code, however this field may contain the 3-character organisation code or a full 5-character code (e.g. a site code, a HQ code ending 01 etc.). Independent providers will always be represented in this field by a 5-character code. Users wishing to group data at 3-character level (which will identify an individual organisation - e.g. NHS Trust) may wish to use the field PROCODE3.<br><br>Note that details of the site code can be found in the field 'site code of treatment' (SITETRET).
HES APC	Hospital Episode Statistics Admitted Patient Care	Organisation	PCTCODE_HIS	The PCTCODE depending on the datayear. Where the data year is equal to or later than 2006, this field contains the code from the field PCTCODE06, otherwise it contains the code from the field PCTCODE02.<br><br>In 2013 following a reorganisation of health areas, Primary Care Trusts were abolished. This field has continued to be derived after this date as a 'frozen' geography.<br>This field will no longer be used from April 2021 onwards
HES APC	Hospital Episode Statistics Admitted Patient Care	Organisation	PCTORIG_HIS	The origin of the primary care trust of responsibility dependent on the datayear. Where the data year is equal to or later than 2006, this field contains the code from the field PCTORIG06, otherwise it contains the code from the field PCTORIG02.<br>This field will no longer be used from April 2021 onwards
HES APC	Hospital Episode Statistics Admitted Patient Care	Organisation	RESSTHA_HIS	The Strategic Health Authority (SHA) of residence dependent on the data year.<br><br>If the HES data year is 2006 or later, it will return the value from the field RESSTHA06. If the data year is earlier than 2006, it will return the value from the field RESSTHA02.<br><br>In 2013 following a reorganisation of health areas, Strategic Health Authorities were abolished. This field has continued to be derived after this date as a 'frozen' geography.<br><br>This field will no longer be used from April 2021 onwards
HES APC	Hospital Episode Statistics Admitted Patient Care	Patient Data	ADMINCAT	Administrative category on admission.
HES APC	Hospital Episode Statistics Admitted Patient Care	Patient Data	ADMINCATST	The patient's administrative category at the start of each episode of care. This may change during a spell as the patient may, for example, opt to change from NHS to private health care.
HES APC	Hospital Episode Statistics Admitted Patient Care	Patient Data	ETHNOS	The ethnicity of the patient, as specified by the patient. This field uses the ethnic data categories as defined in the 2001 census.<br><br>Note that ETHNOS is very poorly populated on activity prior to the 2011-12 data year.
HES APC	Hospital Episode Statistics Admitted Patient Care	Patient Data	MYDOB	Month and year of date of birth only. Day is not made available
HES APC	Hospital Episode Statistics Admitted Patient Care	Patient Data	SEX	Defines the sex of the patient. The classification is phenotypical rather than genotypical, i.e. it does not provide codes for medical or scientific purposes.<br>Notes:<br>• National Code 'Not Known' means that the sex of a person has not been recorded<br>• National Code 'Not Specified' means indeterminate, i.e. unable to be classified as either male or female.
HES APC	Hospital Episode Statistics Admitted Patient Care	Patient Data	STARTAGE	This derived field, calculated from episode start date (epistart) and date of birth (dob), contains the patient's age in whole years (From 1 to 115 (1990-91 to 1994-95) and from 1 to 120 (1995-96 onwards)). For patients under 1 year old, special codes in the range 7001 to 7007 apply.
HES APC	Hospital Episode Statistics Admitted Patient Care	Patient Data	STARTAGE_CALC	Age at start of the episode (STARTAGE), with decimalised values for babies.
HES APC	Hospital Episode Statistics Admitted Patient Care	Patient Pathway	WAITDAYS	This field is derived in HES to calculate the number of days that a patient waited in a referral to treatment period - this is the difference between the Referral to Treatment Start Date (RTTPERSTART) and Referral to Treatment End Date (RTTPEREND).
HES APC	Hospital Episode Statistics Admitted Patient Care	Patient Pathway	WAITLIST	Calculation determining patients whose method of admission was from the waiting list
HES APC	Hospital Episode Statistics Admitted Patient Care	Practitioner	GPPRAC	This field contains a code which defines the practice that the patient is registered with.
HES APC	Hospital Episode Statistics Admitted Patient Care	Practitioner	REFERORG	The organisation code of the organisation from which the referral is made, such as GP practice or NHS trust.
HES APC	Hospital Episode Statistics Admitted Patient Care	System Data	AEKEY	This is a record identifier that is created by the HES system. The digits store a decimal number. This is commonly eight or nine digits but can be up to 14.
HES APC	Hospital Episode Statistics Admitted Patient Care	System Data	CDSEXTDATE	This is a system field that represents the date of the update event that resulted in the need to exchange the data with the Secondary Uses Service (SUS) database. This field is present on records submitted via the Bulk Update Protocol method only, and is used to apply records correctly to SUS.
HES APC	Hospital Episode Statistics Admitted Patient Care	System Data	EPIKEY	This field is present on A&E and APC linked records only. Linkage between these two datasets enables patient pathways to be followed and provides additional information beyond what is available from the standalone datasets.<br><br>EPIKEY represents the record identifier of the APC episode that the A&E attendance has been linked to.<br><br>Full details of the methodology used to link A&E and APC records can be found on the following page of our website: http://content.digital.nhs.uk/article/1824/How-do-we-collect-and-process-HES-data
HES APC	Hospital Episode Statistics Admitted Patient Care	System Data	SUBDATE	Date on which the data used to generate the HES record was received by the Secondary Uses Service.
HES APC	Hospital Episode Statistics Admitted Patient Care	System Data	SUSLDDATE	The date that the record was loaded into the SUS staging system.
HES APC	Hospital Episode Statistics Admitted Patient Care	System Data	SUSRECID	SUS (Secondary Uses Service)-generated record identifier.
HES APC	Hospital Episode Statistics Admitted Patient Care	All fields	PERSON_ID_DEID	The linkable patient identifier, made by NHS Digital linking the HESID with MPS.
HES APC	Hospital Episode Statistics Admitted Patient Care	All fields	GPPRAC	This field contains a code which defines the practice that the patient is registered with.

HES APC	Hospital Episode Statistics Admitted Patient Care	All fields	SITEDIST	Provides the distance in kilometers between the LSOA centroid and the site of treatment. Site of treatment is defined as first five characters of SITETRET or first 3 characters of SITETRET (if SITETRET is not populated with 5 characters)
HES APC	Hospital Episode Statistics Admitted Patient Care	All fields	SITEDIST_FLAG	Flag to indicate if the SITETRET field had 3 or 5 characters to use in deriviation of SITEDIST
HES APC	Hospital Episode Statistics Admitted Patient Care	All fields	PROVDIST	Provides the distance in kilometers between the LSOA centroid and the site of treatment. Site of treatment is defined as PROCODE5 (if populated) or PROCODE3 (if PROCODE5 is not populated or the last two characters of PROCODE5 are 00).
HES APC	Hospital Episode Statistics Admitted Patient Care	All fields	PROVDIST_FLAG	Flag to indicate if the PROVDIST field was calculated using PROCODE5 or PROCODE3 (if PROCODE5 was not populated or last two characters were 00).
HES APC	Hospital Episode Statistics Admitted Patient Care	All fields	NER_GP_PRACTICE	This derived field contains the code for the NHS England region where the patient's GP practice code is registered. It is derived from GPPRAC.
HES APC	Hospital Episode Statistics Admitted Patient Care	All fields	NER_RESIDENCE	This derived field contains the code for the NHS England region where the patient lives.
HES APC	Hospital Episode Statistics Admitted Patient Care	All fields	NER_TREATMENT	This derived field contains the code for the NHS England region where the patient was treated. It is derived from the CCG of treatment.
HES APC	Hospital Episode Statistics Admitted Patient Care	All fields	RANK_ORDER	This field is present on A&E and APC linked records only. Linkage between these two datasets enables patient pathways to be followed and provides additional information beyond what is available from the standalone datasets.<br><br>A score of one to four is applied to each linked record, with a score of one meaning a very good link and a score of four meaning a poor link. Records that score poor in quality, rating either three or four, are excluded from the final output of linked data available to users.<br><br>Full details of the methodology used to link A&E and APC records, and the filters used to assess linkage quality, can be found on the following page of our website: http://content.digital.nhs.uk/article/1824/How-do-we-collect-and-process-HES-data
HES APC	Hospital Episode Statistics Admitted Patient Care	All fields	SPELDUR_CALC	This derived field represents the duration of the spell (SPELDUR), but with day case and regular admission patient classifications excluded. It is derived on finished episodes only.

**HES CC**	**Hospital Episode Statistics Critical Care**	-	-	**Hospital Episodes Statistics Critical Care data. Secondary Care.**
HES CC	Hospital Episode Statistics Critical Care	Critical care adult activity data	aressupdays	Number of days of advanced respiratory support, defined as;<br>• Invasive medical ventilatory support applied via a trans-laryngeal tracheal tube or applied via a tracheostomy.<br>• Bi-level positive airway pressure applied via a trans-laryngeal tracheal tube or applied via a tracheostomy.<br>• Continuous positive airway pressure via a trans-laryngeal tracheal tube.<br>• Extracorporeal respiratory support.
HES CC	Hospital Episode Statistics Critical Care	Critical care adult activity data	bressupdays	Number of days of basic respiratory support defined as;<br>• More than 50% oxygen delivered by face mask. (Note: more than 50% has been chosen to identify the more seriously ill patients in a hospital). Short-term increases in the fraction of inspired oxygen (FiO2) to facilitate procedures such as transfers or physiotherapy do not quality.<br>• Close observation due to the potential for acute deterioration to the point of needing advanced respiratory support. (e.g. severely compromised airway or deteriorating respiratory muscle function).<br>• Physiotherapy or suction to clear secretions at least wo hourly, whether via tracheostomy, minitracheostomy or in the absence of an artificial airway.<br>• Patients recently (within 24 hours) extubated after a period (greater than 24 hours) of mechanical ventilation via endotracheal tube.<br>• Mask / hood continuous positive airway pressure (CPAP) or mark / hood Bi-level positive airway pressure ventilation (non-invasive ventilation).<br>• Patients who are intubated to protect the airway but needing no ventilatory support.<br>• Continuous positive airway pressure (CPAP) via a tracheostomy. Note. The presence of a tracheostomy used for long term airway access only does not quality for any respiratory support.
HES CC	Hospital Episode Statistics Critical Care	Critical care adult activity data	acardsupdays	Number of days of advanced cardiovascular support, defined as:<br>• Multiple intravenous vasoactive and / or rhythm controlling drugs when used simultaneously to support or control arterial pressure, cardiac output or organ perfusion (e.g. inotropes, amiodarone, nitrates). To qualify for advanced support status, at least one drug needs to be vasoactive.<br>• Continuous observation or cardiac output and derived indices (e.g. pulmonary artery catheter, lithium dilution, pulse contour analyses, oesophageal doppler).<br>• Intra aortic balloon pumping and other assist devices.<br>• Insertion of a temporary cardiac pacemaker (criteria valid for each day of connection to a functioning external pacemaker unit).
HES CC	Hospital Episode Statistics Critical Care	Critical care adult activity data	bcardsupdays	Number of days of basic cardiovascular support, defined as;<br>• Use of a central venous pressure (CVP) line for monitoring of central venous pressure and / or provision of central venous access to deliver titrated fluids to treat hypovolaemia.<br>• Use of an arterial line for monitoring of arterial pressure and / or sampling of arterial blood.<br>• Single intravenous vasoactive drug used to support or control arterial pressure, cardiac output or organ perfusion.<br>• Single intravenous rhythm controlling drug to support or control cardiac arrhythmias.
HES CC	Hospital Episode Statistics Critical Care	Critical care adult activity data	rensupdays	Number of days of renal support in the context of critical illness, defined as;<br>• Acute renal replacement therapy (e.g. haemodialysis, haemofiltration etc.) or the provision of renal replacement therapy to a chronic renal failure patient who is requiring other acute organ support in a critical care situation.
HES CC	Hospital Episode Statistics Critical Care	Critical care adult activity data	neurosupdays	Number of days of neurological support, defined as;<br>• Central nervous system depression sufficient to prejudice the airway and protective reflexes, excepting that caused by sedation prescribed to facilitate mechanical ventilation or poisoning (e.g. deliberate or accidental overdose, alcohol, drugs etc.).<br>• Invasive neurological monitoring, e.g. intracranial pressure, jugular bulb sampling, external ventricular drain.<br>• Continuous intravenous medication to control seizures and / or continuous cerebral monitoring.<br>• Therapeutic hypothermia using coding protocols or devices.
HES CC	Hospital Episode Statistics Critical Care	Critical care adult activity data	gisupdays	Number of days of gastrointestinal support, defined as;<br>• Feeding with parenteral or enteral nutrition (implies methods of feeding other than normal oral intake).
HES CC	Hospital Episode Statistics Critical Care	Critical care adult activity data	dermsupdays	Number of days of dermatological support, defined as;<br>• Patients with major akin rashes, exfoliation or burns (e.g. greater than 30% body surface area affected).<br>• Use of complex dressings (e.g. large skin area greater than 30% body surface area, open abdomen, vacuum dressings or large trauma such as multiple limb or limb and head dressings).
HES CC	Hospital Episode Statistics Critical Care	Critical care adult activity data	liversupdays	Number of days of liver support where patients falls into one of the following categories;<br>• Acute on chronic Hepatocellular failure requiring management of coagulopathy and / or portal hypertension (including liver purification and detoxification techniques).<br>• Primary Acute Hepatocellular failure patients who are being considered for transplantation and require management of coagulopathy and / or portal hypertension (including liver purification and detoxification techniques)
HES CC	Hospital Episode Statistics Critical Care	Critical care adult activity data	orgsupmax	Maximum number of organ systems supported at any one time, at any point in the critical care period (note both basic and advanced categories cannot be counted at the same time). This may not be the same as the total number of organs supported throughout the critical care admission. The minimum for this variable is 00 and the maximum is 07 for the full data set.
HES CC	Hospital Episode Statistics Critical Care	Critical care adult activity data	cclev2days	Total calendar days during which level 2 care alone was provided during the period.
HES CC	Hospital Episode Statistics Critical Care	Critical care adult activity data	cclev3days	Description<br>Total calendar days during which level 3 care alone was provided during the period.
HES CC	Hospital Episode Statistics Critical Care	Critical care adult admission and discharge data	unitbedconfig	The composition of bed types for your unit based on maximum funded and intended use, e.g. some units plan to use staff and beds flexibly, others are organised to take a full complement of level three patients or only 'HDU' patients.

HES CC	Hospital Episode Statistics Critical Care	Critical care adult admission and discharge data	ccadmitype	An indication of whether a critical are period was initiated as a result of a non-emergency treatment plan, for example, for none elective surgery. This relates only to the period of critical care and not to the nature of the hospital admission. For example, a planned hospital admission may unexpectedly require an emergency intensive care unit admission, in which case the classification will be '01'.
HES CC	Hospital Episode Statistics Critical Care	Critical care adult admission and discharge data	ccadmisorc	Information on the source of the patient is of use in analysing unit workload and outcomes. Exactly the same classification is used as developed in collaboration with ICNARC (the Intensive Care National Audit and Research Centre). Admission sequences are captured in two stages, i.e. there are two variables collected before unit admission, the critical care admission source and the location associated with the source (CCSORLOC).
HES CC	Hospital Episode Statistics Critical Care	Critical care adult admission and discharge data	ccsorcloc	The type of location the patient was in prior to the start of the critical care period.
HES CC	Hospital Episode Statistics Critical Care	Critical care adult admission and discharge data	ccdisstat	Status at time of discharge.
HES CC	Hospital Episode Statistics Critical Care	Critical care adult admission and discharge data	ccdisdest	Destination of critical care discharge.
HES CC	Hospital Episode Statistics Critical Care	Critical care adult admission and discharge data	ccdisloc	The principal location that the patient is being discharge to for further care at the end of the critical care period.
HES CC	Hospital Episode Statistics Critical Care	Critical care adult admission and discharge data	ccdisrdydate	The date on which the patient has been declared clinically ready for discharge or transfer from the critical care period and a formal request has been made to the Hospital Bed management system (or appropriate staff with authority to admit at the intended destination) and the date and time of this status is recorded as such in the clinical record.
HES CC	Hospital Episode Statistics Critical Care	Critical care period	ccstartdate	provider spell for a health care provider.
HES CC	Hospital Episode Statistics Critical Care	Critical care period	ccstarttime	Time at which the critical care period started.
HES CC	Hospital Episode Statistics Critical Care	Critical care adult admission and discharge data	ccdisrdytime	The time on which the patient has been declared clinically ready for discharge or transfer from the clinical car period and a formal request has been made to the Hospital Bed management system (or appropriate staff with authority to admit at the intended destination) and the date and time of this status is recorded as such in the clinical record.
HES CC	Hospital Episode Statistics Critical Care	Critical care period	ccdisdate	Discharge date from unit if alive, date of death or date of declaration of brain stem death.
HES CC	Hospital Episode Statistics Critical Care	Critical care period	ccdistime	Discharge time from unit.
HES CC	Hospital Episode Statistics Critical Care	Critical care period	ccunitfun	The type of area to which the patient was admitted during a critical care period. This is the principal clinical service provided within the ward.
HES CC	Hospital Episode Statistics Critical Care	Critical care period	bestmatch	A flag stating whether the row represents the best match between the critical care and episode start and end dates for this critical care period. This flag is used to limit the data in instances where there is more than one row per critical care period. See Appendix C in the Critical Care 2008-09 publication for further details.
HES CC	Hospital Episode Statistics Critical Care	Critical care period	epiend	This field contains the date on which a patient left the care of a particular consultant, for one of the following reasons: Patient discharged from hospital (includes transfers) or moved to the care of another consultant. A null entry either indicates that the episode was unfinished at the end of the data year, or the date was unknown.
HES CC	Hospital Episode Statistics Critical Care	Critical care period	epistart	This field contains the date on which a patient was under the care of a particular consultant. If a patient has more than one episode in a spell, for each new episode there is a new value of epistart. However, the admission date which is copied to each new episode in a spell will remain unchanged and will be equal to the episode start date of the first episode in hospital.
HES CC	Hospital Episode Statistics Critical Care	Critical care period	epistat	This field tells you whether the episode had finished before the end of the HES data-year (ie whether the episode was still 'live' at midnight on 31 March). For example, if a patient was admitted on 25 March 2005 and was not discharged (or transferred to the care of another consultant) until 4 April 2005, there will be a record describing the unfinished episode (episode status = 1) in the 2004-05 data, and a separate record describing the finished episode (episode status = 3) in the 2005-06 data. Because hospital providers are advised not to include clinical data (diagnosis and operation codes) in unfinished records, these are normally excluded from analyses. Also, if unfinished episodes are included in time series analyses - where data for more than one year is involved - there is a danger of counting the same episode twice.
HES CC	Hospital Episode Statistics Critical Care	Critical care period	epitype	This field contains a code that defines the type of episode, so that groups of similar episodes can be formed.
HES CC	Hospital Episode Statistics Critical Care	Critical care period	admidate	This field contains the date the patient was admitted to hospital at the start of a hospital spell. Admidate is recorded on all episodes within a spell.
HES CC	Hospital Episode Statistics Critical Care	Critical care period	disdate	This field contains the date on which the patient was discharged from hospital. ; It is only present in the record for the last episode of a spell.
HES CC	Hospital Episode Statistics Critical Care	Critical care period	ccapcrel	A comparison of the dates of the critical care period and those of the associated admitted patient care episode. This is used in the derivation of the best match flag by producing a hierarchy of relationships.
HES CC	Hospital Episode Statistics Critical Care	Patient	Spellno	Hospital provider spell number. A number to provide a unique identifier for each hospital provider spell for a health care provider.
HES CC	Hospital Episode Statistics Critical Care	All fields	CCUNITFUN	The type of area to which the patient was admitted during a critical care period. This is the principal clinical service provided within the ward.
HES CC	Hospital Episode Statistics Critical Care	All fields	CDSVERSION	The version of the commissioning data set (CDS) being used.
HES CC	Hospital Episode Statistics Critical Care	All fields	DERMSUPDAYS	Number of days of dermatological support, defined as;<br>• Patients with major akin rashes, exfoliation or burns (e.g. greater than 30% body surface area affected).<br>• Use of complex dressings (e.g. large skin area greater than 30% body surface area, open abdomen, vacuum dressings or large trauma such as multiple limb or limb and head dressings).
HES CC	Hospital Episode Statistics Critical Care	All fields	CCACTIVDATE	The date the patient receives care which is critical care activity. This field is present on neonatal and paediatric daily critical care records
HES CC	Hospital Episode Statistics Critical Care	All fields	CCACTSEQ	A sequence number for the critical care activity within a critical care period, in order of activity date followed by the order that the activity is presented on the record, starting at 1.
HES CC	Hospital Episode Statistics Critical Care	All fields	CCADMISORC	Information on the source of the patient is of use in analysing unit workload and outcomes. Exactly the same classification is used as developed in collaboration with ICNARC (the Intensive Care National Audit and Research Centre). Admission sequences are captured in two stages, i.e. there are two variables collected before unit admission, the critical care admission source and the location associated with the source (CCSORLOC).
HES CC	Hospital Episode Statistics Critical Care	All fields	CCADMITYPE	An indication of whether a critical are period was initiated as a result of a non-emergency treatment plan, for example, for none elective surgery. This relates only to the period of critical care and not to the nature of the hospital admission. For example, a planned hospital admission may unexpectedly require an emergency intensive care unit admission, in which case the classification will be '01'.
HES CC	Hospital Episode Statistics Critical Care	All fields	CCAPCREL	A comparison of the dates of the critical care period and those of the associated admitted patient care episode. This is used in the derivation of the best match flag by producing a hierarchy of relationships.
HES CC	Hospital Episode Statistics Critical Care	All fields	CCDISDATE	Discharge date from unit if alive, date of death or date of declaration of brain stem death.
HES CC	Hospital Episode Statistics Critical Care	All fields	CCEPIREL	The relationship of the critical care activity to the episode carrying it (0 = within the episode, 1 = before the episode, 2 = ends after the episode, 3 = unfinished, 9 = error)
HES CC	Hospital Episode Statistics Critical Care	All fields	CCPEREPREL	The relations ship of the critical care period to the episode carrying it (0 = within the episode, 1 = starts before episode but ends within episode, 2 = starts within episode but ends after episode, 3 = unfinished, 9 = error)
HES CC	Hospital Episode Statistics Critical Care	All fields	CCPERLASTFLG	Indicates whether the record is the last in a critical care period (0 = not the last record in a critical care period, 1 = the last record in a critical care period). This field is derived on neonatal and paediatric critical care records only.
HES CC	Hospital Episode Statistics Critical Care	All fields	CCPERNUM	A sequence number for the critical care period within the episode in ascending order of critical care start date and critical care start time, starting at 1.
HES CC	Hospital Episode Statistics Critical Care	All fields	CCPERTYPE	Field to indicate whether the critical care period completed within the financial year, or is still open at the end of the financial year.
HES CC	Hospital Episode Statistics Critical Care	Patient	procode3	The organisation code of the organisation acting as the health care provider. This field contains the first 3-characters of the provider code (PROCODE) field, which can be used to identify an individual provider (e.g. NHS Trust or PCT* (*before April 2013)).
HES CC	Hospital Episode Statistics Critical Care	Patient	pseudo_hesid	This field contains a unique identifier for each individual patient. This allows an individual's care to be tracked across years and continuous periods to be identified. This is a pseudonymised version of the HES ID field based on an updated matching algorithm, which supersedes and is compatible with the original HES ID, which is no longer available.

HES CC	Hospital Episode Statistics Critical Care	All fields	GESTDEL	The number of completed weeks gestation at delivery, between 10 and 49. This field is only populated on neonatal critical care records.
HES CC	Hospital Episode Statistics Critical Care	All fields	HCDCODE1 to 20	High Cost Drug code 1 to 20
HES CC	Hospital Episode Statistics Critical Care	All fields	NCCACTS	The total number of critical care activities associated with the critical care period. The maximum value is 999. 9999 is used to indicate 'not applicable'.
HES CC	Hospital Episode Statistics Critical Care	All fields	ORGSUPMAX	Maximum number of organ systems supported at any one time, at any point in the critical care period (note both basic and advanced categories cannot be counted at the same time). This may not be the same as the total number of organs supported throughout the critical care admission. The minimum for this variable is 00 and the maximum is 07 for the full data set.
HES CC	Hospital Episode Statistics Critical Care	All fields	PERIOD	The period, or HES extract number (e.g. 1 = month 1, 2 = month 2 etc.)
HES CC	Hospital Episode Statistics Critical Care	All fields	PROCSCHM	The procedure scheme used to record high cost drugs on paediatric and neonatal critical care records (NB: this field is for future use - currently set to constant value of 02).
HES CC	Hospital Episode Statistics Critical Care	All fields	PROVSPNOPS	Pseudonymised hospital provider spell number. A number to provide a unique identifier for each Hospital Provider Spell for a Health Care Provider.
HES CC	Hospital Episode Statistics Critical Care	All fields	PSEUDSTAT	The pseudonymisation status (NB: this field is for future use - currently set to 0).
HES CC	Hospital Episode Statistics Critical Care	All fields	RECTYPE	Indicates the type of APC record that the critical care record is associated with (C1 = unfinished general, C2 = derived unfinished general, C3 = finished general, C4 = unfinished delivery, C5 = derived unfinished delivery, C6 = finished delivery, C7 = unfinished birth, C8 = derived unfinished birth, C9 = finished birth)
HES CC	Hospital Episode Statistics Critical Care	All fields	WEIGHTTYPE	The patient's weight in kilograms (kg). This field is present on neonatal critical care records only, and represents the last recorded weight on a particular activity date.
HES CC	Hospital Episode Statistics Critical Care	System Data	susrecid	SUS (Secondary Uses Service)-generated record identifier.
HES CC	Hospital Episode Statistics Critical Care	All fields	CCACTCODE1 to 20	Critical Care Activity Code (https://datadictionary.nhs.uk/attributes/critical_care_activity_code.html)
HES CC	Hospital Episode Statistics Critical Care	All fields	CDSEXTDATE	This is a system field that represents the date of the update event that resulted in the need to exchange the data with the Secondary Uses Service (SUS) database. This field is present on records submitted via the Bulk Update Protocol method only, and is used to apply records correctly to SUS.
HES CC	Hospital Episode Statistics Critical Care	All fields	DISDATE	This field contains the date on which the patient was discharged from hospital. ; It is only present in the record for the last episode of a spell.
HES CC	Hospital Episode Statistics Critical Care	All fields	EPIEND	This field contains the date on which a patient left the care of a particular consultant, for one of the following reasons: Patient discharged from hospital (includes transfers) or moved to the care of another consultant. A null entry either indicates that the episode was unfinished at the end of the data year, or the date was unknown.
HES CC	Hospital Episode Statistics Critical Care	All fields	FYEAR	Financial year of the HES data i.e. 2021 - 2020 - 2021
HES CC	Hospital Episode Statistics Critical Care	All fields	PARTYEAR	This field represents the year and month of the HES dataset in the format yyyymm. For example, 201601 represents the month 1 2016-17 HES dataset; 201602 represents the month 2 2016-17 HES dataset etc.
HES CC	Hospital Episode Statistics Critical Care	All fields	PROCODE	The organisation code of the organisation acting as the health care provider.<br><br>NHS organisations are allocated a main 3-character code, however this field may contain the 3-character organisation code or a full 5-character code (e.g. a site code, a HQ code ending 01 etc.). Independent providers will always be represented in this field by a 5-character code. Users wishing to group data at 3-character level (which will identify an individual organisation - e.g. NHS Trust) may wish to use the field PROCODE3.<br><br>Note that details of the site code can be found in the field 'site code of treatment' (SITETRET).
HES CC	Hospital Episode Statistics Critical Care	All fields	PROCODE3	The organisation code of the organisation acting as the health care provider. This field contains the first 3-characters of the provider code (PROCODE) field, which can be used to identify an individual provider (e.g. NHS Trust or PCT* (*before April 2013)).
HES CC	Hospital Episode Statistics Critical Care	All fields	PROCODE5	The organisation code of the organisation acting as the health care provider. This field contains the provider code (PROCODE) to 5-characters. Where the field PROCODE contains a 3-character code, it will be padded to the right with additional zeros in this field.<br><br>Note that details of the site code can be found in the field 'site code of treatment' (SITETRET).
HES CC	Hospital Episode Statistics Critical Care	All fields	SUSLDDATE	The date that the record was loaded into the SUS staging system.
**HES OP**	**Hospital Episode Statistics Outpatients**	-	-	**Hospital Episodes Statistics Outpatient data. Secondary Care. Derived from SUS and made person linkable on pseudonomised NHS number. Updated once a month.**
HES OP	Hospital Episode Statistics Outpatients	Appointments	APPTDATE	The date when an appointment was scheduled.
HES OP	Hospital Episode Statistics Outpatients	Appointments	ATENTYPE	A field derived from First Attendance (firstatt) and Attended or Did Not Attend (attended), used to identify if the attendance occurred and whether it was the first or subsequent.
HES OP	Hospital Episode Statistics Outpatients	Appointments	ATTENDED	This indicates whether or not a patient attended for an appointment. If the patient did not attend it also indicates whether or not advanced warning was given.
HES OP	Hospital Episode Statistics Outpatients	Appointments	ATTENDID	A sequential number or time of day, assigned locally, that is unique to only one activity for a patient within an organisation. As this field is often locally generated, the data in this field are not currently unique within a dataset. However, as the NHS moves towards central systems this should change.
HES OP	Hospital Episode Statistics Outpatients	Appointments	DNADATE	This is recorded when patients who have been offered an appointment date have missed this date with or without advance notice.
HES OP	Hospital Episode Statistics Outpatients	Appointments	FIRSTATT	Gives an indication of whether a patient is making a first attendance or follow-up attendance, and whether the consultation was face-to-face or via telephone/telemedicine consultation.
HES OP	Hospital Episode Statistics Outpatients	Appointments	LOCCLASS	A classification for use within CDS messages of the physical location within which the recorded patient event occurs.
HES OP	Hospital Episode Statistics Outpatients	Appointments	LOCTYPE	This records the type of location. The definition of this field is currently under review.
HES OP	Hospital Episode Statistics Outpatients	Appointments	OUTCOME	This records the outcome of an outpatient attendance.
HES OP	Hospital Episode Statistics Outpatients	Appointments	PARTYEAR	This field represents the year and month of the HES dataset in the format yyyymm. For example, 201601 represents the month 1 2016-17 HES dataset; 201602 represents the month 2 2016-17 HES dataset etc.
HES OP	Hospital Episode Statistics Outpatients	Appointments	PRIORITY	This is the priority of a request for services; in the case of services to be provided by a consultant, it is as assessed by or on behalf of the consultant.
HES OP	Hospital Episode Statistics Outpatients	Appointments	REFSOURC	A classification which is used to identify the source of referral of each consultant outpatient episode.
HES OP	Hospital Episode Statistics Outpatients	Appointments	REQDATE	The date the referral request was received by the healthcare provider.
HES OP	Hospital Episode Statistics Outpatients	Appointments	SERVTYPE	Describes the terms of reference for the referral request.
HES OP	Hospital Episode Statistics Outpatients	Appointments	STAFFTYP	Gives information about the type of care professional staff dealing with the patient during a consultant outpatient attendance, or nurse or midwife contact.
HES OP	Hospital Episode Statistics Outpatients	Appointments	WAIT_IND	WAIT_IND indicates how and whether the field 'Days Waiting' (WAITING) has been calculated.
HES OP	Hospital Episode Statistics Outpatients	Appointments	WAITING	'Waiting' gives the period in days between the date of the appointment date and either the referral request received date (reqdate) or the DNA (did not attend) date, if given. If the calculation returns a negative the waiting time is set as null. Waiting time is not calculated if reqdate is missing or invalid. (Waitind (see below) indicates how and whether waiting time has been calculated.) Note: As records can relate to DNAs, it is possible to have a waiting time for the record even where the patient didn't attend.
HES OP	Hospital Episode Statistics Outpatients	Clinical	DIAG_3_CONCAT	Provides a concatenated string of all diagnoses from the record at a 3 character level, separated by a comma with no spaces. This enables the user to search across the full list of diagnoses to look at mentions and pairs of diagnosis codes.
HES OP	Hospital Episode Statistics Outpatients	Clinical	DIAG_4_CONCAT	Provides a concatenated string of all diagnoses from the record at a 4 character level, separated by a comma with no spaces. This enables the user to search across the full list of diagnoses to look at mentions and pairs of diagnosis codes.
HES OP	Hospital Episode Statistics Outpatients	Clinical	MAINSPEF	This field contains a code that defines the specialty under which the consultant is contracted. It can be compared with tretspef, the specialty under which the consultant worked.
HES OP	Hospital Episode Statistics Outpatients	Clinical	OPERTN_4_CONCAT	Provides a concatenated string of all procedures from the record at a 4 character level, separated by a comma with no spaces. This enables the user to search across the full list of procedures to look at mentions and pairs of procedure codes.

HES OP	Hospital Episode Statistics Outpatients	Clinical	TRETSPEF	This field contains a code that defines the specialty in which the consultant was working during the period of care. It can be compared with mainspef, the specialty under which the consultant is contracted. Prior to 1 April 1996, this data item contained the code for the sub-specialty (subspef). From April 2004 a new list of treatment specialities was introduced (see below). The new list describes the specialised service within which the patient was treated.
HES OP	Hospital Episode Statistics Outpatients	Diagnosis	DIAG_COUNT	The total number of diagnosis codes present on the record (maximum of 20)
HES OP	Hospital Episode Statistics Outpatients	Geographical	AT_GP_PRACTICE	This derived field contains the code for the Area Team (AT) where the patient's GP practice is registered. It is derived from Code of GP practice (GPPRAC).<br><br>In 2015 following an NHS reorganisation, Area Teams were replaced by NHS England regions. This field has continued to be derived after this date as a 'frozen' geography. The NHS England region of the GP practice can be found in the field NER_GP_PRACTICE.<br>This field will no longer be used from April 2021 onwards
HES OP	Hospital Episode Statistics Outpatients	Geographical	AT_RESIDENCE	This derived field contains the code for the Area Team (AT) where the patient lives. It is derived from the CCG of residence.<br><br>In 2015 following an NHS reorganisation, Area Teams were replaced by NHS England regions. This field has continued to be derived after this date as a 'frozen' geography. The NHS Region of residence can be found in the field NER_RESIDENCE.<br>This field will no longer be used from April 2021 onwards
HES OP	Hospital Episode Statistics Outpatients	Geographical	AT_TREATMENT	This derived field contains the code for the Area Team (AT) where the patient was treated. It is derived from the CCG of Treatment.<br><br>In 2015 following an NHS reorganisation, Area Teams were replaced by NHS England regions. This field has continued to be derived after this date as a 'frozen' geography. The NHS England region of treatment can be found in the field NER_TREATMENT.<br>This field will no longer be used from April 2021 onwards
HES OP	Hospital Episode Statistics Outpatients	Geographical	CANNET	This field, which is derived from the patient's postcode in the field HOMEADD, contains a code which defines the former cancer network area that the postcode falls within.<br><br>Cancer networks were abolished in 2013. This field has continued to be derived after this date as a 'frozen' geography.<br>This field will no longer be used from April 2021 onwards
HES OP	Hospital Episode Statistics Outpatients	Geographical	CANREG	This field, which is derived from the patient's postcode in the field HOMEADD, contains a code which defines the cancer registry area that the postcode falls within.
HES OP	Hospital Episode Statistics Outpatients	Geographical	CCG_GP_PRACTICE	This derived field contains the code for the Clinical Commissioning Group (CCG) where the patient's GP practice is registered. It is derived from GP Pracrice (GGPRAC).
HES OP	Hospital Episode Statistics Outpatients	Geographical	CCG_RESIDENCE	This derived field contains the code for the Clinical Commissioning Group (CCG) where the patient lived immediately before admission. It is derived from post code (HOMEADD).
HES OP	Hospital Episode Statistics Outpatients	Geographical	CCG_RESPONSIBILITY	This derived field contains the code for the most suitable Clinical Commissioning Group (CCG) of responsibility. It is derived firstly from the patient's GP practice but if not available the patient's CCG of residence then the CCG of treatment is used.
HES OP	Hospital Episode Statistics Outpatients	Geographical	CCG_RESPONSIBILITY_ORIGIN	This derived field indicates the basis on which the CCG of Responsibility was assigned.
HES OP	Hospital Episode Statistics Outpatients	Geographical	CCG_TREATMENT	This derived field contains the code for the Clinical Commissioning Group (CCG) where the patient was treated. It is derived from the postcode of the Site of Treatment firstly, but where not available the postcode of the Provider is used.
HES OP	Hospital Episode Statistics Outpatients	Geographical	CCG_TREATMENT_ORIGIN	This derived field indicates the basis on which the CCG of Treatment was assigned.
HES OP	Hospital Episode Statistics Outpatients	Geographical	CR_GP_PRACTICE	This derived field contains the code for the Commissioning Region (CR) where the patient's GP practice is registered. Where not available, the code for the Area Team is used. It is derived from gpprac.
HES OP	Hospital Episode Statistics Outpatients	Geographical	CR_RESIDENCE	This derived field contains the code for the Commissioning Region (CR) where the patient lived immediately before admission. Where not available, the code for the Area Team is used. It is derived from the CCG of Residence.
HES OP	Hospital Episode Statistics Outpatients	Geographical	CR_TREATMENT	This derived field contains the code for the Commissioning Region (CR) where the patient was treated. Where not available, the code for the Area Team is used. It is derived from the CCG of Treatment.
HES OP	Hospital Episode Statistics Outpatients	Geographical	GORTREAT	Government Office Region (GOR) of treatment. This field is derived from the hospital provider code (procode). It indicates the GOR area within which the treatment took place.
HES OP	Hospital Episode Statistics Outpatients	Geographical	LSOA01	This field, which is derived from the patient's postcode in the field HOMEADD, represents the 2001 Census Lower Layer SOA code for England and Wales, SOA code for Northern Ireland and data zone code for Scotland. Pseudo codes are included for Channel Islands and Isle of Man. The field will otherwise be blank for postcodes with no grid reference. The first character is either E for England or W for Wales The next two characters are 01 for Lower Super Output Area and the remaining six characters make up the unique 6-digit tag for each zone.
HES OP	Hospital Episode Statistics Outpatients	Geographical	LSOA11	This field, which is derived from the patient's postcode in the field HOMEADD, represents the 2011 Census lower layer SOA code for England and Wales, SOA code for Northern Ireland and data zone code for Scotland. Pseudo codes are included for Channel Islands and Isle of Man. The field will otherwise be blank for postcodes with no grid reference. N.B. this field remains blank for Scottish postcodes until this information is released. The first character is either E for England or W for Wales The next two characters are 01 for Lower Super Output Area and the remaining six characters make up the unique 6-digit tag for each zone.
HES OP	Hospital Episode Statistics Outpatients	Geographical	MSOA01	This field, which is derived from the patient's postcode in the field HOMEADD, represents the 2001 Census Middle Layer SOA (MSOA) code for England and Wales and intermediate zone for Scotland. Pseudo codes are included for Northern Ireland, Channel Islands and Isle of Man. The field will otherwise be blank for postcodes with no grid reference. The first character is either E for England or W for Wales. The next two characters are 02 for Middle Layer and the remaining six characters make up the unique 6-digit tag for each zone.
HES OP	Hospital Episode Statistics Outpatients	Geographical	MSOA11	This field, which is derived from the patient's postcode in the field HOMEADD, represents the 2011 Census middle layer SOA (MSOA) code for England and Wales and intermediate zone for Scotland. Pseudo codes are included for Northern Ireland, Channel Islands and Isle of Man. The field will otherwise be blank for postcodes with no grid reference. N.B. this field remains blank for Scottish postcodes until this information is released. The first character is either E for England or W for Wales. The next two characters are 02 for Middle Layer and the remaining six characters make up the unique 6-digit tag for each zone.
HES OP	Hospital Episode Statistics Outpatients	Geographical	PCON	For activity from 2011-12 onwards, HES users are advised to use the field PCON_ONS instead of PCON.<br><br>This field, which is derived from the patient's postcode in the field HOMEADD, defines the code for the English Westminster Parliamentary Constituency where the patient resides. Note that this field uses old-style geographical codes that were superseded in 2011 and are now no longer supported by ONS - the new-style code can be found in the field PCON_ONS.<br>This field will no longer be used from April 2021 onwards
HES OP	Hospital Episode Statistics Outpatients	Geographical	PCON_ONS	This field, which is derived from the patient's postcode in the field HOMEADD, defines the code for the English Westminster Parliamentary Constituency where the patient resides.
HES OP	Hospital Episode Statistics Outpatients	Geographical	PCTTREAT	This field is derived from the hospital provider code (procode). It indicates the PCT area of the main provider of treatment. Note that the PCT itself may be the provider of treatment.<br>Note: This field was formerly known as "Primary care trust area of treatment".<br><br>In 2013 following a reorganisation of health areas, Primary Care Trusts were abolished. This field has continued to be derived after this date as a 'frozen' geography.<br>This field will no longer be used from April 2021 onwards

HES OP	Hospital Episode Statistics Outpatients	Geographical	RESCTY	For activity from 2011-12 onwards, HES users are advised to use the field RESCTY_ONS instead of RESCTY.<br><br>This field contains a code that defines the county of residence of the patient. It is derived from the patient's postcode in the field homeadd (postcode of patient).<br><br>Note that this field uses old-style geographical codes that were superseded in 2011 and are now no longer supported by ONS. The field RESCTY_ONS represents the county of residence using codes from the current ONS geographical coding system.<br><br>This field will no longer be used from April 2021 onwards
HES OP	Hospital Episode Statistics Outpatients	Geographical	RESCTY_ONS	This field contains a code that defines the county of residence of the patient. It is derived from the patient's postcode in the field homeadd.
HES OP	Hospital Episode Statistics Outpatients	Geographical	RESGOR	For activity from 2011-12 onwards, HES users are advised to use the field RESGOR_ONS instead of RESGOR<br><br>The Government Office Region of residence field contains a code that defines the Government Office Region of residence of the patient. It is derived from the patient's postcode in the field homeadd. The Government Office Regions closed on 31 March 2011, but this regional geography is maintained for statistical purposes and after this date referred to simply as 'regions'.<br><br>Note that this field uses old-style geographical codes that were superseded in 2011 and are now no longer supported by ONS. The field RESGOR_ONS represents the Government Office Region of residence using codes from the current ONS geographical coding system.<br>This field will no longer be used from April 2021 onwards
HES OP	Hospital Episode Statistics Outpatients	Geographical	RESGOR_ONS	The Government Office Region of residence field contains a code that defines the Government Office Region of residence of the patient. It is derived from the patient's postcode in the field homeadd. The Government Office Regions closed on 31 March 2011, but this regional geography is maintained for statistical purposes and after this date referred to simply as 'regions'.
HES OP	Hospital Episode Statistics Outpatients	Geographical	RESLADST	For activity from 2011-12 onwards, HES users are advised to use the field RESLADST_ONS instead of RESLADST.<br><br>This field contains a code that defines the county (first two characters) and local authority district (last two characters) of residence of the patient. It is derived from the patient's postcode in the field homeadd. Note that this field uses old-style geographical codes that were superseded in 2011 and are now no longer supported by ONS. The field RESLADST_ONS represents the local authority district of residence using codes from the current ONS geographical coding system.<br><br>RESLADST is used in conjunction with CURRWARD (current electoral ward) to produce a unique value indicating the ward within a given district where the patient resided (ie because identical CURRWARD codes are allocated to many local authority districts, CURRWARD is meaningless in isolation). If the patient is resident within a Unitary Authority, the first two characters will be 00 (zero, zero) and the local authority component may not be useable.<br>This field will no longer be used from April 2021 onwards
HES OP	Hospital Episode Statistics Outpatients	Geographical	RESLADST_ONS	This derived field contains a code which defines the current local authority district of residence of the patient. It is derived from the patient's postcode in the field homeadd.
HES OP	Hospital Episode Statistics Outpatients	Geographical	RESSTHA	This derived field contains the code for the strategic health authority (SHA) in which the patient lived immediately before admission. It is derived from the patient's postcode in the field homeadd.<br><br>In 2013 following a reorganisation of health areas, Strategic Health Authorities were abolished. This field has continued to be derived after this date as a 'frozen' geography.
HES OP	Hospital Episode Statistics Outpatients	Geographical	SITETRET	This field contains a code that defines the site on which the patient was treated within an organisation. The code recorded should be the one of the Health Care Provider actually carrying out the work.<br>It contains the first 3 digit of the Provider Code with the last two digits being the site identifier.
HES OP	Hospital Episode Statistics Outpatients	Geographical	STHATRET	This field contains a code that defines the Strategic Health Authority (SHA) area where the treatment took place. It is derived from the hospital provider code in the field procode. Note: (1) for NHS trusts, care provided at subsidiary sites will be attributed to the main trust location.<br><br>SHAs were abolished in 2013. This field has continued to be derived after this date as a 'frozen' geography.<br><br>This field will no longer be used from April 2021 onwards
HES OP	Hospital Episode Statistics Outpatients	Healthcare resource groups (HRG) data	SUSHRG	The SUS PbR derived healthcare resource group (HRG) code.
HES OP	Hospital Episode Statistics Outpatients	Organisation	GPPRAC	This field contains a code which defines the practice that the patient is registered with.
HES OP	Hospital Episode Statistics Outpatients	Organisation	PROCODE3	The organisation code of the organisation acting as the health care provider. This field contains the first 3-characters of the provider code (PROCODE) field, which can be used to identify an individual provider (e.g. NHS Trust or PCT* (*before April 2013)).
HES OP	Hospital Episode Statistics Outpatients	Organisation	PROCODE5	The organisation code of the organisation acting as the health care provider. This field contains the provider code (PROCODE) to 5-characters. Where the field PROCODE contains a 3-character code, it will be padded to the right with additional zeros in this field.<br><br>Note that details of the site code can be found in the field 'site code of treatment' (SITETRET).
HES OP	Hospital Episode Statistics Outpatients	Organisation	PROCODET	This field gives a combination of 3-character and 5-character provider codes. Procodet enables you to view a combined list of codes, and related data, from:<br>1. Primary care trusts (3 character, beginning with 5)<br>2. NHS trusts (3 character, beginning with R or T. Trusts with associated treatment centres will have an '- X' following their code)<br>3. NHS trust treatment centres (5 character; listed separately to the NHS trusts)<br>4. Independent providers (5 character, beginning with 8)<br>5. Independent sector healthcare providers (5 character, beginning with N or A)<br>For 3-character codes only see procode (provider code (3 character)) and for 5-character codes only see procode5 (provider code (5 character)).
HES OP	Hospital Episode Statistics Outpatients	Organisation	PURCODE	This field contains a code for the organisation commissioning the patient's health care.
HES OP	Hospital Episode Statistics Outpatients	Organisation	PROCODE	The organisation code of the organisation acting as the health care provider.<br><br>NHS organisations are allocated a main 3-character code, however this field may contain the 3-character organisation code or a full 5-character code (e.g. a site code, a HQ code ending 01 etc.). Independent providers will always be represented in this field by a 5-character code. Users wishing to group data at 3-character level (which will identify an individual organisation - e.g. NHS Trust) may wish to use the field PROCODE3.<br><br>Note that details of the site code can be found in the field 'site code of treatment' (SITETRET).
HES OP	Hospital Episode Statistics Outpatients	Organisation	PCTCODE_HIS	The PCTCODE depending on the datayear. Where the data year is equal to or later than 2006, this field contains the code from the field PCTCODE06, otherwise it contains the code from the field PCTCODE02.<br><br>In 2013 following a reorganisation of health areas, Primary Care Trusts were abolished. This field has continued to be derived after this date as a 'frozen' geography.<br>This field will no longer be used from April 2021 onwards

HES OP	Hospital Episode Statistics Outpatients	Organisation	PCTORIG_HIS	The origin of the primary care trust of responsibility dependent on the datayear. Where the data year is equal to or later than 2006, this field contains the code from the field PCTORIG06, otherwise it contains the code from the field PCTORIG02.<br>This field will no longer be used from April 2021 onwards
HES OP	Hospital Episode Statistics Outpatients	Organisation	RESPCT_HIS	The Primary Care Trust (PCT) of residence dependent on the data year.<br><br>If the HES data year is 2006 or later, it will return the value from the field RESPCT06. If the data year is earlier than 2006, it will return the value from the field RESPCT02.<br><br>In 2013 following a reorganisation of health areas, Primary Care Trusts were abolished. This field has continued to be derived after this date as a 'frozen' geography.<br>This field will no longer be used from April 2021 onwards
HES OP	Hospital Episode Statistics Outpatients	Organisation	RESSTHA_HIS	The Strategic Health Authority (SHA) of residence dependent on the data year.<br><br>If the HES data year is 2006 or later, it will return the value from the field RESSTHA06. If the data year is earlier than 2006, it will return the value from the field RESSTHA02.<br><br>In 2013 following a reorganisation of health areas, Strategic Health Authorities were abolished. This field has continued to be derived after this date as a 'frozen' geography.<br><br>This field will no longer be used from April 2021 onwards
HES OP	Hospital Episode Statistics Outpatients	Patient Data	ADMINCAT	Administrative category on admission.
HES OP	Hospital Episode Statistics Outpatients	Patient Data	APPTAGE	This field is derived in HES as the patient's age in whole completed years, calculated from appointment date (apptdate) and date of birth (dob).<br><br>For patients under 1 year old, special codes in the range 7001 to 7007 apply.
HES OP	Hospital Episode Statistics Outpatients	Patient Data	APPTAGE_CALC	Appointment age (APPTAGE), with decimalised values for babies.
HES OP	Hospital Episode Statistics Outpatients	Patient Data	ETHNOS	The ethnicity of the patient, as specified by the patient. This field uses the ethnic data categories as defined in the 2001 census.<br><br>Note that ETHNOS is very poorly populated on activity prior to the 2011-12 data year.
HES OP	Hospital Episode Statistics Outpatients	Patient Data	MYDOB	Month and year of date of birth only. Day is not made available
HES OP	Hospital Episode Statistics Outpatients	Patient Data	SEX	Defines the sex of the patient. The classification is phenotypical rather than genotypical, i.e. it does not provide codes for medical or scientific purposes.<br>Notes:<br>• National Code 'Not Known' means that the sex of a person has not been recorded<br>• National Code 'Not Specified' means indeterminate, i.e. unable to be classified as either male or female.
HES OP	Hospital Episode Statistics Outpatients	Patient Pathway	WAITDAYS	This field is derived in HES to calculate the number of days that a patient waited in a referral to treatment period - this is the difference between the Referral to Treatment Start Date (RTTPERSTART) and Referral to Treatment End Date (RTTPEREND).
HES OP	Hospital Episode Statistics Outpatients	Practitioner	REFERORG	The organisation code of the organisation from which the referral is made, such as GP practice or NHS trust.
HES OP	Hospital Episode Statistics Outpatients	System Data	ATTENDKEY	This is a record identifier that is created by the HES system. The digits store a decimal number. This is commonly eight or nine digits but can be up to 14.
HES OP	Hospital Episode Statistics Outpatients	System Data	ATTENDKEY_FLAG	Flags whether there is an ATTENDKEY present on the record. This can be used to sum the number of outpatient appointments.
HES OP	Hospital Episode Statistics Outpatients	System Data	CDSEXTDATE	This is a system field that represents the date of the update event that resulted in the need to exchange the data with the Secondary Uses Service (SUS) database. This field is present on records submitted via the Bulk Update Protocol method only, and is used to apply records correctly to SUS.
HES OP	Hospital Episode Statistics Outpatients	System Data	SUBDATE	Date on which the data used to generate the HES record was received by the Secondary Uses Service.
HES OP	Hospital Episode Statistics Outpatients	System Data	SUSLDDATE	The date that the record was loaded into the SUS staging system.
HES OP	Hospital Episode Statistics Outpatients	System Data	SUSRECID	SUS (Secondary Uses Service)-generated record identifier.
HES OP	Hospital Episode Statistics Outpatients	Appointments	FYEAR	TBC
HES OP	Hospital Episode Statistics Outpatients	Clinical	OPERTN_3_01	This provides the first three characters of the primary operation codes
HES OP	Hospital Episode Statistics Outpatients	Clinical	OPERTN_4_01	Contains secondary 4 character procedures. ; The codes are defined in the Tabular List of the Classification of Surgical Operations and Procedures. The current version is OPCS4. Procedure codes start with a letter and are followed by two or three digits. The third digit identifies variations on a main procedure code containing two digits. The third digit is preceded by a full stop in OPCS4, but this is not stored in the field. A single operation may contain more than one procedure.
HES OP	Hospital Episode Statistics Outpatients	Diagnosis	DIAG_3_01	This provides the first three characters of diagnosis codes, for the primary diagnosis fields
HES OP	Hospital Episode Statistics Outpatients	Diagnosis	DIAG_4_01	This provides the first four characters of diagnosis codes, for the primary diagnosis fields
HES OP	Hospital Episode Statistics Outpatients	Geographical	RESPCT	This field contains the code for the primary care trust (PCT) in which the patient lived immediately before the last admission before the date of death.
HES OP	Hospital Episode Statistics Outpatients	Organisation	PREFERER	A pseudonymised version of the code of the person referring the patient. The Person referring patient (Referrer) field contains the actual codes. However, it is a sensitive field that requires the approval of the Data Access Advisory Group (DAAG) before you can be given access to it.
HES OP	Hospital Episode Statistics Outpatients	All fields	PERSON_ID_DEID	Unique pseudonymised identifier for "persons" across the datasets. This field can be used as a key to link other datasets at person level
HES OP	Hospital Episode Statistics Outpatients	All fields	ATTENDANCE	This field is derived from the Attended or Did Not Attend (attended) field. It contains a flag that indicates whether the appointment was attended. A value of 1 is set if the attended field contains 5 or 6, otherwise the field is set to 0.
HES OP	Hospital Episode Statistics Outpatients	All fields	AT_GP_PRACTICE	This derived field contains the code for the Area Team (AT) where the patient's GP practice is registered. It is derived from Code of GP practice (GPPRAC).<br><br>In 2015 following an NHS reorganisation, Area Teams were replaced by NHS England regions. This field has continued to be derived after this date as a 'frozen' geography. The NHS England region of the GP practice can be found in the field NER_GP_PRACTICE.<br>This field will no longer be used from April 2021 onwards
HES OP	Hospital Episode Statistics Outpatients	All fields	AT_RESIDENCE	This derived field contains the code for the Area Team (AT) where the patient lives. It is derived from the CCG of residence.<br><br>In 2015 following an NHS reorganisation, Area Teams were replaced by NHS England regions. This field has continued to be derived after this date as a 'frozen' geography. The NHS Region of residence can be found in the field NER_RESIDENCE.<br>This field will no longer be used from April 2021 onwards
HES OP	Hospital Episode Statistics Outpatients	All fields	AT_TREATMENT	This derived field contains the code for the Area Team (AT) where the patient was treated. It is derived from the CCG of Treatment.<br><br>In 2015 following an NHS reorganisation, Area Teams were replaced by NHS England regions. This field has continued to be derived after this date as a 'frozen' geography. The NHS England region of treatment can be found in the field NER_TREATMENT.<br>This field will no longer be used from April 2021 onwards
HES OP	Hospital Episode Statistics Outpatients	All fields	DIAG_3_02 to 12	This provides the first three characters of the diagnosis code.
HES OP	Hospital Episode Statistics Outpatients	All fields	DIAG_4_02 to 12	This provides the first three characters of the diagnosis code.
HES OP	Hospital Episode Statistics Outpatients	All fields	SITEDIST	Provides the distance in kilometers between the LSOA centroid and the site of treatment. Site of treatment is defined as first five characters of SITETRET or first 3 characters of SITETRET (if SITETRET is not populated with 5 characters)
HES OP	Hospital Episode Statistics Outpatients	All fields	SITEDIST_FLAG	Flag to indicate if the SITETRET field had 3 or 5 characters to use in deriviation of SITEDIST
HES OP	Hospital Episode Statistics Outpatients	All fields	PROVDIST	Provides the distance in kilometers between the LSOA centroid and the site of treatment. Site of treatment is defined as PROCODE5 (if populated) or PROCODE3 (if PROCODE5 is not populated or the last two characters of PROCODE5 are 00).
HES OP	Hospital Episode Statistics Outpatients	All fields	PROVDIST_FLAG	Flag to indicate if the PROVDIST field was calculated using PROCODE5 or PROCODE3 (if PROCODE5 was not populated or last two characters were 00).

HES OP	Hospital Episode Statistics Outpatients	All fields	NER_GP_PRACTICE	This derived field contains the code for the NHS England region where the patient's GP practice code is registered. It is derived from GPPRAC.
HES OP	Hospital Episode Statistics Outpatients	All fields	NER_RESIDENCE	This derived field contains the code for the NHS England region where the patient lives.
HES OP	Hospital Episode Statistics Outpatients	All fields	NER_TREATMENT	This derived field contains the code for the NHS England region where the patient was treated. It is derived from the CCG of treatment.
HES OP	Hospital Episode Statistics Outpatients	All fields	OPERTN_3_02 to 24	This provides the first three characters of a secondary operation. There are twenty-four fields (twelve before April 2007), oper_01 to oper_24, which contain information about a patient's operations. The field oper_01 contains the main (ie most resource intensive) procedure. The other fields contain secondary procedures. The codes are defined in the Tabular List of the Classification of Surgical Operations and Procedures. The current version is OPCS4. Procedure codes start with a letter and are followed by two or three digits. The third digit identifies variations on a main procedure code containing two digits. The third digit is preceded by a full stop in OPCS4, but this is not stored in the field. A single operation may contain more than one procedure.
HES OP	Hospital Episode Statistics Outpatients	All fields	OPERTN_3_CONCAT	Provides a concatenated string of all procedures from the record at a 3 character level, separated by a comma with no spaces. This enables the user to search across the full list of procedures to look at mentions and pairs of procedure codes.
HES OP	Hospital Episode Statistics Outpatients	All fields	OPERTN_4_02 to 24	This provides the first four characters of a secondary operation. There are twenty-four fields (twelve before April 2007), oper_01 to oper_24, which contain information about a patient's operations. The field oper_01 contains the main (ie most resource intensive) procedure. The other fields contain secondary procedures. The codes are defined in the Tabular List of the Classification of Surgical Operations and Procedures. The current version is OPCS4. Procedure codes start with a letter and are followed by two or three digits. The third digit identifies variations on a main procedure code containing two digits. The third digit is preceded by a full stop in OPCS4, but this is not stored in the field. A single operation may contain more than one procedure.
HES OP	Hospital Episode Statistics Outpatients	All fields	OPERTN_COUNT	The total number of procedure codes present on the record (maximum of 24)
**primary_care_meds**	**BSA Primary Care Meds (Dispening / Drugs)**	-	-	**NHS Business Services Authority dataset on drug dispensing**
primary_care_meds	BSA Primary Care Meds (Dispening / Drugs)	All fields	BSAPrescriptionID	The identifier of the prescription (unique id along with item id)
primary_care_meds	BSA Primary Care Meds (Dispening / Drugs)	All fields	ChargeStatus	The category for exemption (Charged, Exempt or Old Charge rate).
primary_care_meds	BSA Primary Care Meds (Dispening / Drugs)	All fields	CostCentreLSOA	Prescriber Cost Centre Post Code area
primary_care_meds	BSA Primary Care Meds (Dispening / Drugs)	All fields	CostCentreODSCode	The ODS code for the cost centre that issued the prescription, eg gp practice
primary_care_meds	BSA Primary Care Meds (Dispening / Drugs)	All fields	CostCentreSubType	Sub code which can help identify things such as out of hours, walk in centre, health and justice, public health service, etc
primary_care_meds	BSA Primary Care Meds (Dispening / Drugs)	All fields	CostCentreType	The type of the prescriber eg GP Practice, hospital, etc
primary_care_meds	BSA Primary Care Meds (Dispening / Drugs)	All fields	DispensedCountryCode	Country the prescription was dispensed in
primary_care_meds	BSA Primary Care Meds (Dispening / Drugs)	All fields	DispensedPharmacyLSOA	Dispenser Cost Centre Post Code area
primary_care_meds	BSA Primary Care Meds (Dispening / Drugs)	All fields	DispensedPharmacyODSCode	The ODS code for the dispensing pharmacy
primary_care_meds	BSA Primary Care Meds (Dispening / Drugs)	All fields	DispensedPharmacyType	The type of the pharmacy eg community pharmacy
primary_care_meds	BSA Primary Care Meds (Dispening / Drugs)	All fields	EPSPrescriptionID	The EPS identifier of the prescription
primary_care_meds	BSA Primary Care Meds (Dispening / Drugs)	All fields	EPSPrescriptionIndicator	EPS Prescription Flag
primary_care_meds	BSA Primary Care Meds (Dispening / Drugs)	All fields	ExemptionCode	The code relating to the exemption category if exempt
primary_care_meds	BSA Primary Care Meds (Dispening / Drugs)	All fields	HighVolVaccineIndicator	Wehter the item is part of a high volume vacine
primary_care_meds	BSA Primary Care Meds (Dispening / Drugs)	All fields	ItemActualCost	The actual cost paid for the medication<br>Formula is<br>NIC - National Average Discount for the month + Out of Pocket + Consumables + Container allowance
primary_care_meds	BSA Primary Care Meds (Dispening / Drugs)	All fields	ItemID	The item identifier - relates to the prescription id (unique id along with prescription id)
primary_care_meds	BSA Primary Care Meds (Dispening / Drugs)	All fields	ItemNIC	The Net Ingredient Cost of the medicine
primary_care_meds	BSA Primary Care Meds (Dispening / Drugs)	All fields	MaternityExemptionFlag	Maternity Exemption Flag
primary_care_meds	BSA Primary Care Meds (Dispening / Drugs)	All fields	NotDispensedIndicator	Whether the item was dispensed
primary_care_meds	BSA Primary Care Meds (Dispening / Drugs)	All fields	OutOfHoursIndicator	Whether the prescription was Out of Hours
primary_care_meds	BSA Primary Care Meds (Dispening / Drugs)	All fields	PaidACBSIndicator	Whether the item is a boarderline substance
primary_care_meds	BSA Primary Care Meds (Dispening / Drugs)	All fields	PaidBNFCode	BNF code of the medicine reimbursed by BSA
primary_care_meds	BSA Primary Care Meds (Dispening / Drugs)	All fields	PaidBNFName	The name of the medicine/device reimbursed by BSA
primary_care_meds	BSA Primary Care Meds (Dispening / Drugs)	All fields	PaidCDIndicator	Whether the item is a controlled drug
primary_care_meds	BSA Primary Care Meds (Dispening / Drugs)	All fields	PaidDisallowedReason	What the reason for the dissallowed payment was
primary_care_meds	BSA Primary Care Meds (Dispening / Drugs)	All fields	PaidDissallowedIndicator	Whether the prescription was dissallowed by BSA, ie dispensed but not paid for by BSA
primary_care_meds	BSA Primary Care Meds (Dispening / Drugs)	All fields	PaidDrugStrength	The strength of the medicine reimbursed by BSA
primary_care_meds	BSA Primary Care Meds (Dispening / Drugs)	All fields	PaidFlavourIndicator	What the flavour of the item is
primary_care_meds	BSA Primary Care Meds (Dispening / Drugs)	All fields	PaidFormulation	The formulation of the drug reimbursed by BSA, e.g. Tablet, Capsules, oral suspension, etc
primary_care_meds	BSA Primary Care Meds (Dispening / Drugs)	All fields	PaidPADMIndicator	Whether the item can be personally administered by the patient
primary_care_meds	BSA Primary Care Meds (Dispening / Drugs)	All fields	PaidQuantity	The quantity reimbursed by BSA
primary_care_meds	BSA Primary Care Meds (Dispening / Drugs)	All fields	PaidSpecContIndicator	Whether the item is a special container
primary_care_meds	BSA Primary Care Meds (Dispening / Drugs)	All fields	PaidSupplierName	The name of the medicine/device manufacturer
primary_care_meds	BSA Primary Care Meds (Dispening / Drugs)	All fields	PaiddmdCode	The dm+d code of the medicine reimbursed by BSA
primary_care_meds	BSA Primary Care Meds (Dispening / Drugs)	All fields	PatientAge	The age of the patient at dispensing (calculated as per DoB)
primary_care_meds	BSA Primary Care Meds (Dispening / Drugs)	All fields	PatientGender	The administrative gender of the patient
primary_care_meds	BSA Primary Care Meds (Dispening / Drugs)	All fields	PrescribedBNFCode	BNF code of the medicine prescribed
primary_care_meds	BSA Primary Care Meds (Dispening / Drugs)	All fields	PrescribedBNFName	The name of the medicine/device
primary_care_meds	BSA Primary Care Meds (Dispening / Drugs)	All fields	PrescribedCountryCode	Country the prescription was prescribed in
primary_care_meds	BSA Primary Care Meds (Dispening / Drugs)	All fields	PrescribedFormulation	The formulation of the drug prescribed, e.g. Tablet, Capsules, oral suspension, etc
primary_care_meds	BSA Primary Care Meds (Dispening / Drugs)	All fields	PrescribedMedicineStrength	The strength of the medicine prescribed
primary_care_meds	BSA Primary Care Meds (Dispening / Drugs)	All fields	PrescribedQuantity	The quantity prescribed
primary_care_meds	BSA Primary Care Meds (Dispening / Drugs)	All fields	PrescribedSupplierName	The name of the medicine/device manufacturer
primary_care_meds	BSA Primary Care Meds (Dispening / Drugs)	All fields	PrescribeddmdCode	The dm+d code of the medicine prescribed
primary_care_meds	BSA Primary Care Meds (Dispening / Drugs)	All fields	PrescriberType	The prescriber type eg GP, Nurse prescriber, hospital doctor etc
primary_care_meds	BSA Primary Care Meds (Dispening / Drugs)	All fields	PrivatePrescriptionIndicator	Whether the prescription was a private prescription
primary_care_meds	BSA Primary Care Meds (Dispening / Drugs)	All fields	ProcessedPeriod	The month during which data is submitted by pharmacists to NHSBSA
primary_care_meds	BSA Primary Care Meds (Dispening / Drugs)	All fields	ProcessingPeriodDate	Processing Period 1st of Month
**SGSS**	**COVID-19 Second Generation Surveillance System (Beta version)**	-	-	**This contains the first positive test associated with covid-19 patients from Pillar 1 or Pillar 2**
SGSS	COVID-19 Second Generation Surveillance System (Beta version)	All Fields	Age_in_Years	The patient's DOB where known or patient's age
SGSS	COVID-19 Second Generation Surveillance System (Beta version)	All Fields	County_Description	The full postcode of the patient residence
SGSS	COVID-19 Second Generation Surveillance System (Beta version)	All Fields	Ethnicity_Description	Required under the Race Relations Amendment Act 2001
SGSS	COVID-19 Second Generation Surveillance System (Beta version)	All Fields	Lab_Report_Date	The date that the report was produced
SGSS	COVID-19 Second Generation Surveillance System (Beta version)	All Fields	Organism_Species_Name	CSF, blood, sputum, serum (where the diagnosis is based on serology, please use serum as the specimen type)
SGSS	COVID-19 Second Generation Surveillance System (Beta version)	All Fields	Patient_Sex	Male. Female and Not known
SGSS	COVID-19 Second Generation Surveillance System (Beta version)	All Fields	PostCode_Source	The full postcode of the patient residence
SGSS	COVID-19 Second Generation Surveillance System (Beta version)	All Fields	Reporting_Lab	Must always be specified, will usually be source lab, in some cases reference lab
SGSS	COVID-19 Second Generation Surveillance System (Beta version)	All Fields	Reporting_Lab_ID	If relevant
SGSS	COVID-19 Second Generation Surveillance System (Beta version)	All Fields	Specimen_Date	The date the specimen was collected from the patient. If this is not known, use the date the specimen was received at the source laboratory.
SGSS	COVID-19 Second Generation Surveillance System (Beta version)	All Fields	CDR_Specimen_Request_SK	The Specimen ID for the specimen associated to the subject
SGSS	COVID-19 Second Generation Surveillance System (Beta version)	All Fields	Lower_Super_Output_Area_Code	Lower Super Output Area
SGSS	COVID-19 Second Generation Surveillance System (Beta version)	All Fields	ODS_Location_Code	ODS Location Code
SGSS	SGSS DPS	All fields	pcds_sector	ONS Postcode (sector) attribute
**SUS PbR**	**Secondary Uses Service Payment By Results**	-	-	**Secondary Care data. Raw hospital episode data. Data dictionary found at: https://datadictionary.nhs.uk/all_items_index__a-z_/all_items__a.html#all_items_index__a-z_.all_items__a**
SUS PbR	Secondary Uses Service Payment By Results	All fields	ADMINISTRATIVE_CATEGORY_AT_START_OF_EPISODE	The ADMINISTRATIVE CATEGORY CODE at the start of the Hospital episode https://datadictionary.nhs.uk/attributes/administrative_category_code.html
SUS PbR	Secondary Uses Service Payment By Results	All fields	ADMINISTRATIVE_CATEGORY_ON_ADMISSION	The ADMINISTRATIVE CATEGORY CODE at the start of the Hospital Provider Spell. https://datadictionary.nhs.uk/attributes/administrative_category_code.html
SUS PbR	Secondary Uses Service Payment By Results	All fields	ADMISSION_METHOD_HOSPITAL_PROVIDER_SPELL	Used to derive Healthcare Resource Group 4 https://datadictionary.nhs.uk/data_elements/admission_method_code__hospital_provider_spell_.html
SUS PbR	Secondary Uses Service Payment By Results	All fields	AGE_AT_CDS_ACTIVITY_DATE	The age in years of the patient derived by the sender as at the relevant CDS activity date of the CDS type.
SUS PbR	Secondary Uses Service Payment By Results	All fields	AS_AT_DATE	The date (with an associated CDS Extract Time) of the update event (or the nearest equivalent) that resulted in the need to exchange this Commissioning Data Set.
SUS PbR	Secondary Uses Service Payment By Results	All fields	COMMISSIONER_REFERENCE_NO	Number (alphanumeric) allocated by the commissioner to a REFERRAL REQUEST.

SUS PbR	Secondary Uses Service Payment By Results	All fields	DIAGNOSIS_SCHEME_IN_USE	The type of CODED CLINICAL ENTRY used for the PATIENT DIAGNOSIS.
SUS PbR	Secondary Uses Service Payment By Results	All fields	DISCHARGE_DESTINATION_HOSPITAL_PROVIDER_SPELL	Used by the Secondary Uses Service to derive the Healthcare Resource Group 4.
SUS PbR	Secondary Uses Service Payment By Results	All fields	DISCHARGE_METHOD_HOSPITAL_PROVIDER_SPELL	Used by the Secondary Uses Service to derive the Healthcare Resource Group 4.
SUS PbR	Secondary Uses Service Payment By Results	All fields	END_DATE_HOSPITAL_PROVIDER_SPELL	The date a patient was discharged from an inpatient provider spell
SUS PbR	Secondary Uses Service Payment By Results	All fields	EPISODE_DURATION	Duration of episode
SUS PbR	Secondary Uses Service Payment By Results	All fields	EPISODE_DURATION_GROUPER	Duration of episode
SUS PbR	Secondary Uses Service Payment By Results	All fields	EPISODE_END_DATE	End date of episode
SUS PbR	Secondary Uses Service Payment By Results	All fields	ETHNIC_CATEGORY_CODE	The 16+1 ethnic data categories defined in the 2001 census is the national mandatory standard for the collection and analysis of ethnicity
SUS PbR	Secondary Uses Service Payment By Results	All fields	EXTRACT_DATE	Data extract date
SUS PbR	Secondary Uses Service Payment By Results	All fields	GENDER_CODE	The gender identity of a PERSON as stated by the PERSON.
SUS PbR	Secondary Uses Service Payment By Results	All fields	MAIN_SPECIALTY_CODE	A unique code identifying each MAIN SPECIALTY designated by Royal Colleges.
SUS PbR	Secondary Uses Service Payment By Results	All fields	MARITAL_STATUS	Code for marital status
SUS PbR	Secondary Uses Service Payment By Results	All fields	ORGANISATION_CODE_CODE_OF_COMMISSIONER	ORGANISATION CODE of the ORGANISATION commissioning health care.
SUS PbR	Secondary Uses Service Payment By Results	All fields	ORGANISATION_CODE_CODE_OF_PROVIDER	ORGANISATION CODE of the ORGANISATION acting as a Health Care Provider.
SUS PbR	Secondary Uses Service Payment By Results	All fields	ORGANISATION_CODE_SENDER	ORGANISATION CODE of the data sender
SUS PbR	Secondary Uses Service Payment By Results	All fields	PROCEDURE_SCHEME_IN_USE	The type of CODED CLINICAL ENTRY used for the CLINICAL INTERVENTION
SUS PbR	Secondary Uses Service Payment By Results	All fields	PROVIDER_REFERENCE_NO	The number convention agreed locally between a provider and Commissioner for use within a Commissioning Data Set message
SUS PbR	Secondary Uses Service Payment By Results	All fields	REFERRER_CODE	The code of the PERSON making the REFERRAL REQUEST.
SUS PbR	Secondary Uses Service Payment By Results	All fields	REFERRING_ORGANISATION_CODE	The ORGANISATION CODE of the ORGANISATION from which the referral is made, such as a GP Practice, NHS Trust or NHS Foundation Trust.
SUS PbR	Secondary Uses Service Payment By Results	All fields	REPORT_PERIOD_END_DATE	The date that a REPORTING PERIOD ends.
SUS PbR	Secondary Uses Service Payment By Results	All fields	REPORT_PERIOD_START_DATE	The date that a REPORTING PERIOD begins.
SUS PbR	Secondary Uses Service Payment By Results	All fields	SECONDARY_DIAGNOSIS_CODE_1 to 24	SECONDARY DIAGNOSIS (CODED CLINICAL ENTRY) is the CODED CLINICAL ENTRY used to identify the secondary PATIENT DIAGNOSIS.
SUS PbR	Secondary Uses Service Payment By Results	All fields	SITE_CODE_OF_TREATMENT_AT_START_OF_EPISODE	SITE CODE of treatment at the start of the episode
SUS PbR	Secondary Uses Service Payment By Results	All fields	START_DATE_HOSPITAL_PROVIDER_SPELL	The date the patient was admitted to a bed at the beginning of a provider spell.
SUS PbR	Secondary Uses Service Payment By Results	All fields	SOURCE_OF_ADMISSION_HOSPITAL_PROVIDER_SPELL	Source of admission to a Hospital Provider Spell in a Hospital Site.
SUS PbR	Secondary Uses Service Payment By Results Accident & Emergency	Birth and Age	AGE_AT_CDS_ACTIVITY_DATE	The age in years of the patient derived by the sender as at the relevant CDS activity date of the CDS type.
SUS PbR	Secondary Uses Service Payment By Results Accident & Emergency	Other Person Data	SEX	Sex of patient
SUS PbR	Secondary Uses Service Payment By Results Accident & Emergency	Treatments Block	PROCEDURE_SCHEME_IN_USE	The type of CODED CLINICAL ENTRY used for the CLINICAL INTERVENTION.
SUS PbR	Secondary Uses Service Payment By Results Episodes	Birth and Age	AGE_AT_CDS_ACTIVITY_DATE	The age in years of the patient derived by the sender as at the relevant CDS activity date of the CDS type.
SUS PbR	Secondary Uses Service Payment By Results Episodes	Birth and Age	EPISODE_AGE	A unique number or set of characters that is applicable to only one ACTIVITY for a PATIENT within an ORGANISATION.
SUS PbR	Secondary Uses Service Payment By Results Episodes	Consultant and Specialty	TREATMENT_FUNCTION_CODE	ACTIVITY TREATMENT FUNCTION CODE (DECISION TO ADMIT) is the TREATMENT FUNCTION CODE of the SERVICE to which a PATIENT is to be admitted.
SUS PbR	Secondary Uses Service Payment By Results Episodes	Consultant Episode - Activity	EPISODE_NUMBER	A unique number or set of characters that is applicable to only one ACTIVITY for a PATIENT within an ORGANISATION.
SUS PbR	Secondary Uses Service Payment By Results Episodes	Consultant Episode - Activity	OPERATION_STATUS	Operation status
SUS PbR	Secondary Uses Service Payment By Results Episodes	Consultant Episode - Activity	EPISODE_START_DATE	Start date of Episode
SUS PbR	Secondary Uses Service Payment By Results Episodes	GP and Referrer	GP_PRACTICE_CODE	GP practice code
SUS PbR	Secondary Uses Service Payment By Results Episodes	Procedures Block	PRIMARY_PROCEDURE_CODE	Primary procedure code
SUS PbR	Secondary Uses Service Payment By Results Episodes	Procedures Block	PRIMARY_PROCEDURE_DATE	Primary procedure date
SUS PbR	Secondary Uses Service Payment By Results Episodes	Procedures Block	SECONDARY_PROCEDURE_CODE_1 to 12	Secondary procedure codes
SUS PbR	Secondary Uses Service Payment By Results Episodes	Procedures Block	SECONDARY_PROCEDURE_DATE_1 to 12	Secondary procedure dates
SUS PbR	Secondary Uses Service Payment By Results Episodes	Provider and Commissioner	PROVIDER_SITE_CODE	Provide site code.
SUS PbR	Secondary Uses Service Payment By Results Episodes	Spell and Admission	PATIENT_CLASSIFICATION	A coded classification of PATIENTS who have been admitted to a Hospital Provider Spell.

Supplementary Table 7: Summary of codes included in the primary care (GP Data for Pandemic Planning and Research) Dataset

For more detail see https://digital.nhs.uk/coronavirus/gpes-data-for-pandemic-planning-and-research/guide-for-analysts-and-users-of-the-data#extract-scope-and-content)

Category of codes	Items included	Comments/notes
**COVID-19-related**	Diagnoses, tests, test results, vaccinations etc	
**Non-COVID diagnoses**	Cardiovascular disorders	including MI, angina, other coronary heart disease, hypertension, heart failure, congenital heart diseases, arrhythmias, other heart diseases, stroke, TIA, peripheral arterial diseases including aneurysms
	Diabetes	
	Cancers	
	Respiratory conditions	asthma, COPD, dust-related lung diseases, flu-like symptoms, other upper and lower respiratory tract infections, other respiratory diseases
	Renal diseases	
	Liver diseases	
	Neurological diseases	
	Dementia	
	Mental health conditions	depression, anxiety, schzophrenia, bipolar disorder and other psychoses, and others
	Autism and learning disability	
	Immunosuppression / immunodeficiency disorders	
	Musculoskeletal disorders	including osteoporosis and rheumatoid arthritis
	Falls and frailty	
	Others	thyroid disorders, organ transplants, pregnancy-related diagnoses, rare genetic, metabolic and autoimmune diseases
**Management, review and monitoring**	Lifestyle intervention and advice	diet, smoking, alcohol, exercise, other
	Cardiovascular diseases	
	Diabetes	
	Cancer	
	Asthma and COPD	
	Mental health conditions	
	Dementia	

	Falls	
	Epilepsy	
	Learning diasbility	
	Immunosuppressive treatments and procedures	radiotherapy, chemotherapy etc
	Palliative care	
	Medication monitoring	e.g. oral anticoagulants
	Rheumatoid arthritis	
**Observations and measurements**	Cardiovacsular disease risk assessment	e.g. Qrisk, Framingham score etc
	Cancer screening	
	Mental health screening	
	Heaight, weight and BMI	
	Blood pressure	
	Dementia assessment	
	Frailty assessment	
	Smoking status and alcohol consumption	
	Diabetic retinopathy and foot risk screening	
	Other retinal screening	
	Beathlessness measurement	
	Asthma symptoms	
**Vaccinations**	Influenza	
	Hep B	
	Haemophilus influanzae type B	
	Meningococcal	
	Measles, mumps, rubella	
	Pneurmococcal	
	Rotavirus	
	Shingles	

	Pertussis	
**Patient characteristics**	Ethnicity	
	Non-English language requiring interpreter	
	Care home and child/youth health worker codes	
**Medication prescriptions**	Statins and other cholesterol lowering medications	
	Antiplatelet and anticoagulant medications	
	Antihypertensive medications	ACE inhibitors, angiotensin receptor blockers, beta blockers, calcium channel blockers and others
	PSK9 inhibitors	
	Diabetes medications (oral and insulin)	
	Antipsychotic medications, including lithium	
	Asthma and COPD medications	
	Bone sparing agents / osteoporosis protection drugs	
	Epilepsy medications	
	Corticosteroids	
	Non-steroid immmunosuppressive medications	
	Smoking cessation medicatons	
	Constipation medications	
	Hypothyroidism medications	
**Laboratory and other measures**	Urea, creatinine and electrolytes	
	Other renal function lab measures (including urine tests)	
	Liver function tests	
	Glucose and HbA1C	
	Haemoglobin	
	Cholesterol and triglycerides	
	Thyroid function tests	
	Calcium	
	Hepatitis B	

	Spirometry and peak expiratory flow rate measures<br><br>DEXA results<br><br>Echocardiogram measures<br><br>Feet examination (neuropathy testing or peripheral pulses)	

Supplementary Table 8: Counts of Covid-19 cases in the new data resource compared with the Public Health England Covid-19 reports for 31 October 2020.

	**Covid laboratory test data**		**Hospitalised participants with Covid-19**		**Deaths with Covid-19 on the death certificate**	
	Definition	Number of unique participants	Definition	Number of unique participants	Definition	Number of unique participants
**Reported by Public Health England**	*Number of people with at least one positive Covid-19 test result (either lab-reported or lateral flow device), by (i) specimen date and (ii) date reported*[9]	(i) 904,105<br>(ii) 1,011,660	*Number of people admitted to hospital who tested positive for Covid-19 in the 14 days prior to admission, and those who tested positive in hospital after admission.*[10]	175,555	*Number of deaths of people whose death certificate mentioned Covid-19 as one of the causes*[11]	53,102†
**Counts from the new data resource**	Number of participants with a positive antigen test from Covid laboratory tests (i) with no restrictions and (ii) in the linked cohort‡	(i) 884,311<br>(ii) 776,503	Number of participants with a diagnosis ICD10 code (U07.1, U07.2, U071, U072) appearing in the hospital episodes (main or secondary diagnostic code position in HES-APC) in the linked cohort‡	126,349	Number of participants with a death registration with a mention of a diagnosis ICD10 code (U07.1, U07.2, U071, U072) in the linked cohort‡	50,504

†Reported for 30 October

‡Amongst 54.4 million participants, representing 96% of the population of England.

Supplementary Table 9. Summary of population based linked healthcare resources for England that cover a population of >5 million people and include primary care data as an anchoring component

Name of resource and website	Brief description	Population coverage of primary care data	Detail of primary care data available	Additional linked data available	Mechanism of access for researchers	Covid-19-related outputs	Approaches to data security and code sharing
OpenSAFELY https://opensafely.org/	New secure electronic health records (EHR) analytics platform created in March 2020 to deliver urgent results during the global COVID-19 emergency. A collaboration between the DataLab (University of Oxford), London School of Hygiene (LSHTM) EHR group, NHSX, and TPP, with more recent addition of EMIS, working on behalf of NHS England.	>58 million people registered with a practice using the TPP SystmOne or EMIS systems.	Comprehensive coded data from TPP and EMIS systems coded using a combination of data dictionaries including Read version 2, CTV version 3, and SNOMED-CT. Data flows in near real time.	Linked to hospital events (inpatient and outpatient SUS, A&E attendance data); hospital death from Covid-19; ONS death data including cause of death; ICNARC ICU data; SGSS covid test data; ISARIC cohort study. Able to rapidly map and link new datasets as required.	Access requests to team@opensafely.org for the pilot programme of external users. All published outputs to date involve the core Oxford/LSHTM team as lead authors.	Published outputs on: risk factors for Covid-19 death; effects on Covid-19 of respiratory conditions, HIV, various medications, living with children, ethnicity; monitoring the Covid-19 vaccine roll-out; health service impact of Covid-19; Covid-19 risk prediction.<br>See https://opensafely.org/research/	Novel security model with analytic code developed against synthetic data and then run against de-identified live data with no direct researcher access to person-level data. Only checked aggregate data can be exported.<br>All codelists are shared automatically at codelists.opensafely.org, and code for all analyses shared automatically at github.com/opensafely
QResearch https://www.qresearch.org/	Long established, ethically approved database derived from the anonymised health records from general practices using the EMIS clinical computer system. Data are held and accessed securely on servers at the University of Oxford, which acts as data controller.	>12 million people registered with a practice using the EMIS system[1]	Comprehensive coded data from EMIS system for contributing practices. Data 'regularly updated'.	Linked to cancer registry data, registered deaths, hospital episodes, ICNARC critical care data, Covid-19 test data, and pregnancy registry (with additional linkages planned).<br>See https://www.qresearch.org/data/	Access to academic researchers, subject to ethical committee and QResearch Scientific Committee approval. Researchers may need to seek approval for additional linked data. Almost all published outputs from 2004 include the core QResearch team as lead or co-authors.	Published outputs on: effects on Covid-19 of smoking, respiratory disease, antihypertensive medications, Down syndrome; predicting poor outcomes of COPvid-19 (QCovid); ethnic variation in vaccine uptake.<br>See https://www.qresearch.org/publications/research-papers/	Remote access to de-identified data within Oxford-based QResearch research environment.<br>Lay summaries of all approved research projects and some protocols in public domain.<br>See https://www.qresearch.org/research/approved-research-programs-and-projects/

Name of resource and website	Brief description	Population coverage of primary care data	Detail of primary care data available	Additional linked data available	Mechanism of access for researchers	Covid-19-related outputs	Approaches to data security and code sharing
Clinical Practice Research datalink (CPRD) https://www.cprd.com/home	Long established real-world data research service that collects and links anonymised patient data from a network of GP practices to support public health and clinical studies. Jointly sponsored by the Medicines and Healthcare products Regulatory Agency and the National Institute for Health Research (NIHR).	>16 million people currently registered with a participating general practice practices using the InPractice Systems Vision or EMIS systems	Comprehensive coded data from participating practices. Data regularly updated. See https://www.cprd.com/primary-care	Linked datasets currently available include: cancer registry data, registered deaths, hospital episodes, NHSD diagnostic imaging dataset, NHSD patient reported outcomes measures, NHSD mental health dataset, Covid-19 test data (with additional linkages planned). See https://www.cprd.com/linked-data	Access to data is subject to protocol approval by an Independent Scientific Advisory Committee (ISAC). No collaboration with CPRD team required. >2700 publications from 1988 have been enabled.	Published outputs on: excess deaths during Covid-19 pandemic in people with cardiovascular disease, cancer and other conditions; effects on mental illness and self-harm; indirect acute effects on physical and mental health; effects of obesity. See https://www.cprd.com/bibliography	CPRD does not operate a TRE; anonymised datasets are securely disseminated to researchers. Summaries of all approved studies in public domain. See https://www.cprd.com/protocol-list
Oxford-RCGP Research and Surveillance Centre https://www.rcgp.org.uk/clinical-and-research/our-programmes/research-and-surveillance-centre.aspx and https://orchid.phc.ox.ac.uk/index.php/orchid-data/	Long established source of information, analysis and interpretation of primary care data. Collects data from member general practices in England and transforms it into an accessible repository of data for health research.	>15 million people registered with a participating practice using the InPractice Systems Vision, TPP or EMIS systems	Coded data extracted by Apollo, Wellbeing Software. Currently >1000 theme variables featured themed dataset extracts curated using SNOMED-CT. Bespoke data extractions also available. See https://orchid.phc.ox.ac.uk/index.php/orchid-data/#theme-browser. Data updated weekly.	Linkage to NHS Digital data being sought to support Covid-19 vaccine effectiveness research	Researchers interested in conducting primary care/ linked data studies must provide a protocol, list of variables, and ethics approval. Monthly approval process in place for applications.	Published outputs on excess deaths during pandemic and effects of hypertension on Covid-19. Online observatory for Covid-19 test results; respiratory diagnoses; all-cause mortality; Covid-19 coding advice; further outputs forthcoming from involvement in several large Covid-19 randomised trials and observational studies. See https://orchid.phc.ox.ac.uk/	Secure, remote access to de-identified data within University of Oxford-hosted ORCHID TRE. Information on ongoing research studies and code lists in public domain. See https://orchid.phc.ox.ac.uk/

Name of resource and website	Brief description	Population coverage of primary care data	Detail of primary care data available	Additional linked data available	Mechanism of access for researchers	Covid-19-related outputs	Approaches to data security and code sharing
CVD-COVID-UK https://www.hdruk.ac.uk/projects/cvd-covid-uk-project/ and https://web.www.healthdatagateway.org/dataset/7e5f0247-f033-4f98-aed3-3d7422b9dc6d	New, secure access via national trusted research environments (TREs) to wide range of linked health data covering almost the entire populations of England (new NHS Digital TRE), Scotland and Wales (established TREs). This paper describes the new resource in England. Ethical and regulatory approvals cover England, Scotland and Wales.	~ 57 million people in England from >98% of English practices (all computer system suppliers), of whom >54 million were alive at the start of January 2020. (>65 million including approved access in Scotland and Wales)	Extensive NHS Digital GP Extraction Service extract of coded data provided in SNOMED-CT format (>34,000 SNOMED-CT concepts). NHS Digital currently receives data updates fortnightly. A more comprehensive primary care extract of all coded data to be updated daily will be in place during 2021	In England, linked to HES and SUS, registered deaths, community dispensed medicines and Covid-19 test data, with Covid-19 vaccination, hospital e-prescribing, ICNARC and multiple cardiovascular specialist audit datasets to follow. Similar linkages in Wales and Scotland. See https://www.hdruk.ac.uk/wp-content/uploads/2021/03/210304-CVD-COVID-UK-TRE-Dataset-Provisioning-Dashboard-1.pdf	Access for interested researchers to a rapid, streamlined project approvals process via https://web.www.healthdatagateway.org/dataset/7e5f0247-f033-4f98-aed3-3d7422b9dc6d or email bhfdsc@hdruk.ac.uk. Researchers join an open and transparent consortium and sign up to its principles (https://www.hdruk.ac.uk/wp-content/uploads/2020/07/CVD-COVID-UK-principles-for-website.pdf)	The present paper is our first output; having established the resource, we anticipate many more in the following weeks and months	Secure, remote access to de-identified data in NHS Digital TRE. Only checked aggregate data can be exported. Summaries of all ongoing projects, all analytic code, code lists and protocols in public domain. See https://www.hdruk.ac.uk/projects/cvd-covid-uk-project/ and https://github.com/BHFDSC

[1] taken from the most recent relevant published report from QResearch: Factors influencing COVID-19 vaccine uptake among minority ethnic groups https://assets.publishing.service.gov.uk/government/uploads/system/uploads/attachment_data/file/952716/s0979-factors-influencing-vaccine-uptake-minority-ethnic-groups.pdf
