# Supplementary material for: Linked electronic health records for research on a nationwide cohort of more than 54 million people in England: data resource
Source: BMJ. 2021 Apr 7;373:n826. doi: 10.1136/bmj.n826 (PMC8413899; doi:10.1136/bmj.n826)
Supplement: Supplementary file 2 — Supplementary information: Annexes 1 and 2 [file wooa065067.ww2.pdf]

Annexe 1: CVD-COVID-UK consortium members (those shaded yellow already in author list)

Institution	Member Name
**Addenbrooke's Hospital**	Jon Boyle
**British Heart Foundation**	Dan O'Connell
**British Heart Foundation**	Kate Cheema
**British Heart Foundation**	Naomi Herz
**British Heart Foundation**	Nilesh Samani
**British Heart Foundation**	Sonya Babu-Narayan
**European Bioinformatics Institute**	Ewan Birney
**European Bioinformatics Institute**	Moritz Gerstung
**Great Ormond Street Hospital**	Katherine Brown
**Health Data Research UK / BHF Data Science Centre**	**Cathie Sudlow**
**Health Data Research UK / BHF Data Science Centre**	Debbie Ringham
**Health Data Research UK / BHF Data Science Centre**	Jackie MacArthur
**Health Data Research UK / BHF Data Science Centre**	Lydia Martin
**Health Data Research UK / BHF Data Science Centre**	Lynn Morrice
**Health Data Research UK / BHF Data Science Centre**	Rouven Priedon
**Health Data Research UK**	Sinduja Manohar
**Health Data Research UK**	Susheel Varma
**Healthcare Quality Improvement Partnership**	Caroline Rogers
**Healthcare Quality Improvement Partnership**	Mirek Skrypak
**Imperial College London**	Alun Davies
**Imperial College London**	Safa Salim
**Imperial College London**	Sarah Onida
**Keele University**	Mamas Mamas
**King's College London**	Abdel Douiri
**King's College London**	Ajay Shah
**King's College London**	Ben Bray
**King's College London**	Charles Wolfe
**King's College London**	Elena Nikiphorou
**London School of Hygiene & Tropical Medicine**	Qiuju Li
**NHS Digital**	Brian Roberts
**NHS Digital**	Sam Hollings
**NHS England**	Deborah Lowe
**NHS Lanarkshire**	Mark Barber
**NHS Scotland**	Carole Morris
**NICE**	Adrian Jonas
**NICE**	Brett Doble
**NICE**	Felix Greaves
**NICE**	Jennifer Beveridge
**NICE**	Seamus Kent
**NICE**	Thomas Lawrence
**Office for National Statistics**	Ben Humberstone
**Office for National Statistics**	Myer Glickman
**Office for National Statistics**	Vahé Nafilyan

Institution	Member Name
**Queen's University Belfast**	Abdul Qadr Akinoso-Imran
**Queen's University Belfast**	Frank Kee
**Royal College of Surgeons of England**	David Cromwell
**Royal Papworth Hospital NHS Foundation Trust**	Florian Falter
**Swansea Bay University Health Board**	Daniel Harris
**Swansea University**	Ashley Akbari
**Swansea University**	Fatemeh Torabi
**Swansea University**	Gareth Davies
**Swansea University**	Hoda Abbasizanjani
**Swansea University**	Jane Lyons
**Swansea University**	Julian Halcox
**Swansea University**	Laura North
**Swansea University**	Libby Ellins
**Swansea University**	Mike Gravenor
**Swansea University**	Ronan Lyons
**Swansea University**	Rowena Griffiths
**University College London**	Alex Handy
**University College London**	Alvina Lai
**University College London**	Ami Banerjee
**University College London**	Ashkan Dashtban
**University College London**	Caroline Dale
**University College London**	Christopher Tomlinson
**University College London**	Eloise Withnell
**University College London**	Harry Hemingway
**University College London**	Honghan Wu
**University College London**	Johan Thygesen
**University College London**	Ken Li
**University College London**	Laura Pasea
**University College London**	Mehrdad Mizani
**University College London**	Michalis Katsoulis
**University College London**	Paula Lorgelly
**University College London**	Pedro Machado
**University College London**	Reecha Sofat
**University College London**	Rohan Takhar
**University College London**	Spiros Denaxas
**University of Aberdeen**	Mary Joan Macleod
**University of Bristol**	Deborah Lawler
**University of Bristol**	Jennifer Cooper
**University of Bristol**	Jonathan Sterne
**University of Bristol**	Livia Pierotti
**University of Bristol**	Massimo Caputo
**University of Bristol**	Neil Davies
**University of Bristol**	Rachel Denholm
**University of Bristol**	Rupert Payne
**University of Bristol**	Tom Palmer
**University of Bristol**	Venexia Walker
**University of Cambridge**	Angela Wood
**University of Cambridge**	David Brind
**University of Cambridge**	Emanuele Di Angelantonio

Institution	Member Name
**University of Cambridge**	Fabian Falck
**University of Cambridge**	Haoting Zhang
**University of Cambridge**	Howard Tang
**University of Cambridge**	Jessica Barrett
**University of Cambridge**	John Danesh
**University of Cambridge**	Mike Inouye
**University of Cambridge**	Samantha Ip
**University of Cambridge**	Spencer Keene
**University of Cambridge**	Tianxiao Wang
**University of Dundee**	David Moreno Martos
**University of Dundee**	Huan Wang
**University of Dundee**	Ify Mordi
**University of Edinburgh**	Annemarie Docherty
**University of Edinburgh**	Gwenetta Curry
**University of Edinburgh**	Tim Wilkinson
**University of Edinburgh**	William Whiteley
**University of Exeter**	John Dennis
**University of Glasgow**	Clea du Toit
**University of Glasgow**	Colin Berry
**University of Glasgow**	Sandosh Padmanabhan
**University of Leeds**	Jianhua Wu
**University of Leicester**	Anna Hansell
**University of Leicester**	Claire Lawson
**University of Leicester**	Francesco Zaccardi
**University of Leicester**	Kamlesh Khunti
**University of Leicester**	Tom Norris
**University of Liverpool**	David Hughes
**University of Liverpool**	Munir Pirmohamed
**University of Liverpool**	Ruwanthi Kolamunnage-Dona
**University of Manchester**	Craig Smith
**University of Manchester**	Maya Buch
**University of Oxford**	Ben Goldacre
**University of Oxford**	Ben Cairns
**University of Oxford**	Eva Morris
**University of Oxford**	George Nicholson
**University of Oxford**	Lucy Wright
**University of Oxford**	Nick Hall
**University of Oxford**	Olena Seminog
**University of Oxford**	Raph Goldacre
**University of Oxford**	Seb Bacon
**University of Strathclyde**	Amanj Kurdi
**University of Strathclyde**	Kim Kavanagh
**University of Strathclyde**	Marion Bennie
**University of Strathclyde**	Raymond Carragher
**University of Warwick**	Harry Wilde
**University Hospital of North Midlands**	Arun Pherwani
**Wellcome Trust**	Bilal Mateen

Annexe 2:
CVD-COVID-UK RECORD statement – checklist of items, extended from the STROBE statement that should be reported in observational studies using routinely collected health data.

	Item No.	STROBE items	Location in manuscript where items are reported	RECORD items	Location in manuscript where items are reported
**Title and abstract**					
	1	(a) Indicate the study's design with a commonly used term in the title or the abstract (b) Provide in the abstract an informative and balanced summary of what was done and what was found	Title and abstract	RECORD 1.1: The type of data used should be specified in the title or abstract. When possible, the name of the databases used should be included.<br><br>RECORD 1.2: If applicable, the geographic region and timeframe within which the study took place should be reported in the title or abstract.<br><br>RECORD 1.3: If linkage between databases was conducted for the study, this should be clearly stated in the title or abstract.	Title and abstract<br><br>Title and abstract<br><br>Title and abstract
**Introduction**					
Background rationale	2	Explain the scientific background and rationale for the investigation being reported	Introduction		
Objectives	3	State specific objectives, including any prespecified hypotheses	Introduction		
**Methods**					
Study Design	4	Present key elements of study design early in the paper	Methods (Data resources, Data processing and linkage), Figure1, Ta-		

			ble 1, Supplementary Figure 1		
Setting	5	Describe the setting, locations, and relevant dates, including periods of recruitment, exposure, follow-up, and data collection	Methods (Data resources, Data processing and linkage), Table 2		
Participants	6	*(a) Cohort study* - Give the eligibility criteria, and the sources and methods of selection of participants. Describe methods of follow-up<br>*Case-control study* - Give the eligibility criteria, and the sources and methods of case ascertainment and control selection. Give the rationale for the choice of cases and controls<br>*Cross-sectional study* - Give the eligibility criteria, and the sources and methods of selection of participants<br><br>*(b) Cohort study* - For matched studies, give matching criteria and number of exposed and unexposed<br>*Case-control study* - For matched studies,	Methods (Derivation of participant characteristic and disease diagnoses), Supplementary Tables 1-5	RECORD 6.1: The methods of study population selection (such as codes or algorithms used to identify subjects) should be listed in detail. If this is not possible, an explanation should be provided.<br><br>RECORD 6.2: Any validation studies of the codes or algorithms used to select the population should be referenced. If validation was conducted for this study and not published elsewhere, detailed methods and results should be provided.<br><br>RECORD 6.3: If the study involved linkage of databases, consider use of a flow diagram or other graphical display to demonstrate the data linkage process, including the number of individuals with linked data at each stage.	Methods (Derivation of participant characteristic and disease diagnoses), Supplementary Tables 1-5<br><br>Methods (Derivation of participant characteristic and disease diagnoses),<br><br>Table 2

		give matching criteria and the number of controls per case			
Variables	7	Clearly define all outcomes, exposures, predictors, potential confounders, and effect modifiers. Give diagnostic criteria, if applicable.	Methods (Derivation of participant characteristic and disease diagnoses)	RECORD 7.1: A complete list of codes and algorithms used to classify exposures, outcomes, confounders, and effect modifiers should be provided. If these cannot be reported, an explanation should be provided.	Methods (Derivation of participant characteristic and disease diagnoses), Supplementary Tables 1-5
Data sources/ measurement	8	For each variable of interest, give sources of data and details of methods of assessment (measurement). Describe comparability of assessment methods if there is more than one group	Methods (Derivation of participant characteristic and disease diagnoses)		
Bias	9	Describe any efforts to address potential sources of bias	Not applicable		
Study size	10	Explain how the study size was arrived at	Results, Table 2		
Quantitative variables	11	Explain how quantitative variables were handled in the analyses. If applicable, describe which groupings were chosen, and why	Not applicable		
Statistical methods	12	(a) Describe all statistical methods, including those used to control for confounding<br>(b) Describe any methods used to	Not applicable		

		examine sub-groups and interactions<br>(c) Explain how missing data were addressed<br>(d) *Cohort study* - If applicable, explain how loss to follow-up was addressed<br>*Case-control study* - If applicable, explain how matching of cases and controls was addressed<br>*Cross-sectional study* - If applicable, describe analytical methods taking account of sampling strategy<br>(e) Describe any sensitivity analyses			
Data access and cleaning methods		..		RECORD 12.1: Authors should describe the extent to which the investigators had access to the database population used to create the study population.<br><br>RECORD 12.2: Authors should provide information on the data cleaning methods used in the study.	Methods (Data resource access, CVD-COVID-UK Consortium, Data updates, Data security, privacy and confidentiality, Derivation of participant characteristics and disease diagnoses)<br><br>Methods (Derivation of participant characteristics and disease diagnoses)<br>Supplementary Figure 1 (notes)
Linkage		..		RECORD 12.3: State whether the study included person-level, institutional-level, or	Methods (Data processing and linkage)

				other data linkage across two or more databases. The methods of linkage and methods of linkage quality evaluation should be provided.	
**Results**					
Participants	13	(a) Report the numbers of individuals at each stage of the study (*e.g.*, numbers potentially eligible, examined for eligibility, confirmed eligible, included in the study, completing follow-up, and analysed)<br>(b) Give reasons for non-participation at each stage.<br>(c) Consider use of a flow diagram	Results (Demographic charactersitics and cardiovascular disease incidence, Covid-19 diagnoses),<br>Table 2	RECORD 13.1: Describe in detail the selection of the persons included in the study (*i.e.,* study population selection) including filtering based on data quality, data availability and linkage. The selection of included persons can be described in the text and/or by means of the study flow diagram.	Results (Demographic charactersitics and cardiovascular disease incidence, Covid-19 diagnoses),<br>Table 2
Descriptive data	14	(a) Give characteristics of study participants (*e.g.*, demographic, clinical, social) and information on exposures and potential confounders<br>(b) Indicate the number of participants with missing data for each variable of interest<br>(c) *Cohort study* - summarise follow-up time (*e.g.*, average and total amount)	Table 3<br><br>Table 3<br><br>Results (Demographic charactersitics and cardiovascular disease incidence, Covid-19 diagnoses),		

			Figure 2		
Outcome data	15	*Cohort study* - Report numbers of outcome events or summary measures over time<br>*Case-control study* - Report numbers in each exposure category, or summary measures of exposure<br>*Cross-sectional study* - Report numbers of outcome events or summary measures	Table 3, Figure 2, Figure 3		
Main results	16	(a) Give unadjusted estimates and, if applicable, confounder-adjusted estimates and their precision (e.g., 95% confidence interval). Make clear which confounders were adjusted for and why they were included<br>(b) Report category boundaries when continuous variables were categorized<br>(c) If relevant, consider translating estimates of relative risk into absolute risk for a meaningful time period	Table 3<br>Table 3<br>NA		
Other analyses	17	Report other analyses done—e.g., analyses of subgroups and interactions, and	Results (final paragraph), Table 3 Supplementary Table 6		

		sensitivity analyses			
**Discussion**					
Key results	18	Summarise key results with reference to study objectives	Discussion (Principal findings)		
Limitations	19	Discuss limitations of the study, taking into account sources of potential bias or imprecision. Discuss both direction and magnitude of any potential bias	Discussion (Strengths and limitations – final paragraph)	RECORD 19.1: Discuss the implications of using data that were not created or collected to answer the specific research question(s). Include discussion of misclassification bias, unmeasured confounding, missing data, and changing eligibility over time, as they pertain to the study being reported.	Discussion (Strengths and limitations – final paragraph)
Interpretation	20	Give a cautious overall interpretation of results considering objectives, limitations, multiplicity of analyses, results from similar studies, and other relevant evidence	Discussion (Conclusion)		
Generalisability	21	Discuss the generalisability (external validity) of the study results	Discussion (Strengths and limitations)		
**Other Information**					
Funding	22	Give the source of funding and the role of the funders for the present study and, if applicable, for the original study on which the present article is based	In Acknowledgements		
Accessibility of protocol, raw		..		RECORD 22.1: Authors should provide information on how	Methods (CVD-COVID-UK Consortium)

data, and programming code				to access any supplemental information such as the study protocol, raw data, or programming code.	

*Reference: Benchimol EI, Smeeth L, Guttmann A, Harron K, Moher D, Petersen I, Sørensen HT, von Elm E, Langan SM, the RECORD Working Committee. The REporting of studies Conducted using Observational Routinely-collected health Data (RECORD) Statement. *PLoS Medicine* 2015; 2:e1001885.

*Checklist is protected under Creative Commons Attribution (CC BY) license.
